# Research on quantitative evaluation of medical insurance fraud supervision policy based on ‘Antecedents-Process-Outcomes’ framework

**DOI:** 10.1371/journal.pone.0313618

**Published:** 2025-01-06

**Authors:** Zixiao Zhang, Shaoqun Ding, Zitao Yang, Huaxia Hu

**Affiliations:** 1 School of Public Administration, Southwestern University of Finance and Economics, Chengdu, Sichuan, China; 2 Aging and Social Security Research Center, Southwestern University of Finance and Economics, Chengdu, Sichuan, China; 3 School of Management, Wuhan University of Technology, Wuhan, Hubei Province, China; Kinnaird College for Women, PAKISTAN

## Abstract

Fraud in medical insurance is a serious problem that threatens the safety and sustainability of medical insurance funds. The process of reducing or even eliminating the impact of fraud is related to maintaining the balance of payments for medical insurance funds and reforming the payment system based on total amount control. As a result of reviewing the policy background of medical insurance fraud in China, combined with the policy evaluation model in the area of public management, this paper develops a conceptual framework of ’Antecedents-Process-Outcomes’ that emphasizes the fraud and governance of medical insurance funds. This paper uses grounded theory to look at 180 cases of medical insurance fraud and then uses the PMC index model to rate 18 policies. It then looks at the joint progressive analysis framework of medical insurance fraud and fraud supervision. In this paper, we analyze the policy similarities and differences of medical insurance fraud supervision in China from three perspectives: policy attributes, policy contents, and policy effects. The average PMC index of the 18 policies is 4.98, which is generally acceptable; however, there are some deficiencies in the policy field, policy supervision chain, policy orientation, and policy tools. Then, it puts forward suggestions for improving the four policy shortcomings in order to provide theoretical and practical enlightenment for the high-quality development of the medical security system and realize the new medical security in the process of Chinese-style modernization.

## Introduction

Medical insurance fraud is incompatible with weaving a tight network of medical insurance fund supervision in order to ensure the safe and sustainable use of medical insurance funds. Fraudulent medical insurance policies not only increase medical costs and reduce available funds for legal medical services but also damage the long-term solvency of medical insurance funds, adversely affecting the balance of payments for medical insurance funds and the overall process of implementing a total control payment system. The frequent occurrence of medical insurance fraud is attributed to the institutional endowment of medical insurance (the information asymmetry in the medical field and the third-party payment mechanism) [1]. Besides determining the necessity and legitimacy of modern medical insurance as a universal institution, these two characteristics also contribute to the medical insurance system’s high moral hazard and difficulty in supervised operations. In other words, there are several potential risk points for fraud in the collection, management, payment, and use of medical insurance fees. How to prevent the medical insurance fund from becoming ‘Tang Monk Meat’ and reduce the impact of the consequences of fraud needs to be strictly supervised and severely punished from the top-level design and specific operation of the fraud supervision policy [2].

The first administrative regulation on the supervision of the use of medical insurance funds in China, the Regulations on the Supervision and Management of the Use of Medical Insurance Funds (hereinafter referred to as the "Regulations") [3], was implemented on May 1, 2021. Approximately 53 relevant regulatory policies were issued over the next two years, involving a wide range of agencies and a wide range of laws and regulations. Specifically, the top-level design and specific operation of the medical insurance fraud supervision policy are involved. The policy attributes, policy contents, and policy effects are further analyzed, both as a concrete manifestation of the central government’s requirements and as an essential component of ensuring the sustainable development of the medical insurance industry. In parallel, in order to improve the governance capability and perfect the national governance system, the governance concept of fraud supervision must be deeply integrated into the safe use of medical insurance funds. It is necessary for the academic and practical circles to deeply analyze the institutional characteristics and institutional problems in the field of medical insurance fraud supervision in China, grasp the characteristics of medical insurance supervision in China, and develop a more targeted policy promotion mechanism.

Accordingly, this paper focuses on the conceptual analysis framework of medical insurance fraud (antecedent facts), fraud supervision policy (policy governance), and targeted collaborative governance (policy improvement). Based on a qualitative analysis of medical insurance fund fraud cases, this paper identifies the subjects, forms, and consequences of medical insurance fraud. A multidimensional evaluation index system based on ’fraud facts + fraud induction + policy evaluation’ is constructed through text mining of medical insurance fraud facts. Based on this system and the subsequent 18 policies, the PMC index model is used as a reference point. China’s medical insurance fraud supervision policy is evaluated quantitatively in this paper to determine its advantages and disadvantages, evaluate its policy attributes, policy contents, and policy effects, objectively sort out the policy, and provide constructive suggestions as to the next step in continuously optimizing the policy.

In addition to providing some references for the formulation of China’s medical insurance fund supervision policy and credit system construction, this study may widen the research horizon and research methods of related research on medical insurance fund supervision policy. It is of theoretical and practical significance to summarize the development trend and current characteristics of existing policies, reveal the shortcomings of policies, and seek policy breakthroughs.

## Literature review and research framework

### Literature review

Although the academic community have defined medical insurance fund fraud from the three levels of "broad, medium, and narrow" of "law enforcement practice, legislative context, and academic context" [4], the term "medical insurance fraud" is still considered a more mainstream term, which emphasizes the vertical integration of concepts as well as the severity of medical insurance fraud [5]. Some academics have different views on the concept of medical insurance fund fraud. It has been interpreted as the joint profit-making behavior of a number of stakeholders, including the supply of medical services, the demand for these services, and the intermediaries [6]. The academic community has produced a large amount of literature on mainstream status, influencing factors, direct and indirect losses, fraud factor identification, fraud detection, early warning model research, and big data technology applications, as well as prevention measures for medical insurance fraud based on the research topics of multi-stakeholders [7].

On the one hand, we examine the differences in the behavior of multi-stakeholder groups, including the characteristics that differ between individuals in terms of medical insurance fraud [8]. As for the grounded theory of Barney Glaser and Anselm Strauss, this paper attempts to find a middle ground between the explanatory public goods, information asymmetry theory, and the highly contextualized and relativistic actual fraud experience in order to construct an evaluation study that respects empirical facts and has some explanatory effect in evaluating the implementation effect of the middle-level theory policy, on the other hand. Generally, this type of evaluation is based on a comparison between the actual consequences of medical insurance fraud and the expected preventive measures [9]. These existing research results have laid a factual foundation for this paper to study the supervision policy of medical insurance funds on different scales, but few studies have qualitatively analyzed the logical coupling relationship between medical insurance fraud and medical insurance fraud supervision policy and further quantitatively evaluated the implementation effect of medical insurance fraud supervision policy.

The purpose of text mining is to derive potential knowledge from structured, semi-structured, or unstructured text data [10]. In the qualitative analysis dimension, grounded theory has previously been the main theoretical guide for text mining. The text plays a crucial role in observing and studying the connotation and quality of policy information, which includes multidimensional information such as policymakers, regulatory attributes, and organizational behavior norms [11]. Due to the rapid increase in texts in the public management discipline, the once popular policy analysis methodology has been gradually replaced by a new policy quantitative evaluation paradigm that is capable of strong text analysis and is scientific and effective [12]. The policy modeling consistency index (PMC-Index) method is based on the context of qualitative analysis. It takes the Omnia Mobilis hypothesis of Estrada et al. [13] as the guiding ideology, solves the pain points of unscientific automatic word segmentation and the heavy workload of manual word segmentation in previous quantitative analysis, and provides a multi-level analysis system and ideas for the surge of text policy information. In the fields of public management, population policy, and macroeconomics, it is widely used [3]. The literature related to medical insurance fund fraud basically discusses the causes of the consequences of medical insurance fund fraud from the perspective of text mining (qualitative analysis) [14], while few literatures discuss the implementation effect and quantitative evaluation of regulatory policies for medical insurance fund fraud.

### Research framework

#### Framework for conceptual analysis of ’Antecedents—Process—Outcomes’

**Fig 1:**
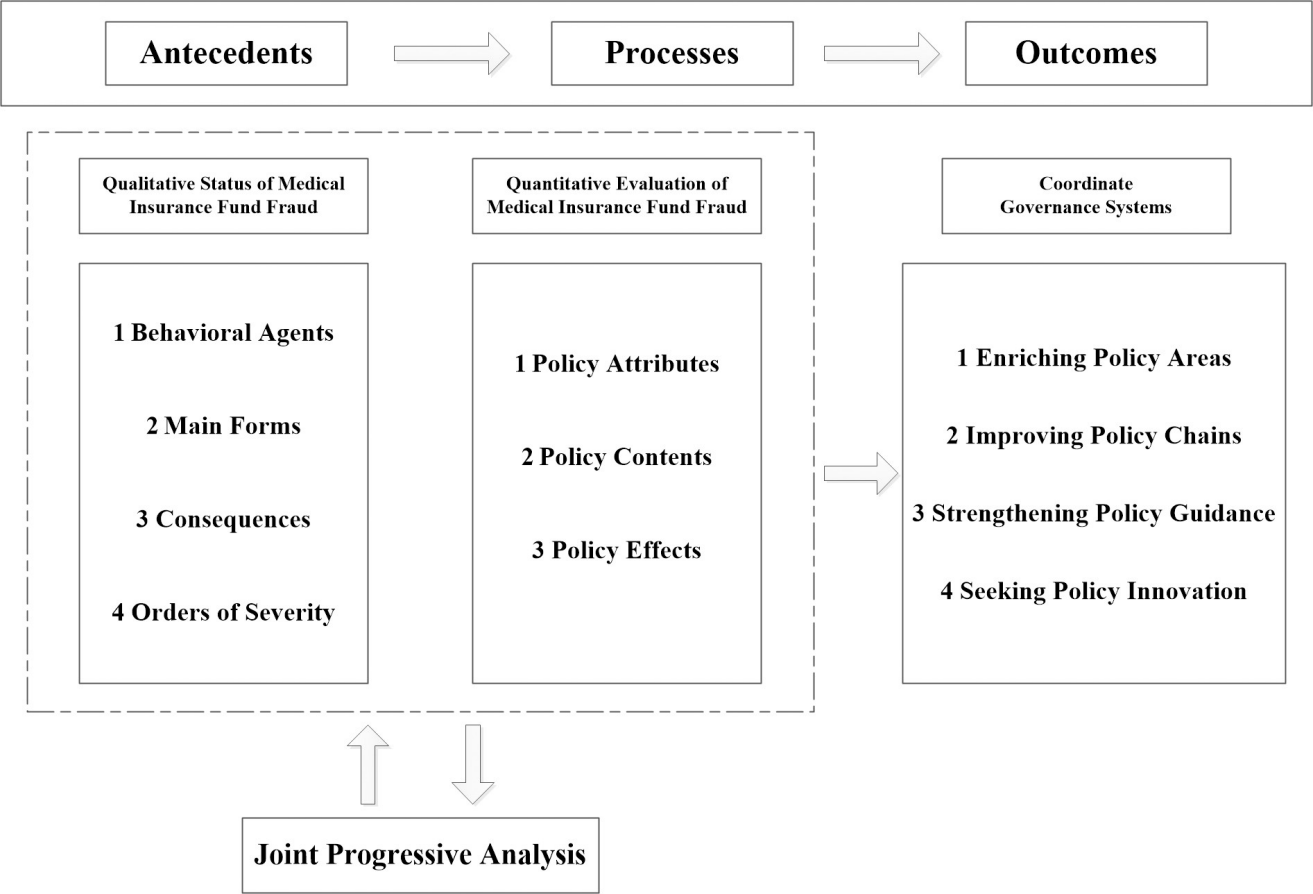
The three stages of conceptual analysis framework.

For those seeking to understand the governance process, social science research provides a wealth of knowledge. Public managers should examine the internal "black box" of policy participation in the governance of public issues [15]. As far as medical insurance fund fraud is concerned, such practical problems are rooted in the systems of public product theory and information asymmetry theory. The theoretical development, however, is limited by the fact that actual fraud behavior limits the effectiveness of public governance. Thus, it has become the consensus of the academic community to develop a ’theoretical discourse system’ with Chinese characteristics. As a result, this paper draws on Gray and Wood’s ’Antecedents-Process-Outcomes’ framework [16], a governance model of government policy participation in specific behaviors in public management, and combines the gradual concealment of medical insurance fraud and the complexity of risk points, while regulatory policies and laws lag behind the fraud facts [17, 18]. As part of this paper, we attempt to construct a conceptual analysis framework for the participation of medical insurance fund regulatory policies in the governance of medical insurance fund fraud, as shown in Fig 1.

Antecedents play an important role in assessing the practical experience and factual context of medical insurance fraud. Process is used to characterize the process by which government departments and medical institutions comply with rule constraints. Outcomes demonstrate the realization of policy objectives, how persistent problems in policy management are transformed into ’socially embedded’ relationships, and how autonomous collective behavior has been used to resolve problems related to institutional supply, commitment, and supervision. In order to avoid ambiguity, refer to the idea of high interdependence between policies [19], the demand for medical resources and fund risk sharing [20], and the solution of complex problems [21]. Here, Antecedents-Process-Outcomes is adjusted to ’antecedent facts-governance policies-promotion suggestions’ that match the governance of medical insurance fraud, which is complementary to the mechanism and path process of medical insurance fund supervision policies participating in the governance of fraud in the governance stage, reflecting each other, and reflecting the construction of medical insurance credit system with Chinese characteristics in the summary and promotion stage [22].

#### Application of the progressive analysis framework to medical insurance fraud and medical fund supervision

The theoretical construction and institutional supervision of the National Healthcare Security Administration have continuously improved the governance capacity to prevent and resolve medical insurance fraud with the popularization of ’digital intelligence economic background’ and ’big data technology’ [23]. However, in its actual implementation, there is always a problem of ’fragmentation’ of supervision. In essence, it can be understood that the loopholes of "institutional" and "regulatory" lead to serious problems in the four aspects of the underlying logic of fraud; social and economic motivation behind fraud, failure of regulatory authorities to supervise fraud, and the optimal utilization of regulatory resources.

Further, the institutional and regulatory shortcomings of the medical insurance fund are in opposition to the comprehensive governance model of "long-term stability" of the medical insurance fund. On the one hand, the inadequacies of the policy supervision system design at different levels and the lack of a linkage and coordination mechanism between the various organizational departments contribute to unsatisfactory supervision results. On the other hand, based on the idea of organizational system theory, the medical insurance fund supervision policy involves many elements that are difficult to couple and match to achieve the ’1 + 1 > 2’ collaborative governance effect, which limits the function of local department supervision and central overall supervision as a whole [24]. Therefore, the perspective of these two dimensions also fits the characteristics of ’policy governance’.

It is inevitable that the established tripartite relationship of medical services will suffer from deficiencies of ’humanity, regulation’, and ’institution’ in the use and supervision of medical insurance funds. In spite of this, the existing research is fragmented and unable to describe in a comprehensive and clear manner the ’ancient and modern’ regulatory policies of medical insurance funds. Thus, it is an urgent problem to determine the path of medical insurance fund fraud governance through the analysis of the practical logic of the regulatory policies of medical insurance funds [25].

Based on the existing research framework of "Antecedents-Process-Outcomes," this paper constructs a progressive research framework of "case grounded + quantitative evaluation" based on the individual behavior of medical insurance fraud [26, 27]. In this paper, a grounded theory and PMC index model are used to analyze the "practical origin" and "implementation effect" of the medical insurance fund supervision policy using the national medical insurance fraud and the corresponding medical insurance supervision policy as the original materials. Based on the evaluation results of the medical insurance fund supervision policy, this paper discusses the shortcomings of the medical insurance fund supervision policy. The purpose of this paper is to provide theoretical suggestions and practical references for improving the effectiveness of China’s medical insurance fund supervision system.

## Analysis of the forms, consequences, and causes of medical insurance fraud

It is necessary to sort out and summarize the practice context of frequent medical insurance fraud in order to investigate the setting logic of the medical insurance fraud supervision policy [25]. This section focuses on the antecedent fact of medical insurance fraud, extracts the synopsis of the original data of the case through case coding, refines the main form and consequences of fraud, forms a social semantic network map, and sorts out the focus of policy supervision on medical insurance fraud.

### Case text mining and extraction

The cases studied in this part are based on the five batches of medical insurance fund fraud cases exposed by the National Healthcare Security Administration from 2018 to 2023. In addition, this paper uses different methods, like semantic mining and web crawlers, to improve text information and policy meanings [28]. It does this by looking at cases provided by the National Healthcare Security Administration and screening out 180 common typical cases of fraud against medical insurance funds. This paper describes the key words for medical insurance fund fraud using the behavior analysis framework of "fraud subject + fraud form + fraud consequence." This sets the stage for the next step, which is a more in-depth look at medical insurance fraud supervision policy evaluation [29].

**Fig 2:**
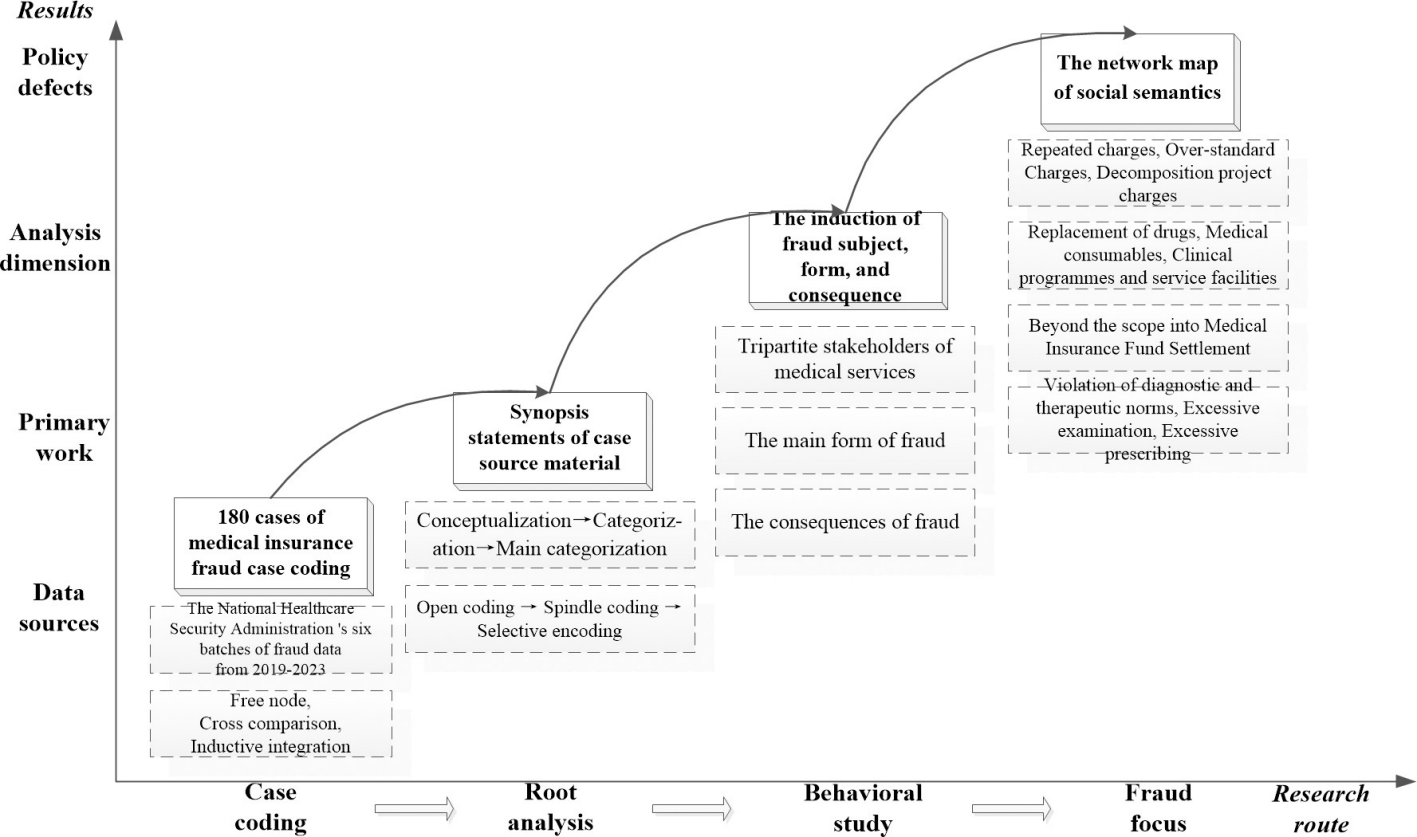
Four stages of qualitative analysis of medical insurance fraud.

Based on the word segmentation semantic library of NVivo 12 Plus software, combined with the professional vocabulary information in the field of medical insurance supervision, this section manually sorts out the word segmentation standards and preliminary processes the selected 180 cases. The processing flow is as shown in Fig 2, which is embodied in: extracting useful information from the original synopsis statement; establishing free nodes; cross comparison; and inductive integration; that is, after completing the open spindle and selective coding, the following 13 main categories are obtained: B1-B3 fraudulent behavior agents of medical services (providers, operators, and demanders); B4-B8 fraudulent main forms (false healthcare, illegal settlement, abuse of authority, ineffective supervision, unreasonable charge); and B9-B13 fraudulent consequences (suspension of eligibility, transfer to relevant departments, imposing criminal punishment, imposing warning punishment, imposing economic punishment). Based on the 13 basic concepts, a more abstract spindle concept category is summarized.

Based on the discussion above, this section focuses on ’Medical insurance fund fraud’ as the core category, which can be further dissected into the fraud subject, the fraud form, and the fraud consequence. The above coding content provides a reference for the subsequent analysis of the subject and cause of fraud, illustrated in Table 1.

**Table 1:** Coding context of qualitative analysis.

Main categorization	Categorization	Conceptualization	Summary statement of the original data of the case
**A1 Fraudulent Behavior Agent**	B1 Medical service providers	C11 Fixed-point clinic C12 Designated hospitals C13 Medical personnel	Ca11 ’A stomatological clinic in Wuhan was settled by Jinjiang Xinglian Shengbang stomatological clinic in the period of suspension of medical insurance service agreement.’ Ca12 ’A hospital in Dazhou City Sichuan Province induced patients to be hospitalized to defraud medical insurance funds.’ Ca13 ’The lax audit of patients by ultrasound doctors in a hospital in Fuzhou has led to fraudulent medical treatment.’
	B2 Medical Service Operators	C21 Staff of health care institutions	Ca21 ’Li, a staff member of the Medical Security Bureau, used the authorization to allocate a large amount of medical insurance fund to the personal accounts of relatives and friends ’ medical insurance cards.’
	B3 Medical service demanders	C31 Personal use C32 Embezzlement by others	Ca31 ’The insured Chen intentionally fabricated to conceal traffic accidents and third-party liability, illegal reimbursement of medical expenses of CNY 10,028.’ Ca32 ’Wu took the injured employees to a designated hospital in Xindu District for medical treatment, concealed the injury and let the employees use their medical insurance card.’
**A2 Fraudulent Main Forms**	B4 False healthcare	C41 False record of drugs C42 Fictional proof C43 Fictional diagnosis and treatment	Ca41 ’Huzhou Nanxun Jiu-an elderly disposable acupuncture needle and acupoint application treatment paste out of the library number and the number of medical insurance use seriously inconsistent and can not provide a reasonable explanation.’ Ca42 ’Deng, a staff member of the health center, forged outpatient invoices and prescription notes after stealing blank invoices.’ Ca43 ’There are some irregularities in a hospital in Taizhou, such as hanging up in bed and admitting insured persons who do not meet the admission indications.’
	B5 Illegal settlement	C51 Repeated reimbursement C52 People and medical insurance card does not match	Ca51 ’The insured Wang deliberately lied about the loss of invoices, concealed the fact that medical expenses had been reimbursed in Wenzhou, and repeatedly reimbursed to defraud the Fuzhou Medical Insurance Fund.’ Ca52 ’Xue fraudulently used other people ’s medical insurance card to overprescribe drugs for the treatment of hypertension and other diseases.’
	B6 Abuse of authority	C61 Assisting others to use medical insurance qualification C62 Misappropriation of medical insurance funds	Ca61 ’Sun, a joint orthopedic surgeon in a hospital in Kunshan City, assisted others to fraudulently use the social security card of the insured to defraud the medical insurance fund of CNY 25,400.’ Ca62 ’Yang, the former head of the financial statistics department of Pu ’er Medical Insurance Center, is suspected of misappropriating medical insurance funds.’
	B7 Ineffective supervision	C71 Non-standard medical practice C72 Poorly controlled drugs	Ca71 ’Assistant physician Zhang independently carried out diagnosis and treatment activities, resulting in the occurrence of non-licensed physician upload fees.’ Ca72 ’The doctor prescribes medicine at will.’
	B8 Unreasonable charge	C81 Noting more costs C82 Recording false fees	Ca81 ’A hospital in Yaodu District of Linfen City defrauded the medical insurance fund of 657,100 yuan through multi-billing of physiotherapy projects.’ Ca82 ’A hospital in Changping District has falsely reported the cost of insured personnel in different places.’
**A3 Fraudulent Consequences**	B9 Suspension of eligibility	C91 Suspension or disqualification of health insurance C92 Rescission of a licenses C93 Exempted from position	Ca91 ’According to the law, the medical insurance department suspended Chen ’s online settlement of medical expenses for 3 months.’ Ca92 ’Repealing the hospital ’s ’ medical institution practice license.’ Ca93 ’At the same time, the joint health department dismissed the president of the hospital.’
	B10 Transfering to relevant departments	C101 Transfering to health department C102 Transfering to the discipline supervision department C103 Transfering to Public Security Organs	Ca101 ’The hospital is beyond the scope of registration, etc., transferred to the health department for treatment.’ Ca102 ’Transfering case clues to the discipline inspection and supervision department for further processing.’ Ca103 ’To transfer the case materials and clue information of Ma ’s alleged fraud and defraud of medical insurance fund to the public security organ for processing.’
	B11 Imposing criminal punishment	C111 Punishment for fraud C112 Punishment for corruption C113 Punishment for misappropriation of public funds	Ca111 ’Xishui County People ’s Court sentenced as follows: Duan committed insurance fraud, several crimes and sentenced to fixed-term imprisonment of one year and three months, and a fine of 10,000 yuan.’ Ca112 ’Mengzi People ’s Court found that Li Yan committed the crime of corruption sentenced to four years in prison.’ Ca113 ’PuYa city medical insurance center former financial statistics section chief Yang misappropriation of public funds of RMB 94783689 yuan constitute the crime of misappropriation of public funds, sentenced to 2 years in prison, suspended for 2 years.’
	B12 Imposing warning punishment	C121 Asking for rectification within a time limit C122 Deducting physician points	Ca121 ’Huzhou city medical security department to make the following: interview the unit head, give a written warning and ordered rectification within a specified period of time.’ Ca122 ’Four doctors in the hospital, such as Wu, were given a deduction of 4 points, and two doctors, such as Zhou, were given a deduction of 8 points.’
	B13 Imposing economic punishment	C131 Recovery of health insurance funds C132 Refusing to pay the medical insurance fund C133 Imposing a penalty	Ca131 ’Quanzhou Medical Insurance Department recovers 74,654.79 yuan of medical insurance fund defrauded by Li et al.’ Ca132 ’Refusing to pay the village clinic ’s general medical expenses of 13,500 yuan in 2018.’ Ca133 ’Two times the fine of 70.28 million yuan for the charging department of the hospital due to the serial exchange project.’

### An analysis of the fraudulent forms and consequences of medical insurance funds

The following are some of the most common forms of medical insurance fund fraud: According to the frequency of occurrence of entries, they are arranged in the following order: inadequate supervision, illegal settlements, abuse of power, unreasonable charges, and false medical treatment. The proportion of each of the five types of fraud is approximately 20 percent, reflecting the diverse forms of medical insurance fund fraud. These five types of behaviors are mostly related to fund supervision policies, regardless of whether they are the result of poor supervision or illegal settlements. "Implementation Opinions of the State Council Office on Strengthening the Standing Supervision of the Use of the Medical Protection Fund No. 17" is an example. This document explicitly stipulates that doctors in designated medical institutions be supervised in advance and within the matter, and that supervision be strengthened in advance and within the matter in light of the non-standard practices of doctors under weak supervision.

As exemplified by a multi-agent conspiracy, medical insurance fraud is often complex rather than simple in real life. The subject of insurance fraud is composed of ’two-two combinations’ or ’three-three combinations." The main forms of fraud mainly include the ’doctor-patient joint fraud’ model based on interest bundling, mostly taking ’occupying hospital beds but no patients are actually hospitalized ’, exchange of medical insurance settlement diagnosis and treatment projects, false hospitalization, and other means. The most common are ’doctor-patient collusion’ and special ’reselling gangs’. In addition, there are a small number of internal personnel of medical insurance agencies who ’guard themselves’ and ’collude inside and outside." For example, the Department of Dermatology of the Third Affiliated Hospital of Guizhou Medical University conspired with patients to defraud insurance; doctors and village officials in Susong County, Anhui Province, conspired to defraud insurance; and the Third Affiliated Hospital of Anhui University of Traditional Chinese Medicine conspired with cardholders to defraud insurance.

**Fig 3:**
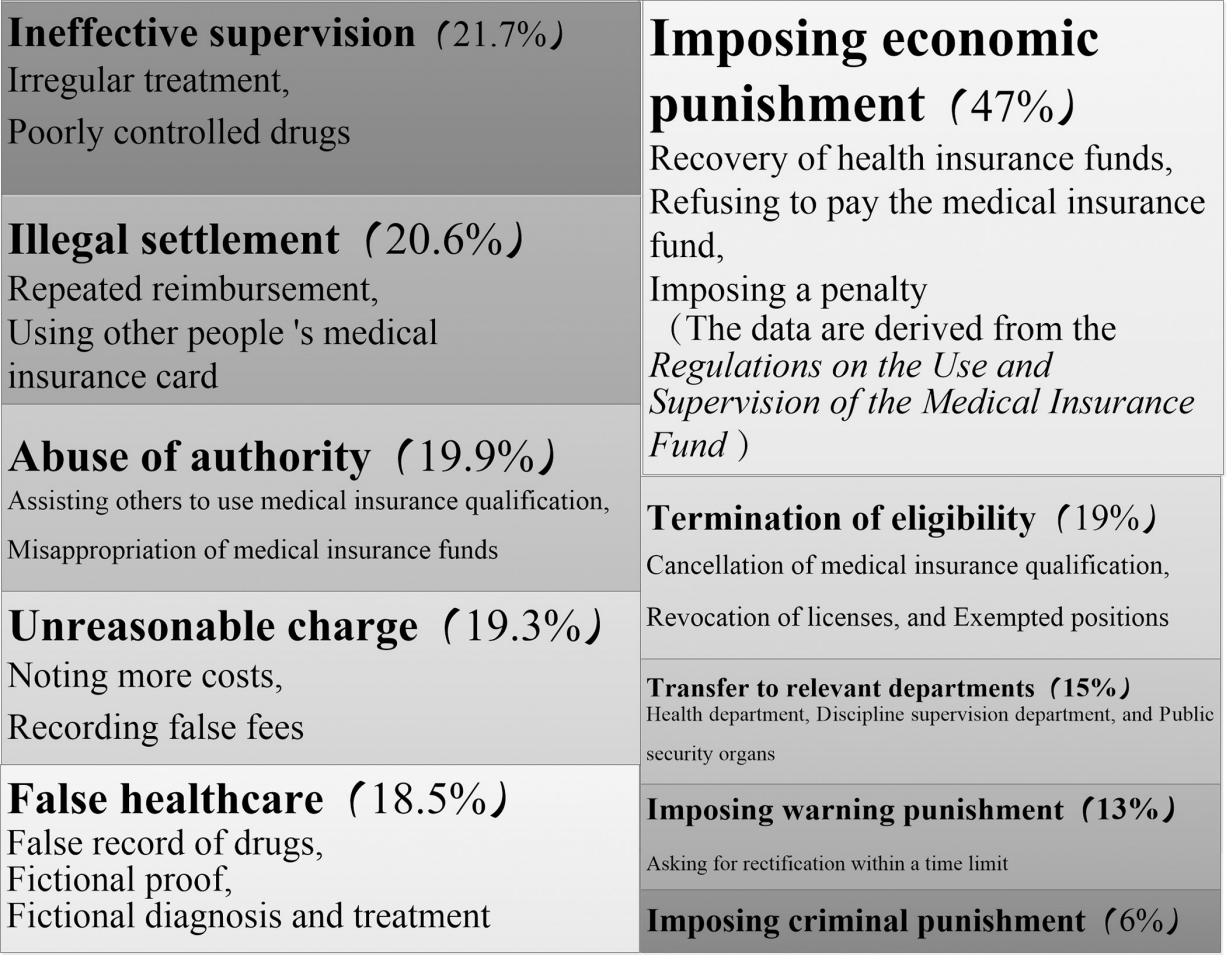
The forms and consequences of medical insurance fund fraud.

A hierarchical chart derived from the data analysis of NVivo 12 Plus software can visually illustrate the fraud consequences. The parent node nests more sub-level nodes, and as the number of sub-level nodes (sub-categories) increases, the area will also increase. Combined with the definition of B4-B8 sub-categories in A2, the frequency of fraud subjects in the synopsis statement corresponding to the original data is positively correlated with the cumulative area of the corresponding results. The final node area diagram is shown in Fig 3. It has two specific characteristics: (1) Economic punishment occupies the dominant position in administrative punishment. When revealing and adjudicating fraud, it is mainly implemented by recovering medical insurance funds, temporarily refusing to pay medical insurance funds, and imposing fines. (2) There are various ways of administrating punishment, which is almost the same as the proportion of economic punishment. (3) Criminal punishment occupies a very small proportion of nodes, and criminal punishment plays a very small role in fraud consequences, so it is difficult to deter fraud.

The above-mentioned measures for the punishment of medical insurance fraud reflect, on the side, the presence of certain omissions in the "after-action control" of the medical insurance fraud regulatory policy: The fraud in previous years relied largely on the correction of subsequent economic and administrative penalties, with no significant decrease in the amount of medical insurance fraud, indicating that the punishment was insignificant, that the "warning effect" of the penalties was weak, and that only 6 percent of the criminal penalties had a warning effect on medical insurance frauds. The National Medical Insurance Service has therefore consciously strengthened the link between "administrative punishment" and "criminal punishments" in recent years. Policies, such as notifications on strengthening the linkage in cases involving the investigation of fraudulent medical insurance funds (Table 2 P10), were developed.

**Table 2:** Summary of 18 medical insurance fund supervision policies from 2018 to 2023.

Numbering	Policy name	Document number	Release date	Publishing institutions
1	Notice on strengthening the management of medical insurance agreements to ensure fund security	No. 21 [2018]	November 28, 2018	Office of National Healthcare Security Administration
2	Notice on the issuance of the interim measures for reporting and rewarding fraudulent and deceptive acts against the medical insurance fund	No. 22 [2018]	November 27, 2018	Office of National Healthcare Security Administration, General Office of Ministry of Finance
3	Notice on the regulation of medical insurance funds for 2019	No. 14 [2019]	February 20, 2019	National Healthcare Security Administration
4	Notice of "two pilots and one demonstration" for the supervision of medical insurance funds	No. 17 [2019]	May 21, 2019	Office of National Healthcare Security Administration
5	Notice on the special management of the standardized use of medical insurance fund behavior of medical insurance designated medical institutions	No. 9 [2020]	June 2, 2020	National Healthcare Security Administration, National Health Commission
6	Guidelines for promoting the reform of the regulatory system of the medical insurance fund	No. 20 [2020]	July 9, 2020	The State Council office
7	Notice on the "review" of the specialized management of point-of-care medical institutions	No. 58 [2021]	December 17, 2020	Office of National Healthcare Security Administration, Office of National Healthcare Security Administration
8	Regulation on the supervision and management of the use of the medical insurance fund	No. 735	February 19, 2021	The State Council
9	Notice of the measures for regulating the discretionary power of administrative punishments in the supervision and management of the use of medical insurance funds	No. 35 [2021]	June 23, 2021	National Healthcare Security Administration
10	Notice on strengthening the work of bridging execution and punishment in investigating and dealing with cases of fraudulent use of medical insurance funds	No. 49 [2021]	November 26, 2021	National Healthcare Security Administration, Ministry of Public Security
11	Interim measures for supervision and management of the use of medical insurance funds and reporting	No. 5 [2022]	January 29, 2022	National Healthcare Security Administration
12	Notice of "illegal use of medical insurance fund reporting incentives"	No. 22 [2022]	November 17, 2022	Office of National Healthcare Security Administration, General Office of Ministry of Finance
13	Notice on launching flight inspection of medical insurance fund in 2022	No. 24 [2022]	May 27, 2022	National Healthcare Security Administration, National Health Commission, Ministry of Finance
14	Interim measures for the management of flight inspection of the medical insurance fund	No. 6 [2023]	April 21, 2023	National Healthcare Security Administration
15	Notice on the special rectification work on combating fraud and insurance fraud in the medical insurance field	No. 15 [2023]	April 21, 2023	National Healthcare Security Administration and other 7 departments
16	Guidelines on strengthening regular supervision of the use of medical insurance funds	No. 17 [2023]	May 26, 2023	The State Council office
17	Notice on launching flight inspection of medical insurance fund in 2023	No. 22 [2023]	July 14, 2023	National Healthcare Security Administration and other 4 departments
18	Notice on further promoting the intelligent audit and monitoring of medical insurance funds	No. 25 [2023]	September 8, 2023	National Healthcare Security Administration

The fraud of medical insurance funds is characterized by a number of social "gullies" between the "rationality" of medical insurance supervision and the "sensitivity" of personal economic value realization, as shown in Fig 3.

Therefore, it is important to improve the "rational depth" of supervision and gradually fill the short board of the medical insurance policy in order to prevent and resolve the fraud of medical insurance funds.

## An analysis of the fraud subject and behavior attribution in medical insurance fund

**Fig 4:**
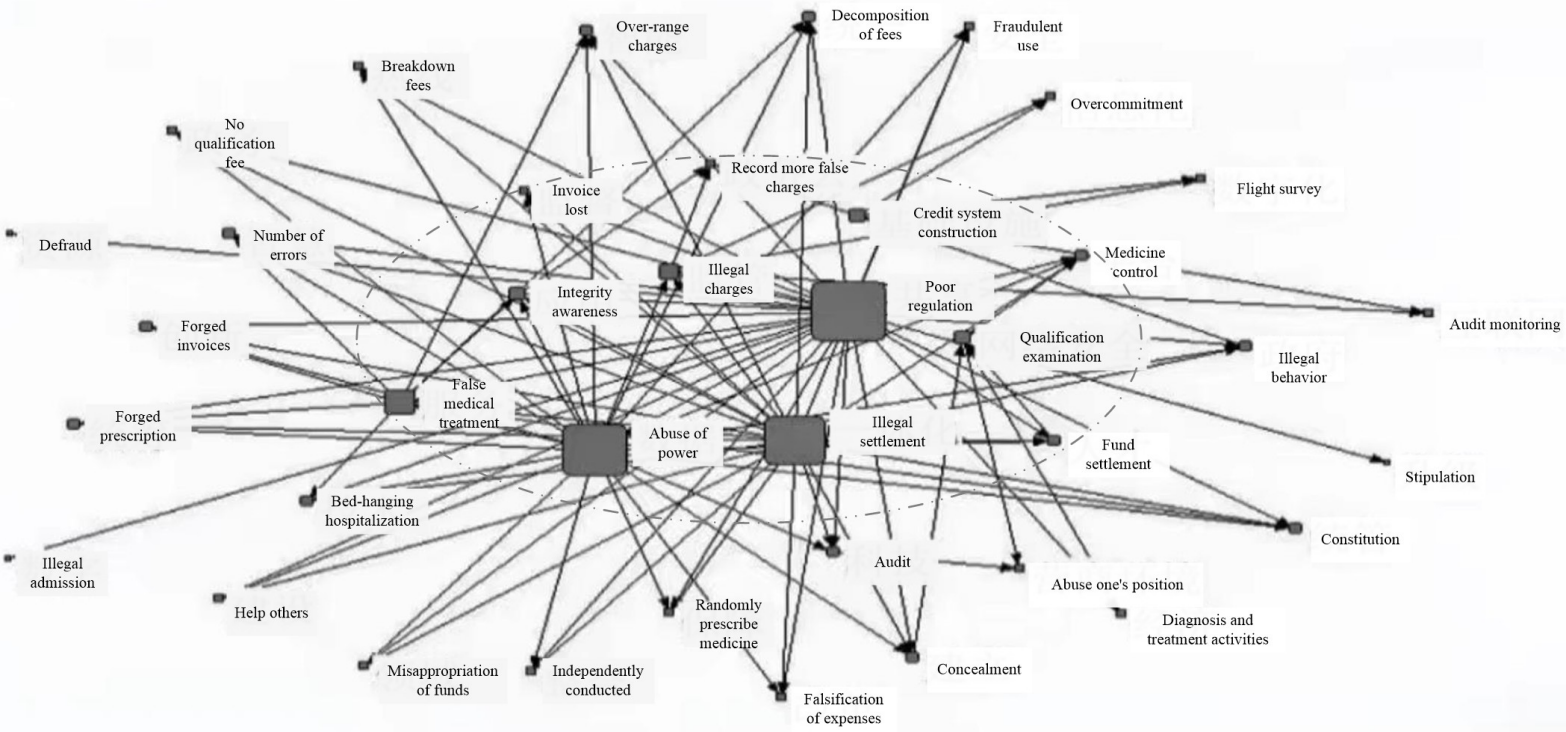
Social semantic network.

In order to index the keywords of medical insurance fund fraud, a social semantic network map is constructed based on word frequency analysis and coding. As can be seen in the Fig 4, the core keywords are supervision (ineffective), violation of rules and regulations (settlement), and abuse (authority). The three nodes with the highest density and centrality are indicative of the main characteristics of medical insurance fund fraud. There are several manifestations of fraud, including no qualification fee, breakdown fees, lax drug control, randomly prescribing medicine, misappropriation of funds, occupying hospital beds, and recording more false charges. The key words of the surrounding radiation: illegal behavior, flight inspections (surprise check), repeated audit, etc. reflect the main countermeasures in the progress of anti-fraud of medical insurance funds, while the fact that a large number of medical insurance funds have been abused requires that the anti-fraud of medical insurance funds needs to be further summarized for three types of illegal settlement, poor regulation, and abuse of power [30]. The reasons for the abuse of medical insurance funds are further summarized in order to provide a research basis for the following policy evaluation and conclusion recommendations.

**Illegal settlement** is reflected in some unreasonable or imperfect factors in the design of the medical insurance system, which leads to the occurrence of medical insurance fund fraud. There are several reasons for this: (1) The pricing, payment standards, and payment methods of medical service projects are unreasonable or unsuitable, providing medical institutions and individuals with space and motivation for illegal settlement; (2) The legal supervision level of medical insurance funds is insufficient, which makes it difficult to identify and effectively stop some illegal settlement behaviors at the appropriate time. (3) Social supervision and legal sanctions are not enough, which makes it difficult to curb and punish some illegal settlement behaviors. **Ineffective supervision** means that there are some deficiencies or defects in the functions and capabilities of the medical insurance regulatory agencies, which makes it difficult to detect and punish the fraud of the medical insurance fund. (1) The lack of personnel in the medical insurance department and backward technical means lead to the weakness of supervision capabilities; (2) The regulatory responsibilities of the medical insurance department are unclear and the regulatory measures are not effective, resulting in poor regulatory results; and (3) The medical insurance department has ineffective supervision power and an improper supervision attitude, which contributes to supervision dereliction. **Abuse of authority** refers to the behavior of medical insurance departments or individuals who use their positions to violate laws, regulations, or professional standards. Among the main reasons are: (1) the low moral level of the medical insurance department or individual, leading to psychological motivation for power abuse; (2) the excessive power of the medical insurance department or individual, which leads to an opportunity for power abuse; and (3) the low criminal risk cost of the medical insurance department or individual, resulting in a lighter consequence of power abuse.

Combining a summary of the semantic network of fraud forms with the three main reasons, it can be concluded that the characteristics of medical insurance fund fraud, such as rich fraud subjects, hidden fraud means, and numerous fraud links, are closely related to the problems encountered when medical insurance funds were illegally supervised [31, 32]. It is concluded that the illegal problems of medical insurance funds are mainly concentrated in three aspects: first, the illegal settlement behavior of medical institutions, such as repeated over-standard charges and serial drug exchange; second, the insured individual’s falsification of hospitalization, falsification of medical records, falsification of bills, and other ineffective supervision behavior. Third, the basic medical insurance government departments are responsible for the illegal use of funds, supervision, and other abuse of authority behavior. Referring to Estrada et al.’s ideas on policy evaluation, the above three main problems and causes are summarized, and it is found that it is necessary to start from the following three dimensions: policy attributes (insufficient legal supervision level, lack of social supervision in the policy field), policy contents (strengthening supervision, inspection, punishment, and audit monitoring for the three parties), and policy effects (strengthening incentives and reducing motivation). Specifically, policy attributes include publishing institutions, timeliness of policies, and policy fields; the policy contents are divided into policy objects, policy orientation, and policy priorities; and the policy effects cover policy tools, incentive measures, and policy evaluation. The above text mining content is an important basis for a subsequent progressive analysis of fraud policies.

### Quantitative analysis and evaluation of China ’s medical insurance fraud supervision policy

**Fig 5:**
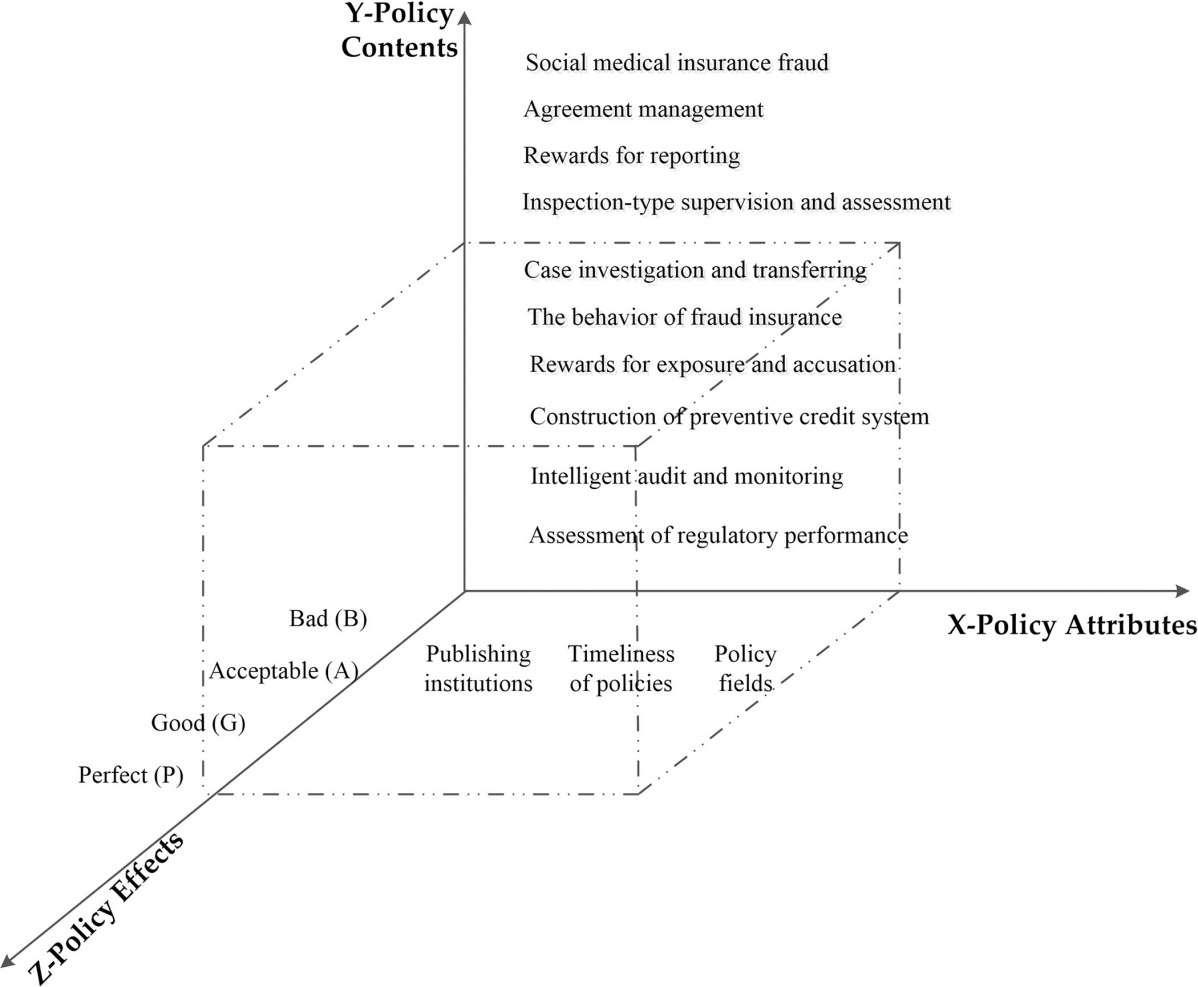
The three-dimensional attributes of medical insurance fraud supervision policy.

A number of policies and measures have been implemented for maintaining medical insurance funds’ security since the establishment of the National Healthcare Security Administration. Regulations, grams, and methods for obtaining medical insurance funds are gradually being improved. As a result of the research focus on medical insurance fraud identified by social semantic network maps [33], this section utilizes medical insurance fraud text mining content as an important basis for selecting regulatory policy indicators from a policy perspective and extracts the three-dimensional research attributes associated with regulatory policies on medical insurance fraud, as illustrated in the Fig 5.

This part focuses on the relevant policies on the supervision of medical insurance funds issued during the period from the establishment of the National Healthcare Security Administration on May 31, 2018 to September 20, 2023. It is collected from Laws & Regulations Database-China law info, the State Council’s policy document library, the National Healthcare Security Administration’s official website, and other appropriate platforms. It is combined with the existing medical insurance fund supervision policies. Relevant articles are screened. Policies are screened according to a number of criteria, including the department releasing the policy. In addition, the type of policy release and the timeliness of the policy. A higher level of release department allows the release category to better reflect the theme relevance and policy attitude, whereas a higher level of release timeliness prevents the situation of policies being revised, revoked, or deleted. In this study, the search policy was expanded based on key words including ’medical insurance’, ’fund’, ’supervision’, and ’fraud’. In addition, manual cleaning is used to remove less relevant policies. There are several examples of this, including stating the regulatory policy but not explicitly stating it, focusing on the description in other policies, and focusing too much on the implementation, as well as how the lower policy components are addressed. Based on the 68 relevant policies selected, this paper selected 18 policies with the highest correlation, prominent keywords, and obvious trends, as shown in Table 2.

Using a set of 18 policies for comparison, this part evaluates policies using the PMC index model proposed by Mario Arturo Ruiz Estrada [13]. It shows the relationship between each policy and specific variables. The level of the PMC index reflects the changing trend of various functions within and between policies. In both the surface chart and radar chart, each index change can be seen more clearly and contrasted more effectively. There have been numerous studies using the PMC index model in the fields of e-commerce, the digital economy, health care, innovation strategy, and other related aspects. These studies have led to the quantification and evaluation of massive text information contained in policy documents. Specifically, PMC analysis involves the mining of policy core texts, the construction and evaluation of evaluation index systems, the establishment of multi-input-output tables, the calculation and evaluation of PMC indexes, the drawing of the PMC surface, and the analysis of policy evaluation results.

### Policy text mining and extraction

The efficacy and rationality of evaluation indicators are dependent on the accuracy and relevance of policy text mining. Firstly, the author utilizes the text mining tool NVivo12 Plus to mine the main contents of 18 medical insurance fraud-related policies and provides keyword frequency and target sentence support for the construction of the next evaluation index system [34]. Words with a frequency greater than 0.5 are then selected through the use of word segmentation queries, word frequency settings, vocabulary filtering, and other methods. Meanwhile, words with semantic repetition are deleted, as are words without an obvious representational meaning. This is based on relevant expert opinion in the field of medical insurance. As shown in Table 3, we obtain the distribution table of the top 60 high-frequency subject words. As a result of the top-ranked keywords, increased emphasis has been placed on the manifestations of insurance fraud, the identification and punishment of medical insurance fraud, the incentives for disclosure and prosecution, and the construction of a credit system. It is also pertinent to note that the latter keywords convey a significant emphasis on the implementation of health care service providers, agencies, and policy measures. Intelligent auditing and monitoring, as well as healthcare expense settlement based on DRG (Diagnosis Related Groups) and DIP (Diagnosis-Intervention Packet), are also closely related to medical insurance fraud.

**Table 3:** High frequency word frequency distribution of subject words in medical insurance fund supervision policy text.

Subject word	Word frequency	Subject word	Word frequency	Subject word	Word frequency
Medical security fund	1126	Protocol	75	Emphasis	52
Inspection-type inspector assessment	1049	Organization	72	Region	51
Illegal punishment	675	Cases	72	Violation of regulations	51
Investigation and transfer of cases	313	In reference to	71	Propulsion	49
The behavior of insurance fraud	252	Administrative	65	Administration	49
Incentives for exposure and accusation	206	Service	65	Mode	49
Construction of preventive credit system	180	Medical establishment	65	Liability	47
Intelligent audit and monitoring	144	Pilots	65	Formulation	44
Defraud	140	Concern	61	Law enforcement	42
Robbery	131	Societies	60	Construction	42
False	124	Health	60	Insured	42
Violating the provisions	120	National Healthcare Security Administration	59	Demonstration site	42
Unreasonable	120	System	58	Drug	41
Misappropriation	114	Cost	57	Progression	41
Concealment	103	Special projects	55	Implementation	39
Establishment	100	Robbery	54	Provides	39
Correlation	90	Perfection	53	Safety	38
Medical treatment	78	Information	53	Requirement	38
Mechanism	76	Reinforcement	52	Settlement	38
Personnel	76	Robust	52	Governance	37

### The construction of evaluation index system and evaluation criteria

Estrada originally created the PMC index model based on Omnia Mobilis’ hypothesis that everything is in motion. A study of the empirical nature of policy modeling must take into account social problems in different time and spatial dimensions. This includes the heterogeneity between government policy documents and policies, as well as variables related to non-economic factors. Thus, based on the PMC index model proposed by Estrada, the present paper examines the results of previous text mining, combines the experience of domestic scholars regarding the selection of PMC indexes, considers the relevant documents on medical insurance fraud and fund supervision, and constructs the PMC index system based on these documents, including 9 first-level indicators and 47 second-level indicators [35–39]. Table 4 illustrates the assignment of 1 to the corresponding policy document if it contains the corresponding attributes of the level 2 indicators; otherwise, it is assigned 0. Specifically, the criterion is to determine whether the policy document uses tendentious text expressions or does not.

**Table 4:** PMC index variables, scoring criteria and index sources.

Evaluation dimension	Level-one variable	Level-two variable	Scoring standard	References of indicators
Policy attributes	X1 Publishing institutions	X1:1 State Council and its constituent departments	Whether the State Council and its constituent departments issued the policy, and mark 1 if they did, otherwise mark 0.	KuangB and HanJ (2020), LiuQ and JiaMT (2023), WangN and WangW (2022), ZhangSJ and YangST(2022)
		X1:2 Departments directly under the State Council	Whether an organization directly under the State Council issued the policy; mark 1 if so, otherwise mark 0.	
		X1:3 State Administration managed by the ministries and commissions of State Council	Whether the policy is issued by the State Bureau under the administration of State Council ministries and commissions, and mark 1 if yes; otherwise, mark 0.	
	X2 Timeliness of policies	X2:1 Long-term (more than 5 years)	Whether the policy is long-term (>5 years), mark 1 if yes, otherwise mark 0.	Estrada MAR (2011), DaiSL et al. (2023), LiuF and LiuZ (2023), LiuLM et al. (2023)
		X2:2 Mid-term (3–5 years)	Whether the policy is a medium-term (3–5 years) policy, and mark 1 if yes, otherwise mark 0.	
		X2:3 Short-term (1–3 years)	Whether the policy is a short-term (1–3 year) policy, and mark 1 if yes, otherwise mark 0.	
		X2:4 The same year	Whether the policy is within the same year, and mark 1 if yes, otherwise mark 0.	
	X3 Policy fields	X3:1 Economy	Whether the policy relates to the economic sphere, mark 1 if yes, otherwise mark 0.	EstradaMAR (2011), HuangGD et al. (2023), YangYL et al. (2022), ZhangXC and JiangM (2023), XiongYC and ZhangCL (2023)
		X3:2 Politics	Whether the policy covers the political sphere, and mark 1 if yes, otherwise mark 0.	
		X3:3 Society	Whether the policy is oriented towards the executive branch, and mark 1 if yes, otherwise mark 0.	
		X3:4 Technique	Whether the policy covers technical areas, and mark 1 if yes, otherwise mark 0.	
Policy contents	X4 Policy objects	X4:1 Administrative department	Whether the policy is oriented towards the administrative department, and mark 1 if yes, otherwise mark 0.	ZhaoY et al. (2022), HuangGD et al. (2023), LiYZ et al. (2022), MaXF et al. (2023), WangXJ et al. (2023)
		X4:2 Designated medical institutions	Whether the policy is geared towards designated medical institutions, and mark 1 if yes, otherwise mark 0.	
		X4:3 Designated retail pharmacies	Whether the policy is for designated retail pharmacies, and mark 1 if yes, otherwise mark 0.	
		X4:4 Medical insurance agency	Whether the policy is oriented towards medical insurance agency, and mark 1 if yes, otherwise mark 0.	
		X4:5 Insured person	Whether the policy is for the insured, and mark 1 if yes, otherwise mark 0.	
	X5 Policy orientation	X5:1 Prediction	Whether the policy is predictable, and mark 1 if yes, otherwise mark 0.	YangCR et al. (2022), ZhangGX et al. (2023), De Groote et al. (2015), LiZH et al. (2022)
		X5:2 Regulation	Whether the policy is regulatory, and mark 1 if yes, otherwise mark 0.	
		X5:3 Proposal	Whether the policy is suggestive, and mark 1 if yes, otherwise mark 0.	
		X5:4 Support	Whether the policy is supportive, and mark 1 if yes, otherwise mark 0.	
		X5:5 Description	Whether the policy is descriptive, and mark 1 if yes, otherwise mark 0.	
		X5:6 Guidance	Whether the policy is instructive, and mark 1 if yes, otherwise mark 0.	
	X6 Policy priorities	X6:1 Supervision and inspection	Whether the policy focus involves supervision and inspection, and mark 1 if yes, otherwise mark 0.	Text mining results, WangBJ et al. (2022), LiSQ et al. (2022), LiuYW et al. (2022), WeiXY et al. (2021)
		X6:2 Agreement management	Whether the policy focus involves agreement management, and mark 1 if yes, otherwise mark 0.	
		X6:3 Case investigation and transfer	Whether the policy focus involves case investigation and transfer, and mark 1 if yes, otherwise mark 0.	
		X6:4 Intelligent audit and monitoring	Whether the policy focus involves intelligent audit and monitoring, and mark 1 if yes, otherwise mark 0.	
		X6:5 Rewards for reporting	Whether the policy focus involves reporting rewards, and mark 1 if yes, otherwise mark 0.	
		X6:6 Illegal activities	Whether the policy focus involves illegal activities, and mark 1 if yes, otherwise mark 0.	
		X6:7 Regulating medical services	Does the policy focus on regulating medical services, and mark 1 if yes, otherwise mark 0.	
Policy effects	X7 Policy tools	X7:1 Command type	Whether the policy is command type, and mark 1 if yes, otherwise mark 0.	ZhangQ et al. (2023), LiYZ et al. (2021), LiuY et al. (2022), Rataj et al. (2018)
		X7:2 Normal type	Whether the policy is normal type, and mark 1 if yes, otherwise mark 0.	
		X7:3 Incentive-based type	Whether the policy is incentive-based type, and mark 1 if yes, otherwise mark 0.	
		X7:4 Capacity-building type	Whether the policy is capacity-building type, and mark 1 if yes, otherwise mark 0.	
		X7:5 System-variation type	Whether the policy is system-variation type, and mark 1 if yes, otherwise mark 0.	
		X7:6 Persuasive type	Whether the policy is persuasive type, and mark 1 if yes, otherwise mark 0.	
	X8 Incentive measures	X8:1 legal protection	Whether the incentive measures involve legal protection, and mark 1 if yes, otherwise mark 0.	ShengLY et al. (2023), WangH et al. (2022), JiangZZ et al. (2023)
		X8:2 System interface	Whether the incentive measures involve system interface, and mark 1 if yes, otherwise mark 0.	
		X8:3 Operating mechanism	Whether the incentive measures involve operating mechanism, and mark 1 if yes, otherwise mark 0.	
		X8:4 Departmental collaboration	Whether the incentive measures involve departmental collaboration, and mark 1 if yes, otherwise mark 0.	
		X8:5 Talent team	Whether the incentive measures involve talent team, and mark 1 if yes, otherwise mark 0.	
		X8:6 Assessment of regulatory performance	Whether the incentive measures involve assessment of regulatory performance, and mark 1 if yes, otherwise mark 0.	
	X9 Policy evaluation	X9:1 Sufficient basis	Whether the policy is based on the sufficient basis, and mark 1 if yes, otherwise mark 0.	LiuJZ et al. (2023), Lloyd-Sherlock et al. (2022), Zhang YK et al. (2023)
		X9:2 Clear aim	Whether the policy goal is clear, and mark 1 if yes, otherwise mark 0.	
		X9:3 Scientific scheme	Whether the policy is scientific, and mark 1 if yes, otherwise mark 0.	
		X9:4 Detailed planning	Whether the policy is planned in detail, and mark 1 if yes, otherwise mark 0.	

In the previous article, three aspects of the illegality of medical insurance funds were extracted from the types and forms of medical insurance fraud. These aspects are illegal settlement, poor supervision, and abuse of power. There is an explanation and discussion of the causes and problems associated with these three aspects. The three-dimensional analysis framework of the PMC index is constructed, and the first-level indicators and second-level indicators of policy evaluation are subdivided: the three first-level indicators of the publishing agency, policy timeliness, and policy field are summarized as the information characteristics that describe the policy attributes; the three first-level indicators of policy focus, policy orientation, and policy object are identified as the information characteristics reflecting the policy content; and the policy tools, incentives, and policy evaluation are summarized as the information characteristics that reflect the policy effect. The three dimensions include the timeliness of the policy, the political level of the publication agency, and the specific behavior of medical insurance fraud. In addition, it provides incentives for reporting, coordination capabilities within departments, and whether or not it involves multiple parties in the medical decision-making process. Considering the relevance of the policy text, it has been categorized into three dimensions of policy text analysis. This provides a basis for calculating classification indices and categorical analyses.

### Establishment of multi-input-output table

As shown in Table 5, the multi-input-output table presented in this paper can be used to assess the data analysis framework and the number of secondary indicators more intuitively. The policy evaluators’ understanding of a single variable is more systematic and multidimensional, making it easier to quantify each indicator and assign a single variable. Several experts in the field were consulted to avoid subjective errors when interpreting the indicators. According to Cohen’s Kappa test with SPSS, the final results were determined to be consistent, indicating that the scoring was adequate to meet the consistency requirements.

**Table 5:** Multi-input-output table.

Level-one variable	X1	X2	X3	X4	X5	X6	X7	X8	X9
**Level-two variable**	X1:1	X2:1	X3:1	X4:1	X5:1	X6:1	X7:1	X8:1	X9:1
	X1:2	X2:2	X3:2	X4:2	X5:2	X6:2	X7:2	X8:2	X9:2
	X1:3	X2:3	X3:3	X4:3	X5:3	X6:3	X7:3	X8:3	X9:3
		X2:4	X3:4	X4:4	X5:4	X6:4	X7:4	X8:4	X9:4
				X4:5	X5:5	X6:5	X7:5	X8:5	
					X5:6	X6:6	X7:6	X8:6	
						X6:7			

### Calculation and evaluation of PMC index

The following section presents three formulas for calculating PMC index in accordance with Estrada’s construction. To begin with, all variables resulting from scoring are imported into the table of multi-input-output variables. The evaluation criteria in Formula (1), Formula (2), and Table 4 are used to find the values of the second-level index variables. Formula (3) is then used to calculate and find the values of the different first-level index variables. The formula divides the total number of secondary variables by the sum of the secondary variables of each policy. This equals the Xt value. Finally, the sum of the Xt values of each first-level index is used in Formula (4) to calculate the PMC index value. Among them, Xt represents the sub-variable, X is the set of sub-variables, Xtj represents the value of the Jth sub-variable under the Tth main variable, and n = 9 is selected in this paper.

X∼N[0,1] (1)

X={XR:[0v1]} (2)

$$X_{t}{=X}_{t}(\sum_{j=1}^{T(X_{tj})}\frac{X_{tj}}{T(X_{tj})})t=1,2,3,4,5,\ldots ,\infty$$ (3)

$$PMC=\sum_{t=1}^{n}\left(X_{t}[\sum_{j=1}^{T(X_{tj})}\frac{X_{tj}}{T(X_{tj})}]\right)$$

$$=\left[X_{1}(\sum_{a=1}^{4}\frac{X_{1a}}{4})+X_{2}(\sum_{b=1}^{4}\frac{X_{2b}}{4})+X_{3}(\sum_{c=1}^{4}\frac{X_{3c}}{4})+X_{4}(\sum_{d=1}^{5}\frac{X_{4d}}{5})+X_{5}(\sum_{e=1}^{6}\frac{X_{5e}}{6})+X_{6}(\sum_{f=1}^{9}\frac{X_{6f}}{9})+X_{7}(\sum_{g=1}^{6}\frac{X_{7g}}{6})+X_{8}(\sum_{h=1}^{6}\frac{X_{8h}}{6})+X_{9}(\sum_{i=1}^{4}\frac{X_{9i}}{4})\right]$$ (4)

According to the above calculation, we are able to arrive at the PMC index of 18 policies in Table 6. In general, a higher PMC index indicates that the policy content is more comprehensive and more influential. In contrast, a higher depression index indicates that some indicators of the policy score are lower and the policy is not yet complete. According to the evaluation criteria of scholars and the existing research situation, this paper determines the policy of PMC index in 0 ~ 3.99 as a bad (B) consistency policy, the policy of PMC index in 4 ~ 5.99 as an acceptable (A) policy, the policy of PMC index in 6 ~ 7.99 as a good (G) policy, and the policy of PMC index in 8 ~ 9 as a perfect (P) policy. Based on Table 6, three policies have high consistency, namely policy P6, policy P8, and policy P18. There are ten policies with high consistency and five policies with low consistency. In general, there are only three policies with good consistency, but the proportion of policies with good consistency has exceeded half, which indicates that some policies still have room for improvement in influencing and reducing medical insurance fraud, and some policies still have room for improvement. There is a relatively low score for P5 and P7 in the lack of consistency policy. As a result of policy P7, policy P5 has been consolidated and strengthened, and the effect test has been strengthened as well. P5 and P7 are policy documents of a special governance nature, which is the principal reason for their low scores. Neither the scope nor the time span of the radiation policy are extensive. Less attention is paid to the prevention and control of the problem at the source, as well as specific implementation measures. Following are some of the more distinctive characteristics of the relevant policies that will be examined in more detail.

**Table 6:** PMC index and depression index ranking and evaluation of 18 policies.

Evaluation Dimension		P1	P2	P3	P4	P5	P6	P7	P8	P9	P10	P11	P12	P13	P14	P15	P16	P17	P18
Policy Attributes	X1 Publishing agencies	0.33	0.67	0.33	0.33	0.67	0.33	0.67	0.33	0.33	0.67	0.33	0.67	1.00	0.33	1.00	0.33	1.00	0.33
	X2 Timeliness of policy	1.00	0.75	0.25	0.50	0.50	1.00	0.50	1.00	1.00	0.75	0.75	1.00	0.25	0.75	0.50	1.00	0.25	0.50
	X3 Policy fields	0.25	0.25	0.75	0.50	0.50	0.75	0.25	0.50	0.50	0.50	0.50	0.25	0.50	0.75	0.50	0.50	0.50	0.50
Policy Contents	X4 Policy objects	1.00	1.00	0.80	0.60	0.20	0.60	0.20	1.00	0.20	0.40	0.40	0.40	0.80	0.80	0.20	1.00	1.00	1.00
	X5 Policy orientation	0.17	0.50	0.50	0.50	0.33	0.50	0.50	0.50	0.33	0.67	0.17	0.17	0.33	0.33	0.50	0.50	0.33	0.67
	X6 Policy priorities	0.71	0.57	1.00	0.43	0.14	1.00	0.57	1.00	0.14	0.43	0.43	0.29	0.57	0.57	0.43	0.86	0.86	0.86
Policy Effects	X7 Policy tools	0.50	0.33	1.00	0.83	0.50	1.00	0.33	0.83	0.33	0.50	0.50	0.17	0.50	0.33	0.33	0.50	0.50	0.67
	X8 Incentive measures	0.17	0.50	0.50	0.33	0.33	0.50	0.17	0.67	0.33	0.33	0.33	0.17	0.33	0.33	0.33	0.33	0.67	0.50
	X9 Policy evaluation	0.50	0.50	0.75	0.75	0.50	1.00	0.50	1.00	0.75	0.75	0.50	0.75	0.75	0.75	0.50	0.50	0.75	1.00
PMC-index		**4.63**	**5.07**	**5.88**	**4.78**	**3.68**	**6.68**	**3.69**	**6.83**	**3.93**	**5.00**	**3.91**	**3.85**	**5.04**	**4.95**	**4.30**	**5.52**	**5.86**	**6.02**
Sag-index		4.37	3.93	3.12	4.22	5.32	2.32	5.31	2.17	5.07	4.00	5.09	5.15	3.96	4.05	4.70	3.48	3.14	2.98
Ranking		12	7	4	11	18	2	17	1	14	9	15	16	8	10	13	6	5	3
Evaluation grades		**A**	**A**	**A**	**A**	**B**	**G**	**B**	**G**	**B**	**A**	**B**	**B**	**A**	**A**	**A**	**A**	**A**	**G**

### PMC surface drawing

In this section, nine representative policies from 18 policies are divided into three groups. Their PMC surface map and radar map are constructed to analyze the specific situation of policy implementation from three different perspectives. In the PMC surface diagram, the vertical axis represents the three characteristics of policy attributes, policy content, and policy effects in the three-dimensional analysis framework, and the horizontal axis illustrates the three first-level indicators set under each framework, while the vertical axis represents the scores associated with each first-level indicator. In the figure, different scores are represented by specific color blocks. To facilitate a comparative analysis of the scores of the nine first-level indicators in the radar chart and to understand the relationship between the scores of policy attributes, policy contents, and policy effects, the radar chart presents scores for each of the nine indicators.

#### Analysis of content-oriented policies

**Fig 6:**
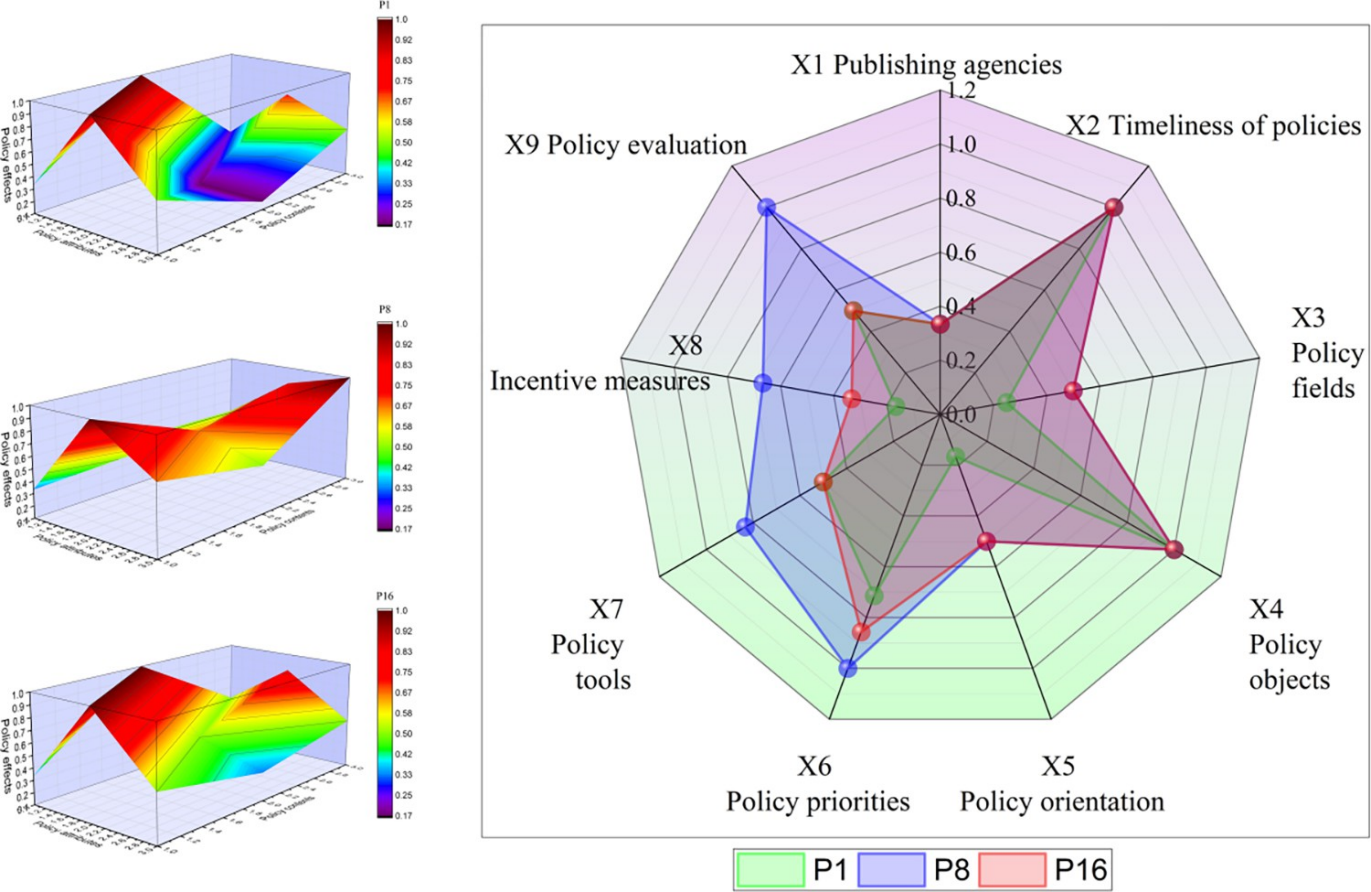
The PMC surface of content-based policy.

As shown in Fig 6, it is imperative to note that the content of the policy should not only reflect the main context but also reflect information about the policy orientation and the participants. In the three policies of P1, P8, and P16, the overall score for P8 is high, and the score of policy effect is the same as the score of policy content, which can be used as a benchmark for content-oriented supervision policies. As a whole, all three policies have relatively low scores for X1 publishing institutions, X3 policy areas, X5 policy orientation, and X8 incentive measures. As a result, policy P1 with the nature of notification has a lower level of issuing agency and less policy enforcement; policy P16 with the nature of opinion still requires improvement in policy implementation details, but enforcement is general. In terms of incentive measures, content-oriented policies tend to specify the scope and responsibility of medical subjects, suppliers, and demanders rather than how to encourage policy implementation. It is also evident from the low policy orientation score that multiple channels and types of means can be used to formulate policy content and ensure its implementation. Policy timeliness and policy objects for X2 and X4 are higher than those for the other two policies. This indicates that policies with a longer timeline and broader policy objectives are more likely to be implemented in the long run.

In comparison with policy P8, the scores for X6 policy focus, X7 policy tools, X8 incentive measures, and X9 policy evaluation of P1 and P16 still require improvement. The lack of policy focus suggests that the comprehensiveness of policy content needs to be improved. In addition, the overall effect of the policy needs to be improved, indicating that the policy pays more attention to the formulation and introduction of the front end. However, the absence of certain constraint mechanisms for effect feedback and assessment can easily result in poor policy implementation and difficult implementation of the results, making it difficult to achieve the purpose of policy formulation.

#### Analysis of effect-oriented policies

**Fig 7:**
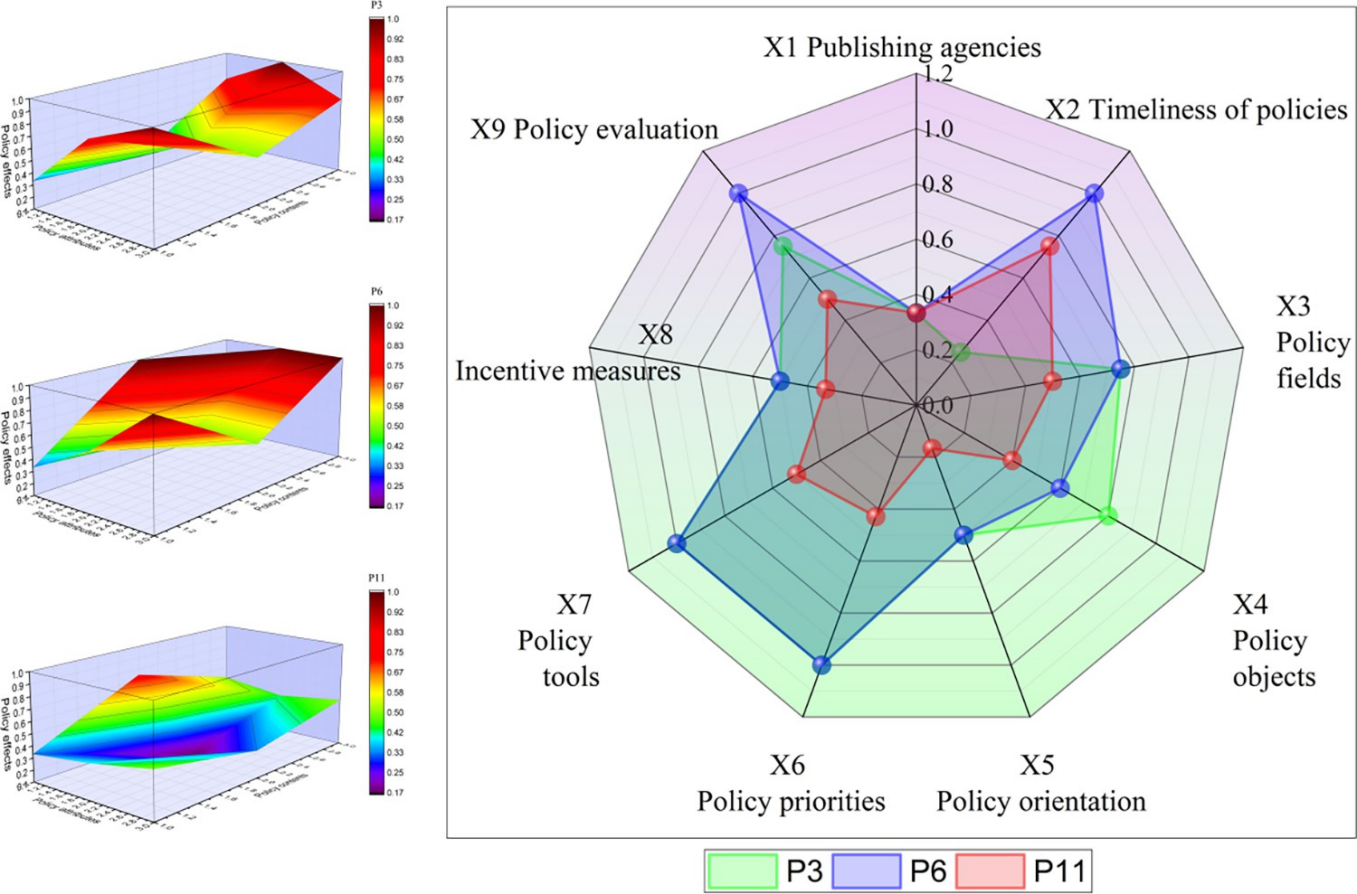
The PMC surface of the effect-oriented policy.

As well as reflecting the level of policy implementation, the level of policy effect can also reflect the incentive feedback mechanism. It can also reflect the use of policy tools after the policy was implemented. As shown in Fig 7, in policies P3, P6, and P11, the scores for policy attribute, policy content, and policy effect are all above 2, which indicates that it is a reasonably balanced and effective policy. When compared with P6, P3 has a big gap in X2 policy timeliness and X9 policy evaluation. It also has good performance in X7 policy tools and X8 incentive measures. There is a large gap between the levels and types of policies offered by the issuing agencies. This is the reason for the large gap. P3 of the National Healthcare Security Administration relates to the regulation of medical insurance funds. When compared to the guidance issued by the General Office of the State Council on reforming the supervision system for medical insurance funds, it is limited to a work guidance for 2019, which emphasizes more specifics and implementation, and its radiation range is also limited in terms of time. Using P6 as a guide for reforming the institutional system, the scope of action is larger, and it provides a comprehensive overview of the overall requirements, regulatory responsibilities, reform of the institutional system, and safeguard measures, as well as providing specific requirements for implementing the work.

In Fig 7, P11’s overall score and the subscores for policy attributes, policy content, and policy effects are slightly inferior compared with P3 and P6. These differences are more evident in X5 policy orientation, X6 policy focus, X7 policy tools, and X9 policy evaluation. There is a slight weakness in P11 due to the fact that it is an interim measure that supervises and manages medical funds. It concentrates on the handling measures and action framework of the report and pays less attention to the policy-oriented content and the implementation effects, as well as the limited impact of the release period. In addition, this illustrates that policies are often introduced with relevance and are aimed at a particular audience. There is no guarantee that a low score signifies a poor policy, but it can indicate the possibility that relevant policies could be improved under certain circumstances.

#### Analysis of implementation-oriented policies

**Fig 8:**
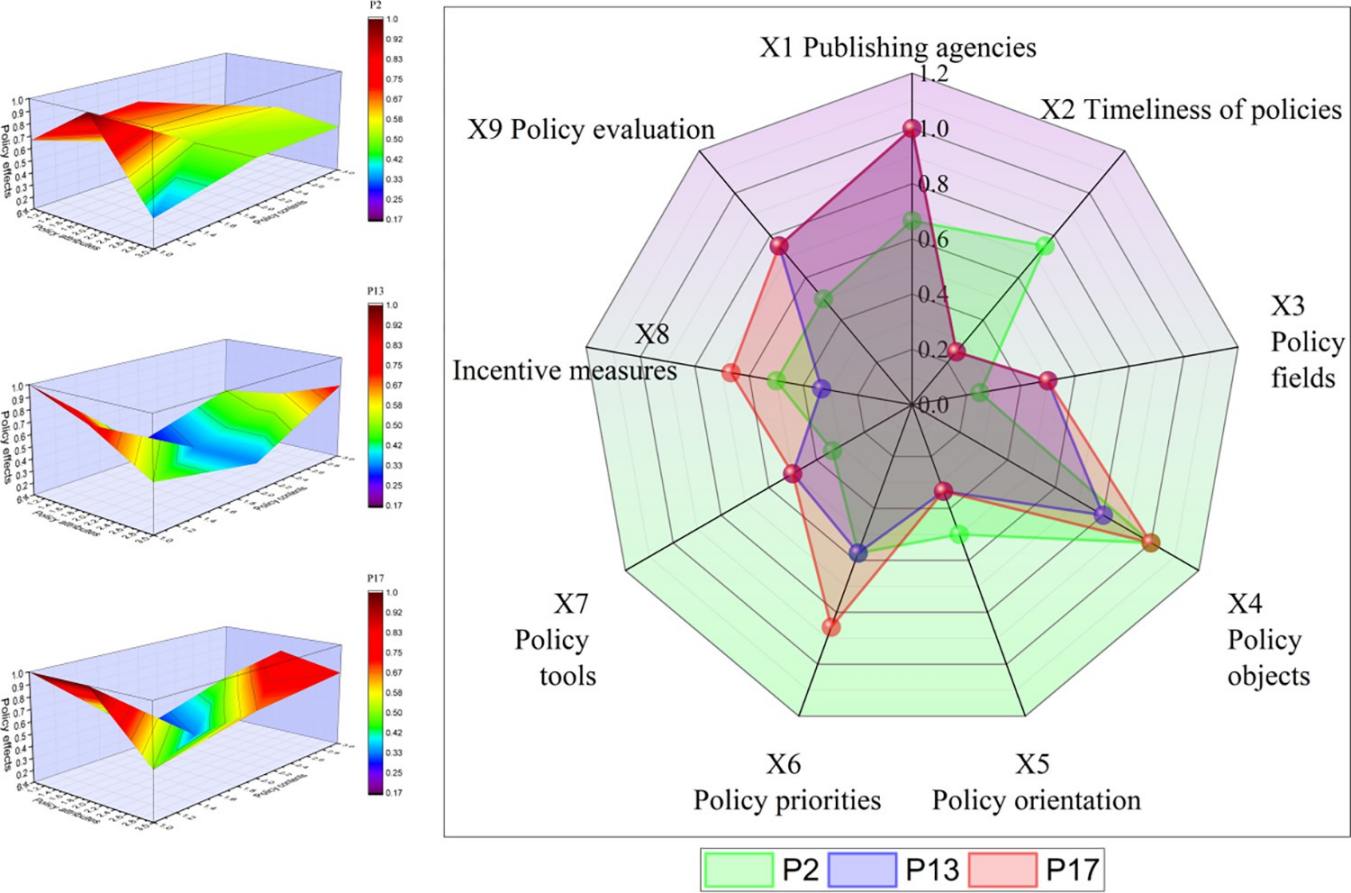
The PMC surface of implementation-oriented policies.

Insurance fraud can be combated through the improvement and standardization of systems and policies, as well as through effective and accurate inspections. As a result of analyzing 18 policies, the author concludes that the three P2, P13, and P17 are the most representative in terms of implementation in Fig 8. One of these policies, P2, provides incentives and methods for fraudulently defrauding medical insurance funds. Medical insurance funds will be subject to flight inspections in 2022 and 2023 in accordance with policies P13 and P17. In 2018, when the National Healthcare Security Administration had not yet begun its flight inspection work, P2’s policy formulation included all X4 policy groups. Although the overall score of P2’s three dimensions is at the middle level, the reporting reward policy is the only effective strategy for resisting insurance fraud. It also strengthens fund supervision. As well, it provides experience and foreshadowing for the formulation and implementation of interim measures for medical insurance fund supervision and management. It also provides special provisions for resolving insurance fraud on the part of plan providers.

The comprehensive advancement of flight inspection has curbed blatant fraud behavior, but more covert means and professional fraud technology have made fund supervision harder. Therefore, the workflow, problem handling, and implementation plan of flight inspection also need to be continuously improved. Comparing P13 and P17, it can be found that the scores of flight inspection in X4 policy object, X6 policy focus, and X8 incentive measures have been significantly improved, which shows two problems. One problem is to cover as many medical behavior participants and all aspects of fraud as possible from the perspective of policy formulation. Another problem is to pay more attention to the use of incentive measures from the feedback mechanism of the policy, give full play to the role of administrative personnel, institutional framework, incentive measures, internal self-examination, and other ways, fully demonstrate China’s determination and action force to improve the supervision mechanism, and carry out flight inspection work in a legal, standardized, and scientific way.

### Analysis of findings from policy evaluations

In terms of policy type and policy hierarchy, among the 18 policies supervised by the medical insurance fund, 13 are ’Notice’ policies, 2 are ’Guidelines’ policies, 2 are ’Interim measures’ policies, and 1 is ’Regulations’ policies. Among them, only the ’Regulations’ formulated by the State Council involved in this article belong to administrative regulations. The ’Interim Measures’ are in accordance with departmental regulations. These three policies belong to laws and regulations. At the same time, ’Notification’ and ’Guidelines’ are also policy documents with legal effect. The policy level, however, could be improved significantly. This will provide a more effective deterrent for the supervision of medical insurance funds and the prevention of fund fraud if the relevant legal documents are issued.

Among the 18 policies, the average score of X8 incentive measures is the lowest among the secondary indicators. In addition to the above analysis, this shows that there are relatively few incentive measures adopted during the policy formulation process, and the implementation effect can be viewed from an incentive perspective. In addition to the low scores for both X1 publishing institutions and X3 policy areas, there is some convergence among the weak links. For medical insurance fund supervision policies to be more rational and scientific, long-term and short-term goals should be taken into consideration comprehensively, as well as X9 policy evaluation.

In the connection between policies, such as P8, P9, and P16, there is a clear guiding relationship, to a certain extent. This is to ensure the continuity and supplementary cooperation of the policy. The scores of the three dimensions of policy attribute, policy content, and policy effect are relatively balanced. Most of the policies have advantages in the scores of X4 policy objects and X7 policy tools. However, they can still be strengthened, indicating that policy receptor scope can still be extended. It is possible to extend the scope of the application of policy tools in a more diverse way in order to enhance their implementation effectiveness.

## Conclusions and suggestions on preventing medical insurance fraud

### Conclusions

This paper utilizes the conceptual analysis framework of "Antecedents, Processes, and Outcomes" to analyze 180 cases of fraud and insurance fraud since the establishment of the National Healthcare Security Administration. In the study, there were concerns about the level of settlement, supervision intensity, and authority use. Thus, the 18 key medical insurance fraud supervision policies for the same period are further evaluated quantitatively. As a result of previous studies, high-frequency keywords in texts and high-frequency supervision policies are extracted through policy document mining. Using this information, a text evaluation model of a medical insurance fraud supervision policy is constructed using nine first-level variables, 47 second-level variables, and corresponding evaluation criteria. On the basis of the input-output table, the PMC index of 18 policies is determined, and the following conclusions are drawn after sorting the depression index:

Firstly, China’s medical insurance fraud has complexity, concealment, and multifaceted involvement. Medical insurance fraud can be classified according to its types and manifestations. In summary, the problem of medical insurance fraud is mainly concentrated in three aspects: policy attributes (inadequate legal supervision level, absence of social supervision in the policy field), policy contents (strengthening supervision, inspection, punishment, and audit monitoring for tripartite stakeholders), and policy effects (strengthening incentives and reducing fraud motivation). The ’frequency’ and ’harmfulness’ of medical insurance fraud not only increases healthcare costs and reduces the available funds for legal medical services, but also damages the long-term solvency of medical insurance funds, affecting the balance of payments of medical insurance funds as well as the reform of the payment system based on total amount control.

Secondly, through the overall analysis of the PMC index, it is found that the overall quality of the regulatory policies for the use of medical insurance funds in China is not high, which is in line with the ’spindle’ conclusion of policy evaluation. In total, 10 policies (55.6%) are rated as middle-level (acceptable), three policies are considered good, and the remaining five are rated low, indicating that the policies are comprehensive.

Thirdly, China’s medical insurance fraud supervision policy has a certain degree of convergence with the actual state of policy-level variables. In terms of policy timeliness, policy evaluation, and policy orientation, a high-quality rating policy clearly has advantages. It is, however, relatively weak in terms of policy institutions and policy fields, particularly in terms of policy orientation and policy tools.

Fourth, the regulatory focus of different types of policies differs. There is heterogeneity among various dimensions of policies as a result of the actual situation of the secondary variables of the policy. As a rule, an effective policy is comprehensive and in-depth, while a poor policy focuses on specific secondary indicators. There is a commonality among policies with "low" ratings (P5, P7, P9, P11, P12) in that X4 (policy object) and X5 (policy orientation) indexes tend to be low, while X7 (policy tool) and X8 (incentive measures) indexes tend to be high. It has been found that the X1 (publishing agency) and X2 (policy timeliness) indexes have improved after the supplementary explanation relationship has been considered for the same category of policies.

Lastly, on the whole, there is still room for improvement with regard to the overall effect of medical insurance fraud supervision. Specifically, it should be conducted at four levels: enriching the policy field X4 (political object), improving the policy supervision chain X5, strengthening the policy orientation X5, and seeking innovative policy tools X7 (policy tools).

### Suggestions

#### Enriching policy areas and intelligent technology enabling audit monitoring

Technically, the policy field has a low rating. The phrase "sound intelligent monitoring mechanism" appears in the latest regulatory policy, indicating that intelligent technology is receiving greater attention [40]. Medical insurance fraud, complexity, and concealment are prominent in today’s environment. “Big Data” and "Internet Plus" should be fully utilized in the field of medical insurance supervision. As a first step, to improve the effectiveness of intelligent monitoring, the rules, indicators, and knowledge base of monitoring should be revised and enriched. Second, it is necessary to improve accuracy in the field of "Internet + video surveillance" from the point of collection of evidence through the judgment process. Furthermore, in the case of face recognition technology, we should pay particular attention to the front-end control, that is, the correlation between face discrimination and illegal data collection. Furthermore, an efficient auditing and feedback system is also required in healthcare organizations’ clinical diagnosis and treatment behavior. This is particularly relevant to medical insurance supervision. By transforming post-audit and passive supervision into pre-intelligent early warning and active supervision, medical insurance operators will be able to improve their ability to identify risk sources and suppress risk trans-mission, thus curbing the excessive use of medical insurance funds.

#### Improving the policy chain and considering the impact of medical insurance payment reform on different policy objects

As a result of the reform of payment methods, the regulatory chain is extended, resulting in more extensive coverage of policy targets for fraudulent insurance and frequent points of exposure to risk. The medical insurance industry should examine incentives and restraint mechanisms that are appropriate to China’s national conditions [41]. Presently, the total amount of medical insurance is controlled by revenue and expenditure, but the cost control effect of this method is not prominent, and there has been an incentive distortion effect. As a result, it is necessary to emphasize the importance of "balance retention" and to further encourage medical institutions to take measures such as "cost control incentives." The total amount control method is difficult to apply to patients with specific characteristics. It is possible to limit medical institutions’ annual budgets more accurately by using various methods of medical insurance payment, such as capitation, DRG, and DIP. As a result, medical institutions’ heterogeneity should be fully considered when designing top-level payment methods for medical insurance, and medical institutions at different levels should adopt different reform strategies for medical insurance payment methods, depending on budget constraints and market bargaining power.

#### Strengthening policy guidance and investigating the responsibility of fraud and insurance fraud

As a result of the characteristics of policy-oriented influence, it can be regarded as being both guiding and far-reaching, and the two angles of positive and negative pol-icy influence can be combined into a joint force. From the perspective of positive incentives, it is possible to construct the DRG audit system embedded within the quality management assessment indicators of medical institutions using real-time monitoring systems that take into account the quality and quantity of services, the structural optimization of services, and possible side effects, as well as ensuring that positive incentives are formed through error correction. At the level of negative punishment, it is necessary to improve the laws and regulations governing medical insurance fund supervision at the legislative level. It is also necessary to establish a unified regulatory authority and punishment standard. During the development of the credit system, it is recommended that the medical security credit system be integrated with the dishonesty punishment system and the trustworthy joint incentive system. It is important to integrate the law enforcement efforts of the various departments and to consider agreement, judicial, and administrative methods within the same framework in order to ensure effective law enforcement. For units and individuals who have committed fraudulent insurance fraud, criminal responsibility for suspected criminal acts should be investigated according to the severity of the circumstances. By cracking down on fraudulent insurance fraud, insured people’s legitimate rights and interests can be protected.

#### Learning from advantages of other countries’ policies and coordinating relationship between three parties of medical insurance

Compared to other countries in the world, China has a higher reporting frequency and reward intensity, as well as the advantage of integrating public security, prosecution, and law in handling cases at the post-event control level. However, there are still deficiencies, mainly because the insured is a relatively weak party. In the future, we should pay attention to protecting and improving the rights and interests of the insured in policy formulation so as to reduce the internal motivation of the insured to participate in fraud or to conspire with medical institutions to carry out medical insurance fraud. At the same time, big data technology is used to monitor and supervise medical institutions so as to better reduce the probability of medical insurance fraud.

#### Seeking innovation policy and providing policy knowledge base

In terms of formulation, selection, and distribution of key elements, the ’Antecedents-Processes-Outcomes’ analytical framework illustrates that fraud regulation policy is a diversified, complex, and dynamic system [42–44]. This process involves a variety of stakeholders and has many components. The macro-orientation and regulatory effectiveness of the policy, as well as the impact of the decision-making environment, should be considered. The combination and weight changes of different variables cannot simply be eliminated; that is, how the command type, normative type, incentive type, capacity building type, system change type, and persuasion type in this paper’s evaluation index system form the medical insurance fraud supervision policy under a variety of fraud response variations through different combinations and weighting changes. Providing an intellectual basis for the construction of a modern national governance system based on Chinese principles will also contribute to the solution of this problem.

## Supporting information

S1 Appendix 180 medical insurance fund fraud cases. (DOCX)

S2 Appendix 18 original policy documents. (DOCX)
