# Supplementary material for: Research on quantitative evaluation of medical insurance fraud supervision policy based on ‘Antecedents-Process-Outcomes’ framework
Source: PLoS One. 2025 Jan 6;20(1):e0313618. doi: 10.1371/journal.pone.0313618 (PMC11703012; doi:10.1371/journal.pone.0313618)
Supplement: S1 Appendix — (DOCX) [file pone.0313618.s001.docx]

Medical insurance fund fraud cases

(a total of 180 cases)

A total of 24 typical cases (2019-01-25——2019-05-15)

First 8 cases in 2019

1. Inner Mongolia Autonomous Region Hulunbeier City Tooth Rock Shi City Tuli River Town Center Hospital to cheat the medical insurance fund case by the false hospitalization

After investigation, there were 207 cases of suspected violation of medical records in the Central Hospital of Inner Mongolia Autonomous Region, including over-examination, over-diagnosis and over-treatment. From June 2016 to October 2017, there were seven false hospitalizations involving 22,000 yuan from the medical insurance fund. In accordance with Article 87 of the Social Insurance Law of the People's Republic of China, Article 54 of the regulations of the Inner Mongolia Autonomous Region on urban basic medical insurance, and the service agreement of designated medical institutions (Level I comprehensive) for urban (rural) basic medical insurance in Hulunbuir City, they recovered 22,000 yuan from the medical insurance fund and were fined 110,000 yuan five times over. Health Care Department to the head of the hospital to stop working decisions, and related violations of discipline staff to deal with.

2. The second Maozhi Hospital of Huainan City, Anhui Province cheated the medical insurance fund of Yingshang County of Fuyang city by false hospitalization

After investigation, Huainan Maoji second hospital to free medical examination as the reason, access to Fuyang City Yingshang County to participate in the mass information, fabricated in-patient treatment materials, to obtain the new rural cooperative fund. From August 2016 to August 2018, the hospital fabricated 450 false hospital admissions information to defraud the new rural cooperative fund 1.36 million yuan. The medical insurance authorities, in accordance with the provisions of articles 17 and 38 of the interim measures for the supervision and administration of the basic medical insurance of Anhui province and articles 14 and 15 of the measures for the investigation and punishment of illegal acts by designated medical institutions under the new rural cooperative medical system, terminate and Huainan City Mao Ji Second Hospital Medical Insurance Service Agreement, and the relevant clues handed over to the public security organs for investigation. With the approval of the Yingshang County People's Procuratorate, the public security organs have taken 6 people into criminal detention, released on bail pending trial 4 people, and pursued 2 people online. In accordance with Article 79 of the Implementing Rules of the regulations on the management of medical institutions, the health and planning department revoked the hospital's medical institution practice license.

3. Pingxiang city, Jiangxi province, Anyuan District, the People's Pharmacy Drug Exchange Fraud Medical Insurance Fund case

After investigation, from January 2017 to October 2018, the sales data in the medical insurance system of the People's big pharmacy in Anyuan District, Pingxiang City, Jiangxi province were larger than the sales data in its stores, and there were problems such as replacing and exchanging medicines, the amount involved was 49,000 yuan. The medical insurance authorities recovered 49,000 yuan for illegal drug sales and suspended the medical insurance service agreement for three months, according to Article 33 of the agreement.

1. Wuzhou Hospital of Qichun County, Huanggang City, Hubei province

Wuzhou Hospital in Qichun County, Huanggang City, Hubei Province, defrauded 41,000 Yuan from the insurance fund in August 2018 by lying in a bed, inducing hospitalization on the grounds of physical examination, and using drugs without doctor's orders. In accordance with the provisions of articles 50,51,52,53, and 54 of the service agreement for medical institutions of Huanggang basic medical insurance, interview the person in charge of the hospital and order him to stop the violation immediately. From November 16,2018, the hospital suspended the fixed-point eligibility for medical insurance, according to the amount of five times the hospital's medical insurance settlement deducted 205,000 yuan, and handed over to the health department for further investigation and punishment.

5. The case of the fictitious five-guarantee household defrauding the medical insurance fund by staying in the hospital of Qijiang Hospital of Chaling County, Hunan province

After investigation, Lei Mou, the director of Qijiang Health Center, instructed the Doctor to contact the Qijiang nursing home for the elderly, under the guise of a medical examination for the five-guarantee households of the Qijiang nursing home, to collect the register of the five-guarantee households of the nursing home for the elderly, in February 2018, Liu, Chen and 28 other five-guarantee households were admitted to hospital illegally by means of suspended beds and fictitious expenses, and arranged the hospital staff to make false records of doctor-patient exchanges, medical records, diagnosis, prescriptions and other relevant information, illegal collection of health insurance funds 36,000 yuan. According to Article 20 of the measures for the administration and supervision of designated medical institutions under the basic medical insurance of Chaling County (for trial implementation) and Article 47 of the service agreement of designated medical institutions under the basic medical insurance of Chaling County, it shut down the hospital's medical insurance reimbursement system, suspended medical insurance services, ordered a comprehensive overhaul, recovered 36,000 yuan from the medical insurance fund, and refused to pay five times the illegal fees to the medical insurance fund, the health care department was also advised to deal with the hospital's medical staff. Discipline Inspection and supervision departments to Ray a serious warning within the party punishment, and give the hospital 6 staff exhorted to talk to deal with.

6. The case of medical insurance fund fraud by the clinic of Pudu Village, Kaizhou District, Chongqing City

According to the investigation, the person in charge of the clinic and the Smart Medicine supermarket in Pudu Village, Kaizhou District, Chongqing City, was both surnamed Li. The village clinic was in charge from February 2018 to November 2018, through the smart drug supermarket drug expenses reimbursement card, drug exchange, and other ways to defraud the health insurance fund 200.9 million yuan. In accordance with Article 25 of the Medical Service Agreement of medical institutions of Chongqing Medical Insurance Agreement and Article 19 of the Medical Service Agreement of clinics (community health service stations) of the medical insurance village of Kaizhou District of Chongqing, suspended the settlement of his medical insurance network for three months, suspended the qualification of a medical insurance physician named Li for six months, recovered 200,900 yuan of illegal expenses, and was sentenced to double the penalty for breach of contract, the village clinic refused to pay 13,500 yuan in 2018 for general consultation and treatment, and ordered the smart drug supermarket to rectify the situation by a deadline.

7. The case of inducing patients to be hospitalized to defraud the medical insurance fund in Sichuan Dazhou Renai Hospital

According to the investigation, from January 2018 to June 2018, the Renai Hospital of Dazhou city in Sichuan province induced patients to be hospitalized by waiving patients' self-payment, transportation by vehicle, and giving out quilts and medicines, the hospital defrauded 90,300 Yuan from the medical insurance fund by overwriting and falsely recording expenses. According to Article 88 of the Social Insurance Law of the People's Republic of China and articles 56 and 57 of the service agreement on designated medical institutions of the Tongchuan district basic medical insurance, recovery of illegal costs of 90,300 yuan, and 5 times the fine of 451,500 yuan, cancel the hospital medical insurance designated medical institutions. In accordance with Article 48 of the Regulations on the administration of medical institutions, the health and planning department revoked the hospital's medical institution practice license and imposed a fine of 342,000 yuan. Two people were arrested and one released on bail pending trial. The Department of Discipline Inspection and Supervision shall give disciplinary treatment to the relevant staff members of the social welfare (medical insurance) and health planning departments.

8. Tianshui City, Gansu Province, Qin 'an integrated traditional and western medicine hospital to induce patients in hospital to defraud medical insurance fund case

According to investigation, from January 2018 to October 2018, Qin 'an integrated traditional and western medicine hospital in Tianshui City, Gansu province, the Insurance Fund was defrauded of 136,200 yuan by over-treatment, hospitalization of insured individuals who could be treated in outpatient department, and hospitalization of insured persons induced by free medical examination. In accordance with articles 79 and 82 of the Social Insurance Law of the People's Republic of China, articles 8,12,13 and 14 of the measures for deducting the default funds of designated medical institutions of the basic medical insurance for urban and rural residents of Gansu Province (annex) , and articles 50,51 and 52 of the medical service agreement of designated medical institutions of Tianshui medical insurance, starting from December 11,2018, the hospital's medical insurance service agreement was rescinded, the hospital's medical insurance designated medical institutions were disqualified, and illegal fees of 136,200 yuan were recovered, and a fine of 681,000 yuan was imposed five times, recovery audit found that overcharged bed fees, decomposition of hospitalization, expanding the scope of illegal reimbursement claims fund 29.5 million yuan.

The second batch of 8 cases in 2019

1. Fuyang Shuanglong Hospital in Fuyang City, Anhui province

After a flight check by the state medical insurance bureau, shuanglong hospital in Fuyang city, Anhui Province cheated the medical insurance fund by buying patients from village doctors, over-treatment, over-examination, out-of-scope practice, and non-health technical personnel to independently carry out diagnosis and treatment activities in the form of paying kickbacks. From 2016 to November 2018, the hospital carried out over-the-range operations to obtain 382,000 yuan from the medical insurance fund, and 181,500 yuan for over-treatment and over-examination. In accordance with Article 34 of the interim measures for the supervision and administration of basic medical insurance in Anhui province, the medical insurance authorities recovered 563,500 yuan from the medical insurance fund and imposed a fine of 907,500 yuan and rescinded the medical insurance service agreement, they handed over relevant clues to the public security organs for further investigation. The Health Department fined the hospital 4,000 yuan in accordance with Article 48 of the regulations on the management of medical institutions and article 81 of the Implementing Rules of the regulations on the management of medical institutions, and revoke its license to practice medicine.

1. The medical insurance fund fraud case of Heyang Gospel Hospital in Weinan city, Shaanxi province

Heyang Gospel Hospital, Weinan city, Shaanxi Province, from September 2017 to 2018, medical insurance funds were defrauded of 388,600 yuan by means of non-prescribed fees, over-prescribed fees and over-examination. According to Article 87 of Chapter 11 of the Social Insurance Law of the People's Republic of China, and Articles 44 and 45 of the service agreement of designated medical institutions for the basic medical insurance in Weinan cities and towns, article 25 of Chapter 5 of the service agreement for designated medical institutions in Heyang County stipulates that 388,600 yuan will be recovered from the medical insurance fund and a fine of 849,500 yuan will be imposed, at the same time, the hospital also rescinded the medical insurance for urban residents and the New Rural Cooperative Medical Service Agreement.

1. Ningxia Hui Autonomous Region Yinchuan Yinchuan Baihe Hall hospital fraud medical insurance fund case

Ningxia Hui Autonomous Region Yinchuan Yinchuan Lily Hall Hospital in 2017 to 2018, the medical insurance fund was defrauded of 180,100 yuan by means of hospitalization, false examination and false charge. In accordance with the first and second paragraphs of Article 21 of the measures for the supervision of the basic medical insurance services of the Ningxia Hui Autonomous Region, articles 23,24, and 25 of the measures of Ningxia Hui Autonomous Region on the administration of the integrity of medical insurance service physicians stipulate that 180,100 yuan of medical insurance funds will be recovered and a fine of 540,300 yuan will be imposed, at the same time, the medical insurance service agreement shall be rescinded.

1. The medical insurance fund fraud case of the Shiguzhuang community health service station of Songzhuang Town, Tongzhou District, Beijing City

After investigation, Songzhuang Town, Tongzhou District, Beijing Shiguzhuang Community Health Service Station through the purchase of false purchase invoices, Yin-yang prescriptions and other ways to defraud health insurance funds. From January to August 2018, the head of the service station from the online purchase of Chinese herbal pieces purchase invoices 8, defrauded Health Insurance Fund 602.3 million yuan. The medical insurance department rescinded the medical insurance service agreement according to the articles 2,52,74 and 91 of the service agreement of the designated medical institutions of Beijing basic medical insurance. The person in charge of the service station is under criminal detention by the Tongzhou branch of the Beijing Municipal Public Security Bureau. The case is still under further investigation and the amount of the illegal amount is to be recovered immediately after verification.

1. The case of the Friendship Hospital of Yaodu District of Linfen City of Shanxi province defrauding the medical insurance fund

According to investigation, from January to December 2018, Yaodu Friendship Hospital in Linfen City, Shanxi province defrauded 657,100 Yuan from the medical insurance fund by charging without medical advice, falsely recording fees for examinations, and charging more for physical therapy items. According to Article 87 of Chapter 11 of the Social Insurance Law of the People's Republic of China and Article 61 of the service agreement of designated medical institutions under Shanxi medical insurance, recovery of the Medical Insurance Fund 657.1 million yuan, 1971.3 million yuan penalty for breach of contract, termination of the medical insurance service agreement, and related clues to the public security organs for further investigation.

1. Shanghai Baimaoling Hospital defraud medical insurance fund case

According to the investigation, from 2013 to 2017, Shanghai Baimaoling Hospital defrauded 94,700 Yuan from the medical insurance fund through false records, tests and overcharging. In accordance with article 17 of the Shanghai Municipal Measures for the supervision and administration of basic medical insurance, the medical insurance department ordered the hospital to rectify the situation, recover 94,700 yuan from the medical insurance fund and impose an administrative fine of 100,000 yuan, the medical insurance settlement of 3 responsible doctors was suspended for 6 months and the clues were transferred to the Health Department for further investigation.

1. The case of changing the medical insurance code to cheat the medical insurance fund in an Bian Health Center of Ma Xiang Health Yuan, Xiang 'an District, Xiamen City, Fujian Province

After investigation, Ma Xiang Health Yuan an Bian Health Center in Xiang 'an District, Xiamen City, Fujian Province, from July 2017 to June 2018, by changing the medical insurance code, over the scope of medical insurance payment for medical treatment and other ways to defraud the medical insurance fund 961,100 yuan. The medical insurance department shall, in accordance with articles 55 and 56 of the 2017 agreement on the medical services of designated medical institutions under the basic medical insurance of Xiamen Municipality, article 4 of the operational rules for the credit scoring of medical insurance personnel in designated service units of Xiamen basic medical insurance stipulates that 961,100 yuan of medical insurance funds will not be paid; the health insurance network of the Anbian Health Institute will be suspended for six months; A responsible physician will receive 12 credit points and 12 months of non-payment for medical insurance services.

1. Case of Children's Hospital defrauding medical insurance fund in Qiandongnan Hongzhou, Guizhou province

The investigation found that Qiandongnan Hongzhou Children's Hospital in Guizhou province defrauded 335,700 yuan from the medical insurance fund between 2017 and 2018 through such means as admission without beds, hospitalization by hanging beds, and multiple record diagnosis and treatment programs. The medical insurance department recovers 335,700 Yuan from the medical insurance fund in accordance with the provisions of articles 43,44 and 49 of the 2018 Basic Medical Insurance Service Agreement for designated medical institutions (trial implementation) of Kaili City, it also fined 170,600 yuan, refused to pay the 2018 year-end liquidation deposit, rescinded the health care service agreement, and issued a statewide alert.

The third batch of 8 cases in 2019

1. Medical Insurance Fund fraud case at Jilin Anzhen Hospital, Changchun City, Jilin province

After a flight check by the State Medical Insurance Bureau, Anzhen Hospital in Changchun, Jilin province, from 2017 to 2018, through the collection of medical insurance card for false hospitalization, impersonation in hospital, hospital bed, false declaration of medical insurance costs and other ways to defraud the health insurance fund 1688.7 million yuan. In accordance with Article 87 of the Social Insurance Law of the People's Republic of China, Article 23, paragraph 1, of the measures for the administration of designated medical institutions for the basic medical insurance in urban areas of Changchun Municipality, and Article 62 of the Agreement on the medical services of designated medical institutions for the basic medical insurance of Changchun Municipality of 2018, recovery of the Health Insurance Fund 1688.7 million yuan, the suspension of health insurance costs 3.42 million yuan, and terminate the health insurance service agreement.

2. A case of defrauding medical insurance funds by Yanshan Hospital of Qinglong Manchu Autonomous County, Qinhuangdao City, Hebei Province

An investigation found that Yanshan Hospital in Qinglong Manchu Autonomous County, Qinhuangdao City, Hebei Province, defrauded medical insurance funds of 86,800 yuan from May 2017 to December 2018 by falsifying examination reports and swapping test items. In accordance with Article 25 of the measures of Hebei province on the administration by agreement of designated medical institutions for basic medical insurance, articles 50,59 and 60 of the agreement on medical services for designated medical institutions in Qinhuangdao City (2018) provide that the medical insurance fund shall be recovered to 86,800 yuan and the penalty for breach of contract shall be 197,900 yuan, the Medical Insurance Service Agreement shall be rescinded, and relevant clues shall be handed over to the public security organs for investigation. Qinglong Manchu Autonomous County Public Security Bureau has a criminal detention, released on bail pending trial 1 person, residential surveillance 2 people, related clues for further investigation.

3. The case of the joint orthopaedic doctor defrauding the medical insurance fund in Kunshan Hospital of Traditional Chinese Medicine, Jiangsu province

According to the investigation, Sun Mou, a doctor in the Department of Orthopaedics and joints of Kunshan Hospital of traditional Chinese medicine in Jiangsu province, helped others to cheat 25,400 yuan from the medical insurance fund by using the social insurance card of the insured from June 15 to 24,2018. In accordance with Articles 6 and 8 of the measures for the examination and assessment of designated medical institutions for the basic medical insurance of Kunshan, and article 57 of the agreement on the medical services of designated medical institutions for the basic medical insurance of Kunshan, “Measures of Kunshan Municipality on the administration of doctors' medical insurance prescription right in designated medical institutions (for trial implementation)”, Articles 10 and 11, articles 4 and 5 of the“Measures for handling the violation of social medical insurance regulations by the insured persons in Kunshan City (for trial implementation)” stipulate that 25,400 yuan of the medical insurance fund will be recovered, and at the same time, the hospital's competent leaders and the director of the medical insurance office will be interviewed Canceling the medical insurance prescription qualification of Sun Mou, turning over the department director Xu Mou and the bed doctor Liu Mou to the health department for processing; including the personnel who lent the social insurance card into the medical insurance blacklist; Sun and four other people were transferred to the public security organs for further verification.

4. Lankao County, Henan province, Lanyang Hospital fraud medical insurance fund case

After investigation, Lankao County, Henan province, Lanyang Hospital through exchange of medical treatment projects, over-treatment and other ways to defraud the health insurance fund 2,668,300 yuan. According to Articles 87 and 88 of the Social Insurance Law of the People's Republic of China, the medical insurance authorities recovered 2,668,300 yuan from the medical insurance fund and fined 5,336,600 yuan, to cancel the subject of a medical insurance xiao medical qualifications, lifting the hospital department involved in fixed-point medical insurance qualifications. The health department dismissed the president of the hospital and criticized the hospital throughout the county.

1. The case of medical insurance fund fraud by Huazhouji Hospital of Huazhouji City of Guangdong province

The investigation found that from October 2016 to 2018, the hospital defrauded 118,600 Yuan from its medical insurance fund by making multiple records of medical treatment and examinations. According to Article 87 of Chapter 11 of the Social Insurance Law of the People's Republic of China, and articles 60 and 61 of the regulations of Guangdong province on the supervision of social insurance funds, article 60 of the Medical Service Agreement of the designated medical institutions of the social insurance in Maoming city stipulates that the medical insurance fund shall be recovered to 118,600 yuan, a fine of 593,100 yuan, and the Medical Service Agreement shall be terminated. The health authorities ordered them to make rectification within a time limit.

1. Case of defrauding medical insurance fund of golden field hospital in Guiping City, Guangxi Zhuang Autonomous Region

According to the investigation, the hospital in Guangxi Zhuang Autonomous Region defrauded 83,800 yuan in February 2017 by making up medical services and breaking down fees. In accordance with Article 65 of the 2018 Agreement on the medical services of designated medical institutions under the basic medical insurance of your city, the medical insurance department recovered 83,800 yuan from the medical insurance fund and ordered it to immediately rectify the situation, at the same time, the joint health department to the President of the hospital for removal, and together with the Commission for Discipline Inspection of the hospital for violations of the law and regulations of the personnel responsible.

7. Medical insurance fund fraud case in Huaxin Hospital of Zhaotong City, Yunnan Province

After investigation, Huaxin Hospital in Zhaotong City, Yunnan Province, from January 2017 to April 2018, through forged drug purchase list, false hospitalization, false declaration of medical insurance costs and other ways to defraud the health insurance fund 108.4 million yuan. According to Articles 67 and 68 of the service agreement for designated medical institutions in the 2018 medical insurance agreement of Zhaotong Municipality, the medical insurance authorities suspended the allocation of medical insurance funds, and transfer the relevant clues to the public security organs for investigation. The Zhaoyang District Public Security Bureau has arrested 5 people and released 1 person on bail pending trial. The case is still under investigation and the amount of the illegal amount will be recovered after further verification by the public security department.

8. Medical insurance fund fraud case in Wenshu County, Aksu Prefecture, Xinjiang Uygur Autonomous Region

According to investigations, the hospital in Aksu Prefecture, Xinjiang, defrauded 21,800 yuan from the insurance fund between May 2017 and December 2018 by falsifying medical records and lowering hospitalization indications. In accordance with Article 62, paragraph 18, of the Agreement on the services of designated medical institutions under the basic medical insurance of Aksu Prefecture, and Article 16 of chapter 8 of the measures for the administration of the access to and withdrawal from designated medical institutions and retail pharmacies under the agreement on the administration of the access to and withdrawal from designated medical institutions under the basic medical insurance of Aksu Prefecture, recovers the medical insurance fund 21,800 yuan, the fine 65,400 yuan, terminates the medical insurance fixed-point agreement, and cancels the medical insurance fixed-point qualification.

A total of 116 typical cases were exposed on the exposure station of the state medical insurance bureau (2020-07-10-2022-11-11)

First 5 cases in 2020

1. Medical insurance fraud case at Yuncheng Benevolent Hospital in Shanxi province

According to the investigation by Yuncheng Salt Lake District Medical Security Bureau, Yuncheng Benevolent Hospital defrauded 48,600 yuan from the medical insurance fund in 2018 by lying in bed and falsely recording medical service fees. In accordance with Article 87 of the Social Insurance Law of the People's Republic of China, Article 68, Paragraph 7, Article 68, paragraph 1, of Chapter 8, Service Agreement on designated medical institutions in 2019, it refused to pay 48,600 yuan to the hospital's medical insurance fund, deducted 145,700 yuan from the tripling penalty, rescinded the medical insurance service agreement of Yuncheng Yan Oi Hospital, and transferred the case to the judicial authorities.

2. The medical insurance fund fraud case of Huiqi Manchu Township Health Center of Wangkui County, Suihua City, Heilongjiang Province

After an investigation by the Suihua Municipal Medical Security Bureau, the health center of Huiqi Manchu town in Wangkui County fraudulently obtained 206,000 yuan from the medical insurance fund in 2018 by falsifying medical record examination reports, at the same time, 17 medical records were examined by medical experts as no basis for diagnosis, medical expenses were written off medical insurance fund 35,300 yuan. In accordance with Article 87 of the Social Insurance Law of the People's Republic of China, Article 22 of the Law of the People's Republic of China on administrative punishment, and Article 3 of the provisions on the transfer of criminal cases by administrative law enforcement agencies -Orderrder of StatetCouncilnNo. no. 310) , he was ordered to return 241,300 yuan to the medical insurance fund and was fined 482,600 yuan twice that amount. He also reported the case to the health authorities and sent it to the public security organs for processing.

1. Quzhou Jiuan Cardiovascular Hospital Co. , Ltd. defrauded Medical Insurance Fund case

After investigation by Quzhou Medical Security Bureau and Quzhou Medical Insurance Center, from 2017 to 2019, Quzhou Jiuan Cardiovascular Hospital (Co. , Ltd.) defrauded the health insurance fund of 1,424,400 yuan by faking medicines and medical treatment programs, forging medical documents, etc. , the medical insurance fund was reimbursed 690,800 yuan due to non-compliance in the sale and storage of drugs and irregularities in the management of physiotherapy registration Through the false record, cross-exchange project reported 444,600 yuan for retired cadres medical security funds (of which 99,800 Yuan retired cadres medical security funds in the district, 344,800 yuan) . In accordance with Article 87 of the Social Insurance Law of the People's Republic of China and articles 69,73 and 78 of the agreement on the service of designated medical institutions for the basic medical insurance of Quzhou City, the hospital was ordered to refund 1.4244 million yuan of the medical insurance fund for false items and forged medical documents, and was fined 4.2732 million yuan three times; It ordered the return of 690,800 yuan from the medical insurance fund, which had been reimbursed for non-compliance with the purchase and storage of drugs and irregularities in the management of physiotherapy registration, and imposed a double fine of 1,381,600 yuan, he was ordered to return 99,800 yuan for medical insurance for retired cadres, rescind the hospital's fixed-point service agreement for medical insurance, and report the false charges and forged medical documents to Quzhou Public Security Bureau To inform the Quzhou municipal committee office of veteran cadres of the relevant situation; to transfer to the Lhasa Bureau of Veteran cadres of Tibet Autonomous Region the cases of the false record and cross-swap project involving 344,800 yuan in medical insurance funds for retired cadres from other places; They will refer the medical and technical personnel's qualifications to the Quzhou Municipal Committee on health care for processing.

1. Medical insurance fraud case of East Branch of Pengpai Memorial Hospital, Haifeng County, Shanwei City, Guangdong province

Through the investigation by the joint inspection team of the Shanwei Municipal Medical Security Bureau, the Shanwei Municipal Social Security Bureau, the Haifeng County Medical Security Bureau and the Haifeng County Social Security Bureau, from 2016 to 2019, the eastern branch of Haifeng Pengpai Memorial Hospital defrauded 351,400 Yuan from the medical insurance fund by charging fees for the swap project, the medical expenses that do not meet the medical insurance reimbursement conditions shall be included in the medical insurance illegal declaration medical insurance fund of 702,700 yuan. In accordance with the provisions of Chapter 11, Article 87 of the Social Insurance Law of the People's Republic of China and Article 27 of the regulations of the State Council on the administration of medical institutions, and article 37 of Chapter 5 of the service agreement for designated medical institutions of the basic medical insurance of Shanwei city, ordering the return of 1,054,100 yuan of the medical insurance fund, the hospital was fined 702,800 yuan twice for the charge of cross-changing items, carried out hemodialysis diagnosis and treatment activities beyond the registered scope, prolonged prescriptions without giving reasons for out-patient prescriptions, and nonstandard medical record writing, etc. , they will be referred to the health department for treatment.

1. Medical Insurance fund fraud case of Linxia harmonious hospital in Linxia Hui Autonomous Prefecture of Gansu province

After investigation by Gansu Medical Security Bureau, Linxia Medical Security Bureau and Linxia Medical Security Bureau, linxia harmonious hospital defrauded 43,400 yuan from medical insurance fund in 2019 through false medical records and low standard admission. According to Article 87 of the Social Insurance Law of the People's Republic of China, Linxia Medical Insurance Bureau ordered the hospital to return 43,400 yuan to the medical insurance fund and was fined 130,300 yuan three times The hospital suspended the six-month medical insurance service agreement, ordered its immediate rectification; and the case transferred to the Linxia State Public Security Bureau, Linxia State Commission for Discipline Inspection for processing.

Phase I, 2021,9 cases

1. The case of fraudulent insurance in Jinjing Hospital, Jinzhou City, Liaoning province

After a joint investigation by the Jinzhou Medical Insurance Bureau and the Public Security Bureau, it was found that the director of Jinjing Hospital, surnamed Huang, and many other people inside and outside the hospital used their medical insurance cards of staff, residents and students to apply for false hospitalization by swiping their cards in the Jinjing Hospital medical insurance system, fraudulent medical insurance fund 2906083.15 Yuan illegal facts, the case of all illegal funds 2906083.15 Yuan was recovered and returned to the medical insurance center fund account. The verdict of the local court is as follows: 1. The defendant, Huang Mou, director of Jinjing Hospital, committed fraud and was sentenced to 8 years and 8 months' imprisonment and fined 500,000 yuan. 2. The defendant, surnamed Tian, a legal person of Jinjing Hospital, committed fraud and was sentenced to 9 years' imprisonment and fined 500,000 yuan. 3. The defendant, surnamed Xu, a doctor in Jinjing Hospital, committed fraud and was sentenced to 7 years and 3 months' imprisonment and fined 300,000 yuan. 4. The defendant, Duan mou-mou, a financial officer of Jinjing Hospital, committed fraud and was sentenced to 5 years and 6 months' imprisonment and fined 250,000 yuan. 5. The defendant, surnamed Yang, an employee of Jinzhou Jiutai Pharmaceutical Co. , Ltd. , committed fraud and was sentenced to 4 years in prison and fined 150,000 yuan. 6. The defendant, Han Mou, a teacher of Liaoning petrochemical vocational and technical college, committed fraud and was sentenced to three years in prison, suspended for three years, and fined 50,000 yuan.

1. Changsha city, Hunan province Wangchengpo Chunwang Hospital fraud insurance case

After an investigation by the Changsha Public Security Bureau and the Joint Medical Insurance Bureau, it was found that Li Mou, vice president of Changsha Wangchengpo Chunwang Hospital, and Liu Mou, president and legal person, cheated the medical insurance fund of RMB 4253947.38 Yuan in medicine, laboratory examination, Chinese medicine package treatment and so on. Those involved in the case, surnamed Li and surnamed Zhu, have respectively returned 2 million yuan and 100,000 yuan of illegal income to the Changsha medical security affairs center, the remaining illegal funds, 2153,947.38 yuan, have also been returned to the Changsha Medical Security Service Center. After the court trial sentence as follows: Liu, Li defraud the National Health Insurance Fund, the amount is particularly huge, their acts have constituted fraud, Liu was sentenced to 10 years in prison, and a fine of 100,000 yuan; Li was sentenced to three years in prison, suspended for five years, and fined 70,000 yuan.

1. The case of fraud and insurance fraud in yi-kang traditional Chinese Medicine Hospital of he-shuo county, Bayingolin Mongol Autonomous Prefecture, Xinjiang Uygur Autonomous Region

The Medical Insurance Bureau of Heshu County in Bayingolin Mongol Autonomous Prefecture launched an investigation based on the tip-off clues, it was found that Yang Mou, a legal person of Heshusilu Yikang Chinese Hospital, had falsified medical records, lowered the standard of hospitalization, falsely calculated expenses, abused antibiotics and other illegal facts. He was suspected of using 104,400 yuan of medical insurance money illegally. After the case was transferred to the Public Security Bureau, the person involved in the case, surnamed Yang, was detained by the Public Security Bureau on suspicion of fraud, and all illegal gains were recovered and returned to the medical insurance center fund account. The verdict of the local court is as follows: the defendant and Yang Mou, a legal person of Yi Kang Chinese Hospital of Shuosi Lu, committed fraud and was sentenced to three years in prison, suspended for five years, and fined 100,000 yuan.

1. Huanggang City, Hubei Province, Hong 'an County, qiliping town center hospital state hospital had a fraud insurance case

After a joint investigation by the Hongan County Medical Insurance Bureau and the Public Security Bureau, it was found that a certain Zeng, an employee of the State Hospital -Departmentment of rehabilitation and treatment) of the qiliping town center health center, had worked fAugustgust 1,2018,May may 25,2019, the funds were recovered and returned to the Medicare Center Fund account after the fact that the funds were defrauded by falsifying medical records. The court verdict: the defendant has committed fraud, fraud amount of 264,927.12 yuan, sentenced to three years and six months in prison, and a fine of 100,000 yuan.

1. Hebei province Tangshan Fengrun District Liu Jiaying Township Health Center former medical insurance commissioner and pharmacy toll collector years a certain corruption case

According to an investigation by the Health Insurance Bureau of Fengrun District in Tangshan City, a medical insurance administrator and pharmacy toll collector of Liu Jiaying Township health hospital, Nian Mou, was suspected of fraudulently obtaining medical insurance funds by falsely reporting hospitalization information in 2018. After the case was handed over to the Public Security Department, the Public Security Department further investigated and fixed the relevant evidence, determined the above illegal facts, and recovered the illegal funds of 44884.65 yuan. The verdict was as follows: the defendant, Nian, was sentenced to seven months in prison, suspended for one year, and fined 100,000 yuan for corruption.

1. Inner Mongolia Autonomous Region Chifeng City Ningcheng County one Ken in the township eight Ken in the village Li a fraud case

After investigation by the Medical Insurance Bureau of Ningcheng County in Chifeng City, the existence of the insured person, surnamed Li, intentionally concealed the third-party liability, between 2017 and 2019, he used his health insurance fund to reimburse 38,964.66 Yuan for medical expenses, which should not be paid by the health insurance fund. On April 2,2020, after a local court trial, the verdict was as follows: the defendant, surnamed Li, committed the crime of fraud and was sentenced to a fixed-term imprisonment of one year and six months, suspended for two years, recover the defendant Li Mou illegal funds 38964.66 Yuan returned to the medical insurance center fund account, and a fine of 5,000 yuan.

1. Case of fraud and insurance of a certain Zhu in Lianyungang City, Jiangsu Province

After investigation by the Medical Insurance Bureau of Lianyungang City, it is illegal for insured person BI's daughter-in-law Zhu to intentionally conceal third-party liability and use the medical insurance fund to reimburse medical expenses of 22859.82 yuan (the expenses should not be paid by the medical insurance fund) by means of falsifying the contents of the“Trauma examination form” and so on, suspected of insurance fraud. After the case was handed over to the Haizhou Public Security Bureau of Lianyungang City, it was verified that the above facts were true. Zhu was sentenced to six months in prison, suspended for one year, fined 20,000 yuan and refunded 22,859.82 yuan to the medical insurance center fund account.

1. A fraudulent insurance case of a certain member Yang of Laian County, Chuzhou City, Anhui province

According to the investigation by the Medical Insurance Bureau of Laian County in Chuzhou city, the insured person, surnamed Yang, was suspected of defrauding 155,000 yuan in medical expenses by deliberately falsifying 3 hospital invoices to prepare medical insurance reimbursement. After the case was handed over to the Public Security Bureau, it was verified that the above facts were true. The verdict of the local court is as follows: the defendant, surnamed Yang, committed the crime of fraud (attempted) and was sentenced to three years' imprisonment, suspended for three years, and fined 20,000 yuan.

1. Yunnan province Pu'er City Medical Insurance Management Center Yang Mou embezzlement case

Pu'er City Health Insurance Bureau investigation found that Pu'er City Health Insurance Center, the former Chief of Financial Statistics Yang Mou exists misappropriation of health insurance funds major suspicion. After the case clues transferred to the Pu'er Municipal Commission for Discipline Inspection, public security for further investigation, lock evidence, recover all illegal funds and return to the medical insurance center fund account. The verdict of the local court is as follows: a former chief of the Financial Statistics Department of Pu'er City Medical Insurance Center, Yang Mou, misappropriated public funds RMB 947836.89 yuan, constituting the crime of misappropriating public funds, sentenced to two years in prison, suspended for two years.

Phase II, 2021,10 cases

1. Cases of illegal use of medical insurance funds by mental health centers in Zibo City, Shandong province

According to the investigation by Zibo Medical Insurance Bureau, it was found that the mental health center of Zibo City (the fifth people's Hospital) used the medical insurance funds illegally by inducing the insured to stay in bed, involving 34269.30 yuan. The local medical insurance departments handle the following: 1. Recovery of health insurance funds illegally used by the Zibo Mental Health Center; 2. Request the hospital to rectify within a time limit; 3. The hospital was fined 171,346.50 yuan. At present, the hospital illegal use of medical insurance funds 34269.30 Yuan has been fully recovered, all administrative fines accounted for.

1. the case of fraud insurance in Youhua Hospital, Qingxi, Dongguan City, Guangdong province

An investigation by the Dongguan municipal medical insurance bureau found that the Qingxi Youhua Hospital in Dongguan was suspected of luring insured people to apply for admission through“Free medical examinations” and defrauding health insurance funds by means of forging medical documents and falsely offering medical services, involving 139,817.40 yuan. The local health insurance department dealt with the following: 1. Recovery of ill-gotten health insurance funds 2. Refer the case to the public security department for further processing. At present, Qingxi Youhua Hospital has recovered 139,817.40 yuan of illegal medical insurance funds. After the case was handed over to the public security, the third people's Procuratorate of Dongguan City has brought a public prosecution to the local court, which is in the process of further hearing.

1. The fraudulent insurance case of Dakang Hospital of traditional Chinese medicine in Shuangcheng District of Harbin city, Heilongjiang Province

After investigation by the Harbin Municipal Medical Insurance Bureau, it was found that the Dakang Hospital of traditional Chinese medicine in Shuangcheng District of Harbin was suspected of obtaining medical insurance funds through falsifying medical records and exchanging items, involving 97,969.03 yuan, it was also found that many kinds of drugs (“Cefoperazone sodium and sulbactam sodium for injection”, “Omeprazole injection”, “Bone peptide injection”, etc.) were in violation of the medical insurance limit, involving 72,081.75 yuan. The local health insurance department dealt with the cases as follows: 1. Recovery of health insurance funds obtained and used illegally under the terms of the agreement. To rescind the fixed-point service agreement of medical insurance in Shuangcheng District Dakang Hospital of traditional Chinese medicine, and to notify the illegal acts; 3. The case will be handed over to the public security department for further handling. So far, the hospital has recovered 170,050.78 yuan from the illegal medical insurance funds.

1. Jilin province Changchun Nong 'an County hongtai hospital fraud insurance case

An investigation by the Nong 'an County medical insurance bureau in Changchun found that Hongtai Hospital in Nong 'an County was suspected of obtaining medical insurance funds by means of falsifying medical records, false hospitalization and impersonating patients, involving 105,8991.06 yuan. The local medical insurance department handled the case as follows: 1. Recovery of illegal medical insurance funds from Hongtai Hospital; 2. Lifting the fixed-point service agreement of Hong Tai Hospital; 3. The case will be referred to the public security bureau for further handling. At present, the hospital has recovered 105,8991.06 yuan from the illegal medical insurance funds.

1. Cases of illegal use of medical insurance funds by health centers in Shiqiao town, Lu County, Luzhou City, Sichuan Province

After investigation by Luzhou City Lu County Medical Insurance Bureau, it was found that there were many illegal behaviors of using medical insurance funds, such as low-standard hospitalization, drug use without indication, drug use beyond the scope of indications, repeated check-up, package check-up, over-standard charge, etc. , 345,039.84 yuan was involved. The local health insurance department dealt with the following: 1. To recover the illegal medical insurance funds obtained by the health center of Shiqiao Town, Luxian County; 2. Suspension of medical insurance services in the department of Traditional Chinese medicine and physiotherapy of the hospital for 3 months; 3. The case will be referred to the commission for Discipline Inspection of Luxian County for further handling. The commission's handling of the case is as follows: 1. President Yin was given a warning by the party; 2. Vice-president Tang received a serious warning from the party; 3. Doctors Liang and Xu received a serious warning from the party. 4. Doctors Wang and Xu received administrative demerits. At present, the hospital's illegal medical insurance funds of 345,039.84 yuan have all been recovered.

1. The fraud case of huaren integrated traditional Chinese and Western medicine clinic in Xianju, Taizhou, Zhejiang province

After an investigation by the Xianju County health insurance bureau in Taizhou City, it was found that the Xianju Huaren integrated traditional and western medicine clinic in Xianju County was suspected of defrauding health insurance funds through the use of medical insurance cards, the use of pseudonyms, false memory items, and swapping items, etc. , the total amount involved was 145,673.18 yuan. The local health insurance department dealt with the following: 1. Recovery of illegal health insurance funds from the outpatient department, and deduction of the corresponding penalty according to the agreement; 2. Rescind the outpatient service agreement; 3. Refer the case to the public security department for further processing. At present, the hospital illegally obtained medical insurance funds 145673.18 Yuan has been fully recovered, default fine 223648.76 yuan has been fully accounted for.

1. Hunan Yueyang City Yueyang floor residents Liu Mou Yin mou fraud insurance case

The investigation by Yueyang Tower District Medical Insurance Bureau found that residents surnamed Liu and yinmou were suspected of defrauding medical insurance funds of 15,309.39 Yuan and 27,146.79 yuan respectively by falsifying medical bills to cover medical expenses. After the case was handed over to the Public Security Bureau, the relevant evidence was further consolidated to confirm the above illegal facts. The judgment of the local court is as follows: 1. The defendant, surnamed Liu, was convicted of fraud and sentenced to one year in prison, suspended for one year and six months, and fined 3000 yuan. 2. The defendant, Yin Mou, committed fraud and was sentenced to one year and six months in prison, suspended for two years, and fined 3000 yuan. At present, 42,456.18 yuan of medical insurance funds illegally obtained by Liu and yin have been recovered.

1. Linfen City, Shanxi Province, Xiangning County insured person surnamed Liu medical case

Investigation by the Medical Insurance Bureau of Xiangning County, Linfen City, it was found that the medical insurance card of insured person surnamed surnamed surnamed was used to reimburse the medical expenses in the People's Hospital of Xiangning County during the period from May 30,2019 to June 16,2019, involving 168,270.29 yuan. The local medical insurance department handled the case as follows: 1. Recovery of medical insurance funds illegally obtained by Liu; 2. Deduct 841,351.45 yuan from the default payment of the Xiangning County People's Hospital; 3. Ordering the hospital to rectify within a time limit; 4. To refer the case to the public security department for further handling. At present, 168,270.29 yuan of medical insurance funds illegally obtained by a certain Liu has all been returned, and 841,351.45 yuan of the hospital's penalty for breach of contract has all been accounted for. The local discipline inspection and supervision organs and public security departments have already filed a case, which is being further dealt with.

1. Jiangsu Province Zhangjiagang city insured ou mou fraud insurance case

The investigation by the Zhangjiagang Municipal Medical Insurance Bureau found that the insured ou was suspected of defrauding the medical insurance funds by frequently prescribing a large number of fixed types of prescription drugs in a number of designated hospitals in the city, involving 178,032.28 yuan. After the case was handed over to the Public Security Department, the Public Security Department further investigated and fixed the relevant evidence to determine the above illegal facts. After the local court trial sentence as follows: the defendant ou committed fraud, sentenced to two years in prison, suspended for three years, and a fine of 10000 yuan. At present, 178,032.28 Yuan has been recovered from the illegal medical insurance funds of the insured person.

1. A fraud insurance case of Xiao Mou, a insured person in Qianshan City, Anhui province

After an investigation by the Medical Insurance Bureau of Qianshan City, Anhui province, it was found that a insured person, Xiao Mou, was admitted to hospital on December 10,2018 due to injuries caused by a traffic accident, after receiving compensation from the party responsible for the traffic accident, his son, surnamed Xiao, allegedly stole 34,059.50 yuan from the medical insurance fund on April 28,2019, by deliberately fabricating and concealing the truth and falsely filling in the“Insurance participant's injury condition form”. The local health insurance authorities handled the case as follows: 1. Recovery of medical insurance funds illegally obtained by Xiao. Transfer the case to the Public Security Department for further investigation. At present, 34,059.50 yuan of illegal medical insurance funds obtained by the insured person has been fully recovered.

Stage III, 2021:10 cases

1. Cases of illegal settlement of medical insurance funds by traditional Chinese medicine hospitals in Hainan province

After further investigation by the Hainan Provincial Medical Insurance Bureau based on the clues handed over by the State Flying Inspection Unit, it was found that there were illegal settlement of medical insurance fund in traditional Chinese medicine hospitals in Hainan province, which involved 15694850 Yuan in medical insurance fund. The local health insurance authorities handled the cases as follows: 1. Under the terms of the agreement, the Medical Insurance Fund of Hainan Provincial Hospital of traditional Chinese medicine will be recovered. 2. The hospital is required to rectify the situation within a time limit. At present, the hospital illegal settlement of the medical insurance fund has recovered 10000000 Yuan, the remaining part is further recovery.

1. Cases of illegal settlement of medical insurance funds by Zhengzhou People's Hospital in Henan province

After further investigation by the Medical Insurance Bureau of Henan province according to the clues handed over by the State Flying Inspection Unit, it was found that there were some illegal settlement of medical insurance funds in Zhengzhou People's Hospital, such as repeated charges, swap charges, bundled charges, excessive charges, over-treatment and over-treatment, involving 9381605.25 yuan of medical insurance funds. The local medical insurance departments handled the cases as follows: 1. According to the agreement, the Medical Insurance Fund of Zhengzhou People's Hospital will be recovered. 2. Request the hospital to rectify within a time limit. At present, the hospital's illegal settlement of the medical insurance fund 9,381,605.25 Yuan has all been recovered.

1. the first People's Hospital of Zunyi City, Guizhou province illegal settlement of medical insurance fund case

After further investigation by the Guizhou Provincial Medical Insurance Bureau based on the clues handed over by the State Flying Inspection Unit, it was found that the first People's Hospital in Zunyi City had the illegal settlement of medical insurance fund, which involved 15577068.76 yuan. The local medical insurance departments handled the cases as follows: 1. In accordance with the agreement, the Medical Insurance Fund of Zunyi No. 1 People's Hospital will be recovered. 2. The first People's Hospital of Zunyi City shall be required to make rectification within a time limit. The hospital has recovered 155,777,068.76 Yuan from its health insurance fund.

1. Baoji high-tech Hospital in Shaanxi province illegal settlement of the medical insurance fund case

Further investigation by the Health Insurance Bureau of Shaanxi Province, based on the clues handed over by the National Flight inspection unit, found that Baoji high-tech hospital had the illegal settlement of medical insurance funds, such as double charges, cross-charges and false charges, the medical insurance fund involved 4906579.3 yuan. The local health insurance authorities handled the following: 1. Under the agreement, the medical insurance fund of Baoji high-tech Hospital will be recovered. 2. The hospital is required to rectify the situation within a time limit. At present, the hospital's illegal settlement of the Medical Insurance Fund 4906579.3 Yuan has been fully recovered.

1. the first People's Hospital of Baiyin City, Gansu province illegal settlement of medical insurance fund case

After further investigation by the Health Insurance Bureau of Gansu province based on the clues handed over by the State Flying Inspection Unit, it was found that the first People's Hospital in Baiyin City had illegal settlement of medical insurance fund, which involved 3113236.34 yuan. The local medical insurance departments handled the cases as follows: 1. In accordance with the agreement, the Medical Insurance Fund of the first People's Hospital of Baiyin City will be recovered. 2. The first People's Hospital of Baiyin City shall be required to make rectification within a time limit. At present, the hospital's illegal settlement of the medical insurance fund 3113236.34 Yuan has been fully recovered.

1. Xining City, Qinghai province Third People's hospital illegal settlement of medical insurance fund case

Further investigation by the Qinghai Provincial Medical Insurance Bureau, based on the clues handed over by the National Flight Inspection Unit, found that the third people's hospital had illegal settlement of medical insurance funds, such as over-standard charges, repeated charges, over-charge, over-range drug use, etc. , involving 1829,682.81 yuan. The local health insurance department dealt with the following: 1. In accordance with the agreement, the Medical Insurance Fund of Xining No. 3 People's Hospital will be recovered. 2. Request the hospital to rectify within a time limit. At present, the hospital illegal settlement of the medical insurance fund has recovered 499004.4 yuan, the remaining part is further recovery.

1. Cases of illegal settlement of medical insurance funds by the People's Hospital of Long 'an County, Nanning City, Guangxi Zhuang Autonomous Region

After further investigation by the Guangxi Zhuang Autonomous Region Medical Insurance Bureau based on the clues handed over by the state flight inspection team, it was found that the People's Hospital of Long 'an County in Nanning city had the illegal settlement of medical insurance funds, such as repeated charges, cross charges, decomposed charges, multiple charges, set check-ups and out-of-scope drug use, etc. , involving 9831,778.43 yuan in the medical insurance fund. The local health insurance authorities handled the following: 1. In accordance with the agreement, the medical insurance fund of Longan County People's Hospital will be recovered. 2. Request the hospital to rectify within a time limit. At present, the hospital has recovered 983,1778.43 yuan of its health insurance fund that had been illegally settled.

1. Ningxia Hui Autonomous Region Guyuan City Xiji County People's Hospital illegal settlement medical insurance fund case

After further investigation by the Ningxia Hui Autonomous Region Medical Insurance Bureau based on the clues handed over by the state flying inspection team, it was found that there were illegal settlement of medical insurance fund in Xiji County People's Hospital of Guyuan city, involving 4539843.3 yuan of medical insurance fund. The local medical insurance departments handled the cases as follows: 1. In accordance with the agreement, the medical insurance fund of Xiji County People's Hospital will be recovered. 2. Request the hospital to rectify within a time limit. At present, the hospital's illegal settlement of the Medical Insurance Fund 4539843.3 Yuan has been fully recovered.

1. Xinjiang Production and Construction Corps Shihezi Eighth Division Chinese medicine hospital illegal settlement of medical insurance fund case

After further investigation by the Medical Insurance Bureau of Xinjiang production and Construction Corps according to the clues handed over by the State Flying Inspection Group, it was found that there were illegal settlement of medical insurance fund in Shihezi Hospital of Traditional Chinese medicine, which involved 3140542.52 Yuan in medical insurance fund. The local health insurance authorities handled the cases as follows: 1. In accordance with the agreement, recover the illegal settlement of medical insurance funds by Shihezi Traditional Chinese Medicine Hospital; 2. Request the hospital to rectify the situation within a time limit. At present, the hospital's illegal settlement of the medical insurance fund 314,0542.52 Yuan has been fully recovered.

10. The case of illegal settlement of medical insurance funds by Linzhi Central District Hospital of Tibet Autonomous Region

Further investigation by the Medical Insurance Bureau of the Tibet Autonomous Region, based on the clues handed over by the state flying inspection unit, found that the Linzhi Central District Hospital had illegally settled the medical insurance fund, such as overcharging, repeated charging, set-up tests, etc. , the medical insurance fund involved 117,8968 yuan. The local health insurance department handled the following: 1. In accordance with the agreement, recover the illegal settlement of the insurance fund of the Central Hospital of Linzhi; 2. Requiring the hospital to rectify the situation within a specified period of time. So far, the hospital has recovered 117,8968 yuan from the hospital's health insurance fund.

Stage 4,2021,10 cases

1. cases of illegal settlement of medical insurance funds by Beicheng Hospital of traditional Chinese medicine, Changping District, Beijing

According to the investigation clues provided by other provinces and cities, the Beijing Municipal Medical Insurance Bureau found that there were false claims for the expenses of people enrolled in insurance in different places in Beicheng Hospital of traditional Chinese medicine in Changping District of Beijing, which were not in conformity with the reality, the actual number of patients in hospital does not match with the number of registered patients, the retention of expired diagnostic reagents and other behaviors, involving the medical insurance fund 28,000 yuan. The local health insurance authorities dealt with the following: 1. According to the agreement, recover Beijing Changping District Beicheng Hospital of traditional Chinese medicine illegal settlement of the health insurance fund; 2. To rescind the basic medical insurance service agreement signed with the hospital. At present, the hospital has recovered all of the 28,000 yuan in health insurance funds that were illegally settled by the hospital.

1. Tianjin Nankai Kangtai Hospital illegal settlement of the medical insurance fund case

After further investigation by the Tianjin Medical Insurance Bureau based on the clues handed over by the State Flying Inspection Unit, it was found that there were illegal settlement of medical insurance funds in Nankai Kangtai Hospital in Tianjin, such as unreasonable treatment times, exceeding doctor's orders, charging for days of hospitalization, over-insurance for restricted drug use, over-addition of drugs, irregular management of purchase, sale and storage, etc. , it involved 392,199.26 yuan in the medical insurance fund. The local health insurance authorities handled the following: 1. According to the agreement, recover the illegal settlement of Tianjin Nankai Kangtai Hospital Health Insurance Fund, suspended the medical insurance services for 6 months; 2. The hospital will be required to rectify the situation within a time limit. At present, the hospital's illegal settlement of the medical insurance fund 392,199.26 Yuan has been fully recovered.

1. The case of illegal settlement of medical insurance fund by Baoji Nursing Hospital, Baoshan District, Shanghai

After further investigation by the Shanghai Municipal Medical Insurance Bureau based on the clues handed over by the State Flying Inspection Unit, it was found that there were illegal settlement of medical insurance funds in Baoji Nursing Hospital, Baoshan District, Shanghai, such as repeated charges, over-standard charges, over-payment scope, over-diagnosis and over-treatment subjects, non-indicated tests, and non-compliance of treatment duration, it involved 1162,686.03 yuan in the medical insurance fund. The local health insurance authorities handled the following: 1. In accordance with the agreement, recover Baoji Nursing Hospital in Shanghai Baoshan district illegal settlement of the health insurance fund; 2. To impose an administrative fine of 100,000 yuan on the hospital in accordance with the Shanghai Municipal Measures for the supervision and administration of basic medical insurance; The hospital is required to make rectification within a time limit. At present, the hospital illegal settlement of the medical insurance fund 1162686.03 Yuan has been fully recovered, a fine of 100,000 yuan has been fully accounted for.

1. The case of illegal settlement of medical insurance fund in Zhangzhou Zhengxing Hospital, Fujian Province

After further investigation by the Fujian Medical Insurance Bureau, based on the clues handed over by the National Flight Inspection Unit, it was found that Zhangzhou Zhengxing Hospital was involved in illegal settlement of medical insurance funds, such as overcharging, disassembling charges, overstandard charges and cross-trading charges, the amount involved in the medical insurance fund was 1,273,9795.72 yuan. After deducting 150,1269.97 yuan, which had been deducted from the daily supervision and special inspections, the amount of non-compliance was confirmed to be 1,123,8525.75 yuan. The actions taken by the local health insurance authorities are as follows: 1. In accordance with the agreement, recover the illegal settlement of the health insurance fund of Zhangzhou Zhengxing Hospital; 2. Request the hospital to rectify the problem within a time limit and transfer other relevant issues to the health department. At present, the hospital's illegal settlement of the medical insurance fund 11238525.75 Yuan has been fully recovered.

1. The case of illegal settlement of medical insurance fund by Guangci Hospital of Qianshan County, Shangrao City, Jiangxi province

Further investigation by the Shangrao City Health Insurance Bureau of Jiangxi Province, based on the clues handed over by the national flying inspection team, found that there were illegal settlement of health insurance funds in Guangci Hospital of Qianshan County, such as swapping drugs, swapping medical items, and overcharging, the medical insurance fund involved 107,957.50 yuan. The local health insurance department will handle the following: 1. In accordance with the agreement, recover Shangrao City, Guangci County Hospital Qianshan illegal settlement of the health insurance fund; 2. The total amount involved was 94,997.5 yuan, and the administrative fine was 189,995 yuan twice as much as the standard fees, the amount involved was 12,960 yuan, and the administrative fine was 10,000 yuan. The total administrative penalty imposed on the hospital was $199,995.3. To request the hospital to make rectification within a time limit. At present, the hospital illegal settlement of the medical insurance fund 107957.50 Yuan has been all recovered, a fine of 199995 Yuan has been fully accounted for.

1. Case of illegal settlement of medical insurance fund by Tang County town center health center, Suizhou City, Hubei province

After further investigation by the Hubei Medical Insurance Bureau based on the clues handed over by the State Flying Inspection Unit, it was found that there were some illegal settlement of medical insurance fund, such as unreasonable charge, over-examination, unreasonable drug use and over-limited drug use, which involved 552620 Yuan of medical insurance fund. The local health insurance department handled the cases as follows: 1. In accordance with the agreement, recover Tang County Township Health Center in Suixian illegal settlement of the health insurance fund; 2. Request the hospital to rectify the situation within a time limit. The local health department issued an administrative penalty against the hospital. So far, the hospital has recovered 552,620 yuan from the medical insurance fund that had been illegally settled.

1. Xiantang town health center of Dongyuan County, Heyuan City, Guangdong province illegal settlement of medical insurance fund case

After receiving the audit inquiry letter, the Heyuan Medical Insurance Bureau of Guangdong Province investigated, it was found that there were illegal settlement of medical insurance fund in Xiantang Township Health Center of Dongyuan County, which involved 866,900 yuan of medical insurance fund. The local health insurance department handled the cases as follows: 1. According to the agreement, recover Heyuan City, Dongyuan County, Xiantang town hospital illegal settlement of the health insurance fund; 2. The hospital was fined 1,052,400 yuan. At present, the hospital illegal settlement of the medical insurance fund 866,900 yuan has been all recovered, a fine of 1052,400 yuan has been fully accounted for.

1. The case of illegal settlement of medical insurance fund in Southwest University Hospital of Chongqing

After further investigation by the Chongqing Medical Insurance Bureau based on the clues handed over by the State Flying Inspection Unit, it was found that there were illegal settlement of medical insurance funds in the hospitals of Southwest University, which involved 170,488.51 yuan in medical insurance funds. The local health insurance departments handled the cases as follows: 1. In accordance with the agreement, recover the illegal settlement of the medical insurance fund of Southwest University Hospital in Chongqing; 2. 2. Deducting the liquidated damages of 461,283.23 yuan; 3. The hospital is required to make rectification within a time limit. At present, the hospital illegal settlement of the medical insurance fund 170488.51 Yuan has been fully recovered, default fine 461283.23 yuan has been fully accounted for.

1. Aier Eye Hospital Group Company Limited

After further investigation by the Medical Insurance Bureau of Yunnan Province based on the clues handed over by the State Flying Inspection Unit, it was found that the Aier Eye Hospital Group Company Limited had the illegal settlement of medical insurance funds, such as the repeated charge, the decomposition charge, the swap charge, the over-examination, the over-payment of medical insurance conditions, the violation of the scope of diagnosis and treatment and the inclusion of medical insurance reimbursement, and the non-compliance of some consumables in the purchase and sale of medical insurance funds, the medical insurance fund involved 265,9019.71 yuan. The local health insurance department will handle the following: 1. In accordance with the agreement, the recovery of Aier Eye Hospital Group Company Limited's illegal settlement of health insurance funds; 2. Request the hospital to rectify the situation within a time limit. At present, the hospital illegal settlement of the medical insurance fund has recovered 680707 Yuan, the remaining part is further recovery.

10. Xinjiang production and Construction Corps 13th Division Xinxing Rocket Farm Xin's traditional Chinese medicine dermatology hospital illegal settlement medical insurance fund case

The Medical Insurance Bureau of Xinjiang Production and Construction Corps found during a flight inspection that the 13th Division Xinxing Rocket Farm Xin's traditional Chinese medicine dermatology hospital was involved in illegal settlement of medical insurance funds, such as inducing hospitalization and forging medical documents, the medical insurance fund involved 1245,896.90 yuan. The local health insurance department will handle the following: 1. According to the agreement, the Recovery Corps 13 Division Xinxing City Rocket Farm Xin's Chinese medicine dermatology hospital illegal settlement of the medical insurance fund; 2. To impose administrative penalties on the hospital; 3. To rescind a health insurance service agreement with the hospital; 4. The case has been referred to the local public security department for further investigation. So far, the hospital has recovered 12,458,96.90 yuan from the illegal settlement of its medical insurance fund.

Stage 5,2021,10 cases

1. Henan province Zhengzhou Sixth People's Hospital cheats the insurance case

In April 2021, the Medical Insurance Bureau of Zhengzhou City, Henan Province, based on a real-name investigation, found that the sixth people's Hospital of Zhengzhou City had a problem with the use of pedicle screws in surgical records and actual implantation, it caused a loss of 1741,491.50 yuan to the health insurance fund. In accordance with the social insurance law of the People's Republic of China, the service agreement of the designated medical institutions of Zhengzhou Municipality for medical security, and the provisional measures of Zhengzhou Municipality on the administration of medical insurance physicians in designated medical institutions of the basic medical insurance, the results of the local health insurance departments are as follows: 1. The hospital's Director of Orthopaedics, Chen Mou, director of the Department of Bone Tuberculosis, Qian Mou, and other responsible persons shall be disqualified from paying for health insurance services; 2. Since midnight on April 28,2021, stop the hospital orthopaedics and Tuberculosis Department of the Health Insurance Fund settlement; 3, recovery of the loss of Health Insurance Fund, and 5 times the fine; 4. The clues of relevant problems shall be handed over to the Commission for Discipline Inspection and supervision of Zhengzhou Municipal Health Commission, Municipal Public Security Bureau and Municipal Market Supervision Bureau, etc. . At present, the Zhengzhou Commission for Discipline Inspection in the City Health Commission supervision team has to the hospital director of Orthopaedics Chen, director of Medical Equipment Lei to review the case (investigation) ; The 27th branch of Zhengzhou Public Security Bureau has opened a case to investigate this clue; the lost medical insurance fund of 1741,491.50 Yuan has all been returned, and the five-fold fine of 870,7457.50 Yuan has all been executed.

1. Non-compliance cases in Chifeng City Hospital of Inner Mongolia Autonomous Region

In August 2020, after the Inner Mongolia Autonomous Region Chifeng City Medical Security Bureau receives the autonomous region to transfer the State Medical Security Bureau to report the clue investigation, it was found that Dou mou, director of the Department of Rehabilitation Medicine in Chifeng City Hospital, had some illegal behaviors, such as the charge of swap items, the treatment items not in accordance with the contents of the items, and so on, which involved 1485982.16 Yuan of the medical insurance fund. According to the“Chifeng city basic medical insurance designated medical institutions medical service agreement”, the results of the local medical insurance departments are as follows: 1, suspended the hospital director of the Department of Rehabilitation Medicine Doumou medical insurance payment eligibility for 6 months; 2. Recovering the illegal settlement of the hospital's Medical Insurance Fund 3. The Chifeng Medical Security Bureau conducted interviews with the Chifeng Hospital and informed it within Chifeng city and exposed it in the media; Order the hospital to rectify the problems within a time limit. At present, the hospital's illegal settlement of the medical insurance fund 1485,982.16 Yuan has been returned.

1. the Second Hospital of Fuzhou in Fujian Province

In November 2020, a joint investigation by the Fujian Provincial Medical Insurance Bureau and the Fuzhou Municipal Medical Insurance Bureau found that the ultrasound department of the Second Hospital of Fuzhou did not conduct strict examination of patients, resulting in such irregularities as fake card visits to the hospital, the medical insurance fund involved 37,478.56 yuan. According to the measures of Fuzhou municipality on the investigation and punishment of violations of the basic medical insurance, the results of the local health insurance departments are as follows: 1, the hospital in batches of suspension of 9 doctors, such as Lin, Peng, Gao, etc. 2. If the insured person involved in this case exceeds the period of 2 years stipulated by the administrative penalty, the counterfeit card fund shall be recovered for processing; if an individual insured person has repeatedly forged a card, to recover the illegal funds, listed in the key supervision list and change the way the settlement of medical insurance; 3, the hospital to recover the illegal settlement of medical insurance funds. At present, the hospital has already returned all the illegal settlement of 37,478.56 yuan of the medical insurance fund.

1. People's Hospital of Sichuan province

In February 2021, the Medical Insurance Bureau of Jinjiang District, Chengdu City, Sichuan Province, found that the fourth people's Hospital of Sichuan province had unspecified examinations, digital photography and film without report forms, general special care, ultrasonic nebulization, bladder irrigation without doctor's advice or doctor's advice and charges inconsistent with the settlement of the health insurance fund, involving 24,745.49 yuan. According to the notice of Chengdu Medical Insurance Administration on strengthening the management of medical insurance physician agreements, the results of treatment by the local medical insurance department are as follows: 1. The Doctor Zhang did not record the medical record according to the basic standard of medical record writing, and did not record the medical record accurately, cao failed to comply with the provisions of the insurance personnel (family members) informed consent and signature system, each deduction of physician points 1 point; 2, recovery of illegal settlement of the medical insurance fund, as well as deduction of default payments; 3, interview the hospital responsible person, ordered the deadline for rectification, etc. . At present, the hospital's illegal settlement of the medical insurance fund 24,745.49 Yuan and default payments have all been paid.

1. The first People's Hospital of Taizhou City, Zhejiang province

In June 2020, the medical insurance department of Taizhou City, Zhejiang province, found during a routine examination that the first People's Hospital of Taizhou had patients who were hospitalized with hanging beds or decomposed, the medical insurance fund 17733.04 Yuan was involved in such illegal acts as lowering the admission standard and admitting the insured who did not meet the admission indication. According to the implementation rules for the administration of medical insurance physician agreement in Zhejiang Province and the service agreement for designated medical institutions of basic medical insurance in Huangyan District of Taizhou City, the results of the local medical insurance department are as follows: 1. Sheng, Ding and Huang were deducted 2 points and 4 points respectively 2. Recover the illegal settlement of the medical insurance fund, and deduct the penalty in accordance with the regulations. At present, the hospital has already paid 17,733.04 yuan into the medical insurance fund that has been illegally settled and the default penalty.

1. Illegal cases of traditional Chinese medicine hospital in Langfang City, Hebei province

In August 2020, nine doctors at Langfang Hospital of Traditional Chinese medicine, including Wang, Zhang and Li, were found to have issued“Large prescriptions” in violation of regulations when they carried out a special treatment and examination in the field of medical insurance in Langfang City, Hebei province, the medical insurance fund of RMB 2017,176.16 was involved in the illegal practice of prescribing drugs for the treatment of prostate cancer for female patients. In accordance with the administrative measures of medical insurance service doctors in cities and towns of Langfang City, the administrative measures of prescriptions, and the medical service agreement of designated medical institutions of Hebei medical insurance, the results of the local health insurance departments are as follows: 1, suspension of Wang Mou, Zhang Mou, Li Mou and other 9 doctors medical insurance service payment eligibility; 2、 Stop the settlement of Medical Insurance Fund of Special Disease Clinic of Langfang Hospital of Traditional Chinese Medicine; 3、 recover the illegal settlement of Medical Insurance Fund; 4、 coordinate the investigation and review procedure of Doctor Wang Mou by the Discipline Inspection Committee of Langfang City; 5. The clues were handed over to the local public security organs, and the Langfang Public Security Organs took coercive measures against the five persons suspected of constituting crimes, and the case is being further investigated. At present, the hospital's illegal settlement of the medical insurance fund 2017,176.16 Yuan has been returned.

1. Illegal cases in Qinzhou Traditional Chinese Medicine Hospital of Guangxi Zhuang Autonomous Region

In May 2021, after an investigation by the medical insurance bureau of Qinzhou City, Guangxi Zhuang Autonomous Region, it was found that there were some problems in Qinzhou Hospital of traditional Chinese medicine from January 2020 to April 2021, such as cross-item charge, over-examination and over-treatment, which involved 1995547.80 Yuan of medical insurance fund. After investigation, it was found that the above illegal problems were mainly caused by the weak concept of medical insurance laws and regulations of medical staff in the first and second districts of the Department of acupuncture and Moxibustion and the department of massage, and the confusion of patient management, in particular, the Department of Acupuncture Doctors Zhangmou, Zhaomou and massage section 2 Zhong Mou, Han Mou and other 11 people, did not seriously perform the duties of doctors. In accordance with the circular of the office of human resources and Social Security of the Guangxi Zhuang Autonomous Region on the issuance of the provisional measures for the administration of medical insurance service doctors in designated medical institutions with basic medical insurance in Guangxi and the agreement on medical services in designated medical institutions with basic medical insurance in Qinzhou, the results of the local health insurance departments are as follows: 1, the hospital suspended the first district doctors Zhang, Zhao and massage section of the second district Zhong, Han, etc. 11 people 3 months of health insurance eligibility; 2. To suspend the settlement of the medical insurance fund for three months in the first and second districts of the acupuncture and Moxibustion Department of the hospital; 3. To recover the medical insurance fund illegally used; 4. To transfer the clues of the hospital's violation to the municipal commission for discipline inspection, etc. . At present, all medical insurance funds that have been illegally settled by the hospital have been returned.

1. Jilin province Liaoyuan City Xiangrui elderly rehabilitation hospital illegal case

In April 2021, after investigation by the Liaoyuan Municipal Medical Insurance Bureau of Jilin province, it was found that there were illegal settlement of medical insurance fund in Liaoyuan Xiangrui Hospital, which involved 1.05 million yuan of medical insurance fund. According to“Liaoyuan city basic medical insurance designated medical institutions services”, the results of the local medical insurance departments are as follows: 1. Illegal doctors in the hospital to suspend the medical insurance payment eligibility for 1 year; 2. Recover the illegal settlement of the hospital's medical insurance funds; 3. Hand over the clues to the local public security organs. At present, the hospital has recovered all 1.05 million yuan of the illegal settlement of the medical insurance fund.

1. Illegal cases of Phoenix Hospital, Eastern Hospital Group, Huainan City, Anhui province

In January 2021, the Anhui Medical Insurance Bureau's Flight Inspection team conducted a special spot check on the Phoenix Hospital of Huainan Dongfang hospital group and found that, the illegal settlement of medical insurance funds in Phoenix Hospital, Huainan Oriental Hospital Group, involved 676907.79 Yuan in medical insurance funds. In accordance with the implementation rules of the medical insurance agreement of Huainan municipality for the management of physicians (trial implementation) and the medical service agreement of the residential designated medical institutions for the basic medical insurance of Huainan Municipality (trial implementation) , the results of the local medical insurance department are as follows: (1) deduct 12 points from the hospital doctor Zang's annual assessment score, suspend the eligibility for payment of medical insurance services this year; 3. Ordering the hospital to make rectification and submit a rectification report. At present, 676,907.79 yuan of the hospital's medical insurance fund has been returned.

1. Disobeying regulations of zhongxintang traditional Chinese Medicine Hospital of Dalian City, Liaoning province

In January 2021, after investigation by Dalian Municipal Medical Insurance Bureau, Dalian Zhongxintang TCM Hospital Doctors Wu Mou and Zhao Mou were found, sun, Cheng, Zhou, Gaimou and other 6 people have medical records, the actual operation and the occurrence of medical costs do not match and reduce the standard of hospitalization and other problems. As an assistant physician, Zhang should provide medical services under the guidance of a medical practitioner because he or she has acquired the medical qualification from a teacher or has expertise. The above-mentioned problem involves a total of 104,109.7 yuan from the health insurance fund. According to the medical service agreement of the designated medical institutions of Dalian Medical Insurance (2020 edition) and the administrative measures of Dalian Medical Service doctors, the results of the local medical insurance department are as follows: 1.4 points were deducted from the doctors of the hospital, such as Wu, Zhao, sun and Cheng, and 8 points were deducted from Zhou and gai, 2. To suspend the settlement of Medical Insurance Fund for 6 months in the in-patient ward of the hospital (First Department of Chinese Medicine, Second Department of Chinese Medicine) ; 3. The illegal expenses will not be settled and will be double checked and reduced, and the total amount of illegal settlement of the medical insurance fund will be recovered 332529.1 yuan; 4. The hospital will be ordered to rectify within a time limit, etc. . At present, the hospital has already returned all the illegal settlement of medical insurance funds. Dalian xigang District Health Bureau to the hospital assistant doctor Zhang gave a fine of 3000 yuan, suspended for 6 months of administrative penalties.

Stage 6,2021,9 cases

1. Illegal settlement of medical insurance funds by Fengcheng Junkang Hospital, Yichun City, Jiangxi province

In March 2021, the Fengcheng Medical Security Bureau of Yichun City, Jiangxi province, found after an investigation based on clues from mass reports, there are some problems in Yichun Fengcheng Jun Kang Hospital, such as the discrepancy of witnesses, using fake cards to seek medical treatment, failing to keep the records of medicine and medical consumables in and out of storage according to the regulations. The above problems involve the medical insurance fund of 436,600 yuan. In accordance with the law of the People's Republic of China on social insurance and the agreement on medical services for designated medical institutions under the basic medical insurance of Yichun Municipality, the results of the local medical insurance department are as follows: 1. Recover the illegal settlement of the hospital's Medical Insurance Fund and impose a double administrative fine according to law; 2. Order the hospital to strengthen the medical insurance management and rectify the existing problems within a time limit; 3. To issue a reward of 8,732.9 yuan to those who provide tips on the case. At present, the loss of 436,600 yuan from the medical insurance fund has been recovered, and the administrative fine of 873,200 yuan has been paid in full.

2. The case of illegal settlement of medical insurance fund by Laixiwan home hospital in Qingdao, Shandong province

In April 2021, the Qingdao Municipal Medical Security Bureau found that the lacivan home hospital had been operating from January to September 2019 and from February to May 2020, there are some illegal and illegal acts, such as falsifying medical records, medical orders not in accordance with actual treatment or charge, bringing self-paid examination items into overall planning and payment, identical examination reports, no indication or repeated examination and treatment, using drugs beyond the limited scope, and not in accordance with the facts of drug accounts, etc. , the above-mentioned problems involved a total of 158,990.95 yuan from the health insurance fund. In accordance with the social medical insurance measures of Qingdao Municipality, the administrative measures of the social medical insurance service doctors of Qingdao Municipality, and the medical service agreement of the residential designated medical institutions for social medical insurance and maternity insurance of Qingdao Municipality, the results were as follows: (1) recover the lost medical insurance fund and impose administrative fine according to law; (2) give the relevant medical insurance doctors a score deduction; (3) rescind the medical insurance service agreement signed with the hospital; 4. To transfer the fraudulent insurance acts involved in falsifying medical records to the local public security organs for investigation and handling according to law. At present, the lost medical insurance fund of 158,990.95 Yuan has been recovered, and the administrative fine has been paid in full.

3. The case of illegal settlement of medical insurance fund by Fenghuang Yimin Tongji Hospital, Fenghuang County, Xiangxi Prefecture, Hunan province

In July 2018, the Medical Insurance Bureau of Fenghuang County in Xiangxi Prefecture, Hunan province, found that fenghuang for the People's Tongji Hospital was suspected of fraudulent insurance. The Fenghuang County Medical Insurance Bureau immediately suspended the fixed-point medical insurance agreement signed with the hospital, and to the Fenghuang County Commission for Discipline Inspection Transfer of suspected fraud clues, with the county commission for Discipline Inspection, the County Public Security Bureau set up a special team to carry out joint investigations, and through third-party audit to carry out a comprehensive investigation and evidence collection work. In September 2021, the Intermediate People's Court of Xiangxi Prefecture issued a final judgment, ruling that fenghuang for the People Tongji Hospital had used such means as hanging empty beds, prolonging the length of stay in the hospital, and falsely using medicines from July 2016 to July 2018, to defraud the health insurance fund of 569,000 yuan, sentence Wu, the former legal person of the hospital, to 15 years in prison for the crime of fraud, recover illegal income, and confiscate 1,000,000 yuan of personal property, to other case personnel Wu Mou, Tian Mou and so on doctor, nursing, finance and so on total 11 people, all by swindles the crime to give the criminal penalty separately. At the same time, the court of Fenghuang County imposed criminal penalties on a public official involved in the case. The county commission for Discipline Inspection imposed discipline on nine public officials for failing to perform their duties. At present, the Phoenix County Medical Insurance Bureau has been lifted and the hospital signed the health insurance fixed-point agreement, the loss of the health insurance fund 5690000 Yuan has been fully recovered.

4. Case of illegal settlement of medical insurance fund by Yihe Rehabilitation Hospital of Dongyuan County, Heyuan City, Guangdong province

In August 2020, the Department of Health Insurance in Heyuan City, Guangdong province, conducted a field inspection at the Yihe Rehabilitation Hospital in Dongyuan County. It was found that there were cases of low-standard admission, high scores, and over-indications for physical therapy in the hospital, a total of 278,591.7 Yuan was involved in the problems. According to the regulations of Guangdong province on the supervision of social insurance funds, the results of the local medical insurance departments are as follows: 1. Recover the lost medical insurance funds and impose administrative fines according to law; 2. Suspension of the medical insurance service agreement of the Rehabilitation Department of the hospital for three months. So far, 278,591.7 Yuan of the lost health insurance fund has been recovered and the administrative fine of 564,579.92 Yuan has been fully paid.

5. The case of illegal settlement of medical insurance fund by the Central Hospital of Nanmuda Town, Rantang County, Aba Prefecture, Sichuan province

In April 2021, the Medical Security Bureau of Rantang County, Aba Prefecture, Sichuan province, after receiving a tip-off clue, made a comprehensive investigation and found that, there are some illegal activities in the Central Hospital of Nanmuda Town, Rantang County, such as forging medical documents, exchanging drug charges, destroying medical documents, making up medical service items, and not strictly enforcing the“Zero-addition” of drugs. Between January 2020 and March 2021, illegal settlement of medical insurance funds amounted to 308,590.43 yuan. According to the“Social Insurance Law of the People's Republic of China”, the results of the local medical insurance departments are as follows: 1. Recover the illegal settlement of the hospital's medical insurance funds, and impose administrative fines according to law; 2. The relevant clues of violation of law and discipline shall be handed over to the local discipline inspection and supervision organs and health departments for further treatment. At present, the lost medical insurance fund of 308,590.43 Yuan has been recovered, and the administrative fine has been paid in full.

1. Cases of illegal settlement of medical insurance funds by modern maternity hospitals in Lhasa City, Tibet Autonomous Region

In May 2021, the Medical Security Bureau of Lhasa, Tibet Autonomous Region, received a tip from the Medical Security Bureau of the autonomous region, indicating that the Lhasa modern maternity hospital was suspected of cheating insurance by using the fourth-floor health club. Lhasa City Medical Security Bureau after secret visits with the City Health Commission, Market Supervision Bureau and other departments on the hospital carried out on-site investigation and verification and evidence collection. It has been found that there are illegal and illegal acts in the hospital, such as exchanging medical treatment items, including medical expenses that are not covered by the medical security fund in the settlement of the medical security fund, and personnel engaged in physiotherapy without relevant credentials, the total amount of medical insurance fund involved was 397,485.6 yuan. According to the regulations on the supervision and administration of the use of the medical insurance fund, the results of the local medical insurance departments are as follows: 1. The medical insurance fund that has been illegally settled by the hospital will be recovered and the administrative fine will be doubled according to law; 2. Ordering the hospital to suspend payments from the medical insurance fund for Traditional Chinese medicine for 12 months. At present, the lost medical insurance fund of 397,485.6 Yuan has been recovered, and the administrative fine of 794,971.2 Yuan twice has been paid in full.

7. The case of illegal settlement of medical insurance fund by Xiashi Hospital of Traditional Chinese medicine in Kunming, Yunnan Province

In April 2020, the Kunming municipal medical insurance administration and third-party regulatory bodies and the Xishan District Medical Insurance Bureau conducted on-site inspections of the Kunming Xiashi Hospital of traditional Chinese medicine to verify the existence of problems in the hospital with false claims of drugs and false claims of tests, the total amount of money involved was 106,700 yuan. According to the Social Insurance Law of the People's Republic of China, the results of the treatment by the local medical insurance departments are as follows: 1. Recovery of the lost medical insurance funds and imposition of twice the administrative fine according to law; 2. Ordering the hospital to strengthen the management of medical insurance, to rectify the existing problems within a time limit. So far, 106,700 yuan of lost health insurance funds have been recovered, and 213,400 yuan of administrative fines have been paid in full.

8. Ningxia Hui Autonomous Region Zhongwei City Renai hospital illegal settlement of the medical insurance fund case

In June 2020, the Ningxia Hui Autonomous Region's medical security bureau found that between August 2017 and June 2020, there were 4442,800 yuan defrauded from the medical insurance fund by forging drug purchase and sale receipts and falsely issuing ordinary invoices for value-added tax, and 179,600 yuan defrauded from the medical insurance fund by fabricating medical records, reducing hospitalization indications, and lying in hospital. All of the above involved losses of 4,622,400 yuan from the health insurance fund. According to the Social Insurance Law of the People's Republic of China, the results of the treatment by the local medical insurance departments are as follows: 1. Recovery of lost medical insurance funds and imposition of administrative fines in accordance with the law; 2. Rescission of the medical insurance service agreement signed with the hospital, 3. Inform the local tax and health authorities of the investigation and punishment of the hospital's fraud in obtaining medical insurance funds, and transfer the relevant evidence materials for investigation to the local public security organs. At present, the public security organs have filed a case for investigation, the loss of the medical insurance fund 4622400 Yuan has been recovered, the administrative fine has been paid in full.

9. Illegal settlement of medical insurance funds by Bazhou Geriatrics Hospital of Xinjiang Uygur Autonomous Region

In November 2020, the Xinjiang Uygur Autonomous Region Bazhou Medical Security Bureau found in the daily audit of the Bazhou geriatric hospital fees suddenly increased. After thorough investigation and evidence collection by Bazhou Medical Insurance Bureau, it was found that the following problems existed in the hospital: 1. Fabricating medical documents: most of the medical records in the hospital had the same contents, the basic information was wrong, and the medical records did not match the patients' conditions; 2. False hospitalization: the Act of using medical insurance card for non-designated organization to obtain medical insurance pooling fund; 3. inducing the patients to take the whole treatment: in the investigation, it was found that the patients in the hospital only need to pay 300 yuan or 400 yuan for the whole treatment every time they were admitted to the hospital, the above questions related to the health insurance fund 52,500 yuan. According to the Social Insurance Law of the People's Republic of China, the results of treatment by the local medical insurance departments are as follows: 1. Termination of the medical insurance service agreement of the Bazhou Geriatrics Hospital; 2. Recovery of lost medical insurance funds; (3) the case will be referred to the local judicial authorities. So far, 52,500 yuan of the lost medical insurance fund has been recovered, and 210,000 yuan of the four-fold administrative fine has been paid in full.

24 cases in 2022 Phase I

Medical security fund is the people's“Medical money”, “Life-saving money”, its use safety involves the immediate interests of the masses, the relationship between the health and sustainable development of the medical security system. In order to crack down on individual fraud and insurance fraud, the national health insurance administration has selected 24 typical cases of individual fraud and insurance fraud, it involves illegal acts such as seeking medical treatment under false name, repeatedly enjoying medical insurance benefits, over-prescribing drugs and reselling them, false bills, concealing third-party liability, etc. . The above-mentioned acts of defrauding medical insurance funds by illegal means have seriously disturbed the management order of the State Medical Security and endangered the vital interests of the people in medical security, and should be severely punished according to law and regulations. Here, the state medical insurance bureau reminds each insured person, maintains the medical insurance fund security, is related to you and I, everybody has the responsibility. Please strengthen the law and risk awareness of insurance personnel, properly take care of their medical insurance card (medical insurance electronic vouchers) , do not rent, do not lend, we should consciously resist such illegal activities as seeking medical treatment under false names, using false bills to claim expenses, prescribing drugs in excess and reselling them, enjoying medical insurance benefits repeatedly, and fraudulently obtaining medical insurance funds by forging certificates. If you find any fraud, please report it to the local medical insurance department. The medical insurance department will reward you according to the regulations and protect the people's“Life-saving money”.

1. A case of fraud by a certain MA in Miyun District, Beijing

In July 2021, the Beijing Municipal Medical Security Bureau received a tip-off clue that a Miyun district insured person, Ma Mou, was suspected of repeatedly using his and others' social security cards to seek medical treatment, purchase medicines and sell medicines. The Beijing Medical Insurance Bureau immediately opened an investigation into the case, it was found that from April 2019 to October 2020, Ma repeatedly used his, Liu's, Li's and Zhang's social security cards to visit hospitals, purchase medicines and sell them. Upon enquiry, Ma admitted breaking the law. After accounting, Ma found out that he had cheated 51,624 yuan out of his health insurance fund. In accordance with the social insurance law of the People's Republic of China, the Beijing Municipal Medical Insurance Bureau ordered the person concerned, Ma Mou, to return the fraudulent medical insurance fund upon delivery of the decision on administrative punishment, and imposed an administrative fine of double the amount of money defrauded. According to the criminal law of the People's Republic of China, the Miyun District People's Court made a judgment according to law, and the defendant, Ma Mou, committed the crime of fraud and was sentenced to one year in prison. At present, the loss of the health insurance fund 51624 Yuan has been fully recovered, double the administrative fine 103248 Yuan has been fully recovered.

1. Tianjin Jizhou district Cui certain fraud case

In 2019, the Tianjin Medical Security Bureau received a tip-off clue, reflecting Jizhou district insured Cui Mou suspected of defrauding health insurance funds through false bills claims. After investigation, Cui was suspected of falsifying the medical records and bills of the 301 Military Hospital of the People's Liberation Army in order to seek medical treatment in other places and defraud medical insurance funds, which was suspected of constituting the crime of fraud, tianjin medical insurance bureau will be suspected of fraud Cui a medical insurance fund case materials and clues to information transferred to the public security organs for processing. After verification, between 2016 and 2018, Cui and Li colluded in holding the medical records and receipts of 301 Military Hospital of the People's Liberation Army that were forged by Li using his identity information, he defrauded his medical insurance fund for 39,339.31 yuan four times. At the same time, from 2014 to 2018, Li used his daughter's identity information to falsify the medical records and bills of the 309th Hospital of the People's Liberation Army, claiming 211,715.48 yuan from the medical insurance fund nine times; In 2018, Jiang conspired with Li to defraud the medical insurance fund of 22,114.98 yuan by holding the medical records and bills of the 309th Hospital of the People's Liberation Army that Li falsified with his identity information In 2018, Liang conspired with Li to defraud the health insurance fund of 12,568.91 yuan by holding the medical records and bills of the General Hospital of the People's armed police, which Li falsified with his identity. In accordance with the criminal law of the People's Republic of China, in July 2020, the People's Court of Jizhou district of Tianjin made a judgment according to law that four defendants, Cui, Li, Jiang and Liang, committed the crime of fraud, they were sentenced to a term of imprisonment ranging from seven months to five years and six months, and fined accordingly. Cui and other four people defrauded the health insurance fund in the court has been effective after all returned to the health insurance fund account.

1. The insurance fraud case of Dong Mou in Yuncheng City, Shanxi province

In May 2020, the Medical Security Bureau of Pinglu County, Yuncheng city, Shanxi province, found that an urban and rural insured person, surnamed Dong, allegedly provided false reimbursement materials to defraud medical insurance funds when reviewing claims made by urban and rural residents in 2019. The Pinglu County medical insurance bureau immediately arranged for staff to check Dong's medical insurance claims. On May 11,2020, the staff of Pinglu County medical insurance bureau went to Xi'an Xijing Hospital for on-site investigation, and found that the reimbursement materials submitted by Dong on October 10,2019 were all forged. On May 14,2020, Pinglu County medical insurance bureau staff to Dong Mou and the agent Ning mou for inquiries, two answers to the hospital and reimbursement are inconsistent. After verification, the hospital reimbursement materials submitted by Dong were forged, and his behavior was suspected of defrauding the medical insurance fund, involving 18,089.21 yuan for reimbursement of basic medical insurance and 12,749.60 yuan for reimbursement of serious disease insurance. According to the Social Insurance Law of the People's Republic of China, pinglu County medical insurance bureau on May 15,2020, Dong Mou defrauded the basic medical insurance reimbursement of 18,089.21 Yuan and serious disease insurance reimbursement of 12,749.60 yuan all recovered. On May 22,2020, the Pinglu County medical insurance bureau transferred the case materials and clue information of a certain Dong to the public security organ for processing. On November 8,2021, the case was handed over by the local Public Security Bureau to the Procuratorate for prosecution.

1. A fraud case of Liu in Tongliao City, Inner Mongolia Autonomous Region

In May 2021, the medical insurance service center of Tongliao City, Inner Mongolia Autonomous Region, when checking the verification procedures for off-site patients, found that the medical bills provided by insured Liu had problems and were suspected of defrauding the medical insurance fund. After verification, Liu provided 5 hospital medical bills are false bills, bills amount of 72428.41 yuan, the total payment of 61207.84 Yuan Health Insurance Fund. According to the“Social Insurance Law of the People's Republic of China”, Tongliao City Medical Insurance Bureau deal with the results as follows: 1, ordered Liu to return the fraudulent medical insurance funds; 2. The case materials and clue information of a certain Liu who is suspected of defrauding the medical insurance fund shall be transferred to the public security organ for processing. At present, 61,207.84 yuan of the lost medical insurance fund has been recovered.

1. Liaoning province Benxi Zhangmou insurance fraud case

In March 2021, the Medical Security Bureau of Huanren Manchu Autonomous County, Benxi City, Liaoning province received a tip-off clue, reflecting the Huanren Manchu Autonomous County insured Zhang in 2017, using her husband, MA, a motorcycle accident after alcohol fraud medical insurance fund. A joint investigation by the Huanren Manchu Autonomous County Medical Insurance Bureau and the County Public Security Bureau found that Zhang had falsified documents proving that her husband, MA, had been injured in a bicycle crash in January 2017 in order to defraud medical insurance funds, between March and May 2017, he visited the Huanren Manchu Autonomous County Social Security Administration to defraud 49,087.99 Yuan from the medical insurance fund through reimbursement of medical expenses on two occasions. According to the Social Insurance Law of the People's Republic of China, the Huanren Manchu Autonomous County Medical Insurance Bureau ordered Zhang to return the fraudulent medical insurance fund. In accordance with the criminal law of the People's Republic of China, the People's Court of Huanren Manchu Autonomous County issued a verdict according to law, in which the defendant, Zhang, committed the crime of fraud and was sentenced to 10 months' imprisonment and a fine of 10,000 yuan. At present, the lost medical insurance fund of 49,087.99 Yuan has been fully recovered.

1. Jilin province Songyuan City Guo mou insurance fraud case

In May 2021, the Medical Security Bureau of Songyuan City, Jilin province, received the notice of the Medical Security Bureau of Jilin province on the verification of double reimbursement, and found that a insured person in Fuyu City, Guo Mou, claimed reimbursement by purchasing fake bills, he was suspected of repeatedly enjoying medical insurance benefits. From June 11 to 17,2021, the staff of the Capital Medical University affiliated Beijing Tiantan Hospital, the Medical Security Bureau of Beijing Fengtai District, and the Medical Security Bureau of Beijing Chaoyang District conducted on-site verification, it was found that the same time in 2018, Guo was reimbursed as an employee at Chaoyang District Medical Insurance Administration Center in Beijing, and as an urban and rural resident in Fuyu City, which involved double reimbursement of 45,058 yuan from the medical insurance fund. According to the Social Insurance Law of the People's Republic of China, on June 21,2021, the Medical Insurance Bureau of Fuyu City transferred the case materials and clue information of a certain Guo who was suspected of defrauding the medical insurance fund to the public security organ for processing. At present, 45,058 yuan of the lost medical insurance fund has been recovered.

1. A fraud case of Liu Mou in Harbin, Heilongjiang Province

In November 2019, the medical insurance center of Harbin city in Heilongjiang province received medical insurance reimbursement materials from insured Liu Mou, and verified that the medical records submitted by Liu Mou were suspected of being forged, an in-depth investigation later found that Liu had also submitted fake medical records for reimbursement in November 2018. Harbin Medical Insurance Center will be the case clues handed over to the Harbin Medical Security Bureau for further processing. After investigation by Harbin Medical Insurance Bureau, it was found that the first affiliated hospital of Dalian Medical University did not find any record of hospitalization, using the name, ID card number and medical record number of the insured Liu Mou, by visiting the medical department and Medical Record Room of the hospital, the medical record and related records were not kept in the hospital. The medical record of the insured person in the Xuanwu Hospital of Beijing affiliated to the Capital University of Medical Sciences was falsified. After verification found that: insured Liu certain medical insurance reimbursement acts suspected of forging medical documents to defraud the medical insurance fund, a total of 21725.16 yuan. According to the Social Insurance Law of the People's Republic of China, the Harbin Municipal Medical Insurance Bureau ordered Liu to return the fraudulent medical insurance fund and transfer the case materials and clue information to the public security organ for processing. In accordance with the criminal law of the People's Republic of China, in August 2021, the People's Court of Shuangcheng District of Harbin city issued a verdict according to law, in which the defendant, surnamed Liu, committed the crime of fraud and was sentenced to one year and six months in prison, suspended for two years, he was also fined 30,000 yuan. So far, 21,725.16 yuan has been recovered from the lost health insurance fund.

1. Xue mou insurance fraud case in Hongkou District, Shanghai

In 2020, the Shanghai Municipal Medical Security Bureau's Office of Supervision and inspection received a tip-off clue that Xue in Hongkou District was suspected of fraud in his practice of repeatedly prescribing drugs with multiple medical insurance cards and seeking medical treatment abnormally, the Shanghai Health Insurance Bureau immediately investigated the tip. After extracting and analyzing health care settlement data and pulling surveillance video, it found that between October and December 2020, xue used someone else's medical insurance card to overprescribe drugs for treating diseases such as hypertension, diabetes, prostate, Parkinson's, and sold them to others for profit. He was suspected of selling drugs to defraud the medical insurance fund, the medical insurance fund was 35,319.29 yuan. Upon enquiry, Xue truthfully told the above illegal facts. In accordance with the social insurance law of the People's Republic of China, the Shanghai Medical Insurance Bureau ordered Xue to return the fraudulent medical insurance fund and transfer the case materials and clues to the public security organs for processing. According to the criminal law of the People's Republic of China, in June 2021, the Jingan District People's Court in Shanghai ruled that the defendant, surnamed Xue, had committed fraud and was sentenced to one year and four months in prison and fined 10,000 yuan. At present, the lost medical insurance fund of 35,319.29 Yuan has been fully recovered.

1. Zhenjiang City, Jiangsu province, Li, Wang fraud case

In 2019, the medical insurance office of Danyang Hospital of Traditional Chinese medicine in Zhenjiang City, Jiangsu Province received a report from an outpatient doctor that some individuals frequently came to the hospital to prescribe drugs. Subsequently, a joint investigation by the Danyang Medical Insurance Bureau and public security organs found that, case officers Li, Wang collect other people's medical insurance card holder drug trafficking, suspected of defrauding the health insurance fund. After investigation, from April 2019 to July 2019, Li used his collected medical insurance card to visit various hospitals in Danyang city frequently, together with other people involved in the case, and will cheat a variety of drugs to the market price of about 50% sold to the co-offender Wang, Wang after the sale of profit, a total of 522,158.81 yuan medical insurance fund fraud. According to the Social Insurance Law of the People's Republic of China, Danyang Medical Insurance Bureau ordered Li and Wang to return the fraudulent medical insurance funds. According to the criminal law of the People's Republic of China, in April 2021, the People's Court of Danyang City ruled that the defendant, surnamed Li, had committed fraud and was sentenced to 10 years in prison with a fine of 90000 yuan The defendant, surnamed Wang, committed fraud and was sentenced to 10 years in prison and fined 90000 yuan. So far, 522,158.81 Yuan has been recovered.

1. The case of Zhang Mou in Ningbo, Zhejiang province

In March 2021, the Medical Security Bureau of Haishu District, Ningbo City, Zhejiang Province received a clue from the Audit Office of Zhejiang Province, which was transferred from the Medical Security Bureau of Ningbo City, regarding an unusual card-swiping case at the Derentang Traditional Chinese medicine clinic in Haishu District, Zhejiang province. After investigation, from September 2018 to September 2020, Zhang took the opportunity to take care of Zhou, Huang and Chen's medical insurance cards, many times in the Haishu District de Rentang Clinic of traditional Chinese medicine using the identity of the above-mentioned people registered, receiving massage treatment, a total of 36,634.92 yuan medical insurance fund fraud. According to the Social Insurance Law of the People's Republic of China, Ningbo Medical Insurance Bureau ordered Zhang to return the fraudulent medical insurance fund. In accordance with the criminal law of the People's Republic of China, in September 2021, the People's Court of Haishu District of Ningbo City handed down a verdict according to law, in which the defendant, Zhang Mou, committed the crime of fraud and was sentenced to a fixed-term imprisonment of one year and two months, suspended for one year and six months, he was also fined 5,000 yuan. So far, 36,634.92 yuan has been recovered from the lost health insurance fund.

1. The insurance fraud case of Xu Mou in Chaohu City, Anhui province

In September 2020, the Chaohu Health Insurance Bureau in Anhui Province received a complaint from a family member of a patient, surnamed Huang, who claimed that the patient had been told by a chronic disease monitoring system set up by the Chaohu Health Insurance Bureau that he had exceeded his limit that month. It was verified that the patient had been prescribed the drug in Soong Ching Ling Charity Hospital under an assumed identity. After investigation, Chaohu City, Xu, together with his son Ding, daughter-in-law Chen embezzled other people's medical insurance card information to open drug sales, suspected of defrauding the Health Insurance Fund 26427.4 yuan. From October 2016 to October 2020, Xu bought medicines from poor and low-income families in Chaohu City. He also contacted drug buyers from other places by telephone to discuss the type, quantity and price of medicines, he then instructed his son, Ding Mou, to sell the drugs he purchased through an online platform, involving a total amount of RMB 2636422.4 yuan. According to the Social Insurance Law of the People's Republic of China, Chaohu City Medical Insurance Bureau ordered Xu to return the fraudulent medical insurance fund. According to the criminal law of the People's Republic of China, on June 22,2021, the People's Court of Chaohu city rendered a judgment according to law, in which the defendant Xu was found guilty of illegal business operations and fraud, he was sentenced to five years and six months' imprisonment with a fine of 110,000 yuan. The defendant, Ding, was sentenced to three years' imprisonment with a suspended sentence of five years, with a fine of 120,000 yuan. The defendant, Chen, was convicted of fraud, he was sentenced to 10 months' imprisonment suspended for one year and fined 5,000 yuan. The defendant Ding returned 100000 Yuan of illegal income, the defendant Chen returned 27000 Yuan of illegal income, all illegal income to the treasury. At present, 26,427.4 Yuan of the lost medical insurance fund has been fully recovered.

1. A fraud case of Jiang Mou in Fuzhou, Fujian Province

In June 2020, the Medical Security Bureau of Fuzhou, Fujian Province, found during a special inspection that Lin, the mother of an urban worker in Fuzhou who was insured by Jiang, died on February 3,2018, her father, surnamed Jiang, died on November 17,2017, however, between December 2017 and December 2018,167 medical insurance settlements were made in medical institutions such as the Shangduan Community Health Service Center in Cangshan District. After investigation, Fuzhou city employees basic medical insurance participants Jiang Mengran in December 2017 to December 2018 period, deliberately hide the fact that mother Lin Menkin and father Jiang Menliang died, as an agent in Cangshan District Community Health Service Center, and other medical institutions counterfeit card settlement 167 times, embezzlement of medical insurance fund 35551.25 Yuan. The investigation also found that in November 2016, Jiang, her son Jiang and her daughter-in-law Wang entrusted“Medical care” to deal with Jiang and Wang's special diseases of hypertension and diabetes with false materials, from April 2017 to March 2021, Ms. Jiang used her and Ms. Wang's medical insurance accounts to buy special drugs for her own use and resell them, defrauding the health insurance fund of 73,680.03 yuan. The above-mentioned illegal acts together defrauded the health insurance fund of 109,231.28 yuan. According to the Social Insurance Law of the People's Republic of China, Fuzhou Medical Insurance Bureau ordered Jiang to return the fraudulent medical insurance fund. In accordance with the criminal law of the People's Republic of China, on 20 July 2021, the Cangshan District People's Court of Fuzhou city rendered a judgment according to law, in which the defendants committed fraud against Jiang, Jiang and Wang, they were sentenced to a term of imprisonment ranging from nine months to three years and three months, and fined accordingly. So far, 109,231.28 yuan of the lost health insurance fund has been recovered.

1. A fraud case of a certain MA in Ji'an city, Jiangxi province

In October 2021, the Medical Security Bureau of Taihe County, Ji'an city, Jiangxi Province, received a tip-off clue that a man named Ma from Baqiu Town, Xiajiang County, Ji'an city, was suspected of impersonating three departments in the Taihe County Hospital of traditional Chinese medicine in July 2021. In October 2021, Taihe County Medical Insurance Bureau after verification and Ma himself check, Ma said impersonation in hospital is no objection. After verification, the medical insurance fund involved 7964.46 yuan. In accordance with the regulations on the supervision and administration of the use of medical security funds and the interim measures of Jiangxi province on reporting and rewarding fraud in obtaining medical security funds, taihe County Medical Insurance Bureau made the following decisions: 1, ordered the return of fraudulent medical insurance funds, and the amount of fraud three times the administrative fine; 2, according to the provisions of the whistle-blower to reward; 3. To transfer the case materials and clue information of a suspected fraudulent medical insurance fund to the public security organ for processing. At present, the loss of the health insurance fund 7964.46 Yuan has been fully recovered, three times the administrative fine 23893.38 Yuan has been all collected.

1. The insurance fraud case of Liu Mou in Zaozhuang city, Shandong province

In July 2020, the Medical Security Bureau of Xuecheng District, Zaozhuang city, Shandong province, received a transfer from the mayor's hotline to an office, reflecting a traffic accident that occurred in 2017 between Liu and others on the southern side of the pedestrian street in Xuecheng District, after negotiations, Liu made up excuses to defraud urban and rural residents from medical insurance and serious illness insurance payments. On August 3,2020, Xue Cheng District Medical Insurance Bureau conducted an on-the-spot interview with Liu, who admitted that he had been injured in a traffic accident on July 13,2017. After that, he falsely claimed that he had fallen and injured his bicycle in a provincial hospital, he was admitted to hospital twice and was reimbursed a total of 52548.02 yuan by medical insurance and serious disease insurance for urban and rural residents in Xuecheng District. According to the“Social Insurance Law of the People's Republic of China”, the Xuecheng District Medical Insurance Bureau ordered Liu to return the fraudulent medical insurance benefits and serious illness insurance benefits for urban and rural residents. In August 2020, Xue Cheng District Medical Insurance Bureau will LIU suspected of fraud to obtain medical insurance fund case materials and clues to the public security organs for processing. On September 15,2020, surnamed Liu voluntarily returned the fraudulent medical insurance and serious disease insurance premiums of 52,548.02 yuan for urban and rural residents.

15. A fraud case of Ding Mou in Xiaogan City, Hubei Province

In December 2019, the Hanchuan Public Security Bureau in Xiaogan City, Hubei province, based on the“Cloud” tip-off tip from the Ministry of Public Security, further investigated and verified with the Hanchuan Medical Security Bureau, and in 2013, Ding Mou and Sun Mou in Hanji Township, Hanchuan City, consulted, provided by Ding his wife Zhang identity information to Sun, Sun responsible for the purchase of false hospital reimbursement materials to ding. Ding in March and May 2014 Zhang in the provincial hospital false hospitalization data fraud medical insurance fund 62930.75 yuan. After the incident, Ding voluntarily turned himself in and confessed to the crime. In accordance with the criminal law of the People's Republic of China, in May 2021, the People's Court of Hanchuan City handed down a verdict according to law, sentencing the defendant, Ding Mou, to three years in prison, suspended for three years, for committing the crime of fraud, he was also fined 20,000 Yuan (the suspect, Sun, was dealt with in a separate case) . At present, 62,930.75 yuan of the lost medical insurance fund has been recovered.

16. The case of Li Mou's insurance fraud in Chenzhou city, Hunan province

In March 2021, a staff member of the Guiyang County Medical Security Bureau in Chenzhou City, Hunan province, found that Li was suspected of providing false bills when he examined the outpatient invoices provided by insured person Li for special outpatient account reporting. Through further verification, Li provided seven anti-rejection drugs from November 2019 to March 2021 special outpatient bills are false bills, involving 66,256.7 yuan health insurance fund. According to the Social Insurance Law of the People's Republic of China, the Guiyang County Medical Insurance Bureau ordered Li to return the fraudulent funds and transfer the case materials and clues related to Li's suspected fraudulent medical insurance funds to the public security organs for processing. In April 2021, Li voluntarily returned 66,256.7 yuan of his fraudulent medical insurance funds. According to the criminal law of the People's Republic of China, in September 2021, the Guiyang County People's court ruled that the defendant, surnamed Li, had committed fraud and was sentenced to one year and ten months' imprisonment and a fine of 20,000 yuan.

1. Insurance fraud cases such as Xiong Mou in Shenzhen, Guangdong Province

In June 2020, the Medical Security Bureau of Shenzhen, Guangdong Province, found that 11 people, including Xiong, were suspected of using their medical insurance cards to cash out and defraud their medical insurance funds. After investigation, starting in March 2019,11 people, including Xiong, in the absence of drug licensing, under the name of“Health insurance card cash”, according to 50% to 55% of the cash back ratio, a large number of foreign social security cards were collected to carry out the act of cash out of medical insurance cards, and thus formed an industrial chain of“Cash in cards + cash in drugs + cash out of resale drugs”. A total of 231 medical insurance cards were charged, with an account amount of 2312,000 yuan, they defrauded the insurance fund of 1,156,000 yuan. According to the Social Insurance Law of the People's Republic of China, the Shenzhen Municipal Medical Insurance Bureau will recover the relevant medical insurance expenses according to the law and regulations. In accordance with the criminal law of the People's Republic of China, on 29 January 2022, the Futian District People's court rendered a judgment according to law, in which 11 persons, including the defendant Xiong Mou, committed the crime of illegal business operations and covered up and concealed the proceeds of crime, he shall be sentenced to a term of imprisonment ranging from one year and two months to six years, and shall also be fined accordingly. The illegal gains of 1156,000 yuan shall be recovered in accordance with the law.

1. Fraud cases of Xie and Xiao in Fengjie County, Chongqing

In March 2019, the Fengjie County Medical Security Bureau of Chongqing City, in an analysis of daily big data, found that hemodialysis patients, such as Xie, Xiao, had abnormal use of their health insurance funds and were suspected of defrauding the health insurance funds. Fengjie County medical insurance bureau in the grasp of relevant information will be the case materials and clues to the public security organs for processing. A joint investigation by the Fengjie County Medical Insurance Bureau and the public security organs found that between January 2018 and March 2019, Xie and Xiao were in charge of collecting and selling drugs, and were responsible for prescribing drugs to people like Chen, Gao, and so on, they were suspected of defrauding the health insurance fund to a total of 224,659.96 yuan. On August 22,2019, Fengjie County public security organs arrested 15 people including Xie, Xiao, Xiang, Gao and Chen according to law. In accordance with the criminal law of the People's Republic of China, between November 20 and December 10,2019, the Fengjie County People's court handed down judgments according to law, in which 15 defendants, including Xie and Xiao, committed the crime of fraud, a term of imprisonment ranging from five months to three years, suspended for one to three years and a corresponding fine. Xie, Xiao and other 15 people in custody during the active surrender of the medical insurance fund 224659.96 yuan in the court after the effective decision has been returned to the medical insurance fund account.

1. A fraud case involving a Liang in Zigong, Sichuan province

In January 2021, an audit of the Basic Medical Insurance Fund in Zigong, Sichuan province, found that Liang, a participant in the Gongjing District of Zigong City, had allegedly falsified medical insurance reimbursement materials between 2017 and 2019. Gongjing District Medical Security Bureau to obtain a Liang in January 2017-december 2019 hospital reimbursement materials, check one by one hospital expense settlement bills, according to the bill, Liang was involved in 33 kidney dialysis treatments in the 11th People's Hospital of Chengdu. The total cost of hospitalization was 610915.14 yuan, the insured person provides the related bill to the Gongjing District Medical Security Service Center to reimburse the related expense, involves the medical insurance fund total 333978.55 yuan. After verification, the actual hospitalization expenses of Liang in the 11th People's Hospital in Chengdu were all settled by the Chengdu Network, and the invoices provided by Liang in the Gongjing district medical insurance center were all false invoices, the medical records and the total list of hospitalization expenses provided are all true. In January 2021, Gongjing District Medical Insurance Bureau transferred the case materials and clue information to the public security organs for processing. According to the criminal law of the People's Republic of China, in September 2021, the Gongjing District People's court ruled that the defendant, surnamed Liang, had committed fraud and was sentenced to six years in prison with a fine of 10,000 yuan, his illegal gains of 333,978.55 yuan were recovered in accordance with the law.

1. An insurance fraud case of a certain Zhao in Qianxinan state, Guizhou province

In November 2018, the medical insurance center of the Social Affairs Bureau of Yilong New District, Qianxinan Prefecture, Guizhou province, found that the medical records of a participant in Yilong new district, surnamed Zhao, were highly similar in their multiple reimbursements, yilong New District Social Affairs Bureau staff to provide reimbursement of their medical records and invoices to verify that their medical records and invoices suspected of fraud. After verification, from 2014 to 2018, the insured person surnamed Zhao repeatedly provided false medical records for reimbursement, involving a total of 99,890.58 yuan health insurance fund. After being informed by the public security organ by telephone, Zhao truthfully confessed his crime, voluntarily pleaded guilty, and signed a guilty plea with a certificate, and actively returned the stolen money. According to the criminal law of the People's Republic of China, on July 16,2020, the Xingyi City People's court issued a judgment according to law, in which the defendant, surnamed Zhao, committed the crime of fraud and was sentenced to three years in prison, suspended for five years, he was also fined 40,000 yuan. So far, 99,890.58 yuan of the lost health insurance fund has been recovered.

1. A fraud case of Zhou in Honghe Prefecture, Yunnan province

In April 2021, the judicial authorities of Honghe Prefecture, Yunnan province, found in their duty to review and prosecute, that a certain Zhou, a participant in Jinping County, Honghe Prefecture, was suspected of defrauding medical insurance funds by making up facts. Jinping County Medical Security Bureau received the judicial transfer of clues, the organization of staff on a Zhou cause of trauma and reimbursement of medical expenses process to verify. Through the joint investigation of the medical insurance system and the judicial organs, it was found that Zhou did reimburse 10375.26 yuan for medical expenses through fictitious injury facts. After investigating the case clearly, Jinping County Medical Insurance Bureau staff to Zhou a home served back to the medical insurance fund notice and explain the medical insurance policy, ordered its reimbursement of medical expenses. According to the criminal law of the People's Republic of China, in April 2021, the People's Court of Jinping County handed down a verdict according to law, in which the defendant, Zhou Mou, was convicted of fraud and sentenced to eight months' imprisonment suspended for one year, with a fine of 2,000 yuan. At present, 10,375.26 yuan of the lost medical insurance fund has been recovered.

1. A fraudulent insurance case was confirmed in Shigatse City, Tibet Autonomous Region

In March 2021, the Medical Security Bureau of Bailang County, Shigatse City, Tibet Autonomous Region, found that residents of Bailang County were suspected of falsifying hospital bills provided by certain insured persons. After checking, certain in 2020 to submit two in 363 hospital reimbursement documents, through verification with the hospital, confirmed that a certain two reimbursement documents are false notes. Bailang County Medical Security Bureau to the party on-site inquiry, the party admitted the fact that its ticket fraud. After verification, a certain in September and November 2020 to provide two false bills, a total of 21,342.61 yuan defrauded health insurance fund. According to the Social Insurance Law of the People's Republic of China, the local medical insurance department ordered a certain person to return the fraudulent medical insurance fund, and imposed an administrative fine of twice the amount defrauded, the case materials and clues were sent to the public security organs for processing. At present, the lost medical insurance fund of 21,342.61 Yuan has been fully recovered, and the administrative fine of 42,685.22 Yuan has been fully recovered.

1. Pingliang City, Gansu province, Li Mou insurance fraud case

In February 2021, the Medical Security Bureau of Jingchuan County, Pingliang City, Gansu Province, received a transfer from Pingliang City's comprehensive convenient service hotline (12345) , reflecting that a villager, surnamed Li, was injured at work, he was suspected of defrauding the medical insurance fund with a false certificate of injury (accident) . After investigation by Jingchuan County Medical Insurance Bureau, it was found that Li's injury had third-party responsibility, which was not included in the scope of basic medical insurance reimbursement, a total of 12,208.4 Yuan was defrauded from the medical insurance fund. According to the“People's Republic of China Social Insurance Law”, Jingchuan County Medical Insurance Bureau ordered Li Mou returned fraudulent medical insurance funds. In accordance with the criminal law of the People's Republic of China, the Jingchuan County People's court ruled that the defendant, Li Mou, had committed fraud and was sentenced to eight months in prison, suspended for one year, and fined 3,000 yuan. So far, 12,208.4 yuan of the lost medical insurance fund has been recovered.

1. Ningxia Hui Autonomous Region in the city of Zhongwei, a fraud case

In May 2019, the Medical Insurance Bureau of Shapotou District, Zhongwei City, Ningxia Hui Autonomous Region, based on clues from the civil judgment of the Intermediate People's Court of Zhongwei City, found that the insured, surnamed Li, was suspected of concealing facts and fabricating injuries, fraudulently obtaining basic medical insurance funds from urban and rural residents. Shapotou District Medical Insurance Bureau organization staff immediately on the involved insured Li Mou launched an investigation to verify. On February 20,2018, a man surnamed Li was admitted to the People's Hospital of Zhongwei city with injuries to his lower back and right knee as he fell down the stairs at home. The total medical expenses during the period of hospitalization were 15,660.8 yuan, on March 6,2018, basic medical insurance funds for urban and rural residents paid 10,375.22 yuan to settle medical expenses. According to the civil judgment of Zhongwei Intermediate People's court, Li was beaten and injured by his husband, he, on February 20,2018. He paid for Li's medical expenses. In June 2020, Shapotou District Medical Insurance Bureau transferred the case materials and clue information to the public security organs for processing. In accordance with the criminal law of the People's Republic of China, the Shapotou District People's court ruled that the defendant, surnamed Li, had committed fraud and was sentenced to eight months' imprisonment suspended for one year and a fine of 2,000 yuan. So far, 10,375.22 yuan of the lost medical insurance fund has been recovered.

Phase II, 2022,10 cases

The state medical security administration has exposed 10 typical cases of illegal and illegal acts by designated retail pharmacies in this period, it involves illegal and illegal activities, such as replacing non-insurance drugs or other commodities with insurance drugs, forging prescriptions or selling non-prescription drugs, selling over-insurance payments for restricted drugs, using the QR code of the electronic health insurance certificate to take screenshots of remote credit cards to buy and sell drugs, settling medical insurance expenses for non-designated retail pharmacies, and changing the location of the medical insurance settlement equipment. The illegal acts of the above-mentioned designated retail pharmacies have violated relevant laws and regulations, caused losses to the medical insurance fund, disrupted the management order of the state medical security, and endangered the vital interests of the people in medical security, they should be dealt with seriously according to law and regulations. The state health insurance administration reminds the management and staff of the designated retail pharmacies that they must strictly abide by the medical security laws, regulations, regulations, relevant policies and the Medical Security Service Agreement of the designated retail pharmacies, to provide insurance drug sales, medical insurance cost settlement and other services in accordance with the provisions to better protect the rights and interests of the vast number of insurance participants, to jointly safeguard the safety of the medical insurance fund.

1. Baicheng Tongchunhe Pharmaceutical Chain Co. , Ltd.

In May 2021, the Baicheng Municipal Public Security Bureau in Jilin province handed over to the Baicheng Municipal Medical Security Bureau clues about the suspected drug exchange of Tongchunhe Medical Chain Co. , Ltd. . After investigation, Tongchun and Pharmaceutical Chain Co. , Ltd. through the exchange of drugs in Baicheng city area actual exchange medical insurance fund 2538544.66 yuan. In accordance with the social insurance law of the People's Republic of China and the 2020 Agreement on designated retail pharmacy service for Baicheng basic medical insurance, the results of the local medical insurance department are as follows: 1. The decision of the penalty was issued to Baicheng Tongchun and Pharmaceutical Chain Co. , Ltd. , refusing to pay 2538544.66 yuan for the illegal use of its medical insurance fund; 2. Rescind the fixed-point service agreement of the pharmacy's medical insurance, and no longer accept the fixed-point application of the medical insurance within three years.

1. Heilongjiang Suihua City Lanxi County Kangtongda Pharmacy Co. , Ltd.

In July 2021, the Medical Security Administration service center of Lanxi County, Suihua City, Heilongjiang Province, conducted a daily inspection of Lanxi County Kangtongda Pharmacy Co. , Ltd. , by comparing the in-store sales system with the health insurance system and visiting patients, it was found that the pharmacy was suspected of illegal use of health insurance funds. After verification, Lanxi County Kang Tong Big Pharmacy Limited Company sold drugs, failed to achieve the authenticity of information, accuracy, integrity, failed to do the accounts match, the accounts match, there were problems such as the retention of Social Security cards for insured persons, which involved the illegal use of 58,715.65 yuan from the medical insurance fund. According to“Lanxi County medical security designated retail pharmacy service agreement”, the results of the local health insurance departments are as follows: 1, refused to pay the illegal use of the pharmacy health insurance fund 58,715.65 yuan; 2. Rescinding the designated Medical Insurance Service Agreement of the pharmacy.

1. Nantong City, Jiangsu province, Beijing Tongren Tang Nanjing Pharmacy Co. , Ltd. . Nantong shop violation case

In February 2021, the Medical Security Bureau of Nantong City, Jiangsu province, found in its daily audit that the Nantong store of Tongrentang, a beijing-based pharmacy company, used a screenshot of the medical insurance electronic certificate QR code to remotely swipe a card to buy and sell drugs, it was found to have illegally used 240,528.2 yuan from the insurance fund. According to the service agreement of Nantong City's basic medical insurance designated retail pharmacy (2021 edition) and the interim measures of Nantong City on management of Medical Insurance Fund and medical service behavior, the results of the local medical insurance department are as follows: 1. Ordering the pharmacy to return the illegal medical insurance fund and deduct 200,000 yuan from the penalty; 2. Rescinding the designated service agreement of the Pharmacy's medical insurance. At present, the lost medical insurance fund of 240,528.2 Yuan has been fully recovered, and the default fine of 200,000 yuan has been fully turned over.

1. In December 2020, a survey conducted by the Hangzhou Medical Security Management Service Center-HangzhouzMedicaliSecurityrInspectiontDetachmentment)

Zhejiangiang province found that betwNovembermber 2018 Augustgust 2019, ke, an employee of Hangzhou Hanghai Road store of Zhejiang Good Mood Big Pharmacy Chain Co. , Ltd. , used his medical insurance card and collected the medical insurance card or wechat Electronic Social Security Card QR code of people like Wang, Ye, Chen, etc. , false settlement of medical insurance expenses, involving illegal use of medical insurance fund 171,088.44 yuan. On the basis of the social insurance law of the People's Republic of China, the Hangzhou Basic Medical Insurance Service Agreement (retail pharmacies) , the provisions on the transfer of suspected criminal cases by administrative law enforcement agencies, and the notice of the Zhejiang Medical Security Bureau and the Zhejiang Public Security Department on the transfer and investigation of fraud cases in the field of medical security, the results of the local medical insurance department are as follows: (1) the illegal use of the pharmacy's medical insurance fund 171,088.44 yuan, and deduct a double penalty of 342,176.88 yuan; (2) rescind the designated service agreement of the Pharmacy's medical insurance, three years no longer accept the fixed-point application for medical insurance; 3, the case clues to the local public security organs for investigation.

1. Fujian Ningde City Fu'an Kangkang Pharmaceutical Co. , Ltd.

In May 2021, the Health Insurance Department of Ningde City, Fujian province, found in an inspection of Fuan Kangkang Pharmaceutical Co. , Ltd. that the drug store's health insurance system sold 170,086 yuan more than the company's drug purchase, sale and deposit account. After investigation, the drug store has the use of empty insurance card to draw medical insurance funds, involving the illegal use of medical insurance funds 170086 Yuan. In accordance with the 2021“Ningde city basic medical insurance designated retail pharmacy service agreement,” the results of the local health insurance department processing as follows: 1, ordered the pharmacy to return the illegal use of the medical insurance fund; 2. Mark the pharmacy 12 points at a time, terminate the health insurance service agreement, and no longer accept the designated application for health insurance for three years. So far, 170,086 yuan has been recovered from the insurance fund.

1. Zhengzhou City, Henan Province, Zhongmou County, new Xiangmin Medical Chain Co. , Ltd. . Wansheng Lu Dian, and other three pharmacies illegal cases

In March 2021, the Medical Security Bureau of Zhongmou County, Zhengzhou City, Henan Province received a tip-off clue, reflect the new to the Civilian Medical Chain Co. , Ltd. . Wansheng Road Shop, Construction Road shop, Xinhua shop there are customers using medical insurance card to buy health products, daily necessities and other violations. Upon investigation, the above-mentioned three pharmacies have staff selling health care products and daily necessities and let the medical insurance patients to use the medical insurance card consumption and other irregularities, involving the illegal use of the medical insurance fund 60,600 yuan. According to the service agreement of appointed retail pharmacies for basic medical insurance in Zhengzhou City, the results of the treatment by the local medical insurance department are as follows: 1. Ordering the above-mentioned three pharmacies to return the illegally used medical insurance funds, deduct the 2021 Quality Guarantee Fee; 2. Order the new Xiangmin Medicine chain company limited to carry out internal rectification and submit a rectification report by a deadline; 3. Suspend the settlement of the above-mentioned three branches of the new Xiangmin Medicine Chain Company Limited; Lifting the fixed-point service agreement for medical insurance. So far, 60,600 yuan of lost health insurance funds have been recovered.

7. The illegal case of Zeng Ling Jiang Pharmacy in Old Town, Tongliang District, Chongqing

In November 2021, the Tongliang District Medical Security Bureau in Chongqing received a tip-off from the public that Zeng Lingjiang Pharmacy, an old town in Tongliang district, Chongqing, was suspected of defrauding medical insurance funds. When the Tongliang District Medical Insurance Bureau of Chongqing conducted an on-site inspection of the pharmacy, it found that, the pharmacy used Tongliang district had made long clinic health insurance POS machine (service terminal) to include the drugs sold in its pharmacy into the Health Insurance Co-ordination Fund for settlement, it confirmed that the pharmacy had fraudulently obtained 62,572.56 yuan from the health insurance fund through fraud and falsification of certification materials. In accordance with the social insurance law of the People's Republic of China, the regulations on the supervision and administration of the use of the medical security fund, and the medical service agreement of the medical security medical institutions of Chongqing Municipality (2020) , the results of the local health insurance authorities are as follows: 1. They ordered Zeng Lingjiang pharmacy to return the illegally used health insurance fund and imposed a double fine; 2. They rescinded Zeng Lingjiang Pharmacy's fixed-point health insurance service agreement, 3. Rescind the medical insurance service agreement of Zeng Linglong Clinic, and transfer the problem of the clinic violation to Tongliang District Health Committee. At present, 62,572.56 yuan of the lost medical insurance fund has been recovered, and a double fine of 125,145.12 yuan has been collected.

8. Illegal cases of renyi-ji pharmacy in Mangkang County, Changdu City, Tibet Autonomous Region

In January 2021, the CHANGDU Medical Security Bureau of the Tibet Autonomous Region further investigated on the basis of the clues handed over by the Medical Security Bureau of the Tibet Autonomous Region, it was found that the renyi-ji pharmacy in Mankang County of Changdu City (the branch of renyi-ji pharmacy in Mankang County of Karuo District of Changdu City) provided credit card service for the adjacent duoyan daily store to obtain medical insurance funds, the illegal use of the health insurance fund was 13,398.8 yuan. According to the Social Insurance Law of the People's Republic of China, the local medical insurance departments dealt with the following results: 1. Ordered the pharmacy to return the illegal use of the medical insurance fund, and imposed double the amount of administrative fines defrauded; 2. To issue a rectification notice to the pharmacy, requiring the pharmacy to report to the Mengkang County Medical Insurance Bureau for the record every inventory entry since February 2021 for inspection. At present, the loss of the medical insurance fund 13398.8 Yuan has been fully recovered, double the administrative fine 26797.6 yuan has been fully recovered.

9.Xi'an Health Medicine Chain Co. , Ltd. . Northwest Sanlu shop illegal cases

In 2020, the Shaanxi Provincial Medical Security Bureau received a tip-off clue, reflecting Xi'an Health Medical Chain Co. , Ltd. . Shaanxi's health insurance bureau handed over the tip to Xi'an's health insurance bureau for further investigation. After verification, the pharmacy has forged prescriptions, illegal sales without prescriptions and other issues, involving illegal use of the health insurance fund 243380 Yuan. According to the Social Insurance Law of the People's Republic of China and the service agreement of the designated retail pharmacy of Xi'an City for medical insurance, the results of the local medical insurance department are as follows: 1. The medical insurance fund illegally used by the pharmacy was recovered, and the 2020 annual service deposit was deducted, 2. Xi'an Lianhu District Medical Insurance Bureau handed over to the police lianhu branch the relevant clues and investigation results of the pharmacy's cash-out by using the medical insurance card. At present, the loss of 243,380 yuan from the medical insurance fund has been fully recovered.

10.The fifth branch of Luzhu Pharmacy Chain Co. , Ltd. . Shihezi city, the 8th Division of Xinjiang Production and Construction Corps

In February 2021, the Shihezi Medical Security Bureau of the 8th Division of the Xinjiang Production and Construction Corps conducted a daily audit of the five branches of the Luzhu Pharmacy Chain Co. , Ltd. . After investigation, Shihezi City Green Pearl Big Pharmacy Chain Co. , Ltd. . Five stores in October 2020 to January 2021 period, there are some problems, such as over-insurance payment, over-selling, non-prescription, non-standard prescription and so on, which involve illegal and illegal use of health insurance fund 2211234.9 yuan. In accordance with the regulations on the supervision and administration of the use of the medical security fund and the supplementary provisions of the special chronic disease dispensary of the basic medical insurance outpatient clinic of Shihezi City of the eighth division, the results of the local medical insurance department are as follows: 1. Ordering the pharmacy to return the illegal medical insurance fund; 2. Rescinding the designated service agreement of the outpatient medical insurance of the pharmacy. At present, the lost health insurance fund of 221,1234.9 Yuan has been fully recovered.

Stage III, 2022,9 cases

Maternity insurance is an important component of social insurance, which covers employers and their employees in accordance with the law, and guarantees that female employees of employers who interrupt their work due to pregnancy and childbirth receive basic economic income, the reimbursement of maternity medical expenses is of great significance to the protection of maternity rights and interests of female workers, the promotion of equal employment between men and women, and the balance of the burden of employers. Individual insured units and individuals participate in maternity insurance by making up and fabricating the information of insured persons; by relying on other means, making up labor relations to participate in maternity insurance; The act of fraudulently obtaining maternity insurance funds, such as falsifying and altering medical records, bills and other related materials to apply for maternity insurance treatment, disturbs the management order of the state medical insurance, and harms the vital interests of the masses of the people, they should be dealt with seriously according to law and regulations. Here, the state medical security bureau has exposed nine typical cases of fraudulent use of maternity insurance funds in this period, in order to remind the vast number of insured units and individuals to participate in maternity insurance through legal channels, provide true, accurate and complete declaration materials to apply for maternity allowance and maternity medical expenses, and jointly maintain the safety of maternity insurance funds.

1. Beijing Chengqi Science and Technology Co. , Ltd. . Beijing Chaoyang District insurance companies fraud case of birth insurance funds

February 2020, according to the relevant procedures, the Chaoyang District Medical Security Bureau received the case file of the Chaoyang District Human Resources and social security bureau on“Beijing Chengqi Technology Co. , Ltd. (hereinafter referred to as 'Chengqi Co. , Ltd. ') forgery and falsification of labor relations in order to obtain birth allowance”. After careful study and consultation with multiple departments, Chaoyang District Health Insurance Bureau in April 2020 in the Chaoyang District Police Station. After investigation and verification by the judicial authorities of the Chaoyang District, between March 2017 and October 2019, Chengqi Company adopted the method of fictitious labor relations, for 36 non-employed women without factual labor relations in the name of Chengqi company employees to pay employee maternity insurance, child-bearing allowance of 987,955.55 yuan. After the incident, Chengqi company will cheat the birth allowance has all returned. In accordance with the criminal law of the People's Republic of China, on 14 July 2021, the People's Court of Chaoyang District, Beijing, sentenced the eight defendants involved in the case to prison terms ranging from nine months to three years and six months for the crime of fraud, they were also fined between 10,000 yuan and 40,000 yuan.

2. The case of fraudulent obtaining of maternity insurance fund by Zhao'an Lamp Fire Network Service Co. , Ltd. of the insured unit of Zhao'an County, Zhangzhou City, Fujian Province

In February 2021, Zhaoan sub-center of Zhangzhou Medical Insurance Center in Fujian Province, Zhaoan Lamp Fire Network Services Co. , Ltd. . Zhaoan sub-center verified through door-to-door audit, inquiry and investigation of relevant personnel of the company, as well as sending letters to the tax department and the Market Supervision Department, the six insured persons, such as Huang and Shen, are affiliated to Zhao'an Lamplight Network Service Co. , Ltd. . They pay the basic medical insurance and maternity insurance, and have no labor relationship with the company. On February 26,2021, the head of the company, surnamed Shen, claimed 35750 Yuan of birth allowance on behalf of surnamed Huang at the health insurance service window of zhaoan sub-center of Zhangzhou Medical Insurance Center. March 25, Shen Shen in a claim on behalf of a child-bearing allowance reimbursement, Zhaoan sub-center found in time, refused to give reimbursement. According to the Social Insurance Law of the People's Republic of China, the results of the local medical insurance departments are as follows: 1. Recovery of the 35,750 yuan maternity allowance already paid; 2. Transfer the case clues to the Zhao'an County Public Security Bureau. According to the criminal law of the People's Republic of China, on September 30,2021, the Zhaoan County People's court sentenced the defendant, Shen Mou, to one year in prison for fraud and a fine of 8,000 yuan. The 35,750 yuan lost from the maternity insurance fund has been recovered.

3. Qingdao City, Shandong province, Qingdao Insurance Unit Zhongjing Yunchuang Enterprise Management Consulting Co. , Ltd. . 13 companies to cheat the case of maternity insurance funds

In August 2021, the Qingdao Medical Insurance Center of Shandong province found that in its work of calculating and distributing birth allowances, qingdao Zhongjing Yunchuang Enterprise Management Consulting Co. , Ltd. and other companies have a large difference between the standard of maternity allowance and the unit's payment base, suspected of using a false labor relationship to cheat maternity allowance, they then reported the case to the Public Security Bureau. A joint task force was set up by the criminal police detachment of Qingdao Public Security Bureau, the Criminal Police Brigade of the development zone public security sub-bureau and Qingdao Medical Insurance Bureau to investigate the case quickly. After investigation, between April 2020 and September 2021, the 17 people involved in the case, including the suspect Wang, successively paid the basic medical insurance and maternity insurance for 302 unemployed women who had no actual labor relations in the names of 13 companies, including Qingdao Zhongjing Yun Enterprise Management Consulting Co. , Ltd. and Qingdao Jingwuzhijing Automobile Service Co. , Ltd. , to defraud them of maternity benefits. At present, the public security departments have arrested 17 suspects involved in the case, the case is being further investigated.

1. Henan Anbang Human Resources Company cheats the maternity insurance fund case

In October 2021, staff of the Yongcheng Medical Security Bureau in Henan province found through daily monitoring, medical data monitoring, big data analysis and verification that, henan Anbang Human Resources Company of Dongcheng District of Yongcheng City falsely declared the labor relations with 13 pregnant and lying-in women without the actual labor relations. In view of the seriousness of the case, suspected fraud insurance, Yongcheng medical insurance bureau on October 19,2021, the case clues to the Yongcheng Public Security Bureau. Yongcheng City Public Security Bureau investigation confirmed that Henan Anbang Human Resources Company exists to cheat the birth insurance fund problem, involving the birth insurance fund 189800 Yuan. In accordance with the criminal law of the People's Republic of China, Yongcheng City People's court sentenced the defendant involved in the case to six months in prison and one year in prison for fraud. At present, the lost 189,800 yuan of the maternity insurance fund has been fully recovered.

5. The birth insurance fund fraud case of Dongguan Sunflower Network Technology Co. , Ltd.

In June 2020, the Medical Security Bureau of Dongguan City in Guangdong province received the case clues handed over by the relevant departments, reflect the existence of Dongguan Sunflower Network Technology Co. , Ltd. did not according to“Guangdong province maternity insurance provisions” will be paid to the full maternity allowance of the situation. After investigation and verification, the company has falsely reported the amount of wages to defraud maternity allowance acts, suspected of defrauding maternity allowance 874500 yuan. The Dongguan Medical Insurance Bureau referred the case to the Public Security Department for further investigation. According to the criminal law of the People's Republic of China, in August 2021, the first People's Court of Dongguan city sentenced the defendant in question, he mou, to 12 years in prison for fraud and a fine of 50,000 yuan, ordered the defendant he mou within 10 days after the judgment came into effect to the unit involved in Dongguan City Medical Security Business Management Center 874500 Yuan. At present, the loss of the maternity insurance fund 874,500 yuan has been fully recovered.

6.Shenzhen Kanglu Selenium Textile Co. , Ltd. of Shenzhen City, Guangdong Province, insurance unit fraud case of maternity insurance fund

In August 2021, the Medical Security Bureau of Shenzhen, Guangdong Province, conducted a big data analysis and found that, shenzhen Kanglu Selenium Textile Co. , Ltd. . 11 employees of the insurance period, payment of wages, maternity allowance, such as the amount of abnormal situation. Further investigation by the Shenzhen Municipal Medical Insurance Bureau found that Shenzhen Kanglu Selenium Textile Co. , Ltd. was suspected to have been involved in the crime between June 2017 and April 2018, between June 2017 and April 2018, the company claimed a total of 540,000 yuan in 11 child-bearing allowances by means of fictitious labor relations. Shenzhen medical insurance bureau in accordance with the“Social Insurance Law of the People's Republic of China,” and other provisions, the case will be handed over to the public security organs for criminal investigation. At present, the lost maternity insurance fund of 540,000 yuan has been fully recovered.

7. Chongqing Shunkun Plastic Products Co. , Ltd. of Chongqing Yuzhong District Insurance Company cheats the birth insurance fund case

At the end of 2019, Chongqing Yuzhong District Medical Security Bureau found that Chongqing Shunkun Plastic Products Co. , Ltd. received abnormal maternity insurance treatment. Through data analysis, it was found that between January 2018 and December 2019, the number of people applying for maternity allowance in the unit had increased abnormally, with some of the workers participating in the insurance scheme paying insufficient contributions or just over six months, some of the workers were immediately suspended after applying for maternity benefits. After the Yuzhong District Medical Insurance Bureau on-site verification of the employment, wage payment, employee income, and related labor contracts, financial statements, accounting vouchers, and other relevant information on payment of medical insurance and maternity insurance premiums, it was found that 25 people in the unit cheated maternity allowance by making up labor relations. Yuzhong District Medical Insurance Bureau transferred the clues of the case to Yuzhong District Public Security Bureau for investigation. After investigation, Chongqing Shunkun Plastic Products Co. , Ltd. . 25 people faked labor relations to cheat maternity allowance 451800 yuan. According to the criminal law of the People's Republic of China, the Yuzhong District People's court sentenced the three defendants involved in the case, he mou, Guo Mou and Zhong mou, to fixed-term imprisonment of three years, three years, two years and six months, for the crime of fraud, and confiscated the illegal gains in accordance with the law, and fined. So far, 451,800 yuan of lost maternity insurance funds have been recovered.

8.The case of Liu Mou, a insured person in Hangzhou, Zhejiang Province, defrauding a maternity insurance fund

In May 2021, the Medical Security Bureau of Hangzhou City, Zhejiang Province, received clues from its subordinate unit, Hangzhou Medical Security Affairs Reception Center, reflect the insurance personnel Liu occurred in the provinces reported reproductive health care costs provided by a hospital in Pizhou City of medical invoices suspected of fraud. After investigation, Liu had an accidental abortion in May 2020, and in order to apply for birth allowance, it found someone through the Internet to forge a hospital in Pizhou medical invoices, discharge records, birth medical certificates and other information, and through their work units to the Hangzhou Medical Security Affairs Acceptance Center for maternity benefits, deception of the birth insurance fund 25233.80 Yuan. According to the“Social Insurance Law of the People's Republic of China”, the results of the local medical insurance department are as follows: 1, recover the birth insurance money defrauded by Liu Mou; 2, impose a fine of twice the amount defrauded by Liu Mou; 3. The clues of the case will be transferred to the public security organs for investigation. At present, the loss of the maternity insurance fund 25233.80 Yuan has been fully recovered, 2 times the fine 50467.60 Yuan has been fully accounted for.

9.Anhui province Hefei Changfeng County insured person Lu Mou cheats to obtain the birth insurance fund case

In June 2021, the Medical Security Bureau of Changfeng County, Hefei City, Anhui province, received a tip-off from the masses that Changfeng County's insured person, surnamed Lu, was suspected of defrauding her to obtain birth allowances. After receiving the tip-off, staff from Changfeng County's medical insurance bureau conducted a serious investigation. After verification, on March 29,2019, Lu made up the fact that he gave birth to a baby girl in a hospital in Anhui province, changfeng County Medical Security Fund Management Center to apply for maternity insurance medical expenses reimbursement, maternity allowance treatment, involving the amount of 23872.50 yuan. According to the“Social Insurance Law of the People's Republic of China” and other provisions, the results of the local medical insurance departments are as follows: 1, recover the birth insurance funds defrauded by Lu Mou; 2. To transfer to the public security organ the clue that Lu was suspected of taking the maternity insurance fund; 3. To reward the informant with 800 yuan. According to the criminal law of the People's Republic of China, on December 30,2021, the Changfeng County People's court sentenced the defendant involved in the case, Lu Mou, to one year in prison, suspended for one year and six months, and fined him 10,000 yuan. The 23,872.50 Yuan lost from the maternity insurance fund has been recovered.

Stage 4,2022,10 cases

The state medical security administration has exposed 10 typical cases of illegal and illegal practices by designated medical institutions in this period, it involves illegal and illegal activities, such as repeated charges, over-standard charges, charge for decomposing items, over-limit settlement of medical insurance payment scope, cross-diagnosis and treatment items, and non-conformity of stock and purchase of medicine consumables. The illegal actions of the above-mentioned medical institutions caused the loss of the medical insurance fund, damaged the image of the designated medical institutions, and had a bad effect on the society. The majority of designated medical institutions should strictly abide by the regulations on the supervision and management of the use of medical security funds, establish and improve the management system of medical insurance, and perfect the relevant systems of post responsibility, risk prevention and control, and accountability, we will resolutely put an end to illegal and illegal activities, constantly improve the people's sense of gain and happiness, and promote the healthy and sustainable development of our medical security system.

1. The case of illegal use of medical insurance fund by Tianjin Anjie Hospital Co. , Ltd.

In April 2022, the Tianjin Medical Security Bureau found in a special inspection on the use of health insurance funds by medical institutions that Tianjin Anjie Hospital Co. , Ltd. was suspected of illegally using health insurance funds. Law enforcement personnel through the Tianjin medical insurance real-time monitoring system to query the hospital's medical insurance settlement information and data analysis, on-site inspection, interview relevant personnel, access to information and other means of investigation and verification, found that the hospital has repeated charges, swap medical treatment items, medical expenses not covered by the medical insurance fund into the medical insurance fund settlement and other illegal acts, illegal use of the health insurance fund 511,596.55 yuan. In accordance with the law of the People's Republic of China on administrative penalties, the regulations on the supervision and administration of the use of medical security funds, and the implementing measures of Tianjin Municipality on the administrative penalty discretion for medical security, the results of the local medical insurance department are as follows: (1) the hospital was ordered to return the medical insurance fund that had been illegally used; (2) the hospital was fined 73,151.72 yuan for illegal acts such as repeatedly charging fees and swapping medical items, the hospital shall impose a fine of 267,757.50 Yuan for the illegal act of including medical expenses that are not covered by the medical insurance fund in the settlement of the medical insurance fund. The total amount of the above two fines shall be 340,909.22 yuan 3. Order the hospital to correct its illegal behavior within a time limit. At present, the lost medical insurance fund of 511,596.55 Yuan has been fully recovered, and the fine of 340,909.22 Yuan has been fully turned over.

2. The case of illegal use of medical insurance fund in Wutaishan Hospital, Yangzhou, Jiangsu province

In October 2021, the Medical Security Bureau of Yangzhou city, Jiangsu province, found that Wutaishan Hospital in Yangzhou, Jiangsu province, was suspected of illegally using health insurance funds during an inspection of illegal fees charged by tertiary medical institutions. After verification, the hospital has repeated charges, over-standard charges, charges for disassembling items, charges without doctor's orders, charges for swapping diagnosis and treatment items, over-payment of medical insurance limits, non-compliance with the sale and storage of drug consumables, and other illegal acts, the illegal use of the health insurance fund involved 2356,896.16 yuan. According to the“Service agreement of the designated medical institution of Yangzhou City”, the results of the local medical insurance department are as follows: 1. Recover the medical insurance fund illegally used by the hospital; 2. Interview the relevant person in charge of the hospital, ordering the hospital to make rectification within a time limit. So far, the hospital has recovered all of the $2,356,896.16 in lost health insurance funds.

3.Cases of illegal use of medical insurance funds by hospitals in Huoshan County, Lu'an City, Anhui province

In June 2021, the Medical Security Bureau of Huoshan County, Lu'an city, Anhui province, found that the Huoshan County Hospital was suspected of illegally using the medical insurance fund during an on-site inspection to deal with illegal activities of designated medical institutions. After verification, the hospital has unreasonable drug use, unreasonable charges, unreasonable examination and treatment, low-standard hospitalization and other medical insurance violations, involving illegal costs 1530344.06 yuan. According to the regulations on the supervision and administration of the use of the medical security fund and the service agreement of the designated medical institutions for the basic medical insurance of Lu'an (2021 edition) , the results of the local medical insurance department are as follows: 1. The non-single disease drug expenses of 122,086.84 yuan were recovered; 2. To recover 1408257.22 yuan of the illegal problems, such as unreasonable drug use, unreasonable examination, unreasonable charge, low standard hospitalization and settlement of the illegal single disease, and deduct the illegal expenses double penalty; 3. Interview the responsible persons of the hospital, order the hospital to strictly implement the responsibility of the main body for the standardized use of the medical insurance fund, and make a comprehensive rectification of the violation and other problems found in the on-site inspection. At present, 1530344.06 Yuan of the lost medical insurance fund has been fully recovered, and 1408257.22 yuan of the default penalty has been fully turned over.

4. The illegal use of medical insurance funds by the People's Hospital of Lichuan County, Fuzhou City, Jiangxi province

In June 2022, the Lichuan County Medical Security Bureau of Fuzhou City, Jiangxi province, carried out daily checks on the use of the Lichuan County People's Hospital Medical Insurance Fund. The law enforcement personnel shall, by means of drawing out the medical records of the patients in the hospital, specifying the expenses, making ward rounds and verifying the charges for laboratory tests, etc. , it was found that there were some problems in the hospital, such as repeated charge, swap charge, high set charge for low-price items and hospitalization in hospital, which involved the illegal use of the medical insurance fund 1100003.29 Yuan. According to the“Social Insurance Law of the People's Republic of China” and“Regulations on the supervision and administration of the use of medical insurance funds”, the results of the local medical insurance departments are as follows: 1. Recover the illegal use of the hospital's medical insurance funds; 2. The court shall be given an administrative penalty of double the amount of the violation; 3. The court shall be ordered to make comprehensive rectification of the problems found. At present, the loss of the medical insurance fund 1100003.29 Yuan has been fully recovered, 1 times the administrative fine 1100003.29 Yuan has been turned over.

5. illegal use of medical insurance funds by the People's Hospital of Nanning City, Guangxi Zhuang Autonomous Region

In October 2021, the Medical Security Bureau of Mashan County, Nanning City, Guangxi Zhuang Autonomous Region, found during an on-site inspection of the county's designated medical insurance institutions that the medical security fund was fully covered, the Mashan County People's Hospital was suspected of illegally using the medical insurance fund. After verification, the hospital has overcharged, cross-item charges, decomposition charges, over-the-limit scope of medical insurance drugs, drug supplies in the sale and storage of non-compliance issues, involving illegal use of the medical insurance fund 1111927.98 yuan. According to“Nanning city basic medical insurance fixed-point medical institutions medical and reproductive services agreement,” the local medical insurance department processing results as follows: 1, recover the hospital illegal use of medical insurance funds; 2. To order the hospital to rectify within a time limit; 3. To notify the hospital of its violation within the scope of designated medical institutions in Mashan County. At present, the lost medical insurance fund of 1111927.98 Yuan has been fully recovered.

6.Cases of illegal use of medical insurance funds by Haikou maternal and Child Health Care Hospital of Hainan province

In May 2021, the Haikou Municipal Medical Security Bureau in Hainan Province, in its daily supervision of the use of medical insurance funds, found that the Haikou municipal maternal and child health care hospital had allegedly illegally used medical insurance funds between June 2020 and April 2021. After verification, the hospital has illegal collection of low-value medical consumables, false charges, over-development of diagnosis and treatment projects, repeated charges, decomposition charges and other problems, involving a total amount of 2178,461.50 yuan. According to the price of medical services in Hainan province (current) and the agreement on medical services for designated medical institutions under the basic medical insurance in Hainan province, the results of the local health insurance department were as follows: 1. They ordered the hospital to return the medical insurance funds that had been illegally used; 2. They interviewed the main leaders of the hospital and ordered the hospital to rectify the relevant problems, and submitted the rectifications within a time limit. At present, the lost medical insurance fund of 217,8461.50 Yuan has been fully recovered.

7. The illegal use of medical insurance fund by Shehong Hospital of traditional Chinese medicine, Suining City, Sichuan province

In June 2021, the Sichuan Medical Security Bureau carried out an inspection on the use of the medical insurance fund of the Shehong Hospital of traditional Chinese medicine in Suining city. After verification, the hospital has a breakdown of the project charges, repeated charges, exchange of medical treatment and other violations of the problem, involving illegal use of medical insurance funds 3762110.71 yuan. According to the“Suining Municipal Medical Security Bureau designated Medical Institutions Service Agreement”, the results of the local medical insurance departments are as follows: 1. Recover the hospital illegal use of the medical insurance fund, deduct 3 times the penalty; 2. Ordering the hospital to make rectification within a time limit. At present, the loss of the medical insurance fund 3762110.71 Yuan has been fully recovered, 3 times the default fine 1128632.13 Yuan has been fully turned over.

8.Cases of illegal use of medical insurance funds by the People's Hospital of Qiandongnan Prefecture, Guizhou province

In December 2021, the Guizhou Medical Security Bureau conducted an inspection on the use of the medical insurance fund of the People's Hospital of Qiandongnan Prefecture and found that, the hospital has the problems of over-diagnosis, false charge, cross-item charge and repeated charge, which involve the illegal use of the medical insurance fund 3408100 Yuan. In accordance with the provisional measures of Guizhou province on the administration of medical security fixed-point medical institutions and the service agreement of Qiandongnan Prefecture fixed-point medical institutions, the results of the local medical insurance department are as follows: 1. Recover the hospital's illegal use of the medical insurance fund, and deduct the corresponding default payment; 2. Interview the head of the hospital and order the hospital to rectify the existing problems within a time limit; 3. Suspend the medical insurance eligibility of two doctors in the hospital for three months. At present, the lost health insurance fund of 3408,100 yuan has been fully recovered, and the default payment of 89,400 yuan has been fully turned over.

9.Illegal use of medical insurance funds by the People's Hospital of Yuanyang County, Honghe Prefecture, Yunnan province

In July 2021, the Medical Security Bureau of Honghe Prefecture, Yunnan Province, conducted an inspection on the use of the medical insurance fund of the People's Hospital of Yuanyang County and found that, the People's Hospital of Yuanyang County has some illegal behaviors, such as exchanging medical fees, over-standard fees, over-diagnosis and treatment in violation of medical norms, over-examination, non-ordered examination, etc. , involving the illegal use of the medical insurance fund 1049205.59 Yuan. According to the“Social Insurance Law of the People's Republic of China” and“Regulations on the supervision and administration of the use of medical insurance funds”, the results of the local medical insurance departments are as follows: 1. Recover the illegal use of the hospital's medical insurance funds; 2. Impose a fine of double the amount of the illegal amount on the hospital; 3. Order the hospital to make rectification within a time limit. At present, 1049,205.59 yuan of the lost medical insurance fund has been returned, and 1049,205.59 yuan of the double fine has been fully accounted for.

10.Case of illegal use of medical insurance fund in Traffic Hospital of Qinghai province

In April 2021, the Audit Office of Qinghai province found in the 2020 Social Security Fund Audit, Qinghai traffic hospital suspected of illegal use of medical insurance funds. The provincial audit office handed the case over to the Qinghai Medical Security Bureau for further investigation. Through communication and coordination with the Audit Office of Qinghai Province, the Qinghai medical insurance bureau verifies relevant data and information, organizes complaints from medical institutions, and confirms that the hospital has violated regulations such as exchanging drug consumables during the year 2020, involving the illegal use of a medical insurance fund of 302,1738.66 yuan. In accordance with the interim measures on the administration of medical security designated medical institutions and the service agreement of Qinghai Medical Security designated medical institutions, the results of the local medical insurance department are as follows: (1) recover the medical insurance fund illegally used by the hospital; (2) interview the relevant person in charge of the hospital and order the hospital to establish and improve the internal control management system for the use of the Hospital Medical Insurance Fund and rectify it within a time limit; 3. To inform the competent department of the hospital and the competent department of the trade of the hospital's violations, and to report the investigation and punishment of its violations to the Department of Discipline Inspection and supervision. At present, the lost medical insurance fund of 302,1738.66 Yuan has been fully recovered.

2023 phase I 10 cases

1. The illegal and illegal use of medical insurance funds by the outpatient department of Fangda Building of Tianjin Medical Group Ma Guang Medical Investment Management Co. , Ltd.

In April 2022, the Tianjin Medical Security Bureau found that the outpatient department of the Fangda building of the Ma Guang Medical Investment Management Company, a Tianjin pharmaceutical group, was suspected of illegally using health insurance funds during its investigation of complaints. Law enforcement personnel through the Tianjin medical security information platform to query the outpatient department medical insurance settlement information data analysis, on-site inspection, interview relevant personnel, access to information and other means of investigation and verification, it found that there were some illegal and illegal acts in the outpatient department, such as exchanging medical treatment items, including medical expenses that are not covered by the medical insurance fund into the settlement of the medical insurance fund, and causing losses to the medical insurance fund, the illegal use of the insurance fund was 605,089.17 yuan. According to the administrative punishment law of the People's Republic of China and the regulations on the supervision and administration of the use of the medical security fund, the local medical insurance departments handled the case as follows: 1. Ordered the outpatient department to return the illegal use of the medical insurance fund; 2. To impose a fine of 579344.97 yuan on the illegal acts of the outpatient department; 3. To order the outpatient department to rectify within a time limit. At present, 605,089.17 yuan of the lost medical insurance fund has been fully recovered, and the fine of 579,344.97 Yuan has been fully turned over.

2.Cases of illegal use of medical insurance funds by Chengde Hospital of traditional Chinese medicine in Hebei province

In June 2022, the Medical Security Bureau of Chengde, Hebei province, conducted a grid check on the use of health insurance funds in designated medical institutions in the city, and found that Chengde traditional Chinese medicine hospitals were suspected of illegally using health insurance funds. After investigation, during the period from January 2020 to December 2021, the hospital committed such illegal acts as medical expenses paid by over-insurance funds being included in the settlement of medical insurance funds, exchange of medical treatment items, repeated examinations, etc. , 511555.40 yuan was involved in the illegal use of the medical insurance fund. According to the“Chengde city medical security fixed-point medical institutions medical service agreement,” the local medical insurance department deal with the results as follows: 1, ordered the hospital to return the illegal use of the medical insurance fund; 2, interview the hospital responsible person, and ordered the hospital to rectify within a time limit. At present, the loss of the health insurance fund 5115555.40 Yuan has been fully recovered.

3. Case of illegal and illegal use of medical insurance fund by New Yuan Hospital in Ejin Horo Banner, Ordos City, Inner Mongolia Autonomous Region

In August 2021, the Medical Security Bureau of Ordos City, Inner Mongolia Autonomous Region, conducted an on-site inspection of the use of health insurance funds by designated medical institutions in the city and found that, new Yuan Hospital in Ejin Horo Banner was suspected of illegally using health insurance funds. After investigation, between January 2020 and September 2021, the new Dollar Hospital in Ejin Horo Banner committed illegal and illegal acts such as overdiagnosis and treatment in violation of diagnostic and treatment norms and over-standard fees, it involved illegal and illegal use of 937,780.72 Yuan (out of which 856,670.92 Yuan has not been allocated to the health insurance fund, and 81,109.80 Yuan has been allocated to the health insurance fund) . In accordance with the regulations on the supervision and administration of the use of the medical security fund and the service agreement of the designated medical institutions for the basic medical insurance of Ordos City, the results of the local medical insurance departments are as follows: (1) refusing to pay 856,670.92 yuan of the medical insurance fund illegally used by the hospital; (2) ordering the hospital to return the medical insurance fund resulting in a loss of 81,109.80 yuan, and caused a loss of 2 times the amount of a fine of 162,219.60 yuan; 3, ordered the hospital deadline for rectification, interviews with the court and the main responsible person; 4. Ordered the hospital to suspend the medical doctor Chi Mou's eligibility to pay for medical insurance services for six months. At present, 81,109.80 yuan of the lost medical insurance fund has been recovered, and a fine of 162,219.60 yuan has been turned over.

4.Heilongjiang province Harbin bin County Fu Ren Hospital Limited company illegal illegal use medical insurance fund case

In 2020, the Harbin Medical Security Bureau in Heilongjiang province received a tip-off that Heilongjiang Furen Hospital Co. , Ltd. (now known as Bin County Furen Hospital Co. , Ltd.) was involved in fraud to obtain medical insurance funds. After investigation, there were 16 illegal and illegal acts in the hospital, such as collecting blood sugar beside the bed and collecting peripheral blood samples, collecting spectrum ECG, etc. , involving illegal use of the medical insurance fund 4684083.92 Yuan (of which, identified as fraud to obtain medical insurance fund 89584.60 Yuan) . According to the“Social Insurance Law of the People's Republic of China” and“Binxian medical insurance fixed-point medical institutions service agreement”, the local medical insurance departments deal with the results as follows: 1. Disqualify the hospital medical insurance fixed-point medical institutions; 2. Order the hospital to return the medical insurance fund that has been illegally used, and impose a five-fold administrative fine of 447923.00 Yuan on the hospital for defrauding the medical insurance fund of 89584.60 Yuan. The bin County Public Security Bureau has taken criminal enforcement measures against the hospital's personnel involved in criminal offences. At present, the lost medical insurance fund of 4,684,083.92 Yuan has been fully recovered, and the administrative fine of 4,479,23.00 Yuan has been fully turned over.

5.Cases of illegal and illegal use of medical insurance funds in Huai'an Hospital, Huai'an City, Jiangsu province

In July 2022, the Jiangsu Provincial Medical Security Bureau carried out a monitoring and inspection of the use of health insurance funds by designated medical institutions in the province and found that Huai'an Hospital in Huai'an City had spent the period from January 2020 to March 2022, there are some illegal problems, such as low-standard admission, no-report fee without doctor's order, over-standard fee, repeated fee and cross-item fee, which involve illegal use of medical insurance fund 3883400.00 Yuan. In accordance with the regulations on the supervision and administration of the use of medical security funds and the measures on the discretion of administrative penalties for regulating the use and administration of medical security funds, and other relevant provisions, in August 2022, the results of the local medical insurance department are as follows: 1. To recover the medical insurance funds illegally used by the hospital, and to impose an administrative fine of double the amount of the illegal funds; 2. To order the hospital to rectify the illegal problems within a time limit; 3. The hospital shall be listed as a general discredit unit of medical insurance; 4. The relevant case clues of the hospital shall be transferred to the local commission for Discipline Inspection. At present, the lost medical insurance fund of 3,883,400.00 Yuan has been fully recovered, and the administrative fine of 3,883,400.00 Yuan has been fully turned over.

6.Cases of illegal and illegal use of medical insurance funds by the Second People's Hospital of Pingyang County, Wenzhou City, Zhejiang Province

In August 2021, the Medical Security Bureau of Wenzhou City, Zhejiang Province, carried out an on-site inspection of the use of the medical insurance fund of the Pingyang County Second People's Hospital and found that the hospital had been in use for the period from 1 January 2020 to 30 June 2021, there are repeated charges, decomposition charges, over-standard charges, over-the-limit scope of medical insurance drug use, will not be included in the settlement of medical insurance fund settlement costs settlement and other illegal acts, resulting in a loss of 2368647.00 Yuan health insurance fund. In accordance with the regulations on the supervision and administration of the use of medical security funds, the measures for regulating the use of medical security funds, the administrative penalty discretion measures, and the service agreement of the designated medical institutions for the basic medical insurance of Pingyang County, the results of the local medical insurance department are as follows: 1, recover the illegal use of the hospital's medical insurance funds, after the implementation of the regulations on the supervision and administration of the use of the medical security fund, the amount of illegal acts involving a fine of 139,571.00 yuan shall be doubled;. At present, the lost 2368,647.00 yuan of the medical insurance fund has been fully recovered, and the administrative fine of 139,571.00 Yuan has been fully turned over.

7. Cases of illegal and illegal use of medical insurance funds by TONGBEI community health service center, Xiangcheng District, Zhangzhou City, Fujian Province

In June 2022, the Fujian Provincial Medical Security Monitoring and electronic settlement center found that the hemophilia special clinic of Tongbei Community Health Service Center in Zhangzhou City was suspected of illegally using health insurance funds. The Zhangzhou Medical Security Bureau immediately organized an inspection team to enter the hospital to carry out an inspection, and found that there were illegal and illegal problems in the hospital, such as duplicating charges, including non-medical insurance expenses in the settlement of medical insurance accounts, and distributing medicines and consumables that did not conform to the accounting standards, the hospital was found to have violated laws and regulations or used the medical insurance fund in violation of a contract, with a total of 2,983,806.77 yuan. In accordance with the regulations on the supervision and administration of the use of medical security funds and the 2021 Zhangzhou Basic Medical Insurance Service Agreement signed by medical institutions, the results of the local medical insurance departments are as follows: 1. deducting the repeated deductions from daily audits, 2. To impose an administrative fine on the hospital for repeatedly collecting fees and including non-medical insurance expenses in the settlement of medical insurance; 3. To order the hospital to make immediate rectification; 4. Discontinue the medical insurance payment eligibility of Doctor Chen for 6 months, discontinue the medical insurance payment eligibility of doctor Guo and pharmacist Lin for 3 months. At present, the loss of the health insurance fund 2815,703.80 Yuan has been fully recovered, administrative fines have been paid in full.

8.Cases of illegal and illegal use of medical insurance funds by the People's Hospital of Leping City, Jingdezhen city, Jiangxi province

In October 2021, the Jingdezhen Municipal Medical Security Bureau of Jiangxi province further investigated and found that, based on the clues handed over by the Jiangxi Provincial Medical Insurance Bureau, there are some illegal behaviors in leping people's hospital, such as lowering the admission standard, over-diagnosis and treatment, breaking down the charge of items, which involve illegal use of the medical insurance fund 5608022.05 Yuan. In accordance with the regulations on the supervision and administration of the use of medical security funds, the detailed rules for the implementation of the power of administrative penalty discretion for the supervision and administration of medical security funds in Jiangxi province (for trial implementation) , and the medical insurance service agreement for designated medical institutions in Jingdezhen municipality, the results of the treatment by the local medical insurance department are as follows: 1. Recover the medical insurance fund illegally used by the hospital, and impose an administrative fine of 1682,000.00 yuan on the hospital for illegal and illegal acts; 2. Interview the relevant person in charge of the hospital and order immediate rectification; Put the hospital on the list of key supervisors. At present, the lost medical insurance fund of 560,8022.05 Yuan has been fully recovered, and the administrative fine of 1,682,000.00 Yuan has been fully turned over.

9.llegal and illegal use of medical insurance funds in Tuen Chang Minfu Hospital, Tuen Chang County, Hainan Province

In November 2021, the Hainan Medical Security Bureau carried out a special inspection on the settlement of the medical insurance fund of the MINFU hospital in Tunchang County from January 1,2020 to September 30,2021. Through big data analysis and on-the-spot verification by medical experts, the inspection team found that there were illegal and irregular problems in the hospital, such as over-standard charges, repeated charges, cross-over charges, low-standard hospitalization, etc. , it found that the hospital had illegally used 1376,110.73 yuan of its health insurance fund. In accordance with the social insurance law of the People's Republic of China, the regulations on the supervision and administration of the use of medical security funds, the rules on the application of the discretionary power of administrative penalties for the supervision and administration of the use of medical security funds of Hainan Province, and the Agreement on medical services for designated medical institutions under the basic medical insurance of Hainan province, the results of the local medical insurance departments were as follows: 1. They ordered the hospital to return the illegally used medical insurance fund, and fined 2064166.10 Yuan (1.5 times the amount of illegal and illegal) ; 2. They ordered the hospital to rectify the situation within a time limit; 3. To warn the hospital and to inform the county of the criticism; 4. To suspend the medical services of the hospital for two months with effect from 1 April 2022 in connection with the medical insurance fund. At present, the lost medical insurance fund of 137,6110.73 Yuan has been fully recovered, and the administrative fine of 20,641,66.10 Yuan has been fully turned over.

10. Case of illegal and illegal use of medical insurance funds by Yan 'ai Hospital of Yushu City, Yushu Prefecture, Qinghai province

In September 2021, the Yushu State Medical Security Bureau of Qinghai province discovered during an on-site inspection of Yushu Renai hospital that, between January 2020 and August 2021, the hospital committed such illegal acts as repeated charges, overpayment of medical insurance limits, over-standard charges, cross-charge charges, hospitalization in beds, overprescribing drug quantities and illegal price increases, the hospital was found to have illegally spent 18,602,43.93 yuan from the medical insurance fund. In accordance with the regulations on the supervision and administration of the use of medical security funds and the service agreement of designated medical institutions for medical security in Qinghai province, the results of the local medical insurance department are as follows: (1) the hospital recovered the illegal use of the medical insurance fund and was fined 277,805.78 yuan; (2) the hospital's medical insurance service agreement was rescinded as of March 30,2022, at the same time, it is prohibited to apply for new eligibility for designated health insurance services within three years, and the whole province is jointly implementing the policy. At present, 186,0243.93 yuan of the lost health insurance fund has been fully recovered, and a fine of 277,805.78 yuan has been fully turned over.

2023 phase II: 10 cases

The state medical security bureau has exposed 10 typical cases of illegal and illegal use of health insurance funds by designated retail pharmacies, it involves illegal acts, such as settlement of medical insurance expenses for non-insurance designated retail pharmacies, replacement of out-of-insurance drugs and health care products with in-insurance drugs for settlement, issuing false drug purchase receipts to pay back the money by swiping the card of the insured persons, and discrepancies in the data on the purchase, sale and storage of drugs. The above-mentioned illegal acts have caused losses to the medical insurance funds and harmed the vital interests of the vast number of insured people. Designated retail pharmacies should strictly abide by the laws, regulations and rules related to health insurance. In accordance with the law, the medical security department should crack down on such illegal activities as fraud and insurance fraud in designated retail pharmacies through daily supervision, on-site inspection, intelligent monitoring and big data analysis, it will increase the efficiency of the use of medical insurance funds and better protect the rights and interests of the vast number of insured people.

1. Case of illegal and illegal use of medical insurance fund by Inner Mongolia Ruichen Pharmaceutical Co. , Ltd.

In August 2021, the Hohhot Medical Security Bureau in Inner Mongolia received a tip-off that the company was suspected of illegal use of health insurance funds. Inspectors retrieved sales records and uploaded data from the health insurance system between January and July 2021, some drugs (such as erding granule, huangqi jing granule, Shiquan dab tonic ointment, Qihu hawthorn granule, etc.) were found to be inconsistent with the purchase and storage, and a total of 23004.50 Yuan was overcharged from the medical insurance fund. According to the regulations on the supervision and administration of the use of the medical insurance fund, the results of the local medical insurance departments are as follows: 1. To recover the illegal use of the medical insurance fund by the pharmacy and impose a fine of twice the amount of illegal violation; 2. Interview the person-in-charge of the pharmacy and order the rectification within a time limit. Return and check the rectification of the pharmacy from time to time to ensure that the rectification is carried out. At present, the lost medical insurance fund of 23,004.50 Yuan has been fully recovered, and the administrative fine of 46,009.00 Yuan has been fully turned over.

2. The case of illegal use of medical insurance fund in Tiedong branch of Jilin province Siping Chengxin Yonghong Pharmacy Chain Co. , Ltd.

In February 2021, the Medical Security Bureau of Siping City, Jilin province, received the“Audit Transfer Letter of the Audit Office of Jilin province on the issue of the withdrawal of medical insurance funds from the Tiedong branch of Siping City Chengxin Yonghong Pharmacy Chain Co. , Ltd..” Siping Municipal Medical Insurance Bureau and Siping Municipal Audit Bureau formed a joint investigation team to carefully investigate and verify the situation reflected in the transfer processing letter. According to the investigation, the pharmacy had some problems, such as the untimely maintenance of the drugs in the medical insurance catalogue and changing the drugs out of the medical insurance catalogue into the drugs in the catalogue, which caused the loss of the medical insurance fund of 147044.32 Yuan. According to the“2020 basic medical insurance designated Pharmacy Service Agreement of Jilin Province,” the results of the local medical insurance department processing are as follows: 1, recover the pharmacy caused by the medical insurance fund losses, refusing to pay all the deposit and deposit of the pharmacy 882343.09 yuan, refusing to pay all the unsettled medical insurance fund 794973.05 yuan; 2, rescinding the pharmacy medical insurance service agreement, and can not apply for medical insurance fixed-point within three years. At present, the loss of 147,044.32 yuan of the health insurance fund has been fully recovered.

3. The case of illegal use of medical insurance fund in goods long store of Nantong Jianghai Pharmacy Chain Co. , Ltd.

In March 2022, the Medical Security Bureau of Haimen district, Nantong city, Jiangsu province, found through remote video surveillance that there was an illegal exchange of medicines at Qianlong store of Nantong Jianghai Pharmacy Chain Co. , Ltd. . After verification, the Qunlong store of Nantong Jianghai Large Pharmacy Chain Co. , Ltd. has committed such acts as taking out cash by using medical insurance cards, using medical insurance cards on behalf of non-designated pharmacies, dispensing medicines with inconsistent witness statements and other violations of medical insurance regulations, the illegal use of the insurance fund was 13,564.58 yuan. According to“Nantong city basic medical insurance fixed-point retail pharmacy service agreement”, the local medical insurance department deal with the results as follows: 1. Recover the illegal use of the pharmacy medical insurance fund 13,564.58 yuan, and deduct breach of contract fine 35,000.00 yuan; 2. Rescind the pharmacy's medical insurance service agreement, and can not apply for medical insurance fixed point within three years. 3. Deduct 40 points from the pharmacy's institutional credit score in 2022. At present, the lost medical insurance fund of 13,564.58 Yuan and the default payment of 35,000.00 yuan have all been recovered.

4. The case of illegal use of medical insurance fund by Shengde pharmacy in Xinyu City, Jiangxi province

In March 2022, the Xinyu Medical Security Bureau of Jiangxi Province, in conjunction with the Market Supervision and Administration Bureau, conducted an on-site inspection of the use of health insurance funds in 30 designated retail pharmacies in the city from January 2020 to November 2021, and found that, shengdeda pharmacy in Xinyu city was suspected of illegally using health insurance funds. After verification, the pharmacy had the following irregularities: using the medical insurance card on behalf of non-medical insurance designated pharmacies; including Chinese herbal pieces that can not be paid for alone in the settlement of the medical insurance fund; the actual sales of some drugs were less than the amount of medical insurance bookkeeping; Changing the medicine and health care products out of the medical insurance catalogue into the medicine in the medical insurance catalogue for settlement;. The above-mentioned actions involve illegal and illegal use of 47,570.40 yuan from the insurance fund. In accordance with the regulations on the supervision and administration of the use of the medical security fund, the implementing rules for the power of administrative discretion in the supervision and administration of the medical security fund of Jiangxi Province (for trial implementation) , and the Xinyu Municipal Medical Insurance Service Agreement for designated retail pharmacies, the results of the local medical insurance departments are as follows: (1) the illegal and illegal use of the medical insurance fund recovered by the pharmacy; (2) the amount involved in defrauding the medical insurance fund by the pharmacy was 22,290.30 Yuan and was fined 4.5 times as much as 100,306.35 yuan, an administrative fine of 45504.18 yuan, totalling 145810.53 yuan, shall be imposed on the amount of 25280.10 Yuan for other general violations, and the fixed-point service agreement of the pharmacy shall be rescinded, it will no longer accept applications for designated health insurance services for three years from the date of publication. So far, 47,570.40 yuan of the lost health insurance fund has been recovered, and an administrative fine of 145,810.53 yuan has been turned in.

5. Case of illegal and illegal use of medical insurance fund in Jishou People's North Road branch of Yifeng Pharmacy Chain Co. , Ltd.

In 2021, the Xiangxi Medical Security Bureau of Hunan province found that the Jishou Renmin North Road branch of Yifeng da pharmacy chain company had an illegal settlement of drug expenses during its daily inspection. Through the data comparison, the inspectors found that the pharmacy had no sales records and no stock of some medicines (such as vinegar yanhusuo, licorice tablets, leucaena flowers, etc.) between January 1,2021 and April 30,2021, however, the actual reimbursement amount in the health insurance system was 76,794.67 yuan. According to the regulations on the supervision and administration of the use of the medical insurance fund, the results of the treatment by the local medical insurance department are as follows: 1. Ordering the pharmacy to return the illegally used medical insurance fund, (2) order the pharmacy to make rectification within a time limit. At present, the loss of 76,794.67 Yuan from the health insurance fund has been fully recovered, and the administrative fine of 76,794.67 Yuan has been fully turned over.

6.Case of illegal and illegal use of medical insurance fund by Chongqing Kaizhou District Pharmaceutical Co. , Ltd.

In December 2021, the Medical Security Bureau of Kaizhou District in Chongqing found in its routine inspection of designated medical institutions that, four designated retail pharmacies, including Kaizhou da pharmacy drug supermarket, Jiulong Road Er Dian, Zhao Jia Dian, Ankang Street, Kaizhou da pharmacy drug supermarket, Kaizhou da Pharmacy Pharmaceutical Co. , Ltd. , were suspected of illegally obtaining medical insurance personal account funds for insured persons. After investigation, the four designated retail pharmacies under the Kaizhou District Pharmaceutical Co. , Ltd. of Chongqing Municipality used the opportunity of their units to purchase epidemic prevention materials for five insured persons to settle accounts using their employees' medical insurance personal accounts, and then issued a tax bill in the name of the unit back to the unit reimbursement, there is the violation of access to employee medical insurance personal account fund, involving employee medical insurance personal account amount of 14869.90 yuan. According to the regulations on the supervision and administration of the use of the medical insurance fund, the results of the treatment by the local medical insurance department are as follows: 1. Ordering the four designated retail pharmacies under the company involved in the case to return the illegally used medical insurance fund; 2, ordered the company to strengthen the health insurance policy propaganda, the inspection of the problems related to self-correction, form a written report; 3. Five participants involved in the case were given warning education because they did not understand the policy of the medical insurance fund. At present, 14,869.90 yuan of the lost health insurance fund has been fully recovered, and the administrative fine of 14,869.90 Yuan has been fully turned over.

7.Cases of illegal use of medical insurance funds in large pharmacies of common people in Beihai City, Guangxi Zhuang Autonomous Region

In February 2021, the Guangxi Zhuang Autonomous Region's Beihai Medical Security Bureau received a tip-off that large pharmacies were suspected of illegally using health insurance funds. Upon investigation, the pharmacy has forged false vouchers in exchange for cash, placing illegal items and other illegal acts, involving illegal use of health insurance fund 71847.05 Yuan. In accordance with the relevant provisions such as the service agreement for designated retail pharmacies of Beihai Municipal Employees' basic medical insurance (single in 2020) , the results of the local medical insurance department are as follows: 1. The full amount of the illegal monthly medical insurance expenses of the pharmacy will be deducted to 71,847.05 yuan, and the illegal situation of the pharmacy will be announced to the public; 2. The fixed-point service agreement of the pharmacy's medical insurance will be rescinded, it will no longer accept applications for designated health insurance within three years. So far, $71,847.05 has been recovered.

8.Cases of illegal use of medical insurance fund in Wuhaotang Pharmacy, Duyun City, Qiannan Prefecture, Guizhou province

In September 2021, the Medical Security Bureau of Duyun City, Qiannan Prefecture, Guizhou province, found that Wuhaotang big pharmacy in Duyun city was suspected of illegally using health insurance funds during routine inspections of designated medical institutions. Qiannan Duyun city health insurance bureau investigators found that the pharmacy there are a number of large medical insurance card settlement anomalies. Through the collection of pharmacy sales system data, interviews with the relevant designated retail pharmacies in charge, telephone interviews with the insured and other means of suspicious clues to investigate and verify. After verification, the pharmacy in Duyun a dental clinic in the Xiong and other 5 medical expenses generated by medical settlement, involving a total of 41880.00 Yuan health insurance fund. According to the service agreement of the designated retail pharmacies of Qiannan Prefecture for medical security and the implementation plan of Duyun City for the evaluation of the credit rating of the medical insurance of the designated medical institutions, the results of the local medical insurance department are as follows: 1. Deduct 41880.00 Yuan from the illegal use of the medical insurance fund by the pharmacy, and pay 2.5 times the penalty for breach of contract. The total amount of the deduction and penalty for breach of contract is 146580.00 yuan, which has all been returned to the medical insurance fund account; 2. From November 2,2021, the medical insurance service agreement of the pharmacy shall be rescinded and its violation shall be disclosed to the public, and it shall be included in the management of the blacklist of medical insurance credit rating; 3. To include the legal representative and the administrator of the pharmacy in the list of those who have broken the promise of the medical insurance; 4. To transfer the cases of the oral clinic concerned to the local health department for handling.

9. The illegal use of medical insurance funds by Hongyan Pharmacy in Shannan City, Tibet Autonomous Region

In April 2022, the Medical Security Bureau of Shannan City, Tibet autonomous region, found that“The same person continuously swiped the card” at Hongyan Dispensary in Shannan City, while checking data on suspected violations. In June 2022, Shannan City Medical Insurance Bureau to Hongyan Pharmacy Illegal Settlement of Medical Insurance Fund conduct a case investigation. After investigation, Hongyan Big Pharmacy has a drug exchange, fictitious medical service projects, will buy local products, health products and other consumer goods for drugs in exchange for medical insurance vouchers settlement fraud and other medical insurance funds, at the same time, there are in-store employees detained medical insurance vouchers, daily fixed card swipe and other illegal acts, involving a total of 102625.00 yuan health insurance funds. According to the Social Insurance Law of the People's Republic of China and the regulations on the supervision and administration of the use of the medical insurance funds, the local medical insurance departments dealt with the following results: 1. Ordered the pharmacy to return the fraudulent medical insurance funds, the penalty is a fine of 205,250.00 yuan (RMB) for double the amount of money defrauded. At present, the loss of the medical insurance fund 102,625.00 Yuan has been fully recovered, 2 times the administrative fine 205,250.00 Yuan has been turned over.

10. The case of illegal and illegal use of medical insurance funds by a branch of Tiancheng pharmacy in Karamay city, Xinjiang Uygur Autonomous Region

In July 2022, the Xinjiang Uygur Autonomous Region Karamay City Medical Security Bureau received a tip-off, reflecting Karamay city tiancheng pharmacy branch suspected of illegal use of health insurance funds. After verification by the Medical Insurance Bureau of Karamay district, Karamay city, the pharmacy has committed illegal acts such as swapping drugs, issuing false drug purchase receipts, and providing insured persons with credit cards to return cash, etc. , the illegal use of the insurance fund was 155,775.69 yuan. According to the regulations on the supervision and administration of the use of the medical insurance fund, the results of the local medical insurance departments are as follows: 1. To recover the illegal use of the medical insurance fund by the drugstore, and to impose a fine of twice the amount of illegal violation; 2. Ordering the pharmacy to make rectification within a time limit. At present, the lost medical insurance fund of 155,775.69 Yuan has been fully recovered, and the administrative fine of 311,551.38 Yuan has been fully turned over.

2023 phase III 10 cases

1. Case of illegal use of medical insurance fund by Chenghui pharmacy in Changchun City, Jilin province

In June 2021, the Kuancheng branch of the Medical Security Bureau of Changchun City, Jilin province, carried out an on-site inspection of Chenghui pharmacy in Changchun City according to the list of on-site inspections of designated medical institutions in Jilin province in 2021, the store manager and the Clerk of the pharmacy were taken records of the purchase of drugs to verify the telephone inquiry. After investigation, the pharmacy provided medical insurance settlement for non-medical insurance fixed-point institutions, with a total settlement amount of 360.70 yuan. It checked the medical insurance upload data with the drug accompanying travel slips of the top 11 drugs in the pharmacy's sales amount, the total amount of each of the 11 accompanying drug bills was less than that of the health insurance upload data, with a cumulative difference of 169,234.52 yuan. In accordance with the regulations on the supervision and administration of the use of medical security funds, the 2021 Changchun Medical Security designated retail pharmacies service agreement, and the 2021 Changchun Medical Security designated retail pharmacies examination rules, the results of the local medical insurance department are as follows: 1. The illegal use of the pharmacy to recover the medical insurance fund, the medical insurance fund losses caused by a fine of 541.05 yuan 1.5 times; 2. Recovering the illegal fee of 169,234.52 yuan that the amount of the medical insurance uploaded by the pharmacy is greater than the amount of the drug accompanying the drug; 3. Rescinding the medical insurance service agreement of the pharmacy; 4. Deducting 15 points from the annual examination score of the pharmacy. At present, the loss of the medical insurance fund 360.70 Yuan and illegal expenses 169234.52 yuan has been fully recovered, the administrative fine 541.05 Yuan has been fully paid.

2.Jiangsu province Suzhou Wuzhong Economic Development Zone Xingsheng Pharmacy Co. , Ltd. illegal use of medical insurance funds case

In July 2022, the Medical Security Bureau of Suzhou City, Jiangsu province, discovered during an on-site inspection of designated medical institutions that, suzhou Wuzhong Economic Development Zone Xingsheng Pharmacy Co. , Ltd. . There are sales non-compliance, incomplete registration of pharmaceutical agents and other irregularities, then the Social Security Center of Wuzhong district through on-the-spot inspection, visits to investigate, ask about the insurance personnel, check bills, etc. , to find out the existence of the following violations of the pharmacy. First, the purchase and storage of non-compliance, spot-check the pharmacy from April 1,2020 to July 27,2022, Gushukang capsules and other five drugs sold and stored, according to the receipts provided by the pharmacies, the previous inventory data and the sales records in the system, it was found that there were irregularities in the data of the import, sale and storage of three drugs, involving a sum of 108,684.90 yuan, during the period from January 2020 to July 2022, the pharmacy did not register or record the drug dispensing according to the regulations when it dispensed drugs to 40 people, involving a sum of 19,948.58 yuan. The total amount of money involved was 128,633.48 yuan. According to the medical service agreement of designated retail pharmacies for Suzhou medical insurance, the inspection and assessment measures for designated medical institutions for Suzhou social basic medical insurance, and the inspection and assessment criteria for designated medical institutions for Suzhou social basic medical insurance, the results of the local medical insurance department are as follows: 1. The amount of the violation will not be paid in 2022; 2.30 points will be deducted from the daily assessment and recorded in the 2022 annual assessment; 3. Rescind the medical insurance service agreement with the pharmacy. At present, the amount of 128,633.48 Yuan has been recovered.

3. The case of illegal and illegal use of medical insurance fund in Huzhou Shuanglin new civilian pharmacy, Shuanglin town, Huzhou City, Zhejiang province

July 2022, Huzhou City, Zhejiang Province, health insurance bureau auditors through data analysis found that Huzhou Shuanglin new large civilian pharmacies exist data anomalies. The auditors found that the card-holder was not the insured person, but there were high-frequency cases of large-sum drug purchase. After verification, the pharmacy has the illegal acts of replacing health care products with medical insurance drugs, assisting others to buy medicines under false names, colluding with others to falsely open expense documents, and settling medical insurance cards with stranded people, etc. , causing a loss of 309,531.93 yuan to the health insurance fund. In accordance with the social insurance law of the People's Republic of China, the regulations on the supervision and administration of the use of the medical security fund, and the Medical Security Service Agreement of the designated retail pharmacies of Huzhou municipality, the results of the local health insurance department are as follows: 1. Recovering the illegal use of the pharmacy insurance funds; 2. Rescinding the pharmacy health insurance service agreement; 3. The pharmacy and the person involved in the case, surnamed Wu, were transferred to the Public Security Department for investigation. At present, the loss of the medical insurance fund 309,531.93 Yuan has been fully recovered.

4.Fujian province Longyan City Huai Ming medicine limited company Liantang Small area branch company illegal use medical insurance fund case

In May 2022, the Xinluo Management Department of Longyan Medical Security Fund Management Center in Longyan, Fujian Province received a notice from the Longyan Medical Security Bureau regarding the submission of clues, the Liantang District branch of Longyan Huaiming Pharmaceutical Co. , Ltd. provides non-medical insurance designated“Huaiming Pharmacy (Rongqiao Yuefu Branch)” with medical insurance card swiping service to settle medical insurance expenses. On May 5,2022, law enforcement officers checked the surveillance video of the Liantang sub-company of Longyan Wyoming Pharmaceutical Co. , Ltd. and found that the pharmacy had not actually purchased drugs from the insured person, payment of medical insurance expenses was made by swiping the medical insurance electronic voucher. On 7 May 2022, law enforcement officers went to the pharmacy to verify the situation on the spot by questioning the shop staff involved, the head of the shop, taking stock of the drug ledger and checking the sales book, it is found that the actual stock of some drugs does not match the registered stock of the system. The information of medical insurance settlement for non-designated retail pharmacies is recorded in the sales book, involving 43551.53 Yuan of medical insurance fund. According to the“2021 Longyan City Basic Medical Security designated retail pharmacy service agreement,” the local medical insurance department deal with the results as follows: 1, refused to pay the pharmacy illegal use of the medical insurance fund 43551.53 yuan; 2. Rescinding the pharmacy's medical insurance service agreement with effect from May 19,2022.

5. The case of illegal and illegal use of medical insurance funds in the general store of Ji'an County of Jiangxi Huang Qing Ren Zhao Hua Shi Da Pharmacy Co. , Ltd.

In December 2021, the Medical Security Bureau of Ji'an County, Ji'an city, Jiangxi province, in accordance with the requirements of Ji'an city for carrying out the key rectification work of irregular cash payments by medical insurance cards, carried out an on-site inspection of Ji'an County general store of Jiangxi Huang Qing Ren Zhao Hua Shi Da Pharmacy Co. , Ltd. . After investigation, the pharmacy there is a medical insurance drugs sales and storage inconsistent with the drug exchange and other illegal acts, resulting in a loss of 29773.90 Yuan Health Insurance Fund. In accordance with the regulations on the supervision and administration of the use of medical security funds and the agreement on drug service for designated retail pharmacies for basic medical security in Ji'an County, the results of the local medical insurance department are as follows: 1. Ordering the pharmacy to return the illegal medical insurance fund and imposing an administrative fine of double the illegal amount; 2. Deducting the performance bond of 1000 yuan. At present, the loss of the medical insurance fund 29773.90 Yuan has been fully recovered, 1 times the administrative fine 29773.90 Yuan has all been turned over.

6.Hunan Rendan Pharmacy Co. , Ltd. . Miluo city, Hunan Province, the public north road branch illegal use of medical insurance fund case

In June 2021, the Medical Security Bureau of Miluo City, Hunan Province, received a complaint from the public about the suspected illegal use of health insurance funds by the public north road branch of the Hunan Rendan Pharmacy Co. , Ltd. . The pharmacy was inspected by law enforcement officers of the Miluo Health Insurance Bureau, found that the pharmacy through Hunan Rendan Pharmacy Co. , Ltd. . Branches and other pharmacies received special disease outpatient (abbreviated as“Special door”)-related information, to assist non-special designated pharmacies to implement special door compensation, the medical insurance fund involved was 105,484.00 yuan. According to the Social Insurance Law of the People's Republic of China and the regulations on the supervision and administration of the use of medical security funds, the results of the local medical insurance department are as follows: 1. Refusing to pay the illegal use of the pharmacy medical insurance fund 105,484.00 yuan, causing the loss of the Medical Insurance Fund 1 times the administrative penalty; 2. Suspend the pharmacy's special service agreement for six months. Currently, the administrative fine of 105,484.00 Yuan has been turned over.

7. The case of unauthorized use of medical insurance fund by Sinan Qinggang Podian of Sinan County, Tongren City, Guizhou province

In January 2022, the Medical Security Bureau of Yinjiang County, Tongren City, Guizhou Province, received an anonymous tip-off that a pharmacy in muhuang town, Yinjiang County, had started the business of settling personal accounts for medical insurance without being eligible for the fixed-point medical insurance. According to the preliminary investigation by the Yinjiang County Medical Insurance Bureau, the clue involves Sinan Tongren Minkang Pharmacy Chain Co. , Ltd. . Sinan Qinggang Podian Yinjiang County of the same chain of pharmacies within the same two non-designated retail pharmacies to provide health insurance settlement. Yinjiang County Medical Insurance Bureau after obtaining relevant evidence will be clues to Sinan County Medical Insurance Bureau (sub-department of Health Insurance) , by the Bureau for further investigation and processing. A further investigation by the Sinan County Health Insurance Bureau found that Sinan Qinggang po shop of the Tongren Minkang da pharmacy chain did not install the required surveillance video and did not require the insured to sign the drug purchase statement to confirm. After verification, the pharmacy for non-designated pharmacies to provide medical insurance settlement, involving 610 person-times, a total of 35400.00 yuan of personal account funds. In accordance with the provisional measures of Guizhou province on the designated administration of retail pharmacies for medical security and the Tongren basic medical insurance designated retail pharmacies (chain pharmacies) service agreement, the results of the local health insurance department are as follows: 1, recover the illegal use of the pharmacy health insurance funds; 2, cancel the pharmacy signed with the Health Insurance Service Agreement. At present, the lost health insurance fund of 35,400.00 Yuan has been fully recovered.

8.Case of illegal and illegal use of medical insurance fund by Shengshi Pharmacy, Yongshan County, Zhaotong City, Yunnan Province

In January 2021, the Medical Security Bureau of Yongshan County, Zhaotong City, Yunnan Province, received a tip-off that Shengshi da pharmacy in Yongshan County was suspected of providing a medical insurance card service on behalf of eyewear shops. Yongshan County Medical Insurance Bureau formed an investigation team, through the collection and collection of documentary evidence, physical evidence, electronic data, questioned the parties, access to the original medical cost statement of the insured, as well as paying for glasses by using the insurance card. After verification, between October 2018 and November 2020, Shengshi da pharmacy in Yongshan County provided medical insurance card service on behalf of eyeglasses shop sales, using false information and passing on fake data, it collected 86,975.00 yuan from the medical insurance personal account fund. In accordance with the social insurance law of the People's Republic of China and the 2020 Zhaotong Medical Insurance Agreement Service Agreement for designated retail pharmacies, the results of the local health insurance department are as follows: 1. Ordering the pharmacy to return the illegal use of the health insurance fund, and a double fine; 2. Rescinding the pharmacy's health insurance service agreement. At present, 86,975.00 yuan of the lost medical insurance fund has been fully recovered, and the administrative fine of 173,950.00 Yuan has been fully turned over.

9. The illegal use of medical insurance funds by the branch of Xizang Medical Co. , Ltd.

In February 2022, the health insurance department of Shigatse, Tibet Autonomous Region, discovered during the year-end assessment of designated medical institutions in the city, xizang Medical Co. , Ltd. . Zade Road store in the detention of a number of health insurance settlement vouchers (Social Security Card) , and cardholders of health insurance settlement records and real-time Monitoring Video Records. Investigation revealed that the pharmacy had left five Medicare vouchers (Social Security cards) in the store between May 2021 and January 2022, and the use of the detention of 5 Social Security card through multiple time-slot payment card to the participants to sell health care products, involving the medical insurance fund 14200.00 Yuan. According to the Social Insurance Law of the People's Republic of China and the regulations on the supervision and administration of the use of the medical insurance funds, the local medical insurance departments handled the case as follows: 1. Recovering the illegal use of the medical insurance funds by the pharmacy, 2. Suspend the medical insurance service agreement of the pharmacy for 6 months. At present, the loss of 14,200.00 Yuan from the medical insurance fund has been fully recovered, and the administrative fine of 28,400.00 Yuan has been fully turned over.

10. The case of illegal and illegal use of medical insurance funds by the 228th chain pharmacy of Tacheng City, Yirentang City, Xinjiang Uygur Autonomous Region

In January 2022, the Tacheng Medical Security Bureau of the Xinjiang Uygur Autonomous Region conducted a special inspection of designated medical institutions and found that the 228th chain pharmacy in the Tacheng City of Yirentang, Xinjiang, was suspected of illegally using health insurance funds. Check personnel through the acquisition of the fixed-point retail pharmacies more than a single settlement of 1000.00 Yuan data, randomly selected part of the cost details to carry out on-site verification. By checking the statement, retail receipts and viewing the surveillance video, found that the drug store exists brush life supplies, health products and other acts. After verification, the drug store exists for the use of medical insurance card for 37 sets brush daily necessities, 19 sets brush health products and other illegal acts. The actual owner of the drugstore Feng Mou and the drugstore manager Wang Mou, and others have admitted the existence of illegal acts, involving the health insurance fund 49129.30 Yuan. According to the regulations on the supervision and administration of the use of the medical insurance fund, the results of the local medical insurance departments are as follows: 1. The illegal use of the medical insurance fund by the drug store is recovered, and a double fine is imposed; 2. Rescinding the pharmacy's medical insurance service agreement. At present, the loss of 49,129.30 Yuan from the health insurance fund has been fully recovered, and the administrative fine of 98,258.60 Yuan has been fully turned over.

2023 phase IV 10 cases

The National Health Insurance Bureau has selected 10 typical cases of illegal use of health insurance funds that were decided by the court to be exposed, it involves such illegal acts as falsifying hospitalization, falsifying medical records, falsifying bills, falsely prescribing medical treatment items, falsely prescribing medical orders, falsely depositing, using medical insurance cards under false names, etc. . The above-mentioned illegal acts caused the loss of the medical insurance fund, disrupted the medical insurance management order, harmed the vital interests of the vast number of insured people, had a bad social impact, and the relevant personnel were severely punished by law. Through case warning, can urge units and individuals to strictly comply with health insurance-related laws and regulations. The medical security department will continue to crack down on such illegal acts as fraud and insurance fraud, strengthen the supervision of medical insurance funds, improve the efficiency of medical insurance funds, and safeguard the safety of medical insurance funds, it will protect the“Medical care money” and“Life-saving money” of insured people.

1. The case of a certain Yuan, a insured person of Danzhai County, Qiandongnan Prefecture, Guizhou province, fraudulently issuing invoices to defraud the medical security fund

In June 2022, when the medical insurance center of Danzhai County, Qiandongnan Prefecture, Guizhou province, examined the sporadic reimbursement data, it was found that there were abnormalities in the monthly reimbursement of outpatient expenses and invoices for chronic special diseases by a certain yuan, by comparing the bill's style, watermark and seal, there were anomalies. After the Danzhai County Medical Insurance Bureau field investigation, Yuan existence of false bills to defraud medical insurance funds, involving the amount of 100459.33 yuan. On August 30,2022, the county medical insurance bureau handed over the clue to the judicial authorities. After verification, Yuan a false bill to cheat the medical insurance fund is true, involving the medical insurance fund 100459.33 Yuan. On 16 May 2023, in accordance with the criminal law of the People's Republic of China, the law of criminal procedure of the People's Republic of China and the answers to certain questions concerning the application of the law in the handling of criminal cases of fraud, danzhai County People's court issued the following verdict: Yuan Mou guilty of fraud, sentenced to three years three months in prison, and a fine of 5000 yuan. At present, the loss of 100,459.33 yuan of the medical insurance fund has been fully recovered.

2.Lidu Hospital of Nanjing City, Jiangsu Province cheats the medical insurance fund case

In September 2021, the Nanjing Municipal Medical Insurance Bureau of Jiangsu Province, after screening and analysis of big data, on-site examination and investigation, found that Lidu hospital in Nanjing was suspected of having committed a crime between April 2020 and August 2021, during the period from April 2020 to August 2021, the hospital cheated the insurance fund by faking the hospitalization of patients and making up medical service programs. The Nanjing Medical Insurance Bureau then promptly sent the information to the public security department. In July 2022, Nanjing medical insurance authorities recovered 5,297,000 yuan from the case-related funds; in March 2023, Nanjing Intermediate People's Court made the following judgment: 1. President Gu was convicted of fraud and sentenced to 12 years' imprisonment and a fine of 10,000 yuan. 2. The financial officer, surnamed Cheng, who committed fraud, was sentenced to three years in prison, suspended for four years, and fined 100,000 yuan.

3. Baoshan district of Shanghai cracked the case of insurance participants who borrowed medical insurance cards to defraud medical insurance funds

In May 2021, the Medical Security Bureau of Baoshan District, Shanghai, conducted an audit of the outpatient and emergency departments and abnormal medical expenses of insured persons, it was found that the medical expenses, the frequency and the track of medical visits of the insured were obviously abnormal, and there were cases of suspected illegal use. Through further data analysis and audit inquiry, to verify the home nanny Lu Xu so-and-so talked about the existence of so-and-so medical insurance card lending to others (Wang) behavior. In accordance with the Shanghai Municipal Measures for the supervision and administration of basic medical insurance and related regulations, the Baoshan District Medical Security Bureau has recovered 117,000 yuan in full from the expenses incurred by Xu and tan in illegally lending their medical insurance cards, they were also fined 5,000 yuan and 1,000 yuan respectively. At the same time, Baoshan District Medical Security Bureau took the initiative to link up the district public security branch for execution, timely transfer of suspicious clues, to cooperate with the public security investigation. In August 2021, after careful investigation, the Baoshan District Public Security Bureau smashed the fraud insurance criminal gang in one fell swoop, and successfully arrested the fraud state medical insurance fund gang headed by Wang and Li, the amount involved was more than 1 million yuan. In February 2023, according to the“Criminal law of the People's Republic of China,” the People's Court of Baoshan District, Shanghai, ruled as follows: Wang committed fraud, sentenced to 11 years and six months in prison, and a fine of 10,000 yuan; Li was convicted of fraud and sentenced to 10 years and 6 months' imprisonment with a fine of 10,000 yuan. So far, all the illegal gains have been handed over.

4.Sichuan province Aba Jiuzhaigou County Wang Mou fraud medical insurance fund case

In 2021, the Medical Security Bureau of Jiuzhaigou County, Aba Prefecture, Sichuan province, investigated and verified the medical expenses of Wang Mou's hospitalization and out-patient clinic, based on the questionable data from the audit department, it was found that Wang had participated in basic medical insurance in both places at the same time. He used the local insurance information to settle the accounts directly at the place where he went to see a doctor and then used forged bills to go to Jiuzhaigou County to apply for medical treatment in another place to defraud the medical insurance fund, he was suspected of committing fraud. The Jiuzhaigou County medical insurance bureau sent the case materials and clues to the public security organs for processing. After verification, since October 2017, Wang has repeatedly forged 40 bills with the same amount of money in the same time period after settlement in the hospital cluster, jiuzhaigou County medical insurance department to apply for reimbursement of medical insurance treatment, a total of 616219.54 yuan medical insurance fund. On 24 September 2022, in accordance with the criminal law of the People's Republic of China, the People's Court of Jiuzhaigou County handed down a verdict according to law: Wang Mou committed the crime of fraud and was sentenced to 10 years and three months' imprisonment and fined 50,000 yuan. At present, the public security organ recovers the medical insurance fund 202608.00 Yuan according to law, and continues to recover the remaining illegal income amount.

5. The medical insurance fund fraud case of Xu Mou, a staff member of the pharmacy of the Fifth People's Hospital in Jiangyin City, Jiangsu province

In October 2021, a joint investigation by the Jiangyin Medical Insurance Bureau and the Public Security Bureau in Wuxi, Jiangsu Province, found that Xu, a former pharmacy staff member of the Fifth People's Hospital in Jiangyin, had repeatedly used other people's medical insurance cards under false names, more than 600 kinds of medicines were purchased in Jiangyin Fifth People's Hospital, Jiangyin Zhutang Community Health Service Center, and Maolong Village Clinic, Zhutang Town, Jiangyin City, a total of 1.82 million yuan was lost from employees' medical insurance funds. Subsequently, the Wuxi Medical Insurance Department recovered more than 1.82 million yuan from the medical insurance funds involved in the case. In July 2022, the local people's court ruled that Xu had committed fraud and was sentenced to three years and three months' imprisonment and a fine of 50,000 yuan.

6.Henan Linying County North Xu Xiaoyang Hospital Fraud Medical Insurance Fund case

In July 2021, a staff member from the Sui County Medical Security Bureau in Shangqiu City, Henan province, was examining the materials of patients seeking medical treatment in different places, it was found that the medical records of some local residents in Luohe Linying County Beixu Xiaoyang hospital were suspected to be falsified. After a joint inspection of the hospital by the Public Security Bureau and the Medical Security Bureau of the two places, it was found that the hospital had induced patients to stay in the hospital by means of free food and lodging and transportation, and that no treatment was given during the period of hospitalization, the illegal act of falsifying patients' medical records to defraud medical insurance funds involved 11 cities, 23 counties and districts of the province, and 65 insured persons, involving a total amount of 1,177,500 yuan. On 27 June 2022, the Sui County People's court sentenced 11 people involved in the case to prison terms ranging from 12 years to one year, and fined them from 50,000 to 500 yuan, depending on the seriousness of the crime.

7.Case of illegal use of medical insurance fund in Tonglin Hospital, Jixi City, Heilongjiang province

In 2021, the Jixi Discipline Inspection Commission of Heilongjiang province found that Jixi Tonglin Hospital (formerly known as Jixi Tongji Hospital) was suspected of defrauding medical insurance funds when investigating a case of violation of law and discipline. Jixi Municipal Commission for Discipline Inspection, Jixi City Public Security Bureau set up Jixi Tonglin Hospital Fraud Insurance Case Special Group to carry out case investigation. In the course of the investigation, the health department organized experts to review the medical records obtained, and the health insurance department organized personnel to verify the reimbursement details that occurred during that period, at the same time, the hospital's questionable drug sales and storage data were checked. Finally, it was found that there were some serious illegal acts, such as induced hospitalization, fictitious medical service, fabricating false medical records, false hospitalization, etc. . According to“Jixi city medical insurance fixed-point medical institutions service agreement, Jixi City Medical Insurance Bureau to lift the hospital medical insurance fixed-point medical institutions qualifications;” The Public Security Bureau of Jixi City collected 17852364.38 Yuan of the medical insurance fund illegally used, returned it to the fund account, and transferred it to the Procuratorate for prosecution. On April 2,2022, Jixi Jiguan District People's Court ruled as follows: Cai Mou committed fraud, sentenced to three years in prison, suspended for five years; At the same time, Cai was prohibited from operating and managing private hospitals during the probation period.

8.The medical insurance fund fraud case of Tianjin Hebei People's Livelihood Clinic, Hebei District, Tianjin

On April 25,2019, an on-site inspection, interview and collection of information by the local health insurance department found that the clinic had violated the law by using its health insurance card to return cash, the amount of money paid by the basic medical insurance fund was 1361030.09 yuan. On May 27,2019, the Tianjin Medical Security Bureau transferred the case to the Hebei branch of the Tianjin Public Security Bureau to investigate its criminal responsibility according to law. On June 10,2021, the first instance judgment of the People's Court of Tianjin Hebei district was as follows: 1. Yin, the legal representative, was convicted of fraud and sentenced to nine years' imprisonment and a fine of 30,000 yuan. 2. The administrator and the person in charge of the medical insurance commit the crime of fraud and are sentenced to five years' imprisonment, two years and six months' imprisonment respectively, and fined one hundred thousand Yuan and five thousand yuan. During the trial, Yin voluntarily withdrew 136,1030.09 yuan from the fund involved. On October 11,2021, the Tianjin No. 2 Intermediate People's court issued a ruling upholding the original sentence.

9. A case of medical insurance fund fraud by false report of drug use in a hospital in Fangchenggang City of Guangxi Zhuang Autonomous Region

In July 2019, the Medical Security Bureau of Fangchenggang City, Guangxi Zhuang Autonomous Region, found in its on-site supervision and inspection that shangsi Chaoyang Hospital was suspected of misreporting drug use and other acts, and on August 6,2019, the medical insurance bureau promptly referred the case to the Public Security Bureau of Fangchenggang for investigation. It was found that between January 22 and July 31,2019, shangsi Chaoyang Hospital overreported drug prices through the health insurance system, totaling 932,825.13 yuan, among them, 483,462.85 Yuan was received from January to May, 2019, and the remaining 449,362.28 yuan was not paid due to the discovery by the health insurance department. According to the“2019 Fangchenggang City fixed-point medical institutions basic medical insurance work injury insurance and maternity insurance service agreement”, the local medical insurance departments handle the results as follows: terminate the fixed-point service agreement of shangsi Chaoyang Hospital, the amount of the violation was recovered was 806,740.44 yuan. On March 30,2021, in accordance with the relevant provisions of the criminal law of the People's Republic of China and the law of criminal procedure of the People's Republic of China, the Shangsi County People's court shall, on the basis of the facts, nature, circumstances and degree of harm to society of the defendant's crime, the sentence was as follows: Ling, Wu, Wei, Zhou, Li and Liu were convicted of fraud and sentenced to prison terms ranging from ten to three years, depending on the circumstances of the crime, and were fined 8 to 20,000 yuan. Ling out of the Dirty Money 463462.85 yuan, Zhou out of dirty money 20000.00 yuan, by the temporary detention of the Shangsi County People's Procuratorate to return the medical insurance fund account.

10. The case of defrauding medical insurance funds by the community health service station of Tongxing Street, Daqing Road, Pingcheng District, Datong City, Shanxi province

In April 2019, the Datong Medical Security Bureau in Shanxi Province received a tip-off clue, reflecting the existence of fraud at the community health service station in Tongxing Street, Daqing Road, Pingcheng District, Datong City. After verification, it was found that the health service station has fictitious medical service items, forged materials, uploading false drug data and other suspected fraudulent insurance acts. According to the Social Insurance Law of the People's Republic of China and the service agreement of the designated medical institutions of Datong City for basic medical insurance, the local medical insurance departments handle the results as follows: 1. Termination of the medical insurance service agreement and rejection of applications from designated medical institutions within three years; 2. The case materials and clues related to his falsification of medical records and other suspected cases of defrauding medical insurance funds shall be transferred to the local public security organs for investigation and handling according to law. After the case was handed over to the public security department, the person involved in the case, Zhu, was detained criminally on suspicion of fraud. After further investigation by the Public Security Department, the relevant evidence was fixed and the above illegal facts were determined, a total of 3,439,893.13 yuan was allegedly defrauded from the medical insurance fund. On October 12,2020, the People's Court of Pingcheng District, Datong City, sentenced Zhu to 15 years' imprisonment for fraud and a fine of 10,000 yuan. At present, the lost medical insurance fund of 343,9893.13 Yuan has been fully recovered.
