# Supplementary material for: Research on quantitative evaluation of medical insurance fraud supervision policy based on ‘Antecedents-Process-Outcomes’ framework
Source: PLoS One. 2025 Jan 6;20(1):e0313618. doi: 10.1371/journal.pone.0313618 (PMC11703012; doi:10.1371/journal.pone.0313618)
Supplement: S2 Appendix — (DOCX) [file pone.0313618.s002.docx]

# Health Insurance Fund fraud regulation policy

# Regulatory policy of medical insurance fund fraud

# November 28,2018

# **Notice on strengthening the management of medical insurance agreements to ensure fund security**

Medical security bureaus of the production and Construction Corps of Xinjiang Autonomous Region, autonomous region and municipality directly under the central government:

The basic medical insurance agency shall, in accordance with the needs of service management, sign a service agreement with a designated medical institution and conduct contractual management, it is the basic management measure and the main handle to standardize the medical service behavior of the fixed-point organization, safeguard the basic rights and interests of the insured, and ensure the safety of the medical insurance fund. Medical security management departments at all levels should fully understand the important role of agreement management, strictly check and strengthen supervision in the process of fixed-point application, agreement implementation, examination of expenses, evaluation and assessment, etc. , they should maintain a high level of pressure on those who violate the agreement to defraud health insurance funds. Here's what we're going to do to manage the current agreement.

First, improve the content of the agreement, improve the withdrawal mechanism

The regional health insurance agencies shall refer to the“Notice of the General Office of the Ministry of Human Resources Security on the issuance of the regulations for the management and administration of the agreements of the designated medical institutions for basic medical insurance”(no. 139) , “Notice of the issuance of the Model Medical Service Agreement for the designated medical institutions for basic medical insurance (version 2016)”(No. 136) , and other documents, further improve and detail the content of the agreement, focusing on the deadline for rectification, suspension of settlement, suspension of agreement, dissolution of the agreement and other treatment measures, clear corresponding violations.

The service agreement shall be rescinded if the designated medical institution defaults in the following ways: 1. To defraud health insurance funds by fabricating medical documents, financial notes or vouchers, or by making up medical services such as“Fake hospitalization and fake medical visits”; 2. Providing settlement of medical expenses for non-designated medical institutions and suspended medical institutions; 3. During the period of validity of the agreement, 3 times have been suspended or the period of suspension of the agreement did not meet the time limit for rectification or rectification is not in place; 4. The medical establishment practice license or business license has been revoked; 5. Refusing, obstructing or not cooperating with the agency in carrying out the necessary supervision and inspection; 6. Other non-compliance acts that cause serious consequences or have a significant impact.

The service agreement shall be cancelled if the designated retail drugstore defaults in the following ways: 1. Forgery of false certificates or collusion with insured persons to exchange cash to defraud funds; 2. Providing fee settlement for non-designated retail pharmacies or other organizations; 3. Reporting Medicare settlements for items that are not covered by the health insurance directory; 4. During the period of validity of the agreement, 3 times have been suspended or the period of suspension of the agreement did not meet the time limit for rectification or rectification is not in place; 5. The drug business license or business license has been revoked. 6. Refusing, obstructing or not cooperating with the agency in carrying out the necessary supervision and inspection; 7. Other non-compliance acts that cause serious consequences or have a significant impact.

Fixed-point medical institutions whose service agreements have been rescinded may not apply for fixed-point medical insurance within 3 years.

2. Strengthen management of agreements and intensify investigation and punishment

(1) the co-ordinating regional health insurance agencies shall, in accordance with the conditions announced by the health insurance administrative departments, accept applications from medical institutions in a timely manner, through expert assessment, Social Security Information System verification, letters to the relevant departments and other forms of advice on the application materials and information of medical institutions to audit. If a medical institution is subject to administrative punishment by the departments of health, drug supervision, Price and market supervision, full consideration should be given in the assessment. The assessment process is subject to social supervision, and the results are made public. The relevant complaints and reports should be carefully investigated and verified.

(2) the regional medical insurance agencies shall establish a standardized two-level auditing mechanism for the initial and reexamination of the expenses declared by the designated medical institutions. We will gradually realize 100 percent coverage of the expenses declared by designated medical institutions through the health insurance information system in the first instance, and the suspected illegal expenses discovered in the first instance shall be verified by consulting medical records and on-the-spot verification. The first trial of the adoption of the cost of a random review of the way, which is not less than 5% of the total cost of hospital checks. The fees that are verified by the examination can be enlarged according to the proportion of the random examination and then refused to be paid.

(3) the regional health insurance agencies shall strengthen supervision and inspection of the implementation of agreements by designated medical institutions, the inspection mode is composed of on-site inspection and off-site inspection, self-inspection and spot-check, manual inspection and intelligent monitoring, combination of prior notification and surprise inspection, complementary, multi-dimensional and full coverage. The focus of supervision and inspection is whether the medical institutions induce the insured persons to stay in hospital, steal and use the social security cards of the insured persons, forge medical documents or bills, assist the insured persons to obtain medical insurance funds, falsely record or make multiple charges for medical services, etc. .

Routine inspection, special inspection and year-end inspection shall be adopted for designated medical institutions. Daily inspection: the frequency and procedure of inspection shall be determined according to the actual situation. In principle, each medical institution shall conduct at least 2 field inspections each year. Special inspection: combined with the centralized problems found in the analysis of big data of medical insurance and the examination of expenses, the agency shall formulate a unified inspection plan, carry out special actions and carry out key inspections no less than 4 times a year in principle. Year-end inspection: combined with year-end liquidation, medical institutions with abnormal indicators are screened through relevant information systems, written verification documents are issued, and relevant materials are submitted by the medical institutions under inspection, and those that are not approved are examined and verified, conduct on-site verification.

The retail pharmacies should adopt the methods of routine inspection and special inspection, and explore the establishment of information-based monitoring methods, such as the system of checking the purchase, sale and storage of drugs, remote video monitoring, etc. , and increase the frequency and scope of surprise inspections for key institutions that may have problems.

(4) designated medical institutions found to be in violation of regulations shall be punished in strict accordance with the agreement. Doctors who are found to have committed such irregularities as defrauding health insurance funds are subject to suspension of medical insurance settlement eligibility for 1-5 years, depending on the severity of the case, and the violation is reported to the health and Health Administration departments. Those who have committed such illegal acts as defrauding medical insurance funds or scalping drugs may be suspended from direct settlement of medical insurance. If a person is suspected of violating laws or administrative regulations, the agency shall request the administrative department to carry out administrative punishment or be transferred by the administrative department to a judicial organ for investigation of criminal responsibility according to law.

3. Strengthen supervisory responsibility and ensure strict accountability in accordance with the law

The health insurance fund supervision departments at all levels should strengthen administrative supervision, conduct medical insurance management, urge management organizations to establish internal control mechanisms, and strictly investigate and punish all kinds of illegal and illegal acts according to laws and regulations. To strengthen supervision and inspection, through the organization of joint review and mutual review, “Double random one open” and other means of random inspection, supervision and coordination of regional agencies to strengthen and standardize the management of agreements. To smooth the channels for reporting complaints, to encourage social supervision, to promote the community to report fraud to the medical security fund acts. To organize and carry out comprehensive supervision, strengthen coordination and cooperation with public security, health, drug supervision and other departments to form a regulatory synergy. Third parties, such as accounting networks and associations, should be actively involved in the supervision to improve the professional quality and working ability of the staff. We will accelerate the development of an integrity system and build a long-term mechanism for the supervision of funds. According to the needs of the work, the State Medical Insurance Bureau every year to determine the key content, the organization of cross-provincial joint review and review and Random Review.

Office of the State Medical Security Bureau

November 28,2018

# November 27,2018

# **Notice on the issuance of the interim measures for reporting and rewarding fraudulent and deceptive acts against the medical insurance fund**

**Medical Insurance Office [2018] No. 22**

Medical insurance bureaus and finance bureaus of provinces, autonomous regions, municipalities directly under the central government and the Xinjiang Production and Construction Corps:

In order to effectively protect the safety of medical security funds, we encourage all sectors of society to report acts of fraud in obtaining medical security funds, and intensify our efforts to combat fraudulent insurance practices, the office of the State Medical Security Bureau and the General Office of the Ministry of Finance have formulated the interim measures on reporting and rewarding fraudulent acts to obtain medical security funds.

Office of the State Medical Security Bureau

Office of the Ministry of Finance

November 27,2018

**Fraud to obtain medical insurance funds**

**Interim measures on rewards for reporting fraud**

Article 1 in order to encourage reporting and severely crack down on fraudulent acts of obtaining medical security funds and ensure the safety of medical security funds, in accordance with laws and regulations such as the Social Insurance Law of the People's Republic of China, these measures are formulated.

Article 2: Citizens, legal persons or other social organizations (hereinafter referred to as informants) shall, in respect of the staff members of medical security agencies, designated medical institutions, designated retail pharmacies and their staff, these measures shall apply to those who report and provide relevant clues and should be rewarded upon verification of the facts.

To encourage regional medical security departments to employ social supervisors to report cases of fraud in obtaining medical security funds.

If the informant is an administrative department of Medical Security, a supervisory and administrative agency, an operating agency or its staff, these measures shall not apply.

The medical security funds mentioned in these measures refer to special funds such as the basic medical insurance for employees, basic medical insurance for urban and rural residents, medical assistance, maternity insurance, and supplementary medical insurance for urban and rural residents, etc. managed by the medical security departments.

Article 3 the regional medical security departments as a whole shall be responsible for the work of reporting and rewarding fraudulent acts involving the local medical security funds as a whole.

Cross-regional reports accepted by higher-level medical security departments shall be investigated and handled by two or more regional medical security departments as a whole, the corresponding co-ordinating regional medical security departments were involved in the co-ordinating regional medical security fund report verification part of the reward.

Article 4 The Acts of defrauding to obtain medical security funds as mentioned in these measures mainly include:

Fraudulent insurance activities involving designated medical institutions and their staff

1. Making up medical services, forging medical documents and bills, and defrauding health care funds;

Providing false invoices to insured persons;

3. Charging medical expenses that should be borne by individuals to the coverage of the medical security fund;

Four. Providing medical treatment to persons not covered by medical insurance;

5. Providing credit card billing services to non-designated medical institutions;

6. Being admitted to hospital under a name;

7. Fraudulently obtaining expenditures from the medical security fund by exchanging medicines, consumables, goods, and medical treatment programs;

8. Other fraudulent insurance practices by designated medical institutions and their staff.

(2) fraudulent insurance activities involving designated retail pharmacies and their staff

1. Stealing the medical insurance identity card, collecting cash for the insured or purchasing non-medical articles such as nutrition and health care products, cosmetics, daily necessities, etc. ;

2. Fraudulently obtaining expenditures from the medical security fund by exchanging medicines, consumables, goods, etc. for insured persons;

3. Providing credit card billing services to non-designated medical institutions;

4. Making false invoices or providing false invoices to insured persons;

5. Designated retail pharmacies and their staff other fraudulent insurance practices.

(3) fraudulent insurance activities involving insured persons

1. Falsifying medical service bills to defraud health care funds;

2. Transferring one's medical security certificate to another for medical treatment or holding another's medical security certificate under false name for medical treatment;

3. Illegally using medical security identity certificates, obtaining medical supplies, etc. , buying and selling illegal profits;

4. Other fraudulent insurance activities involving insurance participants.

(4) fraudulent insurance activities involving staff members of medical insurance agencies

1. Going through medical treatment procedures for persons not covered by medical insurance;

Paying medical insurance expenses in violation of regulations;

3. Other fraudulent insurance activities involving staff members of the operating agencies.

Other acts of defrauding and defrauding the medical security fund

Article 5 the State Medical Insurance Bureau, Provincial (Autonomous Region, municipality directly under the central government) and regional medical security departments shall make public the reporting telephone numbers at the corresponding level to the public. At the same time, expand the website, mail, e-mail, APP and other reporting channels, but also co-ordinate the use of local public service information platform to facilitate whistle-blowers to report.

Article 6 The informant may report the case through any of the reporting channels opened, or through multiple channels simultaneously.

The informant may report directly to the regional medical security department, or to a higher-level medical security department or the State Medical Security Bureau.

Article 7 the informant may report the case in real name or anonymously.

The term“Real-name report” as mentioned in these measures refers to the act of reporting and exposing by providing the informant with a true identity certificate and a true and effective means of contact.

The anonymous report refers to the report behavior that the informant does not provide his true identity. If the informant wants to get the report reward, he can provide other information and effective contact ways to identify his identity, so that the medical security department can confirm his identity after the fact and cash in the report reward.

Article 8 The Medical Security Department shall, within 15 working days after receiving a report, give an opinion on whether to file a case for investigation.

The informant shall, within 15 working days after receiving the report, inform the informant of the inadmissibility and state the reasons for the inadmissibility.

Article 9 The Medical Security Department shall complete the handling of the reported cases within 30 working days from the date of acceptance. If the case is complicated, it may be concluded within three months after approval by the person in charge of the unit. A particularly serious case may be appropriately prolonged after collective study by the unit, but in principle not exceeding 6 months.

Article 10 a reward shall be given to those who meet the following conditions at the same time:

(1) the report is verified to be true, causing losses to the medical security fund or avoiding losses to the medical security fund due to the report;

(2) the main facts and evidence provided by the informant are not in the possession of the administrative department of Medical Security in advance;

(3) the informant chooses to be rewarded for reporting.

Article 11 if the informant is an internal staff member of a designated medical institution, designated retail pharmacy or a former internal staff member, the reward standard may be appropriately raised.

If the informant is a designated medical institution, a designated retail pharmacy competition institution and its staff, and provides reliable clues, the reward standard may be appropriately raised.

Article 12. As a whole, regional medical security departments shall set up incentive funds for reporting cases, which shall be incorporated into the budgets of governments at the same level.

Article 13. Rewards for reporting shall be combined with moral and material rewards.

As a whole, the regional medical security departments may, in accordance with a certain proportion of the amount of the fraud insurance that has been verified, reward qualified informants with a maximum amount not exceeding 100,000 yuan as the reward fund for reporting fraud, in principle, non-cash payments should be used.

Fraud insurance does not involve the value of the goods or the amount of fines confiscated, but if the content of the report is true, financial incentives may be given depending on the circumstances.

Article 14 if two or more informants report the same facts, the first informant shall be the target of reward according to the reporting time; if the informant jointly reports, the informant shall be rewarded according to the reward amount of one informant, the rewards shall be distributed by the informants through consultation.

Article 15 the regional medical security departments as a whole shall open up convenient payment channels to facilitate informants to receive reporting bonuses.

Article 16. When making overall plans for the payment of reporting bonuses by regional medical security departments, they shall strictly examine and verify such payments to prevent fraudulent claims.

Article 17 medical security departments at all levels shall protect the legitimate rights and interests of informants in accordance with the law and shall not divulge information related to informants. Those who harm the interests of the informant by divulging relevant information shall be dealt with in accordance with relevant provisions.

Article 18 false reports shall be strictly prohibited. The informant intentionally fabricates facts to falsely accuse others, or fraudulently obtains rewards, and shall bear corresponding responsibilities according to law.

Article 19 the provincial and regional departments of medical security and finance may, in accordance with these measures, formulate detailed rules for implementation, and make specific provisions on the decision, standards, examination and approval, and issuance procedures of rewards.

Article 20 these measures shall be interpreted by the State Medical Security Bureau and the Ministry of Finance and shall be implemented as of the date of issuance.

Note: these measures were issued on November 29,2018.

# February 20,2019

# **Notice on the regulation of medical insurance funds for 2019**

Medicare [2019] No. 14

Medical security bureaus of the production and Construction Corps of Xinjiang Autonomous Region, autonomous region and municipality directly under the central government:

In order to implement the decisions and plans of the CPC Central Committee and the State Council, strengthen the supervision of the medical security fund, resolutely crack down on fraudulent insurance activities, and ensure the safety of the fund, the following is the circular on the supervision of the Medical Security Fund for 2019:

1. Intensify the strike to consolidate the high-pressure situation

(1) the supervision and inspection shall cover all areas. We will improve work mechanisms and consolidate the high-pressure situation in fund supervision. All the regions should integrate their resources, concentrate their special forces and innovate their working methods, and investigate one by one the non-compliance and illegal acts of the designated medical institutions within their jurisdiction, so as to achieve full coverage of on-site inspections by the designated medical institutions. We should comprehensively use intelligent monitoring, surprise inspection, expert review and other means to bring all medical security fund payments into the scope of post-review, and speed up the interception and advance warning to extend. Provincial health insurance departments should strengthen overall planning, coordination and supervision and inspection, timely coordination of regional designated medical institutions for spot-check, the proportion of spot-check not less than 10% .

(2) carrying out special governance. On the basis of comprehensive inspections, we will launch a dedicated administration to combat fraud and insurance fraud in 2019. On the basis of the 2018 special action against fraud and insurance, provinces should, in light of local realities and in light of weak links, identify 1-2 special governance priorities and concentrate their efforts on cracking down severely. Before the end of March, we will study and formulate a unified special governance work plan for the whole province and report it to the State Medical Security Bureau for the record, provincial health insurance departments to carry out random checks, and by the end of November to the State Medical Security Bureau to submit a summary of special governance.

(3) conducting flight inspections. The State Medical Security Administration will establish a working mechanism for flight inspections, gradually improve the flight inspection workflow and operational norms, and occasionally supervise and guide local work through flight inspections. After receiving the notice of flight inspection, the provincial medical insurance departments should be strict in their work discipline, actively cooperate with the inspection, and complete the follow-up investigation and punishment of the flight inspection as required.

(4) giving prominence to the key areas of attack. In view of the characteristics of frequent and high-frequency violations of different supervision objects, focusing on key points, classified strike, corresponding measures. For designated medical institutions, the focus of supervision should be further determined according to their service characteristics, and public medical institutions at or above the secondary level, focusing on the investigation and punishment of disassembling charges, over-standard charges, repeated charges, fees for applying items, unreasonable diagnosis and treatment, and other illegal and illegal acts; basic-level medical institutions, we will focus on investigating and dealing with behaviors such as hospitalization by hanging beds, drug exchange, consumables, and diagnostic and therapeutic items. We will also focus on investigating and dealing with social medical institutions, and inducing insured persons to be hospitalized, making up medical services, forging medical documents and bills, lying in bed in hospital, and stealing social security cards will be investigated and dealt with. Targeted at designated retail pharmacies, focused on the investigation and punishment of convergence, Stolen Social Security card, to induce insurance personnel to buy cosmetics, daily necessities and other acts. For the insured, we will focus on investigating and punishing such acts as forging false bills for reimbursement, seeking medical treatment under false names, using social security cards for cash or medicine, and buying and selling consumables. For medical insurance agencies (including commercial insurance agencies that undertake basic medical insurance and serious disease insurance) , supervision and inspection should be strengthened, we will focus on investigating and punishing such acts as incomplete internal audit system, incomplete fund audit, incomplete performance check, illegal medical treatment, illegal payment of medical insurance expenses, and internal personnel's“Inside and outside collusion”.

2. Improve the reporting system and standardize the investigation and handling of clues

(1) implementation of reporting incentives. All localities shall, in accordance with the requirements of the“Interim measures for reporting and rewarding fraud in obtaining medical security funds”(issued by the Medical Insurance Office [2018] No. 22) , and in conjunction with relevant departments, formulate specific implementation rules for reporting and rewarding. Clear implementation of the specific standards of incentives, application, approval, issuance process and other related content, to ensure that the masses easy to understand, easy to operate, easy to cash. The legitimate rights and interests of informants shall be protected in accordance with the law, and relevant information concerning informants shall not be divulged.

(2) to standardize the process of handling reports. To formulate specific measures to standardize the working process and mechanism of the receipt, assignment, investigation and punishment of the informants' clues, to clarify the standards of the reception and punishment, and to provide timely feedback to the informants on the reception and punishment of the clues, to continuously improve the satisfaction of informants with the handling of reports. The medical insurance departments at all levels should set up the account one by one for the valuable clues, check them carefully, screen the truth and falsehood, complete the settlement within a time limit and report according to the requirements. The Provincial Medical Security Department is the first person responsible for the investigation and punishment of the clues handed over by the State Medical Security Bureau. We should strengthen the supervision and guidance of the clues for the overall planning of regional investigation and handling, TAMP down the responsibility of investigation and handling, and intensify the random check and review.

3. Promote intelligent monitoring and improve the effectiveness of monitoring

(1) carrying out intelligent monitoring and control in an all-round way. Provincial health insurance departments should comprehensively comb the construction of intelligent monitoring information systems within their jurisdictions, and strengthen supervision and guidance, according to the national unified technical standards, operational standards, operational standards and national medical security information platform construction needs, the construction of regional medical security intelligent monitoring information system, strive to achieve the pilot areas of information construction before the end of 2019 to achieve the online trial operation of the medical security intelligent monitoring system.

(2) improving the quality and efficiency of intelligent monitoring. To improve the quality and efficiency of intelligent monitoring, we should constantly sum up the experience and lessons, according to the change of the characteristics of fraud insurance, improve the monitoring rules, refine the monitoring indexes and the knowledge base of intelligent monitoring. We will actively promote Internet + video surveillance and steadily advance the installation of video probes in key areas such as the main entrances and billing windows of some pharmaceutical institutions, to achieve real-time comparison of medical data and service images, synchronous on-line monitoring, better collection and locking evidence of violations of laws and regulations to enhance the effectiveness of monitoring. We will explore new technologies, such as facial recognition, to move the regulatory threshold forward.

(3) construction of smart monitoring demonstration sites. In 2019, the State Medical Security Administration will carry out the construction of smart monitoring demonstration sites, selecting areas with high motivation and a good information foundation to carry out the construction of smart monitoring demonstration sites, and giving corresponding support to the demonstration sites. All localities should also take this as their main task, give full play to the leading effect of demonstration sites, and push for new breakthroughs in the national intelligent monitoring work.

(4) ensuring information security. Intelligent monitoring involves data mining, collection and use, and cooperation with third parties such as information technology organizations and commercial insurance organizations. In the process of cooperation, confidentiality agreements should be signed in accordance with laws and regulations, and the duty of confidentiality should be clearly defined, strengthening Authority management. All areas should carry out information security physical examination in an all-round way, plug data risk loopholes, and ensure the information security of insured people.

4. Improve the supervision system and enhance the ability of administrative supervision

1. Promoting the development of an administrative supervision system. Various localities should establish and improve a regulatory system for administrative law enforcement in conjunction with institutional reform. The division of powers and responsibilities for good deeds should be made, and the scope of powers and responsibilities for administrative supervision at the provincial, city and county levels should be clearly defined. We should straighten out the relationship between administrative supervision and management by agreement, and promote the relative independence and complementarity of administrative supervision and management to form a joint force. We should strengthen the administrative supervision team and ensure the necessary administrative law enforcement forces and means. We will standardize procedures for law enforcement and case handling in medical security, and improve systems for publicizing information on administrative law enforcement in medical security, recording the entire process, rules for legal review, and rules for collective deliberation, so as to improve the effectiveness of investigating and handling illegal cases.
2. We will carry out pilot projects to innovate regulatory approaches. Third parties, such as information technology service agencies, accounting networks and associations and commercial insurance institutions, will be actively introduced to participate in the supervision of the fund. In 2019, the National Medical Security Bureau will select a number of regions with high motivation and a certain working basis to carry out the pilot of innovative regulatory approaches.

(3) conducting business training in an all-round way. To formulate a fund supervision and training plan in a coordinated manner, and to conduct a round of training for fund supervision teams in 2019, focusing on training in fund supervision laws and regulations, typical cases of violations of laws and regulations, Ways and means of investigation and punishment, and so on, teaching by case, and quickly improving the business capabilities of the supervision team. The State Medical Security Administration is responsible for the training of provincial regulatory teams, and provincial medical insurance departments are responsible for the training of provincial regulatory cadres.

5. Standardize the audit and strengthen the agreement management

(1) to standardize the contents of the agreement. Strictly implement the requirements of the“Notice on strengthening the management of medical insurance agreements to ensure fund safety”-No.-no. 21, issued by MedicaliInsuranceaOfficefice (2018)) , detailing the contents of the agreements, clarify violations and measures to deal with them. Each place should aim at the different type, the different nature fixed-point medicine organization, refines the service agreement clause, enhances the agreement management the pertinence and the validity.

(2) strengthening the administration of agreements. On-site inspection and off-site inspection, manual inspection and Intelligent Monitoring, prior notification and surprise inspection are combined to carry out all-round inspection and audit of the implementation of agreements by designated medical institutions. Strict cost audit, standard first audit, review two-level audit mechanism, through intelligent monitoring and other means, to achieve 100% of the first audit of medical expenses. The rate of random and spot-check was no less than 5% .

(3) improving the internal control mechanism. We will strengthen the internal control system of the fund management organizations, standardize the fund accounting and financial systems, and resolutely plug risk loopholes. Each provincial-level medical insurance department shall formulate an inspection plan to comprehensively carry out internal control inspection on the operating agencies within its jurisdiction, focusing on whether the internal management of the operating agencies is standardized, whether the various systems are sound, and whether the duties of the posts are crossed, whether the non-compliant designated medical institutions are dealt with in accordance with the agreements.

6. Promoting comprehensive supervision and regulation to promote inter-departmental interaction

(1) establishing a working mechanism. All localities should strengthen coordination and communication, actively seek the support of departments such as health, public security, market supervision, auditing, finance, Discipline Inspection and supervision, and establish and improve comprehensive supervision and coordination mechanisms, we will coordinate and guide the supervision of the Medical Security Fund and the investigation and handling of major cases.

(2) to form synergy of supervision. We will strengthen information exchange and put in place a system of multiple investigations into a single case and multiple investigations into multiple cases. For suspected cases of violation of laws and regulations, we should actively consult with the departments of Public Security, health, market supervision, Discipline Inspection and supervision to investigate and deal with them. We should promptly notify the relevant departments within the jurisdiction of the clues of violations and laws that have been found, disqualified from practicing law or investigated for party discipline. Those suspected of committing crimes shall be promptly transferred to judicial organs.

7. Promote the construction of credit system and promote self-discipline of the industry

1. Piloting the building of a integrity system for fund supervision and regulation. We will carry out trials to develop a integrity system for the regulation of medical security funds in different types of regions. Focus on the integrity system to build the relevant standards, norms and indicators system, the collection of relevant information, scoring and use of content. Through pilot projects, we will explore ways to build a health insurance regulatory integrity system, laying the foundation for its popularization.
2. To explore the establishment of a“Blacklist” system for medical insurance. The establishment of“Blacklist” system for medical institutions, medical insurance doctors and insured persons in serious violation of regulations will be explored. • Explore Ways and means to improve the disclosure of blacklists to the public. We will actively promote the incorporation of medical insurance fraud into the national credit management system, establish a disciplinary system for dishonesty, and bring into play the joint deterrent effect of punishment.

(3) promoting industry self-discipline. To encourage and promote the development of industry norms and self-regulation in public medical institutions, non-public medical institutions, retail pharmacies, physicians and other trade associations, and the development of self-regulation conventions to promote industry self-regulation and self-regulation, to participate in the building of an integrity system and enhance the integrity of the industry.

8.Strengthen the legal system and improve the system and mechanism

(1)Accelerate the development of the legal system for the supervision of funds. We will continue to make laws in a scientific manner and promote law-based administration. We will systematically comb the relevant laws and regulations on the supervision of medical security funds, accelerate the legal construction of the supervision of funds, and upgrade the mature, effective and feasible fund supervision measures into corresponding laws and regulations through the legislative process.

(2) we will coordinate and push forward the reform of health insurance. We will push ahead with major reforms such as the reform of the management of the list of benefits, the reform of the health insurance payment system, and the reform of individual accounts, optimize the regulatory environment for funds, and guide designated medical institutions in actively standardizing their medical care services, • Preventing insured people from skimming funds from their personal accounts.

9. Step up publicity efforts and strengthen the guidance of public opinion

(1) launch a monthly campaign to combat fraud and insurance. April 2019 will be the national anti-fraud insurance month. Various localities should take various measures, focus on publicizing and interpreting laws, regulations and policies on the supervision of medical security funds, strengthen the legal awareness of designated medical institutions and insured personnel, and consciously safeguard the safety of medical security funds. We will uniformly print and widely post anti-fraud insurance posters, distribute promotional leaflets and broadcast uniformly produced anime promotional videos, and use popular forms of publicity to strengthen the guidance of public opinion and positive publicity.

(2) to establish a system for reporting the facts of a case. For major cases involving a sum of over 500,000 yuan which have been investigated and dealt with, or which may be transferred to public security organs, or which may cause public concern, all localities shall, within one week after the case is concluded or handed over to the public security organs, report the situation to the state medical security bureau. The report should include the process of discovery, the fact of violation, the result and the basis of treatment.

(3) exposure of typical cases. All places should take the initiative to expose the typical cases of fraud and insurance fraud that have been found, so as to create a deterrent effect. Through press conferences, media briefings and other forms, the release of anti-fraud insurance results and typical cases. Actively invite the news media to participate in flight inspection, open investigation and secret visits and other activities, guide the media to form a benign interaction.

10. Strengthen organizational leadership and improve the incentive and accountability mechanism

All localities should fully understand the importance, urgency and arduous nature of the fund supervision work, regard the maintenance of fund safety as the primary task of current medical security work, strictly enforce political discipline, and strengthen responsibility, work creatively to ensure the successful completion of all tasks in 2019. All localities should put the“Top manager” in charge of fund supervision. The principal comrades in charge should take charge of the fund themselves, strengthen the deployment and dispatch, coordinate and solve the key and difficult problems in the fund supervision, and ensure the smooth development of the supervision work. We should establish an incentive accountability mechanism, commend departments and individuals who have accomplished their tasks well, inform and criticize those who have failed to implement their work effectively, and seriously hold those who are suspected of dereliction of duty and dereliction of duty accountable according to law and regulations.

National Health Insurance Administration

February 20,2019

# May 21,2019

# **Notice of“Two pilots and one demonstration” for the supervision of medical insurance funds**

[2019] No. 17

Medical security bureaus of provinces, autonomous regions, municipalities directly under the central government and Xinjiang Production and Construction Corps:

In order to innovate supervision methods, enhance supervision efficiency, and accelerate the construction of a long-term mechanism for fund supervision, in accordance with the requirements of the notice of the State Medical Security Bureau on the supervision of the Medical Security Fund in 2019(No. 14 of the medical insurance issue (2019)) , recommended by the provincial (regional and municipal) health insurance departments and selected by the state health insurance administration, i hereby print and distribute to you the list and work plan of the National Health Insurance Administration's Fund Supervision Pilot, the fund supervision and credit system construction pilot, and the Health Insurance Intelligent Supervision Demonstration Site (hereinafter referred to as“Two pilot and one demonstration”) . Local medical insurance departments should strengthen the organization and leadership, carry out relevant work according to the work program, and actively promote the formation of replicable and replicable advanced experience in the supervision of medical insurance funds. The National Health Insurance Bureau will take various forms to strengthen supervision and guidance, exchange experiences and practices and timely promotion.

Annex 1: State Medical Security Bureau fund supervision“Two pilot one model” list of regions

Annex 2: medical security fund supervision“Two pilot one demonstration” work program

Office of the State Medical Security Bureau

May 21,2019

Annex 1:

State medical security bureau fund supervision“Two pilot one demonstration” list of regions

Annex 2:

Health insurance fund supervision“Two pilot one demonstration” work program

In order to implement the decisions and plans of the party Central Committee and the State Council, as well as the requirements of the circular of the state medical security bureau on doing a good job in the supervision of medical security funds in 2019(No. 14 of 2019) , this plan is formulated with solid progress in the pilot projects on innovation in fund supervision methods, the pilot project on the construction of a fund supervision and credit system, and the construction of a health insurance intelligent supervision and control demonstration site (hereinafter referred to as“Two pilot projects and one demonstration”) .

General requirements

(1) target tasks

In the past two years, significant progress has been made in the innovation of regulatory approaches, the construction of credit systems and intelligent monitoring in pilot (demonstration) regions, we will form experiences, models and standards that can be used for reference, replicated and popularized, and promote new breakthroughs in the supervision of medical security funds.

2. Basic principles

One is up and down linkage. We will establish a coordination mechanism for the work of“Two pilot and one demonstration”, clarify the responsibilities of the state, the pilot (demonstration) provinces and the pilot (demonstration) regions, and ensure their implementation in a coordinated manner.

Second, multi-party participation. Strengthen the coordination with relevant departments, a variety of channels, various forms of social resources to participate in the pilot (demonstration) work.

Third, strengthen assessment. Establish and improve the pilot (demonstration site) Regional Performance Evaluation Index system, combined with the actual performance of fund supervision and scientific evaluation.

Work content

1. Pilot projects to innovate in the way funds are regulated

Pilot areas may focus on one or more of the following aspects.

First, the introduction of social forces involved in supervision. To actively introduce third-party forces such as information technology service agencies, accounting networks and associations, and commercial and insurance institutions, so as to give full play to the role of professional technical support, we will establish and improve cooperation mechanisms such as data screening, financial auditing and medical record auditing, so as to realize forward movement, high efficiency and accuracy of fund supervision.

Second, the establishment of inter-departmental supervision mechanism. Strengthen cooperation with health, public security, market supervision, drug supervision and other departments, and further improve the unified deployment, joint inspection, Case Notification, case transfer, joint punishment and other work mechanisms; We will establish and improve working mechanisms for joint operations, “Multiple investigations in one case” and“Multiple investigations in one case”, and standardize work processes; we will coordinate the launching of major actions for fund supervision, and the investigation and handling of major cases, we will ensure that the entire chain of fund supervision is seamlessly linked up.

Third, other innovative ways of supervision. The pilot regions will explore their own options in the light of actual conditions.

2. Piloting the construction of a credit system for fund supervision

Pilot areas may focus on one or more of the following aspects.

First, the establishment of Fund Supervision Credit Evaluation Index System. We will explore ways to build a fund regulatory credit system, focusing on standards, norms and indicators, as well as the collection, evaluation and application of relevant information.

The second is to establish the dynamic management mechanism of appointed medical institutions. We will establish and improve the graded management system for designated medical and pharmaceutical institutions and the points management system for medical insurance physicians and medical insurance pharmacists, and explore the establishment of management mechanisms for designated medical and pharmaceutical institutions, such as comprehensive performance evaluation and elimination of the lowest rank, the establishment of sound management systems and mechanisms, the implementation of service agreements, standardized and rational use of health insurance funds, performance appraisal and other situations, as an important basis for the evaluation of appointed medical institutions, medical insurance doctors and medical insurance pharmacists, the evaluation results are related to budget management, inspection and audit, cost settlement, agreement management and so on.

Third, promote industry self-discipline. To encourage public and non-public medical institutions, retail pharmacies, physicians, pharmacists and other trade associations to develop industry norms and self-regulation, and to formulate and implement self-regulation conventions, promote industry norms and self-regulation.

Fourth, promote joint disciplinary action. We will actively promote the incorporation of fraudulent insurance practices into local credit management systems, establish a disciplinary system for dishonesty, and bring into play joint disciplinary deterrence.

(3) health insurance intelligent monitoring demonstration site

Demonstration sites could focus on one or more of the following areas to advance the work.

First, improve the intelligent monitoring function. In the light of the new features of fraudulent insurance practices, the database of basic information such as the catalogue of medical standards, the clinical knowledge database of diagnosis and treatment such as clinical guidelines, and the database of different rules should be further improved, for example, medical treatment standard class, medical insurance policy class, medical authenticity class, improve the coverage and accuracy of intelligent monitoring.

Second, enrich the dimension of intelligent monitoring. Using intelligent monitoring systems to strengthen process monitoring of clinical behaviour in areas where DRG payments are being piloted in countries and where payments are based on big-data disease-specific scores, we will enrich the dimensions of big data analysis and comparison, improve the effectiveness of monitoring, promote the application of new technologies such as video surveillance and face recognition, carry out timely management of the purchase, sale and storage of medicines, and improve the health insurance fund risk control system.

Third, the establishment of provincial-level centralized monitoring system. Based on the national medical insurance information system construction pilot work, to explore the centralized mode in the province to carry out intelligent monitoring work, and constantly improve the effectiveness of monitoring.

Division of responsibilities

(1) the state medical security bureau. Identify Pilot (demonstration) areas, coordinate relevant resources, organize and set up technical guidance expert groups, strengthen technical guidance and financial support. To coordinate and manage major issues in the work of the pilot (demonstration site) , organize exchanges and studies in the pilot (demonstration site) regions, master the overall progress of the work of the pilot (demonstration site) , and regularly review and evaluate the progress of the pilot (demonstration site) , summarizing the experience and effectiveness of the pilot (demonstration sites) .

(2) pilot demonstration provinces. To organize the implementation of the provincial (regional and municipal) pilot (demonstration site) work, report the progress of the work to the State Medical Insurance Bureau on a quarterly basis, and coordinate the solution of problems and difficulties in the progress of the pilot (demonstration site) work; Carry out communication and publicity work.

(3) pilot demonstration areas. Under the leadership of the local party committee or government, to set up a leading group for the pilot (demonstration site) work with the participation of multiple departments, and to formulate an implementation plan for the pilot (demonstration site) work in the light of the actual situation of the region, report on the progress and effectiveness of the work regularly, study and solve the problems and difficulties in the pilot (demonstration site) in a timely manner, and make solid progress in the pilot (demonstration site) work.

(4) expert group on technical guidance. According to the unified requirements of the State Medical Insurance Bureau, the pilot (demonstration site) to carry out the work of follow-up guidance, technical support and assessment.

The overall schedule

The overall work is divided into pilot start, pilot implementation, mid-term evaluation, summary evaluation of the four stages.

(1) pilot launch (may-june 2019)

The state medical insurance bureau determines the list of“Two pilot and one demonstration” areas, issues the working notice, and formulates the work plan. Under the guidance of provincial-level health insurance bureaus, the pilot areas will formulate specific implementation plans according to local realities and report to the state health insurance bureau for the record before June 14.

(2) pilot implementation (June 2019-may 2021)

The pilot (demonstration site) areas shall carry out the pilot (demonstration site) work according to the implementation plan, and report the progress, achievements and problems of the work in a timely manner.

(c) interim assessment (May-june 2020)

The state medical insurance administration has organized supervision and research, experience exchange, visits and visits to pilot (demonstration) areas to promote mutual learning and mutual assistance, guide the advanced and supervise the backward, the state medical insurance administration will organize and expand the construction of pilot (demonstration sites) as appropriate.

(D) summary assessment (may-june 2021)

The National Health Insurance Bureau organizes an overall assessment of the work of the pilot (demonstration site) , summarizes and refines advanced practices and mature experiences, and promotes their application.

Safeguard measures

1. Strengthen capacity building. Medical insurance departments at all levels should attach great importance to it, define its objectives and tasks, establish and improve working mechanisms, enlist the support of all parties, and ensure its in-depth and sustained development.

(2) strengthen supervision and dispatch. The medical security departments in the pilot (demonstration) regions shall promote the implementation of the work in strict accordance with the implementation plan. Provincial Medical Security Departments should strengthen the pilot (demonstration) work of the scheduling and evaluation, timely detection of problems and coordination to solve. The State Medical Insurance Bureau will organize and carry out centralized research at the right time, and regularly report the progress of the work of the pilot (demonstration site) .

(3) strengthening assessment and evaluation. The state medical insurance administration is focusing on the establishment and improvement of the organization and leadership mechanism of the pilot (demonstration site) as well as the inter-departmental, inter-sectoral and inter-institutional coordination working mechanism, the perfection of the supervision system, the efficiency of the investigation and handling of clues, and the investigation and punishment of the designated medical institutions, regular assessment and evaluation will be carried out to encourage the pilot (demonstration sites) to produce practical results.

# June 2,2020

# **Notice on the special management of the standardized use of medical insurance fund behavior of medical insurance designated medical institutions**

Medical Insurance Letter [2020] No. 9

Medical Insurance Bureaus and health committees of provinces, autonomous regions, municipalities directly under the central government and Xinjiang Production and Construction Corps:

In order to implement the decisions and plans of the CPC Central Committee and the State Council, establish and strengthen a long-term mechanism for the supervision of medical insurance funds, resolutely investigate and punish illegal acts in the field of medical insurance, and make unremitting and in-depth efforts to promote the supervision of medical insurance funds, according to the Social Insurance Law of the People's Republic of China, the regulations on the administration of medical institutions and other relevant laws and regulations, the State Medical Insurance Bureau and the National Health Commission have decided to launch a special program to regulate the use of medical insurance funds by designated medical institutions nationwide in 2020. I hereby notify you as follows:

General requirements

(1) guiding ideology

Guided by Thought on Socialism with Chinese Characteristics for a New Era, we will fully implement the spirit of the 19th National Congress of the Communist Party of China and the Second, Third and Fourth Plenary Sessions of the 19th Central Committee of the Communist Party of China, as well as the spirit of the Fourth Plenary Session of the 19th Central Commission for Discipline Inspection, and conscientiously implement the 　 's guidelines on medical security The spirit of a series of important instructions on work, In accordance with the Opinions of the Central Committee of the Communist Party of China and the State Council on Deepening the Reform of Medical Security System, we will strengthen policy guidance and joint law enforcement among departments, severely crack down on fraudulent insurance fraud with a zero-tolerance attitude, standardize the diagnosis and treatment services and charging behavior of designated medical institutions, improve the refined management level of medical institutions, ensure the safe, efficient and rational use of medical insurance funds, and enhance people's sense of acquisition, happiness and security.

(2) objectives

Through measures such as self-examination and rectification of designated medical institutions, spot checks and reexaminations by medical insurance and health departments, and flight inspections, we will strengthen joint efforts in the supervision of medical insurance funds, and urge designated medical institutions to improve their internal medical insurance management systems, • Raise the level of medical insurance management and the ability to prevent and control risks, and earnestly safeguard the safety of medical insurance funds.

(3) basic principles

1. Full coverage. First, it covers all designated medical institutions covered by the national health insurance; second, it covers all medical services and medical expenses that have been covered by the basic health insurance fund since January 1,2018.

2. Focus. In view of different types of medical institutions and their diagnosis and treatment service behavior, highlight the treatment focus, divided into categories of“Symptomatic treatment.”. For the public medical institutions, they should deal with such behaviors as illegal fees, repeated fees, over-coverage of medical insurance, non-indication diagnosis and treatment, package examination, package treatment, over-coverage of diseases, and the illegal inclusion of clinical experimental items in medical insurance reimbursement For non-public medical institutions focus on the treatment of false settlement, witness discrepancies, induced hospitalization, non-indicated hospitalization and other acts.

3. Classification. Fixed-point medical institutions that take the initiative to return the illegal gains in full before the end of the period of self-examination and rectification and all rectification can be mitigated, mitigated or exempted from punishment according to law. After the end of the period for self-examination and rectification, during spot checks or flight inspections, it was found that the self-examination and rectification of the designated medical institutions were not effective, that they did not return in full and on time their illegal gains, or that there were still illegal and illegal uses of the medical insurance funds, we must insist on zero tolerance, impose heavier penalties according to law and regulations, and expose them publicly.

Second, the content of governance

(1) unreasonable charges. Divide the fee-charging items into multiple items, charge the items with clear connotation repeatedly, charge beyond the standard fee; For self-created medical services that are not approved to be covered by health insurance, fees will be charged according to the price items of health insurance; repeated fees will be charged among the various package items; and settlement of high-cover diseases (disease group) will be made.

(2) exchange of items (drugs) . Changing out-of-catalogue drugs, diagnosis and treatment items and medical consumables into catalogue charges, applying high-priced charges to low-priced drugs, diagnosis and treatment items and medical consumables, etc. .

(3) non-standard diagnosis and treatment. The process of diagnosis and treatment in hospital was divided into two or more hospitalization, and the patients who did not reach the indication of hospitalization were admitted to hospital And inclusion of drugs or medical consumables in Medicare settlements beyond the scope of Medicare catalog payments.

(4) the issue of fictitious services. Forged, altered, fictitious settlement of medical services; forged patient information settlement; nominal hospitalization; forged, altered financial vouchers and sales and deposit bills settlement, etc. .

(5) other problems of violation of laws and regulations. Medical services that do not match their own qualifications will be included in the health insurance settlement; drug clinical trials will be included in the health insurance settlement in violation of regulations; and medical institutions that do not have fixed-point qualifications will be transferred to the health insurance network settlement.

Schedule

(1) formulating a work programme. According to the local COVID-19 epidemic prevention and control work, by stages and batches to carry out special governance. In principle, by the end of June, the health departments at the provincial level, in conjunction with the prominent problems of illegal and illegal activities in the area of medical insurance in the region and the weak links in the supervision of funds, will, on the basis of sufficient research and demonstration, to formulate a specific governance work plan and report it to the state medical insurance administration and the National Health Commission for the record; to urge and guide the medical insurance departments and health departments of the coordinating districts in the region to formulate specific governance work plans. Provinces with heavy responsibilities for epidemic prevention and control may apply to the state medical insurance administration for a postponement of the start time of special treatment work, and timely carry out follow-up work such as self-examination, self-correction, random review, flight inspection, etc. .

(2) conducting self-examination and self-correction. By the end of October, the medical insurance departments and health departments of the co-ordinating areas will organize the designated medical institutions in the co-ordinating areas to carry out comprehensive self-examination and rectification work. Each designated medical institution shall check and rectify each item according to the management contents, return the illegal income before the conclusion of the self-examination and rectification, and thoroughly analyze the root causes of the illegal and illegal problems, clear corrective measures, completion time limit and responsible person, will be self-check rectification written report to the local health insurance departments and health departments.

(3) conducting random checks and rechecks. The health departments of the co-ordinating regions shall, in conjunction with the health departments, reasonably arrange the time for conducting random checks and rechecks according to the self-examination and rectification situation of the designated medical institutions in the co-ordinating regions and the impact of the epidemic situation, in principle, all designated medical institutions in the region will be fully covered. All provincial-level medical insurance departments and health departments should strengthen overall coordination and supervision and inspection, and carry out spot checks on the self-examination and rectification of designated medical institutions in the region in a timely manner. By the end of November, the provincial medical insurance departments will report the special management work to the state medical insurance bureau and the State Health Commission.

(4) conducting flight inspections. The National Health Insurance Administration and the National Commission on Health will organize in due course flight checks covering all provinces of the country. Local medical insurance departments and health departments should actively cooperate with the national flight inspection and provide relevant information as required. All provincial medical insurance departments and health departments shall, in accordance with the requirements of relevant documents, carefully review the problem clues handed over by the flight inspection team, and carry out follow-up work in accordance with the law and regulations.

Job requirements

(1) improve the station position and make rectification and implementation. Medical security fund is the people's“Life-saving money”, we must always maintain the security of the fund as the primary task. Medical and health departments at all levels should strengthen political awareness, improve their political positions, strengthen organizational leadership, clarify timetables and road maps, and ensure that special governance tasks are completed on time and in good quality, we will comprehensively improve the management of health insurance funds and give full play to their performance.

(2) making a thorough investigation and rectification. Health Insurance Departments and health care departments at all levels should adhere to the problem-oriented approach, draw inferences from one another in the light of the problems found by medical institutions through self-examination, comprehensively investigate and plug loopholes, and urge rectification and implementation. They should formulate standards of conduct for the standardized use of health insurance funds, for illegal acts, according to the law and regulations of the classification of treatment; to combine the fund supervision of the new situation, new tasks and new requirements, timely adjustment of work ideas, innovative work measures, strengthen risk prevention and control, we will make effective efforts to strengthen the oversight of medical insurance funds.

(3) establishing rules and regulations and strengthening internal management. Designated medical institutions should strengthen self-discipline, sum up good practices, and form a long-term mechanism to strengthen the management of medical insurance. We should perfect the management system of hospital medical insurance, perfect the relevant systems of post responsibility, risk prevention and control, and accountability, and implement the system of responsibility of the President of medical insurance, for the medical personnel who have committed fraudulent insurance acts, one-vote veto should be carried out in such aspects as promotion, priority evaluation, performance appraisal, etc. , we will standardize the management of the purchase, sale, storage and financial management of drugs and consumables, and take the initiative to meet the needs of the supervision of medical insurance funds, launch various forms of publicity and education.

4. Strengthen coordination and form synergy in supervision. Medical Insurance Departments and health departments at all levels should strengthen communication and coordination, jointly supervise and guide designated medical institutions to strengthen self-examination and rectification, and find clues of illegal and illegal problems involving other areas, it is necessary to inform the relevant departments of public security and market supervision in a timely manner, actively explore the establishment of a working mechanism to investigate more cases and place more cases, and strive to form joint supervision efforts. For public medical institutions with serious violations of laws and regulations, relevant clues may be transferred to the disciplinary inspection and supervision organs at the same level, and the leading bodies of the medical institutions and the persons responsible shall be held accountable.

State health insurance administration

National Commission of Health

June 2,2020

# July 9,2020

# **Guidelines for promoting the reform of the regulatory system of the medical insurance fund**

Published by the State Council (2020)

The People's governments of provinces, autonomous regions and municipalities directly under the central government, the ministries and departments of the State Council, and the agencies directly under the State Council:

The medical insurance fund (hereinafter referred to as the medical insurance fund) is the people's“Medical money”, “Life-saving money”, the party Central Committee, the State Council attaches great importance to the safety of the medical insurance fund. Since the establishment of the basic medical security system, the coverage has been continuously expanded and the level of security has steadily increased, it has played a positive role in safeguarding people's health rights and interests, alleviating poverty caused by diseases, and promoting the reform of the medical and health system. In particular, in the course of combating the epidemic of covid-19, relevant policies should be issued in a timely manner to bring the diagnosis and treatment of covid-19 into the coverage of the medical insurance fund and prepay part of the funds, to ensure that patients will not be affected by the cost of medical treatment, treatment hospitals will not be affected by the payment policy of treatment, reflects the superiority of our socialist system. But we should also see that the supervision system is not perfect, the incentive and restraint mechanism is not perfect, the efficiency of the use of health insurance funds is not high, fraud insurance frequent occurrence, the fund supervision situation is more serious. With the approval of the State Council, the following suggestions are put forward for the reform of the medical insurance fund supervision system in order to improve the medical insurance governance ability in an all-round way, thoroughly purify the operating environment of the system and strictly abide by the fund safety Red Line.

General requirements

1. Guiding ideology. Guided by Thought on Socialism with Chinese Characteristics for a New Era, fully implement the spirit of the 19th National Congress of the Communist Party of China and the Second, Third and Fourth Plenary Sessions of the 19th Central Committee, and accelerate the reform of the medical insurance fund supervision system in accordance with the decisions and arrangements of the Party Central Committee and the State Council, build a full-field and full-process fund security prevention and control mechanism, severely crack down on insurance fraud, safeguard social fairness and justice, continuously improve the people's sense of gain, and promote the healthy and sustainable development of my country's medical security system.

(2) basic principles. We will continue to improve rule of law and regulation in accordance with the law, and ensure that regulation of funds is in accordance with law, fair, and just. We will continue to promote government-led and social governance, and usher in a new era in fund regulation. We will continue to pursue reform, innovation, coordination, and efficiency, and continue to improve the ability and performance of fund regulation. Adhere to discipline dishonesty, encourage integrity, guide the supervision object to enhance self-discipline awareness, create a good atmosphere.

(3) major objectives. By 2025, a system of supervision and enforcement of the medical insurance funds will be basically completed, with the rule of law as the guarantee, credit management as the foundation, multi-form inspections and big data supervision as the backing, an all-round supervision pattern combining party committee leadership, government supervision, social supervision, industry self-discipline and individual trustworthiness will ensure that the supervision of medical insurance funds is rule of law, professionalized, standardized and normalized, and continuously improve it in practice.

1. clear responsibility for supervision

(4) Strengthen party leadership. Adhere to and strengthen the party's overall leadership, and continue to improve the health insurance fund supervision party building leadership system and work mechanism. We will urge medical security departments and designated medical institutions to strengthen the construction of party organizations at the grass-roots level, and give full play to the role of party organizations as a bastion of fighting and the vanguard role of party members. To improve the mechanism of supervision and restraint for the leading bodies and personnel of public designated medical institutions, in particular the principal responsible persons, and to strengthen the supervision, assessment and discipline-enforcement accountability for the fulfilment of their political and fund supervisory responsibilities, • Establishing a bottom line for oversight.

(5) strengthening government supervision. To give full play to the leading role of the government in building the rule of law in fund supervision, setting standards, enforcing administrative laws and sharing information, and to supervise and manage, in accordance with the law, the conduct of medical services and medical expenses covered by the medical insurance, we will standardize the operation of medical insurance, investigate and punish illegal acts in accordance with the law, strictly enforce legal responsibilities and increase penalties. We will strengthen the responsibility of medical security departments for supervising funds, and ensure that they play an effective role in their oversight. Establish a fund supervision mechanism led by the medical insurance department and attended by relevant departments, and coordinate the major actions of fund supervision and the investigation and handling of major cases. It will draw up a list of rights and responsibilities and clarify the regulatory responsibilities of medical insurance funds.

(6) promoting self-regulatory management of industries. We will actively promote the development of organizations in the pharmaceutical and health sectors, guide and support their role in formulating management norms and technical standards, standardizing practice and management services, and promoting self-discipline in the sector. Designated medical institutions should effectively implement the main responsibility of self-management, establish and improve internal management mechanisms such as medical insurance services, human resources, finance, system security, etc. , they should conscientiously accept medical insurance supervision and social supervision.

3. Promote reform of the regulatory system

(7) Establish and improve the system of supervision and inspection. A“Double random and open” regulatory mechanism will be introduced, and a multiform inspection system combining routine inspections, special inspections, flight inspections, key inspections and expert reviews will be established and improved, the targets, priorities and contents of inspections will be clarified. Standardize start-up conditions, work requirements and workflow, clarify the rights and obligations of all parties to ensure openness, fairness and fairness. A mechanism will be set up to allow departments to join forces and conduct joint inspections so as to form synergy in supervision. Third parties, such as information technology service organizations, accounting networks and associations, commercial insurance organizations and so on, should be actively introduced to participate in the supervision of health insurance funds, establish and improve the system of government purchasing services, and promote payment according to service performance, we will enhance the professionalism, accuracy and effectiveness of regulation.

(8) establishing an intelligent monitoring system in an all-round way. We will accelerate the standardization and informationization of health insurance, strictly implement the requirements for integrating and sharing government information systems, integrate them with existing systems, and strengthen information exchange and sharing among departments to avoid duplication of efforts. China will establish and improve an intelligent health insurance monitoring system and strengthen the application of big data. We will strengthen the guidance and audit of clinical diagnosis and treatment in designated medical institutions, and strengthen supervision in advance and in the course of events. In the light of the characteristics of fraudulent insurance, the database of basic information standards such as drugs, medical treatment items and medical service facilities, as well as medical knowledge databases such as clinical guidelines, have been continuously improved, and the rules of intelligent monitoring have been improved to enhance the functions of intelligent monitoring. Real-time management of the purchase, sale and storage of medicines and medical consumables will be implemented. It will expand the use of technologies such as video surveillance and biometrics. We will promote instant settlement for medical and drug purchases in other places, and bring all settlement data online. We will accelerate the establishment of a centralized and unified intelligent monitoring system at the provincial and national levels, so as to realize the transformation of fund supervision from manual single-sampling audit to all-round, full-process and full-link intelligent monitoring of big data.

(9). Establish and improve the reward system for reporting crimes. The departments of medical security and finance at the regional level and above shall establish and continuously improve a system of rewards for reporting medical security violations of laws, regulations and contracts, and reward informants in accordance with relevant provisions. Smooth the channels of complaints and reports, standardize the acceptance, inspection, processing, feedback and other work processes and mechanisms to enhance privacy protection, effectively protect the information security of informants. We will improve the standards for rewarding reports, cash in rewards in a timely manner, and encourage the active participation of the public and all sectors of society in supervision.

Establish a credit management system. An information reporting system for designated medical institutions will be established. To establish medical institutions and insured medical insurance credit records, credit evaluation system and points management system. To innovate the comprehensive performance evaluation mechanism of the appointed pharmaceutical institutions, which relates the credit evaluation results and the comprehensive performance evaluation results with the budget management, inspection and Audit, the appointed agreement management and so on. We will strengthen and standardize the management of the lists of persons subject to joint incentives for keeping promises and those subject to joint disciplinary sanctions for dishonesty in the field of medical security, and implement joint incentives for keeping promises and joint disciplinary sanctions for dishonesty in accordance with the law and regulations. Industry associations are encouraged to develop industry norms and self-discipline, formulate and implement self-discipline conventions, and promote industry norms and self-discipline.

(10) establishing a comprehensive regulatory system. To adapt to the characteristics of medical insurance management services, establish and improve inter-departmental coordination, coordinated supervision of the comprehensive supervision system, the implementation of grid management. We will promote information sharing and connectivity, and improve the mechanism for coordinated law enforcement. The relevant departments shall deal with the relevant units and individuals seriously in accordance with the provisions of laws and regulations and the limits of their duties and powers. A working mechanism to link up the execution of sentences against insurance fraud will be established and improved. The medical security departments shall be responsible for supervising and administering the medical services and medical expenses that are covered by the medical insurance, standardizing the medical insurance business, and investigating and punishing illegal acts in the field of medical security according to laws and regulations. The health department is responsible for strengthening the supervision of medical institutions and the medical service industry, and standardizing the medical service behavior of medical institutions and their medical personnel. The Market Supervision Department is responsible for price supervision and inspection in the medical and health sector, and the Drug Supervision Department is responsible for the administration of licensed pharmacists, the market and drug regulatory departments shall, in accordance with their respective responsibilities, be responsible for the supervision of drug circulation and the regulation of drug operation. The audit institution is responsible for following up the implementation of policies and measures related to strengthening the supervision of the medical insurance fund, urging the relevant departments to perform their supervision duties, paying continuous attention to all kinds of fraudulent insurance issues, and promptly transferring them to the relevant departments for investigation and punishment. The Public Security Department is responsible for investigating and punishing all kinds of criminal acts such as fraud and insurance according to law, and carrying out timely investigations into the transferred suspected criminal cases. Other relevant departments shall carry out relevant work in accordance with their duties.

(11) Improve the system of social supervision. We will encourage and support all sectors of society to participate in the supervision of health insurance funds, and achieve positive interaction among government supervision, social supervision and supervision by public opinion. • Establish an information disclosure system. The operating agencies shall regularly announce to the public the income, expenditure, balance and income of the fund and accept social supervision. To establish a system of social supervisors for medical insurance funds, and to employ deputies to the National People's Congress, members of the Chinese People's Political Consultative Conference, representatives of the masses and the news media, etc. , as social supervisors, and conducting extensive and in-depth supervision over designated medical and pharmaceutical institutions, operating agencies, and insured personnel. Actively invite the news media to participate in the work of flight inspection, undercover investigation, through press conferences, media briefings and other forms, published anti-fraud insurance results and typical cases.

Fourth, improve the safeguard measures

(12) Strengthen the rule of law and standardized protection for the supervision of health insurance funds. Formulating regulations and supporting measures on the supervision and administration of the use of the medical insurance fund. We will improve the agreement management system for designated medical institutions, and establish and improve a dynamic management and withdrawal mechanism for designated medical institutions. We should perfect the monitoring mechanism of medical insurance, extend the supervision object from medical institutions to medical personnel, and shift the supervision focus from the control of medical expenses to the control of both medical expenses and medical service performance. We will issue and implement diagnostic and treatment standards for the medical and health sector, gradually develop clinical pathway management, improve and implement the clinical pharmacist system, the prescription review system, and strengthen the application and evaluation of such standards and norms in clinical practice.

(13) Strengthen the ability of supervision and inspection of medical insurance funds. We will strengthen capacity-building for fund supervision and inspection, establish a sound fund supervision and law enforcement system, strengthen personnel strength, and strengthen technical means. We should straighten out the relationship between administrative supervision and management agreement, clarify the boundary of responsibilities between administrative supervision and management audit, and strengthen the link-up of work. Implement the responsibilities of the administrative supervision and management of the management agreements, cost monitoring, and audit of the management agencies. Establish and perfect the internal control system of the fund management organization, regularly employ a third party to evaluate the risk of the fund management organization's internal control, and strengthen the fund management's internal control line of defense. We will strengthen financial support at all levels and strengthen fund supervision through government procurement services. We will ensure that medical and pharmaceutical institutions provide the personnel, equipment, and related facilities necessary to provide health care services.

(14) we will increase punishment for fraudulent insurance activities. We will severely punish units and individuals who defraud insurance by means of judicial, administrative and contractual means. The legislative interpretations of the standing committee of the National People's Congress on fraudulent insurance practices will be strictly implemented, and cases involving suspected crimes will be transferred to judicial organs in accordance with the law for investigation and criminal liability. The health care sector will increase administrative penalties in accordance with the law and regulations. To actively bring into play the joint action of departments in punishing designated medical institutions that have been verified by the medical security departments and whose cases of fraud and insurance fraud are particularly serious, the departments of Health and drug supervision shall, in accordance with the law, impose penalties such as suspension and rectification, suspension of the qualification of practicing (operation) and restriction of employment, so as to enhance the deterrent effect of punishment. Fixed-point medical institutions and individuals with serious fraud insurance cases shall be included in the list of targets of joint punishment for breach of trust, and joint punishment shall be imposed.

(15) Promote the reform of the medical security system as a whole. We will deepen reform of the mode of payment for health insurance, strengthen budget management of funds, and provide early warning on risks. To establish the management system of medical security treatment list, to determine the content of basic security, to clarify the boundary of treatment and payment, to clarify the power of policy adjustment. We will strengthen the role of medical insurance in stimulating and restricting health care and medicine, strengthen the responsibility of overall regional supervision, and optimize the foundation of fund supervision.

(16) Promoting the reform of the pharmaceutical service system in a coordinated manner. Deepening supply-side reform of medical services. We will accelerate the comprehensive reform of public hospitals, establish and improve modern hospital management systems, and standardize diagnosis and treatment practices. We will standardize the use of appropriate medical technologies to address common diseases and health problems. We will continue to improve the market-oriented price formation mechanism for drugs and medical consumables, and improve the linkage mechanism between medical insurance payment and tender purchase prices. We will strengthen supervision and inspection of the quality of accounting information in the pharmaceutical industry, and intensify efforts to address the problem of inflated prices for drugs and high-value medical consumables.

Job requirements

(17) Strengthen organizational and leadership. Local People's governments at all levels should fully understand the importance of promoting the reform of the medical insurance fund supervision system, and strengthen leadership, unified deployment and coordinated progress. The Administrative Department of Health Insurance is the main department responsible for the supervision of health insurance funds, the departments of development and reform, public security, justice, finance, human resources, Social Security, health, auditing, taxation, market supervision, banking and insurance supervision, administration of traditional Chinese medicine, and drug supervision shall perform their corresponding duties in accordance with the law, we will promote reform in a coordinated manner. We should increase the exchange of information, achieve interconnected responses and promote the coordinated application of comprehensive regulatory results.

(18) establish a working mechanism. The People's governments at the provincial level should establish an incentive accountability mechanism and include the work of combating fraud and fraud in the relevant work assessment. We should strengthen responsibility, actively find problems, seriously investigate and punish problems according to law and regulations, have zero tolerance for fraudulent insurance practices, and publicly expose typical cases. We will effectively carry out our supervisory duties and work in concert to ensure that our staff, responsibilities and measures are in place.

(19) Make good use of publicity and guidance. The relevant departments in all regions should vigorously publicize the importance of strengthening the supervision of the medical insurance fund, mobilize all social parties to jointly promote the reform of the supervision system, and innovate the supervision methods and methods according to the actual conditions, effective supervision methods and models should be summarized and disseminated in a timely manner. We should strengthen the guidance of public opinion, respond positively to social concerns, widely publicize advanced models and strive to create a good atmosphere for reform.

State Council General Office

June 30,2020

# December 17,2020

# **Notice on the“Review” of the specialized management of point-of-care medical institutions**

Medical Insurance Office (2020) No. 58

Medical Security Bureaus and health committees of provinces, autonomous regions, municipalities directly under the central government and Xinjiang Production and Construction Corps:

This year, health Insurance and health care departments at all levels conscientiously implemented the requirements of the“Notice of the state medical insurance administration and the State Health Care Commission on carrying out the special management work on the standardized use of health insurance funds by designated medical institutions under the medical insurance system”(medical insurance letter [2020] No. 9) , it has continuously built a high-pressure situation to combat fraud and insurance fraud and achieved phased results. Recently, the news media have exposed some problems of induced hospitalization and false hospitalization in some designated medical institutions in Taihe County, Anhui Province, which are of a bad nature and extremely bad influence, reflecting that the special governance has not gone deep enough, the situation of fraud insurance is still serious, there are still loopholes in the supervision of health insurance funds, and the responsibilities of grassroots supervision have not yet been compacted. We must take this as a lesson, draw inferences from one another, and strike with a heavy fist, we must strengthen oversight and resolutely prevent the recurrence of such problems. After research, we decided to immediately carry out special treatment in designated medical institutions nationwide to“Look Back” and focus on combating induced hospitalization, false hospitalization and other fraud insurance problems. The specific requirements are as follows:

Time Frame

Special governance“Look Back” for all the designated medical institutions nationwide health insurance. The date of publication is January 31,2021.

Second, the content of governance

Induced hospitalization. The use of“Free medical examination, car pick-up and transport” and other names or through“Paid recommendations” and other ways to induce people who do not meet the requirements of hospitalization, such as medical insurance fund fraud.

(2) false hospitalization. The practice of lying in bed and impersonating patients to cheat the medical insurance fund by fabricating medical treatment items and forging medical documents.

Job requirements

(1) compaction of regulatory responsibilities. Each regional health insurance department and Health Department is the lead unit of this special governance“Looking back”. They should join forces with public security, market supervision, Discipline Inspection and supervision departments to set up special work teams, improve the working mechanism, work program, detailed governance measures, clear division of responsibilities, the implementation of responsibility to people. We should focus on key areas, set up ledgers, reverse time, and impose severe penalties to effectively improve governance. All provincial-level medical insurance and health departments should strengthen the unified dispatch and supervision and guidance for the overall planning of regional special governance, and adopt the methods of spot check, recheck and centralized supervision, so as to effectively consolidate the responsibility of supervision and inspection at the grass-roots level. The state medical insurance administration and the National Health Commission will jointly carry out supervision and inspection at an appropriate time.

(2) insist on universal coverage. All localities should use the medical insurance intelligent audit, intelligent monitoring information system to screen the suspected illegal hospitalization settlement data in 2020 with high frequency of hospitalization, relatively concentrated admission time and close amount of discharge reimbursement within their jurisdiction, focus on the screening of archived card poor households, centralized support for five-insurance households, elderly patients with minor hospital settlement. We should make full use of the inspection forces at the city and county levels, and adopt the methods of cross-examination to carry out on-the-spot verification, medical record examination, visiting investigation and surprise inspection for suspicious clues, so as to realize the full coverage of supervision and inspection without blind spots.

(3) strengthening social supervision. We will step up publicity, encourage public participation in oversight, and actively report fraud and insurance fraud. To improve the process of handling clues to report, make full use of clues to report as a starting point to report clues, citing three similar issues within the jurisdiction, similar medical institutions included in the scope of verification. We will implement reward measures for reports, reward rewards according to law and regulations, and create an atmosphere of coordinated supervision in which the whole society pays attention to, participates in, and supports the supervision of funds. A typical case discovered during the period of special administration should be discovered and a case publicly exposed so as to strengthen the deterrent effect.

(4) to step up punishment. If a designated medical institution is found to have committed fraud, the medical insurance department shall order it to return the medical insurance fund and impose a fine of not less than 2 times and not more than 5 times the amount defrauded; Ordering designated medical institutions to suspend designated medical services or rescind service agreements; punishing relevant medical personnel of designated medical institutions by the health departments in accordance with the law; The directly responsible personnel in charge and other directly responsible personnel shall be dealt with according to law and regulations. Medical Insurance, health and other departments staff have been found to abuse of power, dereliction of duty, favouritism malpractice, in accordance with the law and regulations should be seriously held accountable. Those who are suspected of violating relevant laws and regulations shall be handed over to the relevant competent authorities for handling according to law.

(5) strengthen the work schedule. Provincial Medical Insurance and health departments should carry out weekly dispatch of special administration“Looking Back” work, and before 12:00 every Monday morning, the progress of special governance in the province last week in written form simultaneously reported to the state medical insurance bureau and the State Commission of Health, in case of major reports at any time. By the end of January 2021, all provincial-level medical insurance and health departments will submit special“Looking Back” summary reports to the state medical insurance administration and the state commission of Health.

Office of State Medical Insurance Bureau, General Office of National Health Commission

December 17,2020

# February 19,2021

# **Regulation on the supervision and management of the use of the medical insurance fund**

The regulations on the supervision and administration of the use of medical security funds were adopted by the State Council at its 117th Executive meeting on December 9,2020 and are now promulgated for implementation from May 1,2021.

Premier Li Keqiang

January 15,2021

Regulations on the supervision and administration of the use of medical security funds

Chapter I general

Article 1 in order to strengthen the supervision and administration of the use of medical security funds, ensure the safety of funds, promote the effective use of funds, and safeguard the legitimate rights and interests of citizens in medical security, these regulations are formulated in accordance with the social insurance law of the People's Republic of China and other relevant legal provisions.

Article 2 these regulations shall apply to the use, supervision and administration of the basic medical insurance (including maternity insurance) funds, medical assistance funds and other medical security funds in the territory of the People's Republic of China.

Article 3 the use of medical security funds shall be centered on the health of the people, the level of security shall be commensurate with the level of economic and social development, and the principles of legality, safety, openness and convenience shall be observed.

Article 4 the supervision and administration of the use of medical security funds shall be a combination of government supervision, social supervision, industry self-discipline and individual trustworthiness.

Article 5 the People's governments at or above the county level shall strengthen their leadership over the supervision and administration of the use of medical security funds, and establish and improve the mechanism for the supervision and administration of the use of medical security funds and the system for the supervision and administration of funds for law enforcement, we will strengthen capacity-building in the supervision and management of the use of medical security funds to ensure their management.

Article 6 the Administrative Department for Medical Security under the State Council shall be in charge of the supervision and administration of the use of the national medical security funds. Other relevant departments under the State Council shall be responsible for the supervision and administration of the use of the relevant medical security funds within their respective areas of responsibility.

The administrative departments of medical security under the local people's governments at or above the county level shall be responsible for the supervision and administration of the use of medical security funds in their respective administrative regions. Other relevant departments of the local people's governments at or above the county level shall be responsible for the supervision and administration of the use of the medical security funds within their respective areas of responsibility.

Article 7 the state encourages and supports the media in publicizing the laws, regulations and knowledge of medical security, and exercises public opinion supervision over the use of medical security funds. Publicity reports on medical insurance shall be true and impartial.

People's governments at or above the county level and their administrative departments such as medical security shall solicit opinions in writing and hold symposiums, we will listen to the opinions of deputies to the National People's Congress, members of the Chinese People's Political Consultative Conference and representatives of insured personnel on the use of medical security funds, open channels for social supervision, and encourage and support all sectors of society to participate in the supervision of the use of medical security funds.

Medical institutions, drug dealers (hereinafter collectively referred to as pharmaceutical institutions) and medical and health trade associations shall strengthen self-discipline of the industry, regulate the conduct of medical services, and promote the regulation and self-restraint of the industry, guide the lawful and rational use of medical security funds.

Chapter two, the use of funds

Article 8 the use of the medical security fund shall conform to the payment scope prescribed by the state.

The payment scope of the medical security fund shall be formulated by the administrative department of Medical Security under the State Council in accordance with the law. The People's governments of provinces, autonomous regions and municipalities directly under the Central Government shall, in accordance with the authorities and procedures prescribed by the state, supplement and formulate specific items and standards for payment of medical security funds within their respective administrative regions, and reporting them to the administrative department of Medical Security under the State Council for the record.

Article 9 the State shall establish and improve a nationwide unified management system for medical security and provide standardized and standardized medical security services, full coverage of provinces, cities, counties, townships (streets) and villages (communities) shall be realized.

Article 10 a Medical Security Agency shall establish and improve the system of business, finance, Safety and risk management, and do a good job in the management of service agreements, monitoring of expenses, allocation of funds, examination and payment of benefits, etc. , and regularly to the public health care fund income, expenditure, balance and other conditions, subject to social supervision.

Article 11 the Medical Security Agency shall establish a collective bargaining and consultation mechanism with the designated medical institutions, and reasonably determine the budgetary amount of the medical security fund and the time limit for disbursement by the designated medical institutions, according to the need of protecting public health and managing service, we should negotiate and sign service agreement with the appointed medical institutions, standardize the medical service behavior, and clarify the behavior and responsibility of violating the service agreement.

The Medical Security Agency shall promptly release to the public the list of designated medical institutions that have signed service agreements.

The Administrative Department of Medical Security shall strengthen supervision over the conclusion and performance of service agreements.

Article 12 the Medical Security Agency shall settle and allocate the medical security fund in a timely manner as stipulated in the service agreement.

Designated Medical Institutions shall provide medical services in accordance with relevant regulations, improve the quality of services, make rational use of medical security funds, and safeguard citizens' health rights and interests.

Article 13 if a designated pharmaceutical institution violates the service agreement, the Medical Security Agency may urge it to fulfill the service agreement, to suspend or withhold the payment of fees, recover the illegal fees, suspend the medical services of the persons responsible or the departments involved in the use of the medical security fund according to the service agreement, until the service agreement is rescinded; Designated medical institutions and their relevant responsible personnel shall have the right to make representations and pleadings.

If the medical security agency violates the service agreement, the designated medical institution shall have the right to request correction or to request the administrative department of Medical Security to coordinate and handle, supervise and rectify, they may also apply for administrative reconsideration or file an administrative lawsuit in accordance with the law.

Article 14. Designated medical institutions shall establish an internal management system for the use of medical security funds, with special institutions or personnel in charge of the management of the use of medical security funds, and establish and improve assessment and evaluation systems.

Designated Medical Institutions shall organize training in the relevant systems and policies concerning medical security funds, regularly inspect the use of the medical security funds of their units, and promptly correct irregularities in the use of medical security funds.

Article 15 designated pharmaceutical institutions and their staff shall implement the administrative provisions on seeking medical treatment and purchasing drugs under their real names, verify the medical insurance vouchers of insured persons, and provide reasonable and necessary medical services in accordance with the diagnostic and therapeutic norms, the insured shall provide the cost documents and relevant information to the insured person truthfully, and shall not be disassembled or hospitalized in a hospital bed, and shall not overdiagnose and treat, overexamine, disassemble prescriptions, overprescribe drugs, or prescribe drugs repeatedly in violation of the diagnostic and treatment norms, no repeated charges, over-standard charges, or charges for disassembling items, no cross-trading of medicines, medical consumables, medical treatment items and service facilities, or inducing or assisting others to seek medical treatment under false names or to purchase medicines.

A designated medical institution shall ensure that the expenses paid by the medical security fund comply with the prescribed scope of payment, and provide medical services beyond the scope of payment by the medical security fund, except in exceptional circumstances such as emergency treatment or rescue, the insured persons or their close relatives or guardians shall give their consent.

Article 16 A designated pharmaceutical institution shall, in accordance with relevant regulations, keep financial accounts, accounting vouchers, prescriptions, medical records, records of medical examinations, details of expenses, records of the entry and exit of drugs and medical consumables, and other materials, to transmit the relevant data on the use of the medical security fund through the medical security information system in a timely manner, and to report the information needed for the supervision and management of the use of the medical security fund to the medical security administrative department; Making public information on medical expenses and fee structure to the public, and accepting public supervision.

Article 17 a insured person shall hold his own medical insurance certificate for medical treatment, purchase medicine and present it for examination on his own initiative. Insured persons shall have the right to request designated medical institutions to produce truthfully cost documents and relevant information.

The insured personnel shall properly keep their own medical insurance certificates to prevent others from using them under false names. If, for special reasons, it is necessary to entrust another person to purchase drugs on its behalf, the identity certificates of the ENTRUSTOR and the trustee shall be provided.

Insured persons shall enjoy medical insurance benefits in accordance with relevant regulations and shall not enjoy such benefits repeatedly.

The insured personnel shall have the right to request the medical security agencies to provide medical security consultation services, and to put forward suggestions for improving the use of the medical security funds.

Article 18 during the use of the medical security fund, administrative departments such as medical security, medical security agencies, designated medical institutions and their staff shall not accept bribes or obtain other illegal income.

Article 19 insured persons shall not take advantage of the opportunity of enjoying medical security to resell drugs, accept the return of cash, goods in kind or obtain other illegal benefits.

Designated pharmaceutical institutions shall not provide facilities for insured persons to take advantage of the opportunity to enjoy medical security to resell drugs, receive cash or in kind returns or obtain other illegal benefits.

Article 20, medical security agencies, designated medical institutions and other units, as well as their staff and insured personnel, shall not forge, alter, conceal, alter or destroy medical documents, medical certificates, accounting vouchers, electronic information, etc. , or make up medical service items to defraud the medical security fund.

Article 21 the special funds of the medical security fund shall be used exclusively, and no organization or individual may encroach upon or misappropriate them.

Chapter Three, supervision and management

Article 22 the departments of Medical Security, health, traditional Chinese medicine, market supervision and administration, Finance, auditing and public security shall work in a division of work and cooperate with each other, we will establish mechanisms for communication, coordination and case transfer, and work together to supervise and manage the use of medical security funds.

The Administrative Department of Medical Security shall strengthen supervision over the medical services and medical expenses covered by the medical security fund, and standardize the medical security operation, and investigate and punish, in accordance with the law, the illegal use of medical security funds.

Article 23 the Administrative Department of Medical Security under the State Council shall be responsible for formulating measures for the administration of service agreements, standardizing, simplifying and optimizing the procedures for designated applications, professional assessment, consultation and negotiation by medical institutions, to prepare and regularly revise model service agreements.

The administrative department of Medical Security under the State Council shall, in formulating measures for the administration of service agreements, listen to the opinions of relevant departments, medical and pharmaceutical institutions, trade associations, the public, experts, etc. .

Article 24 the Administrative Departments of medical security shall strengthen the exchange and sharing of information with relevant departments, innovate the methods of supervision and management, and promote the use of information technology, establishing a national unified, efficient, compatible, convenient and safe medical security information system, implementing real-time dynamic and intelligent monitoring of big data, and strengthening the management of the whole process of using shared data, ensuring the safety of shared data.

Article 25 the Administrative Department for Medical Security shall, in the light of such factors as the risk assessment of the medical security fund, the clues for reporting complaints, and the monitoring of medical security data, determine the inspection focus and organize special inspections.

Article 26 the administrative departments of medical security may conduct joint inspections with the departments of health, Traditional Chinese medicine, market supervision and administration, finance and public security.

The use of cross-regional health care funds shall be examined by the health care administrative department designated by the same higher health care administrative department.

The administrative department for medical security may take the following measures in the implementation of supervision and inspection:

(1) to enter the scene for inspection;

(2) to question the persons concerned;

(3) to request the inspected objects to provide documents and information related to the matters under inspection, and to give explanations and explanations;

(4) to collect relevant information and materials by means of recording, audio recording, video recording, photography or reproduction;

(5) to seal up materials that may be transferred, concealed or lost;

(6) engaging qualified third-party institutions and professionals such as accounting networks and associations to assist in carrying out the inspection;

Other measures prescribed by laws and regulations.

Article 28 the Administrative Department for Medical Security May, in accordance with the law, entrust organizations that meet the legal requirements to carry out the administrative law enforcement work for medical security.

Article 29 to supervise and inspect the use of medical security funds, there shall be no less than two supervisors and inspectors, and they shall show their law enforcement certificates.

When the administrative department of Medical Security carries out supervision and inspection, the subject under inspection shall cooperate, provide relevant materials and information truthfully, and shall not refuse or obstruct the inspection or falsely report or conceal the report.

Article 30 where a designated medical institution is suspected of defrauding the expenses of the medical security fund, during the period of investigation, the administrative department of medical security may take measures such as increasing the frequency of supervision and examination and strengthening the monitoring of expenses, to prevent further losses. If a designated medical institution refuses to cooperate with the investigation, with the approval of the principal person in charge of the medical security administrative department, the medical security administrative department may request the Medical Security Agency to suspend the settlement of the medical security fund. After investigation, those who defraud medical security fund shall be dealt with in accordance with the provisions of Article 40 of these regulations; those who do not defraud medical security fund shall be settled in accordance with the provisions.

If the insured person is suspected of defrauding the medical security fund and refuses to cooperate with the investigation, the medical security administrative department may request the Medical Security Agency to suspend the settlement of medical expenses through the network. The medical expenses incurred during the suspension of settlement through the network shall be fully covered by the insured personnel. After investigation, those who defraud medical security fund shall be dealt with in accordance with the provisions of Article 41 of these regulations; those who do not defraud medical security fund shall be settled in accordance with the provisions.

Article 31 before making an administrative punishment or decision on administrative treatment for an act in violation of these regulations, the Administrative Department of Medical Security shall listen to the statements and pleas of the parties concerned; make an administrative punishment or decision on administrative treatment, the party concerned shall be informed of the right to apply for administrative review or to initiate administrative proceedings in accordance with the law.

Article 32: Administrative Departments such as medical security, medical security agencies, accounting networks and associations and their staff, it shall not use the data or related information of the investigated objects obtained or known in the course of work for purposes other than the supervision and administration of the use of the medical security fund, it shall not divulge, tamper with, damage or unlawfully provide any personal information or trade secrets of the parties to others.

Article 33 the Administrative Department of Medical Security under the State Council shall establish a credit management system for designated medical institutions and personnel, and supervise and administer them according to their credit rating and classification, the results of daily supervision and inspection and administrative punishment shall be incorporated into the national credit information sharing platform and other relevant information disclosure systems, and disciplinary actions shall be imposed in accordance with the relevant provisions of the state.

Article 34 the Administrative Departments for medical security shall regularly publish the results of supervision and inspection on the use of medical security funds to the public, increase the exposure of cases of illegal use of medical security funds, and accept social supervision.

Article 35 any organization or individual shall have the right to report and complain against any illegal or illegal act that infringes upon the medical security fund.

The Administrative Department of Medical Security shall unblock the channels for reporting complaints, deal with such complaints in a timely manner in accordance with the law, and keep the information of the informant confidential. Informants shall be given rewards in accordance with relevant state regulations for the verification of reports.

Chapter four, legal liability

Article 36 where a medical security agency has one of the following circumstances, the administrative department for Medical Security shall order it to make corrections and impose sanctions on the person in charge who is directly responsible and other persons who are directly responsible according to law:

(1) the system of business, finance, security and risk management has not been established and improved

(2) failing to perform the duties of service agreement management, cost monitoring, fund allocation, treatment review and payment, etc. ;

The income, expenditure and balance of the medical security fund shall not be disclosed to the public on a regular basis.

Article 37, by forging, altering, concealing, altering, or destroying medical documents, medical certificates, accounting vouchers, electronic information, or making up medical service items, those who defraud the medical security fund shall be ordered by the medical security administrative department to return it, and shall be fined more than twice and less than five times the amount defrauded, those in charge who are directly responsible and those who are directly responsible shall be punished according to law.

Article 38 a designated medical institution shall be ordered by the administrative department for Medical Security to make corrections under any of the following circumstances and may interview the person in charge concerned; if losses are caused to the medical security fund, it shall be ordered to return it, a fine of not less than 100% but not more than 200% of the amount of the damage caused; refusing to correct or causing serious consequences, ordering the designated medical institutions to suspend the medical services used by the relevant responsible departments for not less than six months and not more than one year in connection with the medical security fund; violations of other laws and administrative regulations shall be dealt with by the relevant competent departments in accordance with the law:

(1) decomposing hospitalization and hanging bed hospitalization;

(2) over-diagnosis and treatment, over-examination, decomposition of prescriptions, over-prescription, repeated prescription or provision of other unnecessary medical services;

(3) repeated charges, over-standard charges and charges for disassembling items;

(4) exchange of medicines, medical consumables, diagnostic and therapeutic items and service facilities;

(5) providing facilities for insured persons to take advantage of the opportunity of enjoying medical security to resell drugs, accept the return of cash or goods in kind or obtain other illegal benefits;

(6) to include medical expenses that are not covered by the medical security fund in the settlement of the medical security fund;

Other illegal acts causing losses to the medical security fund.

Article 39 a designated medical institution shall be ordered by the administrative department of Medical Security to make corrections in any of the following circumstances and may interview the person in charge concerned Violations of other laws and administrative regulations shall be dealt with by the relevant competent authorities in accordance with the law:

(1) no internal management system has been established for the use of the medical security fund, or no specialized institution or personnel is responsible for the management of the use of the medical security fund;

(2) failing to keep financial accounts, accounting vouchers, prescriptions, medical records, records of medical examinations, details of expenses, and records of the entry and exit of drugs and medical consumables into and out of storage as required by regulations;

(3) failing to transmit the relevant data on the use of the medical security fund through the medical security information system in accordance with the provisions;

(4) failing to report the information required for the supervision and management of the use of the medical security fund to the administrative department for Medical Security in accordance with the relevant provisions;

(5) failing to make public the information on medical expenses and fee structure to the public in accordance with the relevant provisions;

(6) providing medical services other than those covered by the medical security fund without the consent of the insured person or his close relatives or guardians, except in special cases such as emergency treatment or rescue;

(7) refusing supervision and examination by administrative departments such as medical security or providing false information.

Article 40 if a designated medical institution defrauded medical security funds by the following means, the medical security administrative department shall order it to return the money and impose a fine of more than two times and less than five times the amount defrauded; Ordering the designated medical institutions to suspend the medical services used by the relevant responsible departments for a period of not less than six months and not more than one year until the service agreement is rescinded by the medical security agencies; if the designated medical institutions are qualified to practice, the competent department concerned shall revoke the practicing qualification in accordance with the law:

(1) inducing or assisting others to seek medical treatment under false names or to purchase medicines falsely, providing false certificates, or colluding with others to falsely issue expense documents;

(2) forging, altering, concealing, altering or destroying medical documents, medical certificates, accounting vouchers, electronic information, etc.

(3) fictitious medical service items;

Other acts of defrauding medical security funds.

For the purpose of defrauding the medical security fund, the designated medical institutions have carried out one of the acts stipulated in article 38 of these regulations and caused losses to the medical security fund, which shall be dealt with in accordance with the provisions of this article.

Article 41 if an individual has one of the following circumstances, the administrative department of Medical Security shall order him to correct it; if he causes losses to the medical security fund, he shall be ordered to return it; if he belongs to a insured person, suspending the settlement of their medical expenses through the network for 3 to 12 months:

(1) handing over one's medical insurance voucher to another for use under an assumed name;

(2) enjoying the treatment of medical security repeatedly

(3) making use of the opportunity to enjoy medical security to resell drugs, accept the return of cash or goods in kind or obtain other illegal benefits.

Individuals who, for the purpose of defrauding medical security funds, have committed one of the acts mentioned in the preceding paragraph, causing losses to the medical security funds, or who use other people's medical security certificates to seek medical treatment or purchase medicines under false names; Or by forging, altering, concealing, altering or destroying medical documents, medical certificates, accounting vouchers, electronic information, or by making up medical service items, in addition to dealing with defrauding medical security fund expenses in accordance with the provisions of the preceding paragraph, the Administrative Department of Medical Security shall also impose a fine of more than two times and less than five times the amount defrauded.

Article 42 where administrative departments such as medical security, medical security agencies, designated medical institutions and their staff accept bribes or obtain other illegal income, the illegal income shall be confiscated, the responsible persons shall be punished according to law; violations of other laws and administrative regulations shall be dealt with by the competent authorities according to law.

Article 43 where a designated medical institution violates the provisions of these regulations and causes major losses to the medical security fund or other serious adverse social effects, its legal representative or principal person-in-charge shall, within five years, be prohibited from engaging in the management activities of designated medical institutions, and shall be punished by the relevant departments according to law.

Article 44 whoever, in violation of these regulations, embezzles or misappropriates the medical security fund shall be ordered by the medical security and other administrative departments to recover it The directly responsible person in charge and other directly responsible persons shall be punished according to law.

Article 45 The returned funds shall be returned to the original special financial account of the medical security fund; the illegal proceeds from fines and confiscation shall be turned over to the state treasury according to law.

Article 46: Administrative Departments such as medical security, medical security agencies, accounting networks and associations and their staff, where a person divulges, tampers with, damages or illegally provides personal information or trade secrets to another person, the person in charge who is directly responsible and other persons directly responsible shall be punished according to law; Those who violate other laws and administrative regulations shall be dealt with by the competent authorities concerned in accordance with the law.

Article 47 staff members of administrative departments such as medical security shall be punished according to law if they abuse their power, neglect their duties or commit malpractices for selfish ends in the supervision and administration of the use of medical security funds.

Article 48 whoever violates the provisions of these regulations and constitutes a violation of the administration of public security shall be given a punishment for public security administration according to law; whoever constitutes a crime shall be investigated for criminal responsibility according to law.

Whoever violates these regulations and causes losses to relevant units or individuals shall be liable for compensation according to law.

Chapter V Annex

Article 49 the supervision and administration of the use of medical security funds such as large medical expenses subsidies for employees and medical subsidies for civil servants shall be carried out by reference to these regulations.

The use of the residents' serious disease insurance funds shall be carried out in accordance with the relevant provisions of the state, and the administrative departments of medical security shall strengthen supervision.

Article 50 these regulations shall come into effect on May 1,2021.

# June 23,2021

# **Notice of the measures for regulating the discretionary power of administrative punishments in the supervision and management of the use of medical insurance funds**

Medical Insurance number 35[2021]

Medical security bureaus of provinces, autonomous regions, municipalities directly under the central government and Xinjiang Production and Construction Corps:

The measures for regulating the use, supervision and administration of the medical security fund and administrative penalty discretion have been considered and adopted by the 46th office meeting of the director of the State Medical Security Bureau on June 9,2021. It is now being printed and distributed to you. Please earnestly follow and implement it.

National Health Insurance Administration

June 23,2021

To standardize the use of medical security funds to supervise and manage the use of administrative penalty discretion

Article 1. In order to regulate the administrative law enforcement activities concerning the use of the medical security funds and to ensure that the medical security administrative departments exercise their discretion in administrative penalties lawfully, reasonably and appropriately, to protect the lawful rights and interests of citizens, legal persons and other organizations, these measures are formulated in accordance with relevant laws and regulations such as the law of the People's Republic of China on administrative penalties and the regulations on the supervision and administration of the use of medical security funds.

Article 2. The term“Discretionary power to supervise and manage the use of medical security funds” as mentioned in these measures means that when the medical security administrative department implements administrative penalties for the supervision and management of the use of medical security funds, in accordance with the provisions of laws, regulations and regulations, taking into account the facts, nature, circumstances, degree of social harm and the subjective fault of the parties involved, the authority to determine the type and extent of administrative punishment.

Article 3 these measures shall be applicable to the administrative departments of provincial-level medical security when formulating administrative penalty discretion benchmarks and exercising administrative penalty discretion.

Article 4 the exercise of discretion in administrative punishment shall comply with the provisions of laws, regulations and rules, follow legal procedures and protect the legitimate rights and interests of the administrative counterpart.

Article 5 the exercise of discretion in administrative punishment shall be in conformity with legal purposes, exclude the interference of irrelevant factors, and adopt necessary and appropriate measures and means.

Article 6 the exercise of discretion in administrative punishment shall be based on facts, and the type and extent of administrative punishment shall be equal to the fact, nature, circumstances and degree of social harm of the illegal act, corresponding to the level of economic and social development of the place where the illegal act takes place.

For the same or similar illegal acts, such as illegal facts, nature, circumstances and consequences of social harm, the types and extent of administrative punishment in the same administrative region shall be basically the same.

Article 7 the provincial-level medical security administrative departments may, in accordance with the unified and standardized measure of discretion for the administrative enforcement of the National Medical Security Fund supervision, formulate a benchmark of discretion for specific medical security fund supervision and administrative punishment.

Article 8 where laws, regulations and regulations provide for discretionary space for matters concerning administrative punishment, the provincial-level administrative departments of medical security shall, in accordance with these measures and in the light of the actual situation of their respective regions, formulate discretionary benchmarks, the standards and applicable conditions of penalty discretion shall be made clear for the regional medical security administrative departments to carry out administrative penalties by reference.

Article 9 the provincial-level administrative departments of medical security shall, in accordance with the formulation of laws, regulations and regulations, changes in the applicable rules of the administrative discretionary power of punishment formulated by higher-level departments, and actual law enforcement work, to revise and improve their own administrative penalty discretion benchmarks in a timely manner and file them with the state medical insurance administration.

Article 10 the following provisions shall be observed in the formulation of the discretionary benchmarks for administrative penalties:

(1) where laws, regulations and rules provide for the possibility of choosing whether or not to impose administrative punishment, the specific discretion standards and applicable conditions for imposing administrative punishment shall be clearly defined;

(2) where laws, regulations and rules provide for the possibility of choosing the types of administrative punishment, the specific discretion standards and applicable conditions for the application of different types of administrative punishment shall be clearly defined;

(3) where a law, regulation or regulation provides that the scope of administrative punishment may be chosen, specific discretion standards and applicable conditions shall be determined on the basis of such factors as the facts, nature, circumstances and degree of social harm of the violation;

(4) where laws, regulations and rules provide that administrative punishment may be imposed either singly or concurrently, the specific discretion standards and applicable conditions of the administrative punishment to be imposed singly or concurrently shall be clearly defined.

Article 11 where the amount of a fine set by laws, regulations or regulations has a certain range, it shall be divided into severe punishment, general punishment and lenient punishment within the corresponding range. Except as otherwise provided for in laws, regulations and regulations, the amount of a fine shall be determined in accordance with the following criteria:

(I) where a fine is an amount of a certain magnitude and a minimum fine and a maximum fine are set at the same time, a lighter penalty shall be less than the median between the maximum fine and the minimum fine, and a heavier penalty shall be higher than the median;

(II) where a fine is a multiple of a specified amount and a minimum and a maximum multiple of the fine are provided at the same time, the lighter penalty shall be less than the intermediate multiple of the minimum and the maximum multiples of the fine, the heavier penalty shall be higher than the intermediate multiple.

Article 12 where there are two or more aggravating circumstances at the same time and there are no mitigating or mitigating circumstances, the punishment shall be imposed at the highest level within the range of punishment corresponding to the illegal act.

If there are two or more mitigating circumstances and no aggravating circumstances, the offender shall be punished according to the lowest level within the range of punishment corresponding to the illegal act.

If there are both aggravating and mitigating circumstances, the corresponding range of punishment shall be determined according to the nature and main circumstances of the illegal act, and the punishment shall be imposed after comprehensive consideration.

The Administrative Department for Medical Security shall not punish any of the following cases:

A minor under the age of 14 who commits an illegal act;

(2) a mentally ill or mentally handicapped person who commits an illegal act when he can not recognize or control his own behavior;

(3) the illegal facts are unclear and the evidence is insufficient

(4) the illegal act is minor and corrected in a timely manner without causing harmful consequences;

(5) if the party concerned has sufficient evidence to prove that there is no subjective fault, and if there are other provisions in laws and administrative regulations, such provisions shall prevail;

(6) if the illegal act is not discovered within two years, no administrative punishment shall be imposed; if it involves the life, health and financial safety of citizens and has harmful consequences, the aforesaid period shall be extended to five years. Unless otherwise stipulated by law.

The period prescribed in the preceding period shall be counted from the date on which the illegal act takes place; if the illegal act has a continuous or continuous state, it shall be counted from the date on which the act ends.

Other cases in which punishment is not given according to law as prescribed by laws, regulations and rules.

The Administrative Department for medical security shall give a lighter or mitigated punishment under any of the following circumstances:

(1) a minor who has reached the age of 14 but not the age of 18 commits an illegal act;

(2) taking the initiative to eliminate or mitigate the harmful consequences of illegal acts committed by the fund;

(3) being coerced or tricked by others into committing an illegal act;

(IV) to make an unsolicited confession of key clues or evidence of the illegal use of the fund that the administrative organ does not yet have, and if it is verified to be true;

(5) actively cooperating with administrative organs in investigating and punishing illegal acts, truthfully stating illegal facts and providing evidence materials on their own initiative;

(6) voluntarily surrenders the case and truthfully confesses the illegal act to the administrative organ;

(7) other circumstances in which the punishment shall be mitigated or mitigated as prescribed by laws, regulations or regulations.

Article 15 the Administrative Department for medical security may give a lighter or mitigated punishment under any of the following circumstances:

(1) any mentally ill or mentally handicapped person who has not completely lost the ability to recognize or control his or her own behavior commits an illegal act;

(2) having violated the law for the first time and having minor harmful consequences and rectifying it in a timely manner;

(3) other circumstances in which the punishment may be mitigated or mitigated as prescribed by laws, regulations or regulations.

The Administrative Department for Medical Security shall impose a heavier punishment in any of the following cases:

(1) the circumstances of the violation are abominable, causing serious harmful consequences;

(2) ordering correction or refusing to do so, or committing more than two illegal acts of the same nature within one year;

(3) obstructing, obstructing or resisting law enforcement officers in investigating and handling their illegal acts according to law;

(4) intentionally transferring, concealing, destroying or fabricating evidence, or retaliating against the complainant or witness who has made a complaint;

(5) other circumstances in which severe punishment shall be imposed as prescribed by laws, regulations and rules.

Article 17 where a mitigated punishment is given, it shall be given below the minimum limit of the statutory administrative punishment according to law.

Article 18 in exercising the discretionary power of administrative punishment, it shall adhere to the combination of punishment with education, law enforcement with law popularization, and integrate law popularization into the whole process of administrative law enforcement, to educate and guide citizens, legal persons or other organizations to learn and abide by the law conscientiously.

Article 19. The administrative departments for medical security shall strengthen the collection, collation, research and publication of typical cases for supervision and law enforcement of medical security funds, and establish a database of cases for supervision and law enforcement of medical security funds, give full play to typical cases in the guidance and norms of administrative punishment discretion work in the guidance and norms of the function.

The Administrative Department of Medical Security shall not exercise discretion in administrative punishment under the following circumstances:

(1) the facts, nature, circumstances and degree of social harm of the illegal act are abnormally light or heavy in comparison with the administrative punishment received;

(2) in cases of the same type in the same period, where the illegal acts of different parties are the same or similar and the administrative penalties imposed vary considerably;

(3) where an administrative penalty shall not be imposed or mitigated or mitigated according to law, the administrative penalty shall be imposed or not mitigated or mitigated;

(4) other cases of abuse of the discretionary power of administrative punishment.

Article 21 the administrative departments for medical security at all levels shall establish and improve a supervisory system to standardize the discretion of the medical security funds in supervising administrative penalties, to strengthen supervision over the exercise of discretionary power by the administrative departments of medical security within their respective administrative areas through the following means:

(1) collective discussion on administrative punishment

(2) legal review of administrative penalty decisions

(3) evaluation and assessment of administrative law enforcement;

(4) review of administrative penalty files;

(5) handling complaints and reports on administrative law enforcement;

(6) the results of the administrative penalty shall be made public;

(7) other means prescribed by laws, regulations and rules.

Medical security administrative departments at all levels shall strengthen supervision and guidance over the exercise of administrative penalty discretion by medical security administrative departments at lower levels. If it discovers that the administrative penalty discretion is illegal or improper, it shall promptly correct it.

Article 22 where a medical security fund supervises an administrative law enforcement official who abuses the discretion of administrative punishment, his administrative responsibility shall be investigated according to law. Those who are suspected of violating discipline or committing crimes shall be handed over to the discipline inspection and supervision organs and judicial organs for handling according to law and regulations.

Article 23 the administrative penalty discretion benchmarks formulated by the provincial-level medical security administrative departments shall be made public to the public in a timely manner.

Article 24 these measures shall come into effect on July 15,2021.

# November 26,2021

# **Notice on strengthening the work of bridging execution and punishment in investigating and dealing with cases of fraudulent use of medical insurance funds**

Medical Insurance [2021] No. 49

Medical security bureaus and public security bureaus of provinces, autonomous regions, municipalities directly under the central government and Xinjiang Production and Construction Corps:

In order to improve the linkage mechanism between administrative law enforcement and criminal justice, strengthen the cooperation between the administrative departments of medical security and public security organs, punish the crime of defrauding medical insurance funds according to law, and ensure the safety of medical insurance funds, safeguarding the legitimate rights and interests of the people participating in the insurance program, and promoting social integrity and the rule of law, in accordance with such laws and regulations as the criminal law of the People's Republic of China, the Social Insurance Law of the People's Republic of China, the regulations on the supervision and administration of the use of medical security funds, and the provisions on the transfer of suspected criminal cases by administrative law enforcement agencies, the following is the notice on the joint work of strengthening the investigation and handling of cases of defrauding medical insurance funds for execution:

1. Strengthen the joint work of investigating and handling cases of defrauding medical insurance funds

Medical security administrative departments and public security organs at all levels should adhere to the people-centered development thought, implement the criminal justice policy of combining leniency with severity, and strengthen the effective link between the administrative law enforcement of medical insurance fund supervision and criminal justice, we will effectively transfer and accept cases. Medical insurance departments and public security organs at all levels should, in accordance with their functions and powers, do a good job of investigating, transferring, filing, investigating, investigating and prosecuting cases of defrauding medical insurance funds, so as to remove as much as possible and receive as much as they can, no administrative punishment may replace criminal accountability.

2. Clearly investigate and deal with cases of defrauding medical insurance funds

In the course of supervising and enforcing the law on medical insurance funds, administrative departments of medical insurance at various levels have discovered that citizens, legal persons and other organizations have committed acts listed in the“Cases of defrauding medical insurance funds for transfer”(see Annex 1 for details) and are suspected of committing crimes, shall be transferred to the public security organs at the corresponding levels in accordance with the law.

Third, standardize the procedures for transferring cases of fraudulent medical insurance funds

(1) transfer. In the case of medical security administrative departments transferring cases of defrauding medical insurance funds, no less than two administrative law enforcement personnel shall be determined to form a special task force, and after verifying the situation, a written report on the transfer of suspected criminal cases shall be submitted, report to the head of the department or the person in charge of the work for examination and approval. The head of the department or the person in charge of the work shall, within 3 days from the date of receiving the report, make a decision to approve or disapprove the transfer. If it decides to approve, it shall be transferred to the public security organ at the same level within 24 hours; if it decides not to approve, the reasons for not approving shall be recorded in the record.

(2) transfer of materials. When transferring a case, the following materials shall be attached: “Letter of transfer of suspected criminal cases”(see Annex 2 for details) , the case investigation report (see Annex 3 for details) , the list of items involved in the case and the relevant documentary evidence, physical evidence, test report or identification conclusion, as well as other materials related to the suspected crime are enclosed.

Where a decision on administrative punishment has been made at the time of the transfer of the case, the decision on administrative punishment shall be copied together.

(c) accepting a case. The public security organ shall accept the case of defrauding the medical insurance fund transferred by the Administrative Department of medical security, and sign the“Transfer letter of suspected criminal cases (receipt)”(see Annex 4 for details) . If the public security organ considers that the case materials transferred by the medical security administrative department are incomplete, it shall, within 24 hours after accepting the case, notify the medical security administrative department transferred to correct it within 3 days, however, they shall not refuse to accept the transfer of a case on the ground that the materials are incomplete. If the public security organ considers that the case transferred by the medical security administrative department does not fall within its jurisdiction, it shall, within 24 hours, transmit the case to the competent organ and inform the medical security administrative department that transferred the case in writing.

The public security organ shall, from the day it accepts a case, file a case for examination, and the time limit for filing a case for examination shall not in principle exceed three days, and where a suspected criminal clue needs to be verified, the time limit for filing a case for examination shall not exceed seven days, with the approval of the person in charge of the public security organ at the county level or above, the time limit for filing and examining major, difficult and complicated cases may be extended to 30 days. Those who believe that a crime has been committed shall be held criminally responsible and file a case in accordance with the law. The public security organ shall, within 3 days from the date of making the decision to file a case or not file a case, inform in writing the medical security administrative department that transferred the case. If it decides not to file a case, it shall explain in writing the reasons for not filing a case and return the file materials. If the medical security administrative department has any objection to the decision of the public security organ not to file a case, it may, within three days after receiving the notice, file a reconsideration with the public security organ that has made the decision not to file a case, it may also propose to the procuratorial organ that it supervise the filing of a case in accordance with the law.

4. Improve the coordination mechanism for investigating and handling cases of defrauding medical insurance funds

1. Deepening cooperation in the investigation and handling of transferred cases. Administrative Departments of medical security at all levels and public security organs shall establish a mechanism of liaison for the coordination of execution, so as to coordinate the investigation and punishment of transferred cases. The administrative department of Medical Security shall promptly provide relevant medical insurance information, supporting materials and policy basis to the public security organ for cases that should be transferred; In cases of possible flight, transfer of funds and destruction of evidence, the public security organs shall be informed in a timely manner, and the public security organs shall assist the medical security administration in taking urgent measures. If necessary, both parties shall coordinate to accelerate the transfer process, take emergency measures in accordance with the law. The public security organs should step up their efforts to investigate and deal with cases of defrauding medical insurance funds, promptly recover medical insurance funds used in violation of regulations and return them to the Special Medical Insurance Fund account, those who manipulate the organizations behind the scenes, key members, professional card collectors and professional drug traffickers should be severely punished in accordance with the law, and those who commit first or occasional crimes that do little harm to society and are not deeply involved in the case should be given a lighter punishment, medical personnel and patients who plead guilty to crimes and punishment should be given leniency in accordance with the law.

(2) establishing a system of joint meetings and briefings. Medical security administrative departments and public security organs at all levels shall regularly hold joint meetings to exchange information on the investigation and punishment of cases of defrauding medical insurance funds and on the work of linking administrative law enforcement with criminal justice, by constructing a real-time analysis and early-warning monitoring model, analyzing the situation and tasks of the crime of defrauding medical insurance funds, coordinating and solving the problems in the work, and studying and putting forward measures to strengthen the prevention, investigation and punishment, find clues to crimes committed by defrauding health insurance funds in a timely manner, and organize inspections according to authority. To strengthen information briefing, through work briefings, information networks and other forms, timely notification and exchange of relevant information, information sharing.

Improve the case management and reporting system. Medical security administrative departments and public security organs at all levels should establish a standardized and effective case management system, strengthen case follow-up and supervision and summary reporting, and regularly report cases of defrauding medical insurance funds to higher-level departments. We will improve the mechanism for recording and applying information on crimes committed by units and individuals who defraud medical insurance funds, and promote social integrity. We will strengthen the analysis of typical cases of crimes committed by fraudulently obtaining medical insurance funds, summarize and grasp the law and characteristics of cases, strengthen professional training, and constantly improve our ability to investigate and handle cases and the level of law enforcement. The administrative departments of medical security and public security organs shall supervise and inspect the implementation of this notice by the administrative departments of medical security and public security organs at lower levels, and regularly conduct spot checks on the investigation and handling of cases, and promptly correct any problems and deficiencies in the transfer of cases.

4. Strengthen consultation on the investigation and handling of major cases. The major cases transferred by the public security organs to the administrative departments of medical security should be solved quickly by concentrating their superior police forces and using a variety of investigative methods. For cases with complex circumstances and great social impact, special forces should be organized to investigate and solve the cases, and the results should be announced to the public in due course. We should strengthen case consultation, handle cases in strict accordance with the law, and strictly distinguish crime from non-crime according to legal duties, powers and procedures.

Job requirements

Strengthen organizational leadership. Medical security administrative departments and public security organs at all levels should attach great importance to the joint work of investigating and handling cases of defrauding medical insurance funds, further improving the political position, strengthening the organization and leadership, improving the working mechanism, and clarifying the division of duties and responsibilities, they should press down on work responsibilities, strengthen supervision and assessment, and work hard to implement them. To do a good job in transferring, accepting, placing on record, investigating and dealing with cases, and other links to form a joint force, according to the law to crack down on defrauding medical insurance funds illegal criminal acts, we must protect the people's“Money for medical treatment and life-saving services.”.

(2) to carry out listing supervision. The Ministry of Public Security and the state medical security administration will exercise“Double listing” supervision over the investigation and handling of major cases. The Ministry of Public Security and the State Medical Security Bureau shall be responsible for determining the degree of importance and urgency for listing and supervising cases, and strengthening case supervision and notification. Public security organs at all levels and administrative departments of medical security shall implement the general responsibility of the principal person-in-charge in order to ensure the timely completion of cases. If there are difficulties, they should promptly report them to the Ministry of Public Security and the State Medical Security Bureau for Adjustment. The Ministry of Public Security and the State Medical Security Bureau shall organize and verify whether the case is completed, and those who fail to complete the case within the time limit shall be notified and criticized.

(3) step up publicity. We should strengthen the policy of investigating and punishing fraudulent medical insurance funds, encourage the whole people to participate in supervision, actively report fraudulent medical insurance funds illegal crimes. We will improve the process for handling clues in reports, implement reward measures for reports, and reward fast rewards in accordance with the law and regulations. We will strictly enforce the confidential reporting system, protect the legitimate rights and interests of informants, and create a good atmosphere of social concern, participation and support for fund supervision. We should step up efforts to expose cases of crimes committed by defrauding medical insurance funds, do a good job in publicizing and guiding public opinion, and play an effective role in warning and education, so as to better punish crimes and deter criminals.

This work is organized and directed by the Fund Supervision Department of the State Medical Security Bureau and the Criminal Investigation Bureau of the Ministry of Public Security.

Annex: 1. Cases of fraudulent transfer of health insurance funds

A letter of referral for a suspected crime

Investigation report

4. Letter of transfer (acknowledgement) for suspected criminal cases

State health insurance administration

Ministry of Public Security

November 26,2021

# January 29,2022

# **Interim Measures for supervision and management of the use of medical insurance funds and reporting**

Decree no. 5 of the State Medical Security Administration

The interim measures for handling reports on the supervision and management of the use of the medical security fund, which was considered and adopted at the 4th Bureau meeting on January 20,2022, are hereby promulgated and shall come into effect on March 1,2022.

Director: Hu Jinglin

January 29,2022

Interim measures for the handling of medical insurance fund reports through supervision and administration

Article 1 to regulate the use of medical security funds, supervise and manage the handling of reports, ensure timely and effective handling of reports, and safeguard the safety of medical security funds, to protect the lawful rights and interests of natural persons, legal persons or other organizations, in accordance with relevant laws and administrative regulations, such as the law of the People's Republic of China on social insurance and the regulations on the supervision and administration of the use of medical security funds, formulating these measures.

Article 2 these measures shall apply to the handling of reports on illegal and illegal use of basic medical insurance (including maternity insurance) funds, medical assistance funds and other medical security funds.

As mentioned in these measures, refers to the act of a natural person, legal person or other organization (hereinafter referred to as the informant) reporting to the administrative department of Medical Security that the informant is suspected of violating the laws, regulations and rules on the supervision and administration of the use of medical security funds.

Article 3 the Administrative Department of Medical Security under the State Council shall be in charge of the handling of reports throughout the country, and shall guide the handling of reports by local administrative departments of medical security. Administrative Departments of medical security at all levels shall establish and improve the working mechanism for handling reports.

The administrative departments of medical security at or above the county level shall be responsible for the handling of reports within their respective administrative regions. Where there are other provisions in laws, administrative regulations or departmental rules, such provisions shall be followed.

Article 4 the Administrative Departments of medical security shall follow the principles of unified leadership, territorial administration, hierarchical responsibility, impartiality and efficiency in handling reports, and ensure that the basis of application is correct and the procedure is lawful.

Article 5 the public and the news media shall be encouraged to exercise social and public opinion supervision in accordance with the law on illegal and illegal acts suspected of violating the supervision and administration of the use of medical security funds.

Article 6 a report to the administrative department of Medical Security shall be made through the internet, telephone, fax, postal address and other channels for receiving the report as announced by the Administrative Department of Medical Security. Where a medical security agency or other department receives a tip-off clue that should be handled by the medical security administrative department according to law, it shall be handed over to the medical security administrative department for handling.

The administrative departments of medical security at all levels shall unblock the reporting channels and strengthen the professionalization and integration of the reporting channels.

Article 7 the informant shall provide specific clues on the suspected violation of the laws, regulations and rules on the supervision and administration of the use of the medical security fund. If the informant reports in a non-written manner, the staff member of the Administrative Department of Medical Security shall record the report.

Article 8 The informant may report the case in real name or anonymously. If the informant makes a report under his real name, the informant shall provide his true identity information and the true and effective contact method. Real-name informants are encouraged to report, and the administrative departments of medical security shall, in accordance with the requirements of these measures, carry out relevant notification procedures and strictly keep confidential the information of real-name informants.

The Administrative Department of Medical Security Shall Register the reports received.

Article 9 a report shall be handled by the administrative department for medical security at or above the county level where the reported act took place. Where there are other provisions in laws, administrative regulations or departmental rules, such provisions shall be followed.

If the administrative department of Medical Security that receives the report does not have the authority to handle it, it shall inform the informant to submit the report directly to the administrative department of Medical Security that has the authority to handle it.

If the administrative department of Medical Security at a lower level considers it necessary for the administrative department of Medical Security at a higher level to handle a report, it may report it to the administrative department of Medical Security at a higher level for a decision; if the administrative department of Medical Security at a higher level considers it necessary, it may handle reports received by the lower medical security administrative department.

Article 10 where a dispute arises over the handling authority of two or more administrative departments of medical security, the dispute shall be resolved through consultation within 7 working days from the date of the dispute; if consultation fails, report to the Administrative Department for medical security at the same higher level for designation of the handling department.

Article 11 the administrative departments for medical security at or above the county level shall uniformly receive the working agencies for reporting, the report shall be promptly transmitted to the medical security administrative department at the lower level or to the relevant organ of the medical security administrative department at the same level for processing.

The relevant organs of the administrative departments of medical security at the same level shall, upon receipt of the reports transmitted, handle them in a timely manner in accordance with the relevant provisions of these measures. If it does not have the authority to handle the report, it shall give timely feedback to the working organization that receives the report, and may not transfer the report by itself.

Article 12 the Administrative Department for Medical Security shall handle the report in accordance with the relevant provisions such as administrative penalties for medical security.

If the informant reports the case in real name, the medical security administrative department with the authority to handle the case shall inform the informant within 5 working days from the date of making the decision on whether to file the case.

Article 13 where laws, regulations and regulations stipulate that the administrative department of Medical Security shall inform the informant of the results of the handling of the report, the Administrative Department of Medical Security shall inform the informant.

Article 14. Before the administrative department of Medical Security makes a decision on the handling of a reported matter that has already been filed, if the informant voluntarily withdraws the report, the investigation and handling by the administrative department of Medical Security shall not be affected; The Administrative Department of Medical Security shall no longer inform the informant of the outcome of the handling of the report.

Article 15 the informant shall cooperate with the administrative department for medical security in the investigation according to law.

Article 16 the Administrative Department of Medical Security shall keep confidential the information of the informant and shall not divulge the personal information of the informant or the information on the handling of the report to the informant or to persons unrelated to the handling of the report.

The administrative departments of medical security shall strictly keep confidential the state secrets that they have learned during the handling of the report, as well as the information that may endanger national security, public security, economic security and social stability if it is made public.

Information concerning trade secrets and personal privacy shall be governed by the regulations of the government of the People's Republic of China on the disclosure of information.

Article 17 the Administrative Departments of medical security shall strengthen the statistics, analysis and application of the reporting information in their respective administrative areas, and regularly publish the statistical analysis reports on the reporting.

Article 18 the informant shall be responsible for the authenticity of the contents of the report and the materials provided by the informant. Those who fabricate or distort the facts, falsely accuse and frame others, shall bear relevant legal responsibilities according to law.

Article 19. The administrative department of Medical Security under the State Council shall establish an annual report system for the handling of reports. Each provincial-level administrative department of Medical Security shall, before April 30 of each year, before April 30 of each year. In case of major issues, the provincial-level administrative departments for medical security shall report to the administrative department for Medical Security under the state council in a timely manner.

Article 20. The administrative departments of medical security shall make known to the public the verified typical cases which have a major social impact, but where state secrets, work secrets, business secrets and individual privacy are involved, in accordance with the“Government of the People's Republic of China's regulations on the disclosure of information” and other relevant provisions.

Article 21 the Administrative Department of Medical Security shall, upon verification, reward reports that meet the conditions for reporting rewards.

Article 22 the Administrative Department of Medical Security shall, within 5 working days after the completion of the work, file and file the relevant materials involved in the process of handling the report in accordance with the relevant archives management regulations, and keep the files for future reference.

Article 23 the Administrative Departments for medical security at all levels shall be staffed with professionals and provide necessary office premises and equipment to ensure the smooth receipt and handling of reports.

Article 24 the handling of reports on illegal use of medical security funds, such as residents' serious disease insurance, large medical expenses subsidies for employees and medical subsidies for civil servants, shall be carried out by reference to these measures.

Article 25 these measures shall not be applicable to the conduct of consultation, application for publication of Government Information, application for administrative reconsideration, letters and visits, etc. in the form of reports, the administrative department of medical security may inform and submit the application through the corresponding channels.

Article 26 these measures shall go into effect on March 1,2022.

# November 17,2022

# **Notice of“Illegal use of medical insurance fund reporting incentives”**

Medical insurance bureaus and finance bureaus of provinces, autonomous regions, municipalities directly under the central government and Xinjiang Production and Construction Corps:

In order to further adapt to the new situation of fund supervision, continuously strengthen the role of social supervision, and jointly maintain the safety of medical security funds, the State Medical Insurance Bureau and the Ministry of finance jointly formulated the“Illegal use of medical security fund report incentives”, is now printed and distributed to you, please implement it carefully.

Office of the state medical insurance administration

Office of the Ministry of Finance

November 17,2022

(voluntary disclosure)

Measures for rewarding illegal and illegal use of medical security funds

Article 1

In order to encourage the reporting of illegal and illegal use of medical security funds, to mobilize social forces to participate in the supervision of medical security funds, and to safeguard the safety of medical security funds and the legitimate rights and interests of citizens in medical security, according to the Social Insurance Law of the People's Republic of China,

these measures are formulated in accordance with such laws, regulations and regulations as the interim measures on social assistance, the regulations on the supervision and administration of the use of medical security funds and the interim measures on the handling of reports on the supervision and administration of the use of medical security funds

Number Two

A natural person (hereinafter referred to as a whistle-blower) shall report to the administrative department of medical security the suspected illegal and illegal use of basic medical insurance (including maternity insurance) funds, medical assistance funds, and other medical security funds and provide relevant clues, if it is verified and should be rewarded, these measures shall be applied to those cases where the administrative department for medical security entrusts organizations such as medical security agencies to carry out the work of handling reports.

Rewards for illegal use of medical security funds, such as residents' serious disease insurance, employees' large medical expenses subsidies, and civil servants' medical subsidies, shall be implemented by reference to these measures

Article 3

The rewards for reporting shall follow the principles of protecting the legitimate rights and interests of the informant according to law, voluntary collection and appropriate rewards.

The rewards for informants shall also meet the following conditions:

Article 4

(1) having a clear target to be reported and specific clues for violating laws and regulations, and providing effective evidence;

(2) the main facts and evidence of the report were not in the possession of the medical security department in advance; (3) the reported matter was verified to be true, and the reported act has caused losses to the medical security fund; (4) the informant is willing to receive a reward for the report, and provide real and effective identity information and contact information for verification; (5) other necessary conditions for rewards according to laws and regulations;

Article 5

In any of the following circumstances, no reward shall be given: (1) the informant shall be a staff member of the Medical Security Department or a staff member of a third-party institution entrusted by the Ministry of Medical Security to perform the functions of fund supervision; (2) any person who illegally uses the medical security fund shall, on his own initiative, confess the facts of his or her violation of the law and regulations as well as those of his or her co-workers, or report and expose his or her violation during the period of investigation and handling; (3) before the administrative department for Medical Security makes a decision on handling the matter reported, the informant takes the initiative to withdraw the report;

(4) the informant can not be identified or contacted;

(5) before the report, the relevant illegal and illegal use of the medical security fund has entered legal procedures such as litigation and arbitration;

(6) other cases in which rewards are not granted according to law and regulations

Article 6

The administrative department of Medical Insurance shall give a one-off financial reward to the informant who meets the reward conditions according to a certain proportion of the value of the case, the maximum amount of which shall not exceed 200,000 yuan, and the minimum amount of which shall not be less than 2 million yuan.

Article 7 the funds required for reporting rewards shall be incorporated into the budgets of administrative departments for medical security at the county level and above.

Article 8 the Administrative Departments of medical security that handle the reports shall be responsible for issuing the rewards for reports.

Article 9. Rewards shall be given to those who report multiple reports in accordance with the following rules:

(1) if the informant makes multiple reports on the same illegal and illegal use of the medical security fund, the reward shall not be issued repeatedly;

(2) if two or more informants report the same illegal and illegal use of the medical security fund, and the content of the report and the clues provided are basically the same, rewards the first informant;

(3) if two or more informants jointly make a report, they shall be deemed to be the same informant and will be rewarded

Article 10 a whistle-blower shall, within 2 months from the date of receiving the notice of receiving the reward, receive the reward on the basis of his valid identity certificate. In case of entrustment, the trustee shall hold simultaneously the power of attorney of the informant, the valid identification of the informant and the trustee.

If the informant fails to collect the reward within the time limit, it shall be deemed to have voluntarily given up. A joint informant shall nominate a representative to receive the reward,

Self-allocated internally.

Article 11 the Administrative Department of Medical Security shall open up convenient channels for payment so as to facilitate the informant to receive the report reward funds. In principle, the reward funds for reporting shall be paid by non-cash means and shall be handled in accordance with the provisions on centralized payment by the state treasury.

Article 12 the Administrative Department of Medical Security shall strictly examine and verify the granting of reward funds for reporting crimes. Having discovered that the administrative department of medical security that issued the reward has the right to withdraw the reward for reporting a crime after verification that the reward was obtained by fraudulent means such as falsifying materials or concealing facts, or that there were other circumstances that did not meet the requirements for receiving the reward, and investigate the relevant responsibilities of the parties in accordance with the law.

Article 13 the case value as mentioned in these measures refers to the amount of loss of the medical security fund that should be recovered in respect of the reported matters. Except for the items reported, the amount of other illegal violations verified shall not be included in the calculation of the case value.

Article 14 the administrative departments and financial departments at the provincial and municipal levels of medical security may, in accordance with these measures, formulate detailed rules for implementation and make specific provisions on the standards and procedures for awarding awards.

Article 15

These measures shall be interpreted by the state medical insurance bureau and the Ministry of Finance and shall be implemented as of January 1,2023. Circular of the office of the State Medical Security Bureau and the General Office of the Ministry of Finance on the issuance of the interim measures on reporting and rewarding acts of defrauding and obtaining medical security funds

(issued by the Medical Insurance Office [2018] No. 22) shall be abolished at the same time.

# May 27,2022

# **Notice on launching flight inspection of Medical Insurance Fund in 2022**

Medical Insurance Letter [2022] No. 24

The medical insurance bureaus, finance bureaus, health committees and Chinese medicine bureaus of all provinces, autonomous regions, municipalities directly under the central government and the Xinjiang Production and Construction Corps:

In order to fully implement the decisions and plans of the CPC Central Committee and the State Council on strengthening the supervision of medical insurance funds, crack down on illegal and illegal acts in the field of medical insurance, earnestly safeguard the safety of medical insurance funds, and protect the health rights and interests of citizens, the State Medical Insurance Bureau, the Ministry of Finance, the State Health Commission and the state administration of traditional Chinese medicine have decided to organize a nationwide medical insurance fund flight inspection in 2022, and formulated the“2022 Medical Security Fund Flight Inspection Work Program”, is now printed and distributed to you. Please do a good job according to the plan, such as personnel selection, organization, implementation, rectification and other work, a solid and orderly completion of the inspection task.

National Health Insurance Administration, Ministry of Finance

National Health Commission, State Administration of traditional Chinese medicine

May 27,2022

2022 Medical Security Fund Flight Inspection Work Programme

In order to thoroughly implement the decisions and plans of the CPC Central Committee and the State Council, in accordance with the guiding opinions of the State Council General Office on promoting the reform of the regulatory system for medical security funds -No.-no. 20 of [2020]) , we have innovated and improved fund supervision methods, consolidated the high-pressure trend of combating fraud and insurance fraud, strictly observed the health insurance fund safety Red Line, and now decided to organize a nationwide medical insurance fund flight inspection in 2022.

First, the work goal

Guided by Thought on Socialism with Chinese Characteristics for a New Era, we will fully implement the spirit of the 19th National Congress of the Communist Party of China and all plenary sessions of the 19th National Congress of the Communist Party of China, adhere to people's health as the center, focus on key points, and make targeted efforts to investigate and deal with violations of laws and regulations in the field of medical insurance in accordance with the law. Effectively safeguard the safety of medical insurance funds, protect the legitimate rights and interests of citizens in medical insurance, and improve people's health and well-being.

Check the object

Nationwide designated medical institutions, county and district-level medical insurance agencies, depending on the situation can be extended to check the relevant institutions and the insured. As a matter of principle, institutions that have previously received national flight inspections will no longer be subject to such inspections.

The time frame for inspection is January 1,2020 onwards.

Check the content

For the medical service behaviors and medical expenses covered by the medical security fund in the fields of hemodialysis in designated medical institutions, high-value medical consumables (orthopaedics, Cardiology) and other fields (including the settlement expenses of local personnel receiving cross-provincial medical treatment) , health insurance agencies shall conduct checks on the performance of service agreements, the verification and settlement of expenses, and the defrauding of health insurance funds by means of falsifying medical-related information and making up medical service items.

(1) targeted inspections at designated medical institutions. Including the internal management of the use of funds, financial management, medical records and related information management, management of the purchase, sale and storage of drugs and medical supplies, and the behavior of disassembling hospitalization, sickbed hospitalization, violating the standard of diagnosis and treatment, illegal charges (including illegal charges for detection of covid-19 nucleic acid and antigen) , swapping items, illegal procurement of off-line drugs, failure to purchase and use the selected products collected by the state organization according to the requirements, etc. .

(2) the examination of medical insurance agencies. Including the registration and direct settlement of off-site medical treatment, the treatment of out-patients with chronic special diseases, manual reimbursement, and the cost of medical institutions audit and settlement of payments, the fund“Income and expenditure two lines” implementation and accounting, verification of insured persons' enjoyment of medical insurance benefits and the implementation of agreements with designated medical institutions.

Composition of personnel

The state medical insurance administration, together with the Ministry of Finance, the State Health Commission, the state administration of traditional Chinese medicine and other departments, will jointly organize a number of flight inspection teams to carry out flight inspections, and the team leader system will be implemented, the number of people in each group should be limited to 40.

(1) inspection staff (about 30 persons) .

1. Team leader (1 person) . The leaders in charge of fund supervision or audit and audit of the medical insurance bureaus of the participating provinces shall be in charge of the flight inspection work.

2. Deputy team leader (about 4 persons) . A department-level cadre familiar with fund supervision or auditing and auditing shall be appointed by the medical insurance bureaus of the participating provinces. Departments of finance, health and traditional Chinese medicine may also designate a department-level cadre each to act as a deputy team leader, to assist the team leader in conducting the flight inspection.

3. Professionals (about 25 persons) . The provincial health insurance bureaus in the participating provinces have drawn about 15 business backbones from local administrative and management organizations, and a total of about 10 medical experts and staff members from departments of Finance, health and traditional Chinese medicine, responsible for on-site inspection, investigation and evidence collection, communication and feedback, clue handover, etc. .

(2) Inspector Liaison Officers (about 3 persons) .

1. Inspectors (1-2) . The state medical insurance administration and the departments and departments concerned shall be responsible for supervising and instructing the flight inspection team to carry out the inspection work in accordance with the law and regulations, and to study and decide on major matters of flight inspection.

2. Liaison Officers (1-2 persons) . Officials of the state medical insurance administration (NHS) are responsible for coordinating the conduct of flight inspections.

(3) personnel of third-party organizations (about 7 persons) .

Medical, pharmaceutical, financial and information professionals selected by third-party organizations shall be responsible for data screening and analysis.

Fifth, organization and implementation

The provinces were selected by drawing lots, and the cross-matching groups were selected from Beijing, Tianjin, Shanghai and Chongqing. In addition, the state medical insurance bureau organized teams to carry out special flight checks on relevant agencies according to the work needs. The duration of each group is limited to 10 days. As appropriate, invite the news media to participate in the publicity.

Preparation for work.

1. Identify the district. The flight inspection team and the Health Insurance Bureau of the province under investigation to determine the city under investigation, but also on the basis of reporting problems clues, intelligent monitoring data and other suspicious designated.

2. Develop an implementation plan. Lead by the team leader, the program should be clear about the content of the inspection, inspection methods, personnel groups, implementation steps.

3. Strengthen data analysis. According to the key points of examination, the data of medical insurance settlement and HIS system were extracted in advance, and the pre-screening analysis was carried out.

Four. Conduct mobilization training. Before the on-site inspection, organize all the inspectors to familiarize themselves with the local medical insurance policies, and train them on law enforcement procedures, inspection focus, division of duties, work discipline, etc. , ensure inspections are standardized, standardized and refined.

(2) on-site inspection.

1. Identify the subject. According to the scale of fund expenditure, one or two designated medical institutions and one medical insurance agency were selected at random or combined with relevant clues, and announced at the launch meeting of the flight inspection.

2. Conduct on-site inspections. After reading and serving the notice of on-site inspection to the inspected institution, the on-site inspection shall be carried out according to the implementation plan.

3. Perform feedback handover. On the basis of fully listening to and judging the opinions of the institution and the District Medical Insurance Bureau, objective and impartial written feedback is formed. At the same time, the completion of data collection and cleaning, and the inspection of data transferred to the examined province medical insurance bureau for follow-up verification processing.

(3) rectification and implementation.

Within 30 working days after receiving written feedback and transferring data, the examined provincial medical insurance bureau will report the rectification to the state medical insurance bureau in Written Form, and do a good job of follow-up processing and exposure. The state medical insurance bureau will inform the relevant departments about the work of the flight inspection and, depending on the situation, make public the results of the investigation and punishment. According to the reform of the provinces or work needs, the National Health Insurance Bureau can timely organize forces to carry out“Look back.”.

Job requirements

(1) perform their duties and duties with due diligence, and tamp down the principal responsibilities. Departments at all levels, including medical insurance, finance, health care and traditional Chinese medicine, should consciously enhance their political awareness, strengthen their responsibility for the mission, earnestly perform their supervisory duties, and strictly fulfill their inspection tasks as required. The relevant departments in the provinces under inspection should attach great importance to it and make good preparations for the inspection. At the same time, they should press down on the responsibility of compacting and rectifying, determine the precise rectification measures, and focus on strengthening the governance at source, we will accelerate the construction of local regulatory systems and mechanisms with the aid of flight inspections and comprehensively improve the management of medical insurance funds.

(2) coordinate and efficiently, and inspect in accordance with laws and regulations. Relevant departments should strengthen cooperation and coordination, make full use of the advantages of multi-department joint law enforcement, form joint supervision efforts, make the problem qualitative, and push for effective flight inspection. At the same time, strictly regulate the law enforcement behavior, for the problems found in the inspection, to find out, and do a reasonable basis to ensure that the facts of the problem is clear, conclusive evidence.

(3) to exercise strict discipline and do a good job in epidemic prevention and control. The inspectors shall conscientiously implement the Central Committee's eight provisions and the spirit of its implementation rules, and strictly abide by the provisions of law enforcement, security, confidentiality, integrity and so on. It shall not affect the normal working order of the institutions under examination, and it is strictly forbidden to make use of work facilities to make life difficult for the objects under examination, or to accept the objects' property and banquets, etc. . Flight inspection will be timely organized according to the needs of epidemic prevention and control, and by the relevant departments to make arrangements for unified deployment. Inspection staff should strictly implement the responsibility of epidemic prevention and control, to comply with the territorial requirements of prevention and control, if there are symptoms of discomfort, timely medical treatment, not to conceal the condition.

# April 21,2023

# **Interim Measures for the management of flight inspection of the medical insurance fund**

It was deliberated and adopted at the 5th Bureau meeting on 14 February 2023, and is hereby announced for implementation as from 1 May 2023.

Director: Hu Jinglin

March 13,2023

Interim measures on the administration of flight examinations for the medical insurance fund

Chapter I general

Article 1 in order to strengthen the supervision and inspection of the medical security fund and standardize the work of flight inspections, these measures are formulated in accordance with the provisions of the Social Insurance Law of the People's Republic of China, the regulations on the supervision and administration of the use of medical security funds and other relevant laws and regulations.

Article 2 the Medical Security Fund flight inspection mentioned in these measures (hereinafter referred to as flight inspection) refers to the organization and implementation by the state and provincial medical security administrative departments, on-site supervision and inspection without prior notice to the inspected objects such as designated medical institutions, medical insurance agencies and other organizations undertaking medical insurance business.

Article 3 the principles of seeking truth from facts, fairness, civilization and lawful procedures shall be observed in flight inspections.

Article 4 the State Administrative Department for Medical Security shall be responsible for organizing and implementing flight inspections throughout the country.

The provincial administrative departments for medical security shall be responsible for organizing and implementing flight inspections within their respective administrative areas. Joint and cross-provincial air inspections conducted by provincial-level medical security administrative departments shall be filed with the National Medical Security Administrative Departments before the commencement of the inspection.

Article 5 the Administrative Department of Medical Security shall establish a communication mechanism with the relevant departments of finance, health, market supervision, traditional Chinese medicine and so on, strengthen coordination and cooperation, and May, when necessary, jointly carry out flight inspections with relevant departments.

The administrative department of medical security may employ qualified information technology service agencies, accounting networks and associations, commercial insurance agencies and other third-party organizations and professionals to assist in conducting flight inspections.

Article 6 the Administrative Department of Medical Security shall establish a database of objects to be examined, personnel to be examined and experts to be dynamically managed.

Article 7 the Administrative Departments of medical security shall strengthen capacity-building for flight inspections and, in accordance with the relevant provisions of the state, allocate necessary inspection equipment and law enforcement and evidence collection equipment for flight inspections, so as to improve the quality and efficiency of flight inspections.

Article 8 the Administrative Department of Medical Security shall accept the supervision of the work of flight inspection by all sectors of society on its own initiative.

Article 9 the personnel participating in the flight inspection shall abide by laws and regulations and strictly enforce the provisions on confidentiality, avoidance and honesty.

Chapter two starts

Article 10 in any of the following circumstances, the administrative department of medical security may initiate a flight inspection:

(1) the annual work plan is arranged

(2) where the reporting clues indicate that the medical security fund may have significant security risks;

(3) the medical insurance intelligent monitoring or big data screening indicates that the medical insurance fund may have significant security risks;

(4) the exposure of the news media has caused major social impacts

(5) other circumstances requiring flight inspection.

Article 11 the Administrative Department of Medical Security shall, in principle, adopt the method of“Double random and one public” to organize and carry out the annual work plan-arranged flight inspections.

Under the circumstances stipulated in subparagraphs 2 to 5 of Article 10 of these measures, inspection may be carried out directly.

Article 12 the Medical Security Administrative Department that organizes flight inspections shall make overall arrangements, provide operational guidance and job security, and dispatch flight inspection teams to carry out on-site inspections.

The flight inspection team shall be composed of administrative law enforcers for medical security and other personnel familiar with medical insurance, medical treatment, medicine, finance, information and other related professions.

Article 13. The administrative department for Medical Security of the Procuratorate shall cooperate with the work related to the flight inspection, and promptly provide truthful, complete and accurate policy documents, data and information, and other relevant materials, according to the needs of the inspection, administrative law enforcement and medical insurance auditors may be dispatched to cooperate with the on-site inspection.

Chapter three check

Article 14 The Flight Inspection Team shall formulate specific implementation plans for flight inspection, make clear the inspection time, method, procedure, key points, standards and methods for determining the objects to be inspected, etc. , and actively study and judge risks, putting forward prevention and control plans as appropriate.

The specific implementation plan shall be submitted to the medical security administrative department that organizes the flight inspection for approval before implementation.

Article 15 after arriving at the inspection site, the flight inspection team shall show the inspected object the law enforcement certificate and related work certificate and serve the inspection notice, informing him of his rights and obligations.

Article 16. The subject under inspection shall cooperate with the flight inspection work and specify the person in charge of the scene, provide real, valid and complete documents, records, bills, vouchers, data, medical records and other relevant materials in a timely manner, and truthfully answer the queries of the flight inspection team, provide explanations and supporting materials to the doubtful data and related questions.

Where necessary, the flight inspection unit may ask other units and individuals concerned with inspection matters for clarification of issues related to inspection matters and for relevant supporting materials. The relevant units and individuals shall cooperate.

Article 17 at least two Inspectors Holding Law Enforcement Certificates shall participate in the on-site inspection. On-site inspection shall be recorded in written or audio-visual form, and the record shall be timely, accurate, complete and effective, and reflect the situation of on-site inspection objectively and truthfully.

On-site inspection shall be made on-site notes, which shall be confirmed by the parties or relevant personnel by means of page-by-page signature or seal. Where an inquiry is made against a relevant person, the inspector shall make a record of the inquiry, which shall be confirmed by signature or imprint of the inquiry object page by page.

Article 18 before the conclusion of the on-site inspection is made, the flight inspection team shall provide information about the inspection to the inspected object. If the inspected objects have objections, they may make representations and pleadings, and supplement relevant materials. The flight inspection team shall record truthfully, examine carefully, study fully, make collective decisions and properly handle disputes.

Article 19 if the flight inspection team fails to cooperate with the inspection of the inspected object, fails to provide relevant materials and information truthfully, and refuses to accept the inspection conclusion without justifiable reasons, it shall record truthfully, and promptly handed over to the procuratorial district medical security administrative departments or other competent departments in accordance with law and regulations for disposal.

Article 20 where the on-site inspection requires an increase in the inspection force or an extension of the inspection time, or where the inspection needs to be suspended or cancelled due to special circumstances, the Flight Inspection Team Shall Report to the administrative department for medical security that organizes the flight inspection for approval.

Article 21 after the on-site inspection, the flight inspection team shall fully communicate with the medical security administrative department of the inspected district on such matters as whether the facts discovered by the inspection are clear, whether the evidence is sufficient, and whether the qualitative and quantitative aspects are accurate, and the relevant laws and regulations as well as the state and the subject of inspection in the unified area of medical security policy as a basis for identifying violations of the law.

If there are major problems in the flight inspection, the flight inspection team shall report them to the administrative department for medical security that organizes the flight inspection in a timely manner.

Article 22 The Flight Inspection Team shall, at the end of the flight inspection, form a written flight inspection report and report it to the medical security administrative department that organizes the flight inspection, and hand over the relevant materials for the flight inspection to the administrative department of Medical Security of the Procuratorate.

The fourth chapter deals with

Article 23. Within 30 working days of receipt of the transferred materials, the administrative department of Medical Security of the procuratorial district shall report the progress of treatment and the plan for rectification to the administrative department of Medical Security that organizes the flight inspection, and submit a written report within five working days of the completion of the processing.

If there is a big difference between the results of the treatment and the results of the examination handed over by the administrative department for Medical Security of the Procuratorial District, a written explanation shall be given.

Article 24 the administrative departments for medical security of the procuratorial district shall require the objects under examination to rectify the problems found in the examination in a timely manner, and deal with the cases of suspected violations of laws and regulations in the feedback opinions in accordance with the law and regulations:

(1) administrative punishment shall be imposed in accordance with the law on any illegal act that does warrant administrative punishment;

(2) those who violate the medical insurance service agreement shall be dealt with by the medical insurance agency in accordance with the agreement

(3) to transfer to the discipline inspection and supervision organs, in accordance with the relevant provisions, the clues to the problems suspected of violating discipline, violating the law by taking advantage of duty or committing crimes by taking advantage of duty;

(4) where the suspected violation of relevant laws, regulations or rules should be handled by other departments, the case shall be transferred to the relevant departments for handling;

(5) other situations requiring handling shall be handled in accordance with relevant regulations.

Article 25 the Medical Security Administrative Department that organizes the flight examination shall incorporate the results of the flight examination into the comprehensive evaluation system for the work of the medical security administrative department of the procuratorial district, and the results of the flight inspection process for supervision, timely organization of strength to carry out flight inspection“Look back.”.

Article 26 in view of the regional, universal or long-standing and relatively prominent problems found in the flight inspection, the medical security administrative department that organizes the flight examination may interview the subject under examination and the person in charge of the relevant medical security department.

The interviewee shall propose rectification measures within 15 working days and report the rectification. Among them, for regional, universal problems, the object of inspection in the local medical security departments should be organized within the jurisdiction of the designated medical institutions to carry out self-examination self-correction.

Article 27 in any of the following circumstances, the personnel participating in the flight inspection shall be punished by the relevant departments according to law and discipline, in accordance with the administrative authority. Those suspected of committing crimes shall be handed over to judicial organs for handling according to law:

(1) violating the spirit of the eight provisions of the Central Committee and its implementing rules;

(2) divulging the relevant information of the flight inspection, the information of the informant, the information of the subject under inspection and commercial secrets;

(III) use the materials and related information obtained and known through inspection for purposes other than supervision and management;

(4) those who have relatives, economic interests and other interests with the subject under inspection or related persons and do not comply with the request for withdrawal;

(5) other violations of laws, regulations and disciplines.

If the irregularities of the personnel participating in the flight inspection cause adverse effects, the medical security administrative department organizing the flight inspection shall notify it within a certain scope.

Article 28 the Medical Security Administrative Department that organizes flight examinations may, when necessary, notify the relevant departments of finance, health, market supervision, traditional Chinese medicine and other relevant departments at the same level of the results of flight examinations.

Article 29 the Administrative Department for medical security that organizes flight examinations shall announce typical cases to the public in a timely manner.

Chapter V Annex

Article 30 in these measures, the administrative department of medical security of the Procuratorate refers to the administrative department of Medical Security at the lower level that organizes the flight examination.

Article 31 the State Medical Security Bureau shall be responsible for the interpretation of these measures. Administrative departments for medical security at or below the prefecture level may organize inspections in accordance with these measures.

Article 32 these measures shall come into effect on May 1,2023.

# April 21,2023

# **Notice on the special rectification work on combating fraud and insurance fraud in the medical insurance field**

All provinces, autonomous regions, municipalities and Xinjiang production and Construction Corps Medical Insurance Bureau, People's Procuratorate, Public Security Department (Bureau) , Finance Department (Bureau) , Health Commission

Now the“2023 health insurance field anti-fraud Special Rectification Work Program” to you, please comply with the implementation.

State health insurance administration

Ministry of Public Security

Supreme People's Procuratorate

Ministry of Finance

National Commission of Health

April 21,2023

(voluntary disclosure)

A 2023 plan to crack down on fraud in health care

In order to implement the decisions and plans of the CPC Central Committee and the State Council, and to organize and implement the year of safety norms for the supervision and administration of medical insurance funds, we will continuously strengthen the supervision and administration of medical insurance funds, the state medical insurance administration, in conjunction with the Supreme People's Procuratorate, the Ministry of Public Security, the Ministry of Finance, and the National Health and Hygiene Commission, has launched a nationwide campaign to crack down on fraudulent insurance schemes and severely crack down on all types of fraudulent insurance schemes, we will earnestly safeguard the safety of medical insurance funds and formulate this plan.

General requirements

Guided by Thought on Socialism with Chinese Characteristics for a New Era, we will fully implement the spirit of the important instructions of the 20th National Congress of the Communist Party of China and the 　 on strengthening the supervision of medical security funds, faithfully perform duties, cooperate closely, carry out in-depth special rectification, investigate and deal with a number of major and important cases, crack down on a number of criminal gangs, constantly improve institutional norms, improve supervision mechanisms, resolutely uphold the safety bottom line of medical insurance funds, and realize, safeguard, and develop the fundamental interests of the overwhelming majority of the people

Basic principles

1) be problem-oriented. Focus on the party Central Committee, the State Council and the People's strong focus on prominent issues, focus on fund supervision key difficult issues, and strive to crack down on the bottom line, repeatedly prohibited fraud insurance.

2) empowering the public with information. Based on the national unified health insurance information platform to build a big data model, screening and analysis of suspicious data clues, and constantly improve the organic integration of off-site supervision and on-site supervision of the overall layout. A mechanism for sharing and evaluating data between departments will be established to crack down on all types of insurance fraud.

(3) adhering to coordination and linkage. To coordinate supervision resources, clarify the division of responsibilities, strengthen the coordination between departments and the interaction between the upper and lower levels, and improve the coordination and supervision mechanism of each department, each responsible for its own duties, each coordinating with the other, and working together to achieve common management, realizing a national“Chess game”

Division of responsibilities

All departments shall carry out special rectification work according to law and duties to ensure the rectification effect.

The health insurance department is responsible for taking the lead in carrying out special rectification, strengthening the strength of personnel, strengthening technical means, and supervising the conduct and expenses of medical services that are covered by the health insurance fund, to investigate and punish illegal use of medical insurance funds. The procuratorial organs shall be responsible for examining, arresting and prosecuting all types of fraud and insurance crimes in accordance with the law, and shall exercise legal supervision over the handling of relevant cases. Considering the need of special rectification, we should push forward the relevant judicial interpretation or guiding opinions in the field of medical security, and further solve the outstanding problems of law application reflected in the judicial practice of fraudulent insurance, and explore the formation of guiding cases or typical cases. The Public Security Department is in charge of cracking down on all kinds of crimes of fraud and insurance fraud, and those suspects who do not constitute criminal punishment in the field of medical insurance and need to be dealt with administratively shall be transferred to the medical insurance department according to law. The financial department shall supervise the use and management of the medical insurance fund according to its duty and assist in the examination of electronic bills for medical charges. The health department is responsible for strengthening the supervision of medical institutions and the medical service industry, supervising and urging medical institutions to standardize their diagnosis and treatment practices, and according to verified circumstances, treating illegal acts committed by medical institutions and related personnel outside the law in accordance with regulations.

All departments should constantly improve the mechanism of coordinated supervision, strengthen the investigation of clues and notification of cases, improve the system of synchronizing and listing major cases for supervision and management, and promote a deep link between administrative law enforcement and criminal justice.

4. measures

Focus on the focus of regulation. According to the regulations on the supervision and administration of the use of the medical security fund, we should crack down on such fraudulent insurance activities as“Fake patients” and“Fake medical conditions”. First, focus on orthopaedics, Blood Purification, cardiovascular medicine, examination, testing, rehabilitation physiotherapy and other key areas. In the fields of orthopaedics, blood purification and cardiovascular internal medicine, combined with the guidelines issued by the State Medical Insurance Bureau on the special examination of high-value medical consumables for orthopaedics, coronary artery intervention treatment and blood purification, combating fraud and insurance fraud. All localities should carry out investigation and rectification in the light of local conditions. In the field of examination, examination and rehabilitation physiotherapy, the typical cases of fraud insurance shall be investigated and dealt with by means of the state flying test and provincial flying test. Second, focus on key drugs and consumables. Make good use of the existing monitoring big data, to monitor the use of the funds for the top-ranking medical consumables in 2022(Annex 1) , and to monitor the abnormal growth of other drugs, consumables, etc. , we should also focus on the analysis of the possible existence of fraud insurance acts, and to crack down severely. Third, we should focus on such key activities as fraudulent medical treatment and medical insurance drug scalping. In particular, we should crack down on institutions and gangs suspected of violating laws and regulations and take effective measures to strengthen supervision over such illegal acts that may easily occur after the implementation of policies such as visiting hospitals in other places and coordinating outpatient services.

2. Strengthen supervision over big data. The National Health Insurance Administration will conduct big data supervision trials to screen for suspicious clues through big data models such as“Fake hospitalizations”, “Insurance drug scalping”, “Insurance e-vouchers cashing out” and“Key drug monitoring and analysis”, on the one hand, we should conscientiously complete the verification tasks issued by the state, check each item, feedback each item and report each level On the other hand, we can combine the local reality, actively carry out big data supervision, targeted screening and analysis

Strengthen the use of inter-departmental data sharing, break down data barriers, and constantly strengthen the data to enhance the level of precision and intelligence.

3. Strengthen publicity and public opinion monitoring. All departments should combine the focus and progress of special rectification work, comb and summarize typical experience, expose typical cases, strengthen warning and deterrence. A public opinion response mechanism should be established to assess public opinion risks and formulate a public opinion response plan

The court shall promptly handle and report any case with significant risk of Olympic situation.

4. Improving the long-term mechanism. All departments should improve the medical insurance fund supervision system throughout the special rectification work, formulate effective measures, and constantly improve the long-term mechanism to combat fraud insurance.

Work arrangement

1. Start the rectification work. The five departments jointly issued a notice on special anti-fraud insurance rectification work, and held a 2023 national anti-fraud insurance special rectification video-teleconference to mobilize the rectification work. (completed in April 2023)

(2) carrying out centralized rectification. We will carry out joint rectification work in accordance with the priorities of the rectification work of the previous year and the relevant laws and regulations. (completed by the end of December 2023)

3. Enhanced reporting of summaries. Provincial health insurance departments should timely sort out the progress of special rectification, analysis of typical cases, strengthen experience summary and timely report. Quarterly Report on the Health Insurance Fund work statistics, in December 2023 Comprehensive Summary Report on special rectification action. (completed by end of December 2023)

Job requirements

The departments of medical insurance, procuratorial work, public security, finance and health care at all levels should fully realize the importance of special rectification, strengthen coordination and linkage, and effectively crack down on fraudulent insurance activities

1. Strengthen organizational leadership. A Leading Group for special rectification work was set up, requiring all units to exercise strict discipline, strictly abide by the provisions of law enforcement, security, confidentiality and integrity, clearly defining the focal points of rectification work, detailing the division of responsibilities, and faithfully performing their duties in accordance with the law

2. Deepening inter-departmental cooperation. We should give full play to the advantages of each department, strengthen the joint efforts of departments, strengthen the investigation of clues, case transfer, etc.

Joint Investigation and Liaison Office, information briefing, etc. . We should strengthen coordination with discipline inspection and supervision departments, actively hand over clues to suspected corruption-related problems, and push forward the fight against fraud and insurance, correct malpractices in the medical field and rectify corruption as one.

3. Strengthen the implementation of responsibilities. We will see to it that work responsibilities are met and an evaluation and assessment mechanism is put in place. The state health insurance administration will link up the implementation of the special rectification work with the Comprehensive Evaluation of the fund supervision, and will notify and commend those places that have done positive work and achieved remarkable results, it will supervise and implement those areas where progress has been slow and responsibility has not been met.

(4) enhanced safeguards. We need to increase support for supervision and inspection agencies, personnel, and vehicles, especially for big data regulation, and promote the development of new tools and methods for regulation, establishing a new pattern for fund regulation.

# May 26,2023

# **Guidelines on strengthening regular supervision of the use of medical insurance funds**

Published by the State Council (2023) on December 17,2010

The People's governments of provinces, autonomous regions and municipalities directly under the central government, the ministries and departments of the State Council, and the agencies directly under the State Council:

The medical insurance fund (hereinafter referred to as the medical insurance fund) is the people's“Medical money”, “Life-saving money. It is of great significance to strengthen the regularization supervision of the use of medical insurance funds for ensuring the safe operation of medical insurance funds, improving the efficiency of fund use, standardizing the medical service behavior and reducing the burden of medical treatment for the masses. In order to further implement the decisions and plans of the CPC Central Committee and the State Council, and steadily push forward the work of regularizing the use of medical insurance funds, with the consent of the State Council, the following opinions are put forward.

General requirements

Guided by Thought on Socialism with Chinese Characteristics for a New Era, we will fully implement the spirit of the 20th National Congress of the Communist Party of China, conscientiously practice the people-centered development philosophy, base ourselves on the new development stage, completely, accurately and comprehensively implement the new development concept, accelerate the construction of the new development pattern, strive to promote high-quality development, solidly promote administration according to law, strengthen the supervision and law enforcement of medical insurance funds, effectively implement the supervision responsibilities of all parties, strengthen the construction of fund supervision capacity, comprehensively use various supervision methods, constantly improve the long-term supervision mechanism, accelerate the construction of a normalized supervision system for the use of medical insurance funds with clear rights and responsibilities, strict and powerful, safe and standardized legal efficiency, and resolutely guard the safety bottom line of medical insurance funds.

2. Clarifying the responsibilities of all parties

(1) Strengthening the supervisory responsibility of the administrative departments for medical insurance. Medical insurance administrative departments at all levels should strengthen supervision over the signing and implementation of medical insurance agreements of medical insurance agencies, and promote the business norms of medical insurance agencies. We will strengthen supervision over the medical services, medical expenses, and the use of medical insurance funds by designated medical institutions. The state health insurance administration is responsible for supervising and guiding the regularization of the use of national health insurance funds, and the provincial health insurance administrative departments are responsible for supervising and guiding the regularization of the use of health insurance funds within their respective administrative areas, health insurance administrative departments at or below the prefecture level shall carry out the task of regular supervision.

(2) to strengthen the responsibility of the medical insurance agencies for auditing and examination. Administrative Departments of medical insurance at all levels should urge medical insurance agencies to establish sound business, financial, security and risk management systems, and strengthen internal whole-process management. The medical insurance agencies should improve their daily auditing ability, and strengthen the auditing of medical insurance expense declaration and medical expense reimbursement of insured persons in designated medical institutions. After the medical insurance agencies examine the medical expenses of the insured through intelligent examination, the medical insurance funds shall settle and pay the expenses in time according to the prescribed time limit. Verification shall be carried out on the compliance of designated medical institutions with medical insurance agreements, the implementation of medical insurance reimbursement policies, and the enjoyment of medical insurance benefits by insured persons. Those who terminate or rescind insurance agreements should report to the health insurance administration in a timely manner. If the problem clues discovered or received shall be handled by the administrative department of Medical Insurance, they shall be handed over for handling in a timely manner.

(3) strengthening the principal responsibility of self-management of designated medical institutions. Designated medical institutions should establish and improve internal management systems related to the use of medical insurance funds, use medical insurance funds in a rational and standardized manner, and make it clear that special institutions or personnel are responsible for the use and management of medical insurance funds, organize training on policies and regulations related to medical insurance fund as required, conduct self-examination and self-correction in time, cooperate with medical insurance department audit and supervision. We will strengthen standardized management of medical services, and do a good job in verifying the medical insurance status of patients and drug buyers, identifying the application of medical insurance catalogues, and recording and archiving inspection reports. The medical institutions led by close medical consortia should carry out their internal management responsibilities and strengthen the management of the use of medical insurance funds.

(4) to strengthen the responsibilities of heads of industry departments. The departments of health, Traditional Chinese medicine, market supervision, drug supervision and auditing shall, in accordance with their respective responsibilities, carry out the relevant supervision responsibilities. Focusing on such illegal issues as over-treatment, fraudulent insurance policies, and illegal purchase and sale of drugs purchased through fraudulent insurance policies, we will continue to strengthen the supervision of pharmaceutical institutions and standardize medical service practices, • Enhancing Education on Professional Ethics and ethics for medical personnel. We will strengthen supervision and inspection of the prices of medical services, curb arbitrary charges and safeguard the rights and interests of consumers. Institutions that are not covered by health insurance agreements but whose actions are closely related to the use of health insurance funds and affect the rational use of funds should follow the principle of“Who approves, who supervises, who is in charge, who supervises”, implement regulatory responsibilities.

(5) to strengthen the responsibility of local governments for supervision over their localities. The local people's governments at all levels shall take the lead in the regular supervision of the use of medical insurance funds within their respective administrative regions, coordinate the resources of various departments within the regions, and form supervision synergy. We will further improve the regulatory mechanism and law enforcement system for the use of health insurance funds, organize and urge relevant departments and people's governments at lower levels to conscientiously perform their regulatory duties, strengthen the building of regulatory capacity, and actively promote integrated cross-departmental supervision, timely coordination to solve the major issues in the work of supervision, to provide a strong guarantee for the use of regular supervision of health insurance funds.

Third, do the actual normal supervision

(1) to normalize the flight inspection. A joint inspection mechanism will be established and improved, and flight inspection plans will be formulated and made public. Improve the management of flight inspection, detailed operating procedures, standardized flight inspection and follow-up treatment, the establishment of annual announcement of flight inspection and exposure of typical cases system. Give full play to the leading role of flight inspection, make good use of the results of flight inspection, focus on typical, stubborn and complex violations of laws and regulations, and compile and establish a list of problems found in flight inspection in a timely manner, it will provide reference for strengthening day-to-day supervision and preventing the recurrence of similar problems in a systematic manner.

(2) promoting the normalization of special rectification projects. We will strengthen joint efforts in cross-departmental oversight, strengthen coordination and interaction among health insurance, public security, finance, health care, and market oversight, and carry out special rectification actions on a regular basis. Focusing on key areas, key institutions and key behaviors, strengthening inter-departmental data sharing and monitoring and analysis, strengthening the notification of case clues, improving the mechanism for connecting executions, and improving the system of synchronizing the filing and listing of major cases for supervision and management, we will actively launch joint law enforcement efforts by departments, and form a joint disciplinary mechanism that involves investigating more than one case and dealing with more than one case. We will promote the transformation of the results of special rectification work into effective experience and regulatory standards, push forward the improvement of medical service prices and medical insurance payment policies, and establish and improve relevant mechanisms.

(3) normalizing day-to-day supervision. To study and formulate methods for daily supervision over the use of medical insurance funds, improve and perfect working mechanisms, and refine norms and requirements for supervision and inspection. Issue a unified and clear list of supervision and inspection items, inspection guidelines, and so on, to improve the day-to-day level of standardization of supervision. Rationally formulate and strictly implement the annual supervision and inspection plan, and strengthen on-site inspection for designated medical institutions with abnormal data indicators, on-site verification shall be carried out on the problem clues assigned by higher-level departments and the designated medical institutions involved in reporting complaints, and the cases shall be dealt with in accordance with laws and regulations. We will strengthen the verification of payments made by the medical insurance agencies, and ensure that all routine checks are carried out in full.

(4) promote the normalization of intelligent monitoring. Relying on the unified national health insurance information platform, making full use of the health insurance intelligent supervision subsystem, establishing a command and dispatch platform for the whole process of administrative inspection and law enforcement, and strengthening the real-time and dynamic tracking of the use of health insurance funds, realize in advance to remind, audit, after the whole process of intelligent monitoring, improve precision, intelligent level. We will speed up the construction and application of the health insurance fund intelligent monitoring knowledge base and rule base, strengthen dynamic maintenance and upgrading, and constantly enhance the effectiveness of intelligent monitoring. Implementing the National Health Insurance anti-fraud Intelligent Monitoring Project, normalizing the screening and analysis of health insurance data, and using big data analysis to lock in illegal and illegal acts of using health insurance funds and discover patterns of fraudulent insurance acts, targeted efforts will be made to step up macro-control, on-the-spot inspection and law enforcement, and crack down on fraud.

5. Promoting the normalization of social supervision. We will further improve the complaint reporting mechanism, rely on the National Health Insurance Fund complaint management system, unblock the channels for complaints, standardize the handling process, and strictly verify and handle them. We will implement a reward system for reporting complaints and mobilize the public to participate in the monitoring of the use of health insurance funds. We will continue to expose typical cases and strengthen warnings and deterrence. We will explore a system for publicizing the use of health insurance funds by designated medical institutions to the public and encourage social supervision.

Fourth, improve the system and mechanism

1. Improve the regulatory system and mechanism. We will further improve the mechanism for checking up and cross-checking, and address difficult issues in supervisory work at the same level. We will establish a system of random checks, backward checks, and accountability, and see that oversight responsibilities are consolidated. Measures will be implemented on a case-by-case basis, using a combination of protocol, administrative and judicial means. For the existence of subjective intent, the impact of Bad Fraud Insurance Act, in accordance with the law from a serious investigation, while doing a good job deal with the effective interface between administrative penalties. To establish and improve the regulatory mechanism of both incentive and constraint, and to stimulate the internal motivation of the standardized use of health insurance funds in medical institutions.

(2) improving the inter-departmental coordinated supervision mechanism. To strengthen coordination between the health insurance departments and the departments of Public Security, finance, health care, traditional Chinese medicine, market supervision and drug supervision, and promote information sharing, we will promote the exchange of clues, mutual recognition of standards, and results between government departments. We will strengthen effective coordination between administrative law enforcement and criminal justice before, during and after the event, and crack down on illegal and criminal acts in the field of medical insurance in accordance with the law. Prompt referral of problem leads concerning suspected disciplinary violations, duty-related crimes and duty-related crimes to disciplinary inspection and supervision organs; establishment and improvement of important leads, joint investigation and investigation of major cases, and accountability mechanisms; and enhancement of the deterrent effect.

(3) establish and improve the credit management system. We will promote the classified management of the credit ratings of designated medical institutions, pharmaceutical enterprises, and personnel, explore the establishment of a commitment system for the monitoring and disclosure of medical insurance funds, and incorporate the implementation of commitments into credit records, linked with the frequency of supervision and inspection, and the discretion of punishment, it will encourage designated medical institutions to regulate the use of medical insurance funds through self-examination and self-correction, and actively fulfill the main responsibility for the use of medical insurance funds. According to the credit rating, the designated medical institutions can take disciplinary measures in terms of fund settlement through agreement management, and the relevant responsible personnel can be suspended from medical insurance payment according to the medical insurance agreement For dishonest pharmaceutical enterprises, measures may be taken to deal with such aspects as access to medical insurance catalogues, price recruitment and credit evaluation, centralized medical and pharmaceutical procurement, online qualification, etc. , measures such as suspending network settlement of medical expenses may be taken. We will strengthen joint incentives and penalties for breaking promises across industries, fields and departments, and explore the establishment of mechanisms such as credit repair and appeal against objections. We will encourage industry associations to develop industry norms and self-discipline and promote industry norms and self-regulation.

(4) establishing a mechanism for cross-regional supervision of medical treatment in different places. We will innovate ways and means to improve the cooperative supervision system and cross-regional working mechanism for medical care in different places, and carry out supervision responsibilities in the places where medical care is sought and insured. The administrative departments of medical insurance at all levels should focus on the flight examination and daily supervision, and guard against the risks of fraudulent insurance.

5. Establishing and improving a mechanism for handling major issues. We will strengthen the daily reporting of regulatory information, carry out early-warning monitoring and research in advance, improve procedures for handling and responding, strengthen targeted training, and enhance the ability of administrative departments at all levels to handle major issues. If the health insurance fund supervision policy is not implemented in place, serious problems arise in the supervision of the health insurance fund, or serious hidden risks exist, the state health insurance bureau may adopt the methods of letter inquiry or interview, etc. , to supervise and guide the relevant medical insurance administrative departments and designated medical institutions to strictly carry out the relevant responsibilities and do a good job of rectification and implementation.

Fifth, strengthen safeguard measures

(1) strengthen the organization and implementation. All regions and departments concerned should fully understand the importance of regularizing the use of health insurance funds, strengthen organizational leadership, refine objectives and tasks, clarify the division of duties and responsibilities, strengthen the implementation of responsibilities, and coordinate the implementation of various tasks. Departments should improve the linkage mechanism and work in coordination to regularize the supervision of the use of health insurance funds.

(2) enhancing regulatory capacity. All regions and departments concerned should increase the support of personnel, vehicles, equipment, technology, funds and other aspects to provide a strong guarantee for the supervision of health insurance funds. We will establish and improve systems for checking work attendance and promoting supervisors, strengthen the training of supervisors in their professional competence, carry out regular training in policies and regulations, and focus on building a multi-disciplinary supervisory team, we will continue to improve the rule of law, standardization, and specialization.

(3) strengthening accountability. The local people's governments at or above the county level shall earnestly perform their supervisory duties, and shall not exercise effective supervision or strict law enforcement, which may lead to the existence of major hidden risks or serious consequences for the safety of the medical insurance funds, and those who abuse their power, neglect their duties or commit malpractices for selfish ends in their supervisory work shall be seriously investigated for their responsibility. The administrative departments of medical insurance at all levels shall establish and improve the comprehensive evaluation system for the supervision of medical insurance funds, and regularly report the progress of the supervision of funds. Actively explore the establishment of accountability, due diligence exemption items list, detailed accountability exemption situation, do a good job of error correction.

(4) do a good job in publicity and education. All regions and departments concerned should step up publicity of policies and regulations on the supervision of medical insurance funds, make full use of various media platforms, and strengthen the interpretation of policies on the supervision of medical insurance funds. We will continue to do a good job in the central publicity month, focusing on such topics as combating fraud and insurance fraud, and carry out regular publicity and education on safeguarding the safety of medical insurance funds, so as to create a good atmosphere for public opinion.

State Council General Office

May 26,2023

# July 14,2023

# **Notice on launching flight inspection of Medical Insurance Fund in 2023**

The medical insurance bureaus, finance bureaus, health committees and Chinese medicine bureaus of all provinces, autonomous regions, municipalities directly under the central government and the Xinjiang Production and Construction Corps:

We are now issuing to you the programme of work for the medical security fund flight check for 2023, to be carried out accordingly.

State health insurance administration

National Commission of Health

Ministry of Finance

State administration of traditional Chinese medicine

(voluntary disclosure)

July 14,2023

2023 programme of work for the flight inspection of the medical security fund

To fully implement the decisions and plans of the CPC Central Committee and the State Council, crack down on illegal and illegal activities in the field of medical insurance, and resolutely protect the people's“Money for medical treatment” and“Life-saving money”, the State Medical Insurance Bureau, the Ministry of Finance, the State Commission of Health and the state administration of traditional Chinese medicine have decided to organize and carry out nationwide flight examinations for the medical insurance fund in 2023. The following work plans have been formulated

General requirements

Guided by Thought on Socialism with Chinese Characteristics for a New Era, we will fully implement the spirit of the 20th National Congress of the Communist Party of China, adhere to a problem orientation, carry out unannounced inspections closely around key areas of the use of medical insurance funds of social concern, and effectively safeguard the health rights and interests of the people; Adhere to laws and regulations, clarify the inspection contents, inspection methods, code of conduct, result judgment, follow-up disposal, etc., and improve the level of legalization, specialization and standardization of unannounced inspections; Adhere to the concept of system, take unannounced inspection as an important starting point for improving the supervision system of medical insurance funds, strengthen the organic connection with other supervision and inspection methods, and systematically promote the supervision of medical insurance funds; Adhere to coordinated governance, improve the working mechanism, improve the institutional system, and promote the high-quality development of medical security and medical and health undertakings in response to the weak links and institutional shortcomings found in the inspection

Second, the work goal

By thoroughly investigating all kinds of illegal and illegal acts in the field of medical insurance, we will further consolidate the principal responsibility of designated medical institutions for rational and standardized use of medical insurance funds, and promote the healthy and orderly development of the medical industry, to improve the people's sense of getting medical treatment, to optimize the policies of medical and medical insurance service, to strengthen the responsibility of examination and examination of the management organizations, to promote the construction of medical insurance informationization and standardization, • Further improving the management capacity of health insurance funds.

Check the object and content

Continue to focus on the focus, this year selected medical imaging examination, clinical examination, rehabilitation as the three areas of examination focus. The review covers the period from January 1,2021 to December 31,2022 for the use and management of the health insurance fund and, if necessary, can be carried out retroactively in previous years or extended to 2023.

(1) check the object

1. The city under investigation. The state medical insurance administration, in collaboration with relevant departments, will select cities with large medical insurance fund usage (generally prefecture-level cities) as the cities to be examined, and municipalities directly under the central government as the cities to be examined

2. Units inspected. Two hospitals and one pharmacy were selected by the flight inspection team from the top-ranked fixed-point medical institutions in the inspected cities, and the inspected units were taken together with the municipal medical insurance agencies. They could also determine the units directly based on clues from reports and suspicious points of intelligent monitoring. In principle, institutions that have previously received national flight inspections will no longer be regarded as subject to inspection.

(2) contents of inspections

1. Targeting targeted medical institutions. Including medical insurance internal control management, financial management, drugs, consumables centralized belt procurement implementation, the National Unified Medical Insurance Information Service Code application situation as well as the medical service behavior and the charge behavior involved in the medical insurance fund use process.

2. Targeting targeted retail pharmacies. These include replacing medicines or other commodities that are not paid for by the health insurance fund with medical insurance medicines, airbrushing and stealing medical insurance vouchers, forging and altering medical insurance medicines' “Import, sale and deposit”: Bills and accounts, falsification of prescription or insurance personnel expense lists, non-designated retail pharmacies, designated retail pharmacies during the suspension of health insurance agreements or other organizations to settle health insurance costs and other acts.

3. Targeting health insurance agencies. Including the audit and settlement of payments with medical institutions, the identification of treatment for out-patients with chronic special diseases, the admission of designated medical institutions, the non-payment of third-party liability medical insurance funds, and the settlement of medical expenses in other places, etc. , dRG/DIP payment method to determine the annual budget, intelligent audit system use, daily verification of the implementation of designated medical institutions, etc. .

Division of responsibilities

The state medical insurance administration, in conjunction with the Ministry of Finance, the State Commission of Health and the state administration of traditional Chinese medicine, and other departments, conducts flight inspections. The responsibilities of the departments are as follows:

(1) medical security departments. The state medical insurance administration, in conjunction with relevant departments, shall determine the inspection focus and formulate the inspection plan according to such factors as the risk assessment of the medical insurance fund, the clues for reporting complaints, the monitoring of medical security data, etc. , to be responsible for guiding and supervising the flight inspection work; to assign staff to participate in the whole flight inspection, to be responsible for communication and coordination of specific work; to summarize the experience and practice of solidification, to analyze and improve the current health insurance policies in the light of the problems found in the inspections. Each provincial-level medical security administrative department appoints the person in charge of the supervision of funds as the team leader, organizes and completes the work of team formation, inspection and implementation, result handling, rectification and implementation, and refers to the national model, we will continue to improve the working mechanism for provincial-level flight inspections and regularize them within the province. The medical security departments will ensure the travel expenses of experts in the flight inspection team

(B) other departments. The Ministry of Finance, the National Health Commission, the state administration of traditional Chinese medicine and other departments may designate department-level cadres as inspectors. The competent department of the industry shall analyze and gradually solve the functional problems of the unit found in the inspection. Each provincial-level department of Finance, health, traditional Chinese medicine and other departments may designate department-level deputy team leaders at the ministry level, select law enforcement personnel according to the inspection focus, and coordinate medical experts to participate in spot inspections of designated medical institutions, to seriously deal with any violations of laws and regulations discovered and transferred during flight inspections

5. Organizational procedures

The model of“Cross-examination among provinces” is adopted to determine the participating provinces and the provinces to be examined by drawing lots. In principle, the number of inspectors in each group should be limited to 60 persons, and the inspection time should be limited to 10 days. From August 2023 to December 2023, it is planned to carry out inspections in all 31 provinces (autonomous regions and municipalities directly under the central government) and Xinjiang production and Construction Corps

(1) flight inspections (July 2023) . The state medical insurance bureau, in conjunction with relevant departments, will study and formulate a unified inspection process, inspection methods, inspection standards and complete the drawing of lots to assign teams. The Provincial Medical Security Administrative Department formulates the implementation plan according to this plan, examines each content in detail, simultaneously completes the training work ahead of time according to the inspection key.

(2) implementation of flight inspection (August 2023-december 2023) . Prior to the on-site inspection, the flight inspection team completed the data screening, and the medical security departments of the area under examination cooperated with the relevant work, providing real, complete and accurate policy documents, data and information in a timely manner. During the on-site inspection, the flight inspection team formed objective and fair written conclusions on the basis of fully listening to the opinions of all parties and transferred the relevant data to the medical security administrative department of the province being examined for follow-up verification. After the on-site inspection, within 5 working days after handling the case, the administrative department of the medical security of the province under examination shall submit a written report to the state medical insurance bureau, and the State Medical Insurance Bureau shall timely organize the strength to“Look Back” on the rectification and rectification of the examined institution to ensure that the rectification and rectification of the problems found by the flight inspection are carried out in place.

(3) summary of flight inspections (Jan-mar 2024) . The State Medical Insurance Bureau forms an annual report on the work of flight inspections. After consulting the departments of finance, health and traditional Chinese medicine, it will, as appropriate, inform the inspection and expose typical cases. At the same time, the overall situation of flight inspection and key areas of inspection methods were summarized, to provide follow-up supervision experience and system norms, to promote the normal day-to-day supervision.

Organizational security

1. Strengthen organizational leadership. Medical Insurance, finance, health care, traditional Chinese medicine and other departments should fully understand the importance of joint medical insurance fund flight inspection work in accordance with the unified deployment, strengthen organization and coordination, strengthen coordination, they should form synergy to ensure the effectiveness of flight inspections.

(2) strictly regulating law enforcement. The inspection shall be carried out in strict accordance with such rules and regulations as the provisional measures for the administration of flight examinations for the medical security fund and the provisional provisions on the procedures for administrative penalties for medical security, to achieve law enforcement procedures norms, law enforcement based on accurate, law enforcement results must strictly implement the“Health Insurance Fund Flight Inspection Code of conduct (trial)”, consciously correct. They should observe political discipline, discipline of integrity, discipline of the masses, discipline of confidentiality, and discipline of work. We should study the examination data issued by the state medical insurance bureau, Grasp the target, emphasis, method and requirement of examination, and ensure the scientific and accurate examination. At the same time, the National Health Insurance Bureau (NHA) set up a questionnaire to evaluate the participation/acceptance of examination of the provincial medical insurance administrative departments, and the results were included in the Comprehensive Evaluation of the NHA fund supervision.

(3) serious follow-up actions. We should make full use of judicial, administrative, and agreement means to deal with serious follow-up, strengthen the coordinated use of the results of flight inspection, and actively play the role of inter-departmental punishment. Those who violate the regulations on the supervision and administration of the use of the medical security fund shall be subject to administrative punishment in accordance with the law Party members, cadres and state functionaries who violate the law and discipline shall be transferred to the discipline inspection and supervision organs. At the same time, greater publicity should be given to cases that are of a bad nature, and the deterrent effect should be actively strengthened. In addition, medical institutions self-examination self-correction found and rectified in place of the problem, can be treated leniently

4. Addressing both symptoms and root causes. In order to extend the effectiveness of the inspection, we should effectively carry out the centralized rectification in the scope of the province in combination with special rectification and daily supervision, aiming at the typical outstanding problems found in the flight inspection, so as to bring about a comprehensive solution to this problem. We need to do a good job in the transformation of results, focusing on the institutional obstacles, institutional flaws and institutional loopholes behind the problems, and focus on informationization, institutionalization and standardization, so as to comprehensively improve the management of health insurance funds, we will strengthen the role of health insurance in guiding and promoting health care and medicine. Credit management should be used to link the results of flight inspections with the management of gross amounts, examination and inspection, and management of fixed-point agreements, so as to strengthen the implementation of the responsibility of the safety subjects of the medical insurance funds of the fixed-point medical institutions, we will push designated medical institutions to establish and improve internal management systems related to the use of medical insurance funds, and take the initiative to manage and make good use of the people's“Medical money” and“Life-saving money.”.

# September 8,2023

# **Notice on further promoting the intelligent audit and monitoring of medical insurance funds**

The medical insurance bureaus of the production and Construction Corps of Xinjiang Autonomous Region, autonomous region and municipalities directly under the central government:

In order to implement the opinions of the CPC Central Committee and the State Council on deepening the reform of the medical security system and the regulations on the supervision and administration of the use of the medical security funds (order of the State Council No. 735) . “Guiding opinions of the State Council General Office on advancing the reform of the regulatory system for medical security funds”(issued by the State Council General Office [2020] No. 20) , and“Opinions of the State Council General Office on strengthening the implementation of the regulation of the use of medical security funds on a regular basis”(issued by the State Council General Office [2023] No. 17) , fully establish an intelligent monitoring system, implement real-time, dynamic and intelligent monitoring of big data, and establish a mechanism for the prevention and control of fund security in advance, during and after the whole process of supervision, we will deepen the application of the smart monitoring subsystem of the national health insurance information platform, further advance smart auditing and monitoring, and tighten the line of defense for fund monitoring. The following is a notice of the relevant work:

Identify your goals and tasks

In 2023, we will focus on the construction of a knowledge base and a rule base (hereinafter referred to as“The two bases”) for intelligent auditing and monitoring of medical security funds, actively adapting to the supervision of funds under the payment mode of DRG/Dip, we will promote the application of the system on the ground, and by the end of 2023, all regions will have an online intelligent monitoring subsystem, and data from intelligent audits and monitoring will be accurately uploaded to the national health insurance information platform, and all areas will have an intelligent audit, we will standardize the service behavior of appointed medical institutions, strengthen the connection between agreement processing and administrative supervision, process verification and administrative law enforcement, and initially realize the“One net” of national intelligent monitoring. By the end of 2025, a standardized, scientific and regular intelligent auditing and monitoring system will be basically established, “The construction and application of the two databases, intelligent auditing and anti-fraud big data intelligent monitoring and analysis will become more mature. We will improve information-based, digital and intelligent auditing and monitoring of health insurance and funds, and form a regular monitoring system that handles daily auditing and on-site verification, big data analysis and all-scene intelligent monitoring, to ensure the safe, efficient and rational use of funds.”.

2. Strengthen the construction and application of the two repositories

1. Accurately grasping the key points of the construction of the“Two reservoirs”. To clarify the construction of the relation rule base of“Two databases”, we should lay stress on straightening out the operation logic of the rules, keeping the reasonable thickness of the rules, regularly analyzing and maintaining them, and strengthening the management of the rule base system. The construction of knowledge base should focus on the accuracy and completeness of the corresponding knowledge points of rules, and maintain and update the intelligent auditing and monitoring engine in time, the construction of“Two repositories” should be strengthened to improve the running efficiency and accuracy of the results of the engine.

(2) give overall regional planning authority to localize the two reservoirs. Provincial and municipal health insurance departments are promoting the simultaneous updating and localization of the“Two reservoirs” under the national“Two reservoirs” framework, and strengthening the scientific and accurate construction and application of the“Two reservoirs”. Health insurance departments at all levels should be given full autonomy to set rules parameters, indicators, thresholds, application scenarios and so on. Provincial health insurance departments should strengthen the management of the rules system, and the rules applied locally should be formed according to the rules logic corresponding to the national“Two banks”, and coded according to the national rules coding standard, local rules that do not correspond to national rules should be locally coded.

(3) scientifically set the index to break the value and gradually go online to apply the rules. The health insurance departments of each co-ordinating region should take into account the operation logic of the rules, the objective of supervision, the intensity of the effect, and the match with the actual working conditions, and urge the designated medical institutions to improve their ability to appeal and review, they should reasonably determine the number of rules, relevant indicators and thresholds, and ensure that each rule is applied as it matures. Dynamically adjust the rules and knowledge in the application to continuously improve the accuracy and effectiveness of the rules. For the rules with high false positive rate, the corresponding knowledge points should be modified in time, the parameters and min values should be adjusted, and the rules with weak practicability should be stopped in time.

(4) continue to make good use of the“Two reservoirs.”. For“Clear violation” of the rules of the operation results, we should strengthen supervision and intervention, to achieve the illegal acts of automatic interception, for“Suspicious” of the rules of the operation results, we should fully listen to the opinions of targeted medical institutions, and establish and open channels for complaints. We should establish a sound management mechanism, formulate procedures for handling suspicious information, communicate fully with designated medical institutions, and handle complaints from medical institutions. It is necessary to evaluate the application effect of rules, strengthen the statistics of the application of various rules, follow up and evaluate the application effect in time, grasp the progressive strength of rules in application, and adjust the equipping of the corresponding knowledge points of rules, to explore the formation of scientific and reasonable regulatory standards, gradually solidify the“Gold standard” of reasonable rules, constantly optimize the application of rules, and enhance the level of“Two storehouses” intelligence.

Optimize the intelligent audit and monitoring process

(1) a scientific classification of work processes before, during and after the event. The basic workflow of Intelligent Audit and monitoring includes data collection and transmission, data comparison, illegal screening, detailed audit, investigation and verification, illegal handling, evaluation and analysis. Taking the audit and settlement of medical insurance fund as the central node, it is divided into pre-warning, in-the-middle audit and post-supervision. Pre-alert is a real-time reminder of the medical service behavior in the designated medical institutions, and pre-trial and pre-warning before uploading the medical insurance fund settlement documents to the agency. In-case audit is the implementation of process control between the occurrence of medical service behavior and the completion of settlement by the agency. In the process, the agency shall audit the settlement documents of the Medical Insurance Fund uploaded by the designated medical institutions, the“Clear violation” of the cost of direct refusal to pay, found suspicious problems, timely feedback to designated medical institutions, by their complaints, after communication feedback to refuse to pay the cost of violations. After-the-fact supervision shall be carried out by the medical insurance administrative department, the fund supervision and management agency, etc. , to verify, supervise and inspect the expenses of the designated medical institutions after completion of settlement, using information technology means such as big data modeling and knowledge mapping to expand the scope and depth of post-incident supervision and verification, and recovering the expenses found in violation of laws and regulations, and dealing with administrative penalties and agreements in accordance with laws and regulations, cases suspected of crimes should be promptly transferred to competent public security organs. Afterwards supervision should focus on big data monitoring and analysis, pay attention to integrity, trend and relevance, draw accurate pictures of all kinds of supervision objects, and provide support for on-site inspection and administrative law enforcement.

(2) encouraging designated medical institutions to carry out advance reminders. The fixed-point medical institutions are encouraged to interface with the intelligent supervision subsystem or embed rules in the system, and carry out advance warning. The key points of warning in advance are strict real-name medical treatment, diagnosis and treatment behavior, standardized charging, early-warning over-prescription, early-warning cost declaration. Through identity identification, video monitoring, strict implementation of real-name medical purchase system to ensure that the witness. Real-time reminders of medical personnel violating policy restrictions and unreasonable examination, diagnosis and treatment, and drug use, and guiding medical personnel to consciously abide by clinical diagnosis and treatment norms and medical insurance management policies, providing medical services and charging fees in accordance with laws, regulations and reasonable norms. Designated medical institutions shall conduct pre-examination and self-examination of the settlement documents of the medical insurance fund before reporting.

4. Promote comprehensive and intelligent auditing by the institutions handling the medical insurance funds

Full coverage of the audit of the settlement documents. The administrative agencies at all levels shall, taking the overall planning area as a unit, use the intelligent supervision subsystem to comprehensively audit the expenses declared by the designated medical institutions, and achieve the full coverage of the intelligent audit by the medical insurance funds, the first line of defense for the safety of health insurance funds must be strictly enforced. For problems that have been warned in advance but are not complied with by the designated medical institutions, the key audit should be conducted manually during the audit stage. Direct settlement of the implementation of the management of medical treatment in different places, medical treatment agencies responsible for auditing the costs of medical treatment in their own jurisdiction.

(2) improve internal management processes. The administrative organs at all levels should establish and improve the management mechanism of the whole audit process, such as on-line initial examination, review, appeal, review, feedback and processing. Clear the audit authority to ensure that the audit process and audit results can be traced, regulate discretion, plug audit loopholes. First Review, review, review post settings should be separated from each other, mutual constraints, mutual supervision, according to the needs of each link of data and information authority control, to establish management systems for data access, operation, maintenance, confidentiality and effective feedback mechanisms for information flow, and to improve the verification and handling mechanisms for intelligent auditing and monitoring of suspicious points, strengthen the communication and feedback with designated medical institutions, improve the process of non-payment and recovery of illegal funds, to ensure that illegal issues are effectively addressed.

3. Strengthen the initiative of coordinating regional and designated medical institutions. It is necessary to strengthen the responsibility and initiative of regional users, and give the regional health insurance departments full autonomy in the selection of rules, engine invocation, audit process, and the establishment of Audit Authority. We should strengthen the management of intelligent audit and monitoring, combine it with the work of protocol management, and take effective measures to encourage the appointed medical institutions to link up the intelligent monitoring subsystem, the system connection situation and implementation, the annual assessment of designated medical institutions should be linked. To explore the policy of reducing the frequency of on-site inspection for the medical institutions that actively strengthen the application of intelligent supervision system and carry out self-examination and self-correction.

5. expand the application scenarios of intelligent monitoring

1. Doing a good job in the supervision of designated medical institutions through the reform of the outpatient ataxia security mechanism. In view of the new characteristics of fund use and the behavior of the objects under supervision in outpatient ataxia, we should adopt the abnormal behaviors such as frequent out-patient service, frequent drug purchase, diagnosis that is not in accordance with the patient's sex or age, over-prescribing of drugs, etc. , lock high-risk medical insurance accounts and personnel, and thus identify relevant institutions and personnel violations. To promote designated retail pharmacies to the health insurance department to upload drug“Sales and storage” data, health insurance expenses details and other information, to ensure that the upload data comprehensive, accurate and timely. It is necessary to carry out pre-warning, mid-audit and post-supervision on the outpatient medical service behavior of the designated medical institutions. If conditions permit, areas should explore and strengthen scene monitoring of designated retail pharmacies. Explore establishing price monitoring mechanisms, strengthening the linkage of settlement prices (2) strengthening the intelligent auditing and monitoring of the health insurance funds under the DRG/DIP payment mode, making full use of the rules such as abnormal information in the health insurance settlement list, abnormal information in settlement details, abnormal coding of disease diagnosis, abnormal coding of operation operation, non-conformity of operation coding with gender, non-conformity of diagnosis coding with operation coding, etc. , the quality of DRG/DIP data is continuously improved by checking the relationship between diagnostic codes and surgical operation codes. We should pay attention to DRG/DIP payment regulation, strengthen the research and development of regulation rules such as high-rely grouping, low-bid admission, decomposition of hospitalization, transfer of fees and so on. At the same time, through the comparison and analysis of the cost of the disease and the monitoring of the key disease, we found that the medical service was insufficient and the behavior of the high staff was doubtful.

(3) applying new technologies to comprehensively empower fund supervision. Relying on the medical insurance electronic certificate face recognition technology to carry out insurance participants, medical insurance physician identity authenticity authentication. To strengthen the application of new technology in intelligent monitoring, to promote artificial intelligence to the medical security supervision, to achieve all kinds of doubt automatic capture, intelligent research and early warning. Based on big data technology to promote data exchange and scene interconnection, to explore patient-centered collection of diagnostic and treatment data, and to establish a big data model of related topics, strengthen the high-risk groups, institutions of the integrity of the portrait and fraud insurance risk identification. To carry out the comparative analysis of the medical treatment behavior and expense data between the medical treatment in different places and local medical treatment, and to strengthen the supervision of the medical treatment fund in different places to integrate the means of medical insurance intelligent monitoring and big data analysis, to realize clue discovery, investigation and evidence collection, illegal handling, application of results and other regulatory links online and offline combined to form the whole process of closed-loop supervision.

Job requirements

1. Strengthen organizational leadership. The medical insurance departments at all levels should fully understand the important role of promoting the intelligent monitoring of medical insurance in promoting the efficiency of the supervision of medical insurance funds, and strengthen the responsibility of the organization and leadership of the provincial medical insurance departments and the overall planning of regional users, • make unified arrangements for implementation and promote implementation in a coordinated manner. We should increase coordination in administrative supervision, it application, management and implementation. The Administrative Supervision Department should take the lead in promoting the construction of“Two storehouses” and ensure the actual application effect. Information construction departments to do a good job of system construction, data support, fully ensure that business needs landing. Operating agencies should use the intelligent supervision subsystem to carry out intelligent audit, real-time evaluation of the feedback“Two-library” application effect, and constantly optimize the iteration“Two-library”. We should actively link up public security, health, Chinese Medicine Administration, drug supervision, civil affairs, Market Supervision and other relevant departments, and strengthen information exchange and data to achieve joint response and sharing.

(2) improving the working mechanism. The state medical insurance administration will regularly dispatch the intelligence audit and monitoring of various localities, report the progress of the work, and incorporate the application effectiveness of intelligence audit and monitoring into the annual Comprehensive Evaluation of fund supervision as the key evaluation indicators. Each year, we select 30-50 areas with good application results as“Intelligent Audit and monitoring technology demonstration”, extract typical practices and effective experience, timely summary and promotion, improve the system's intelligent monitoring work. The provincial-level health insurance departments shall guide the health insurance departments at all levels within their jurisdictions to establish an intelligent management mechanism for auditing and monitoring work, strengthen work coordination and dispatch notification, and designate special personnel to engage in intelligent supervision and related work, to strengthen the training of professional skills, focusing on the cultivation of intelligent audit and monitoring business backbone in all areas. To improve the management mechanism of rules and knowledge release, business personnel and information personnel double account, double check management, shall not be the rules, knowledge to stop the use of authority to a third party to exercise alone.

(3) strengthen the data base. The provincial-level health insurance departments shall follow the standardized data application model proposed in the circular on further clarifying the data application model for municipal and county-level health insurance departments (ICAO -LNo. no. 202212]) , to provide data support for regional health insurance intelligent audit and monitoring, big data analysis, to fully protect the overall use of the region, analysis of the region's health insurance settlement data authority. To strengthen governance at the source of data, urge designated medical institutions to fill in the“Medical Security Fund settlement list” in a standardized manner, and upload the full amount of data needed for protocol management and fund supervision, and strengthening the verification function of the health insurance information platform's checklists to enhance the accuracy and comprehensiveness of data. Improve data security mechanism, strictly in accordance with relevant laws and regulations, improve information security protection and emergency handling capacity to ensure network information security.

(4) do a good job of communication and publicity. Health insurance departments at all levels to do a good job in coordination, strengthen communication and interaction, timely solution to work problems encountered. We will intensify policy advocacy, strengthen communication with designated medical institutions, take the initiative to respond to social concerns, and create a good atmosphere for the whole society to pay attention to and consciously safeguard the safety of medical insurance funds. The state health insurance administration will promptly report any problems arising from its intelligent audit and monitoring.

National Health Insurance Administration

September 8,2023
